# Emission color-tunable oxazol(in)yl-substituted excited-state intramolecular proton transfer (ESIPT)-based luminophores†

**DOI:** 10.1039/d0cc05780k

**Published:** 2020-11-10

**Authors:** Dominik Göbel, Pascal Rusch, Daniel Duvinage, Nadja C. Bigall, Boris J. Nachtsheim

**Affiliations:** Institute for Organic and Analytical Chemistry, University of Bremen Leobener Straße NW2 D-28359 Bremen Germany nachtsheim@uni-bremen.de; Leibniz University Hannover, Institute of Physical Chemistry and Electrochemistry Callinstraße 3a D-30167 Hannover Germany nadja.bigall@pci.uni-hannover.de; Cluster of Excellence PhoenixD (Photonics, Optics, and Engineering – Innovation Across Disciplines) Hannover Germany; Institute for Inorganic and Crystallographic Chemistry, University of Bremen Leobener Straße NW2 D-28359 Bremen Germany

## Abstract

Oxazolinyl- and arylchalcogenazolyl-substituted hydroxyfluorenes exhibiting excited-state intramolecular proton transfer (ESIPT) are described as potent and highly modular luminophores. Emission color tuning was achieved by varying the π-expansion and the insertion of different chalcogen atoms.

## Abstract 2

Oxazolinyl- and arylchalcogenazolyl-substituted hydroxyfluorenes exhibiting excited-state intramolecular proton transfer (ESIPT) are described as potent and highly modular luminophores.

## Graphical abstract

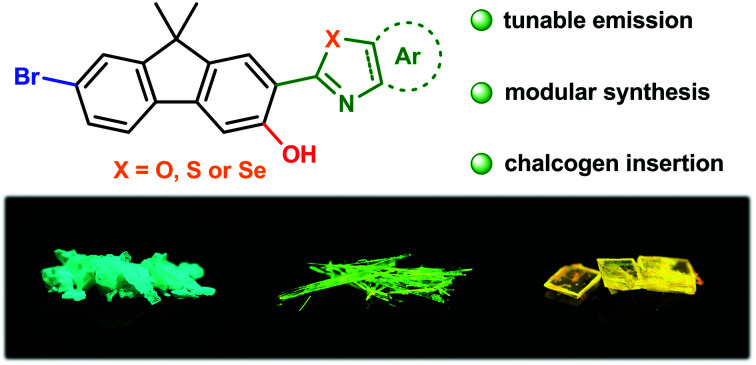

Excited-state intramolecular proton transfer (ESIPT)-based luminophores have attracted great attention due to their remarkable characteristics like large Stokes shifts (up to 12.000 cm^−1^) and dual emission^1^ behavior leading to white light generation.^2,3^ These properties derive from a four-level enol–keto-phototautomerism cycle, which comprise light absorption of the enol form, an ultrafast tautomerization,^4^ emission of the keto form and a cycle-completing second proton transfer. Extended application in electroluminescent devices,^5^ chemical sensing^6^ and biological imaging^7^ have been described. ESIPT-based fluorophores have a hydrogen bond donor in close proximity to a proton acceptor within an arene. While hydroxy groups are typically used as the hydrogen donors, the design of the proton acceptor is multifarious. Carbonyl groups,^3,8^ imines^9^ and benzazoles^10^ are well investigated proton acceptors, while the latter scaffold is widely used owing to its versatile synthesis and robustness towards harsh reaction conditions. Recently we demonstrated the efficiency of oxazolines as proton acceptors in minimalistic ESIPT-based fluorophores.^11^ While in these initial investigation simple mono- and benzo-fused cyclic arenes were used as the underlying π-scaffold, we were intended to expand oxazoline and oxazole-based ESIPT luminophores on fluorenes^12^ and other established fluorophores. Herein such oxazolinyl- and arylchalcogenazolyl-substituted hydroxyfluorenes as potent fluorophores (Fig. 1) are communicated. Seven fluorenes were synthesized and analyzed regarding their photophysical properties and crystal structures to gain a deep understanding towards their structure–property relationship.

**Fig. 1:**
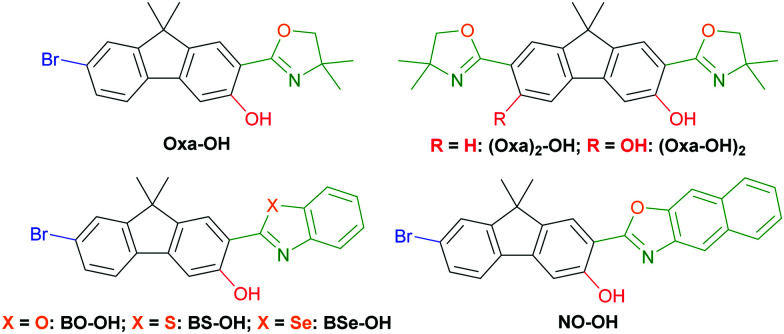
Chemical structures of hydroxylated fluorenes.

Oxazoline-substituted hydroxyfluorenes **Oxa-OH**, **(Oxa)2-OH** and **(Oxa-OH)2** were synthesized according to our previously reported procedures in very good yields.^13,14^ Hydroxylated benzoxazole (**BO-OH**), naphthoxazole (**NO-OH**), benzothiazole (**BS-OH**) and benzoselenazole (**BSe-OH**) were achieved using either a deprotonative metalation/aerobic hydroxylation method^14^ or a Pd-catalyzed hydroxylation with 1,4-dioxane as oxygen source.^15^

The optical properties of the respective precursor oxazolinyl- and arylchalcogenazolyl-substituted fluorenes were examined in different solvents (see the ESI,† chapter 3 and Table S2) as well as the final hydroxylated compounds which were additionally analyzed in their crystalline form. In the following, only the emission properties of hydroxylated fluorenes are discussed with a focus on the solid state (Table 1 and Table S3, ESI†).

**Table 1:** Photophysical data of **Oxa-OH**, **(Oxa)2-OH**, **(Oxa-OH)2**, **BO-OH**, **NO-OH**, **BS-OH**, **BSe-OH** and BSe-OH* in the solid-state at 295 K^a^

Compd	*λ*_exc_ b [nm]	*λ*_em_ [nm]	Δ*ν̃*^b^ [cm^−1^]	*Φ*_F_ [%]	*Φ*_F_ c [%]	*τ* [ns] (Rel%)^d^	*k*_r_ e [10^8^ s^−1^]	*k*_nr_ e [10^8^ s^−1^]	*x*;*y* (CIE 1931)
**Oxa-OH**	364	481	6680	38	17	2.9	1.32	2.13	0.160; 0.358
**(Oxa)2-OH**	370	485	6410	11	10	2.1	0.54	4.22	0.177; 0.420
**(Oxa-OH)2**	380	493	6030	18	11	2.3	0.77	3.57	0.187; 0.469
**BO-OH**	397	(452),^f^ 520	5960	37	34	1.6 (28); 4.3 (72)	1.04	1.78	0.259; 0.494
**NO-OH**	369	506, (546)^g^	7340	3	38	1.6 (62); 3.8 (38)	0.11	3.99	0.302; 0.554
**BS-OH**	450	575	4830	39	16	7.3	0.54	0.83	0.487; 0.501
**BSe-OH**	450	566	4550	7	6	1.7	0.41	5.47	0.459; 0.526
BSe-OH*	435	579	5720	7		1.8	0.39	5.04	0.510; 0.483

a Additional spectroscopic data (samples and non-hydroxylated precursors in solution) is given in the ESI.

b Excitation spectra of the crystalline samples were used for evaluation of the Stokes shift as absorption could not be measured due to low sample amount.

c Quantum yields measured in CHCl_3_ solution.

d Intensity-weighted relative ratios of the two decay components are given in parentheses if a bi-exponential fit was used.

e *k*_r_ and *k*_nr_ were calculated using the equations *k*_r_ = *Φ*_F_/*τ* and *k*_nr_ = (1 − *Φ*_F_)/*τ* using the average lifetime.

f Emission maximum of the enol form is given in parentheses.

g The emission wavelength of the shoulder is given in parentheses. The amorphous sample is marked with an asterisk.

All target structures display emission in solution and solid state (Fig. 2a–e). The emission color of crystals formed from the oxazoline-substituted derivatives are in a close range from blue-cyan (481 nm, **Oxa-OH**) to green-cyan (485 nm, **(Oxa)2-OH**; 493 nm, (**Oxa-OH2**); Fig. 2a, e and Table 1). Switching the acceptor unit to π-extended benzo[*d*]oxazoles resulted in a bathochromic shift of *λ*_em_ to 520 nm for **BO-OH** and 506/546 nm for **NO-OH** (Fig. 2b, e and Table 1) and thus green to green-yellow emission. A more significant red-shift was observed when the oxygen of the benzo[*d*]oxazoles was substituted against heavier chalcogens. The corresponding benzothiazole (**BS-OH**) and benzoselenazole (**BSe-OH**) exhibit *λ*_em_ of 575 nm and 566 nm (Fig. 2c, e and Table 1). Interestingly, as the only substrate, amorphous **BSe-OH** exhibits a significant difference in emission color, since the keto emission band is broadened compared to crystalline **BSe-OH** resulting in a further bathochromic emission shift to 579 nm (Fig. 2d, e and Table 1). These observations indicate a continuous red-shift of emission maxima upon (1) π-expansion of the proton acceptor and (2) varying the heteroatom in the azole ring by walking down the chalcogenic group. This bathochromic shift can be explained by a decreased HOMO–LUMO gap.^16^

**Fig. 2:**
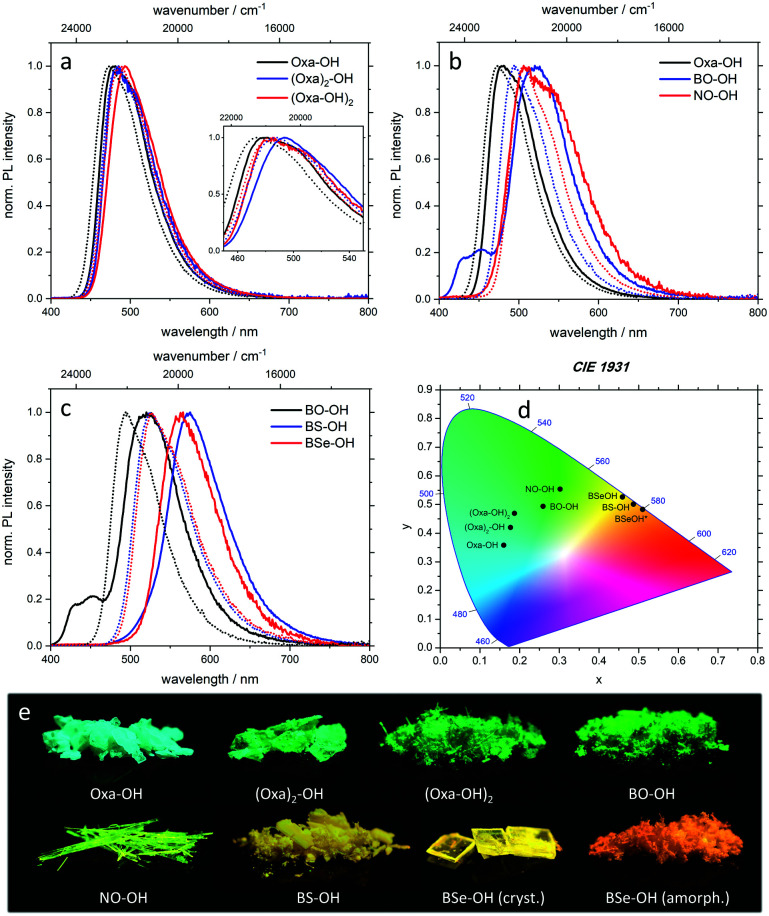
(a–c) Normalized emission spectra of hydroxylated fluorenes measured for crystalline samples (solid lines) and as solution in chloroform (dotted lines). (d) CIE 1931 chromaticity plot with emission color coordinates of fluorenes in the solid-state. The amorphous sample is marked with an asterisk. (e) Representative images of hydroxylated fluorenes in the solid-state under 366 nm irradiation.

In order to compare the efficiency of the presented luminophores, the photoluminescence quantum yield *Φ*_F_ was determined in the crystalline state. With *Φ*_F_ = 38%, **Oxa-OH** has the highest emission efficiency of all oxazolinyl-substituted fluorenes. Interestingly, substitution of the bromine atom on the fluorene scaffold by a second oxazoline group (**(Oxa2)-OH**, 11%) leads to a decreased *Φ*_F_. Since heavy atoms like bromine facilitate intersystem crossing processes,^17^ the absence of such substituents should significantly increase *Φ*_F_. Incorporation of a second hydroxy group increased the emission efficiency slightly to 18% for **(Oxa-OH)2**. **BO-OH** and **BS-OH** exhibit almost similar quantum yields of 37% and 39%. A diminished *Φ*_F_ was observed for π-expanded **NO-OH** (3%) and heavy atom incorporated **BSe-OH** (7%). Despite the possibility of phosphorescence generation through selenium incorporation, no long living species were detected in our studies. However, fluorescence lifetimes (*τ*) of the excited states were determined by time-correlated single photon counting (TCSPC), with values ranging from 1.7 ns for **BSe-OH** to 7.3 ns for **BS-OH** (Table 1 and Table S3, ESI†).

Due to the ESIPT character of the presented compounds, a large Stokes shift is expected. This is indeed the case with Stokes shifts ranging from 4550 cm^−1^ for **BSe-OH** to 7340 cm^−1^ for **NO-OH** in the solid state. Thus, efficient proton transfer in the excited state takes place resulting in exclusive keto emission. Also, the Stokes shift of the target structures in solution is in all cases significantly larger than the Stokes shift of the respective compounds prior to hydroxylation (see the ESI,† Tables S2 and S3). Apart from the large Stokes shift, ESIPT also manifests in dual emission behavior. For crystalline samples, only **BO-OH** exhibits a minor maximum at 452 nm, which is attributed to enol emission (Fig. 2b, c and Table 1). In case of diluted hydroxylated fluorenes, enol emission features are only detected for **Oxa-OH**, **NO-OH** and **BS-OH** in definite solvents (Fig. S3, S13 and S18, ESI†).

In addition, temperature-dependent emission measurements (from 77–200 K) were performed for **BO-OH**, **BS-OH** and **BSe-OH** (Fig. S15, S24 and S29, ESI†). In case of **BO-OH** an increase in luminescence intensity was observed upon cooling with a maximum intensity at 180 K (Fig. S15, ESI†). Such a behaviour is common for ESIPT luminophores, due to suppression of the proton transfer at deep temperatures.^11,18^**BS-OH** and **BSe-OH**, however, showed a steady increase of intensity during cooling with a maximum at 77 K (Fig. S24 and S29, ESI†). Moreover, at deep temperatures a more pronounced resolution of enol emission was achieved.

For gaining a better understanding of the structure–property relationship, single crystals of all seven ESIPT-luminophores were grown. A detailed analysis of the crystal properties is given in the ESI,† chapter 4.8. Despite the distorted structure of oxazoline-based fluorenes on the one hand and planar arylchalcogenazole-based fluorenes on the other hand (Fig. 3), no significant difference in emission efficiency is observed. **Oxa-OH**, **BO-OH** and **BS-OH** show similar quantum yields (Table 1), thus demonstrating the efficiency of oxazoline as potent proton acceptor in ESIPT-based fluorophores and an attractive alternative for widely used benzoxazoles and -thiazoles. The combination of a less-organized crystal lattice and good quantum yield in one system is extremely rare, since potent emitters often show extremely ordered crystal packing.^11,19^ Therefore, **Oxa-OH** represents an extraordinary case in which good quantum yields are achieved, although the molecular packing is highly unorganized.

**Fig. 3:**
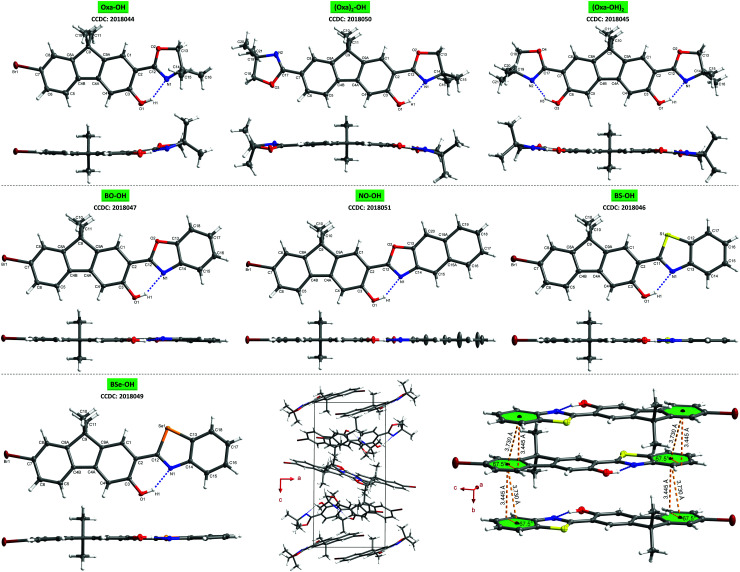
Molecular structures showing 50% probability ellipsoids, including top view and side view of the single structure. Upper row: **Oxa-OH** (left, molecule 1 of 4 of the asymmetric unit is shown), **(Oxa)2-OH** (center), **(Oxa-OH)2** (right); central row: **BO-OH** (left), **NO-OH** (center), **BS-OH** (right); lower row: **BSe-OH** (left), unit cell packing view of **Oxa-OH** along the *b*-axis demonstrating the unorganized molecular packing in the crystal lattice (center), intermolecular π–π-interactions of **BS-OH** demonstrating the well-organized crystal packing (right).

In conclusion, ESIPT-based fluorenes varying in their proton acceptor groups have been synthesized and studied towards their photophysical properties to understand their structure–property relationship. Single crystal analysis revealed well-organized crystal lattices and planar molecular structures for arylchalcogenazolylfluorenes, whereas oxazolinyl-substituted fluorenes exhibit less-organized molecular packing and a distorted π-framework. Depending on the proton acceptor group, emission colors can be varied from blue-cyan to yellow in the crystal state. Quantum yields of up to almost 40% for **Oxa-OH**, **BO-OH** and **BS-OH** in the solid-state, demonstrates the efficiency of oxazoline as proton acceptor in ESIPT-based luminophores and as an alternative choice for arylchalcogenazoles. Furthermore, with **Oxa-OH** we present a potent emitter, whose molecular packing in the crystal lattice is highly unorganized compared to known strong luminophores.

N. C. B. and P. R. thank the DFG for partial funding under Germany's Excellence Strategy within the Cluster of Excellence PhoenixD (EXC 2122, Project ID 390833453) and the European Research Council (ERC) under the European Union's Horizon 2020 research and innovation programme (grant agreement no. 714429).

## Conflicts of interest

There are no conflicts to declare.

## Supplementary Material

CC-056-D0CC05780K-s001

CC-056-D0CC05780K-s002
