# Supplementary material for: Emission color-tunable oxazol(in)yl-substituted excited-state intramolecular proton transfer (ESIPT)-based luminophores
Source: Chem Commun (Camb). 2020 Nov 10;56(98):15430–3. doi: 10.1039/d0cc05780k (PMC8517962; doi:10.1039/d0cc05780k)

Electronic Supplementary Material (ESI) for Chemical Communications.
This journal is © The Royal Society of Chemistry 2020

Emission-color-tunable oxazol(in)yl-substituted excited-state intramolecular proton transfer (ESIPT)-based luminophores

Supporting Information

Dominik Göbel,‡[a] Pascal Rusch,‡[b,c] Daniel Duvinage,[d] Nadja C. Bigall*[,b,c] and Boris J. Nachtsheim*[,a]

[a] Institute for Organic and Analytical Chemistry, University of Bremen,
Leobener Str.7, 28359 Bremen, Germany

[b] Leibniz University Hannover, Institute of Physical Chemistry and Electrochemistry,
Callinstraße 3a, D-30167 Hannover, Germany;

[c] Cluster of Excellence PhoenixD (Photonics, Optics, and Engineering – Innovation Across Disciplines),
Hannover, Germany

[d] Institute for Inorganic and Crystallographic Chemistry, University of Bremen,
Leobener Str.7, 28359 Bremen, Germany

‡ These authors contributed equally to this work.

*Corresponding authors: nachtsheim@uni-bremen.de, nadja.bigall@pci.uni-hannover.de

Table of Contents

1 **General Information** S1

2 **Experimental Procedures** S3

2.1 **Preparation of Metalation Agents and Salt Solutions** S3

2.1.1 **Preparation of the Reagent *i*PrMgCl·LiCl** S3

2.1.2 **Preparation of the Reagent TMPMgCl·LiCl** S3

2.2 **General Procedure for *ortho*-Hydroxylations** S4

2.2.1 **General Procedure for the *ortho*-Hydroxylation of Fluorenes using O_2 (GP 1)** S4

2.2.2 **General Procedure for the *ortho*-Hydroxylation of Fluorenes using $Na_2S_2O_8$ and Dioxane (GP 2)** S4

2.3 **Preparation of Oxazoline bearing Bromofluorene (Oxa-OH)** S5

2.3.1 **2,7-Dibromo-9*H*-fluorene (S1)** S5

2.3.2 **2,7-Dibromo-9,9-dimethyl-9*H*-fluorene (S2)** S6

2.3.3 **7-Bromo-9,9-dimethyl-9*H*-fluorene-2-carbaldehyde (S3)** S6

2.3.4 **2-(7-Bromo-9,9-dimethyl-9*H*-fluoren-2-yl)-4,4-dimethyl-4,5-dihydrooxazole (Oxa)** S7

2.3.5 **7-Bromo-2-(4,4-dimethyl-4,5-dihydrooxazol-2-yl)-9,9-dimethyl-9*H*-fluoren-3-ol (Oxa-OH)** S8

2.4 **Preparation of Dioxazoline bearing Fluorenes ($(Oxa\text{-}OH)_2$) and ($(Oxa)_2$-OH)** S9

2.4.1 **9,9-Dimethyl-9*H*-fluorene-2,7-dicarbaldehyde (S5)** S9

2.4.2 **2,2'-(9,9-Dimethyl-9*H*-fluorene-2,7-diyl)bis(4,4-dimethyl-4,5-dihydrooxazole) ($(Oxa)_2$)** S10

2.4.3 **2,7-Bis(4,4-dimethyl-4,5-dihydrooxazol-2-yl)-9,9-dimethyl-9*H*-fluorene-3,6-diol ($(Oxa\text{-}OH)_2$) and 2,7-Bis(4,4-dimethyl-4,5-dihydrooxazol-2-yl)-9,9-dimethyl-9*H*-fluoren-3-ol ($(Oxa)_2$-OH)** S11

2.5 **Preparation of 2-(Benzo[*d*]oxazol-2-yl)-7-bromo-9,9-dimethyl-9*H*-fluoren-3-ol (BO-OH)** S12

2.5.1 **2-(7-Bromo-9,9-dimethyl-9*H*-fluoren-2-yl)benzo[*d*]oxazole (BO)** S13

2.5.2 **2-(Benzo[*d*]oxazol-2-yl)-7-bromo-9,9-dimethyl-9*H*-fluoren-3-ol (BO-OH)** S15

2.6 **Preparation of 7-Bromo-9,9-dimethyl-2-(naphtho[2,3-*d*]oxazol-2-yl)-9*H*-fluoren-3-ol (NO-OH)** S16

2.6.1 **2-(7-Bromo-9,9-dimethyl-9*H*-fluoren-2-yl)naphtho[2,3-*d*]oxazole (NO)** S16

2.6.2 **7-Bromo-9,9-dimethyl-2-(naphtho[2,3-*d*]oxazol-2-yl)-9*H*-fluoren-3-ol (NO-OH)** S17

2.7 **Preparation of 2-(Benzo[*d*]thiazol-2-yl)-7-bromo-9,9-dimethyl-9*H*-fluoren-3-ol (BS-OH)** S18

2.7.1 **2-(7-Bromo-9,9-dimethyl-9*H*-fluoren-2-yl)benzo[*d*]thiazole (BS)** S18

2.7.2 **2-(Benzo[*d*]thiazol-2-yl)-7-bromo-9,9-dimethyl-9*H*-fluoren-3-ol (BS-OH)** S19

2.8 Preparation of 2-(Benzo[*d*][1,3]selenazol-2-yl)-7-bromo-9,9-dimethyl-9*H*-fluoren-3-ol (BSe-OH) S20
2.8.1 2,2'-Diselanediyldianiline (S6) S20
2.8.2 2-(7-Bromo-9,9-dimethyl-9*H*-fluoren-2-yl)benzo[*d*][1,3]selenazole (BSe) S22
2.8.3 2-(Benzo[*d*][1,3]selenazol-2-yl)-7-bromo-9,9-dimethyl-9*H*-fluoren-3-ol (BSe-OH) S22
3 Optical Properties S24
3.1 2-(7-Bromo-9,9-dimethyl-9*H*-fluoren-2-yl)-4,4-dimethyl-4,5-dihydrooxazole (Oxa) S24
3.2 7-Bromo-2-(4,4-dimethyl-4,5-dihydrooxazol-2-yl)-9,9-dimethyl-9*H*-fluoren-3-ol (Oxa-OH) S25
3.3 2,2'-(9,9-Dimethyl-9*H*-fluorene-2,7-diyl)bis(4,4-dimethyl-4,5-dihydrooxazole) ($(Oxa)_2$) S26
3.4 2,7-Bis(4,4-dimethyl-4,5-dihydrooxazol-2-yl)-9,9-dimethyl-9*H*-fluoren-3-ol ($(Oxa)_2$-OH) S27
3.5 2,7-Bis(4,4-dimethyl-4,5-dihydrooxazol-2-yl)-9,9-dimethyl-9*H*-fluorene-3,6-diol ($(Oxa\text{-}OH)_2$) S28
3.6 2-(7-Bromo-9,9-dimethyl-9*H*-fluoren-2-yl)benzo[*d*]oxazole (BO) S29
3.7 2-(Benzo[*d*]oxazol-2-yl)-7-bromo-9,9-dimethyl-9*H*-fluoren-3-ol (BO-OH) S30
3.8 2-(7-Bromo-9,9-dimethyl-9*H*-fluoren-2-yl)naphtho[2,3-*d*]oxazole (NO) S32
3.9 7-Bromo-9,9-dimethyl-2-(naphtho[2,3-*d*]oxazol-2-yl)-9*H*-fluoren-3-ol (NO-OH) S33
3.10 2-(7-Bromo-9,9-dimethyl-9*H*-fluoren-2-yl)benzo[*d*]thiazole (BS) S34
3.11 2-(Benzo[*d*]thiazol-2-yl)-7-bromo-9,9-dimethyl-9*H*-fluoren-3-ol (BS-OH) S35
3.12 2-(7-Bromo-9,9-dimethyl-9*H*-fluoren-2-yl)benzo[*d*][1,3]selenazole (BSe) S37
3.13 2-(Benzo[*d*][1,3]selenazol-2-yl)-7-bromo-9,9-dimethyl-9*H*-fluoren-3-ol (BSe-OH) S38
3.14 Solid-State Spectra of Hydroxyfluorenes S40
3.15 Photophysical Data of non-hydroxylated Fluorenes S41
3.16 Photophysical Data of hydroxylated Fluorenes S42
3.17 Photographs of Luminophores S43
4 Crystal Structures S45
4.1 7-Bromo-2-(4,4-dimethyl-4,5-dihydrooxazol-2-yl)-9,9-dimethyl-9*H*-fluoren-3-ol (Oxa-OH) S45
4.1.1 Crystal Data for Oxa-OH S45
4.1.2 Crystal Packing Views of Oxa-OH S61
4.2 2,7-Bis(4,4-dimethyl-4,5-dihydrooxazol-2-yl)-9,9-dimethyl-9*H*-fluoren-3-ol ($(Oxa)_2$-OH) S64
4.2.1 Crystal Data for $(Oxa)_2$-OH S64
4.2.2 Crystal Packing Views of $(Oxa)_2$-OH S71

4.3 2,7-Bis(4,4-dimethyl-4,5-dihydrooxazol-2-yl)-9,9-dimethyl-9*H*-fluorene-3,6-diol ((Oxa-OH)$_2$) S75
4.3.1 Crystal Data for (Oxa-OH)$_2$ S75
4.3.2 Crystal Packing Views of (Oxa-OH)$_2$ S83
4.4 2-(Benzo[*d*]oxazol-2-yl)-7-bromo-9,9-dimethyl-9*H*-fluoren-3-ol (BO-OH) S88
4.4.1 Crystal Data for BO-OH S88
4.4.2 Crystal Packing Views of BO-OH S94
4.5 7-Bromo-9,9-dimethyl-2-(naphtho[2,3-*d*]oxazol-2-yl)-9*H*-fluoren-3-ol (NO-OH) S97
4.5.1 Crystal Data for NO-OH S97
4.5.2 Crystal Packing Views of NO-OH S104
4.6 2-(Benzo[*d*]thiazol-2-yl)-7-bromo-9,9-dimethyl-9*H*-fluoren-3-ol (BS-OH) S108
4.6.1 Crystal Data for BS-OH S108
4.6.2 Crystal Packing Views of BS-OH S113
4.7 2-(Benzo[*d*][1,3]selenazol-2-yl)-7-bromo-9,9-dimethyl-9*H*-fluoren-3-ol (BSe-OH) S117
4.7.1 Crystal Data for BSe-OH S117
4.7.2 Crystal Packing Views of BSe-OH S123
4.8 Crystal Properties of Hydroxylated Fluorenes S127
5 References S129
6 NMR Spectra S130

1 General Information

Unless otherwise noted, all reactions were carried out under a nitrogen atmosphere using standard *Schlenk* techniques. All chemicals were purchased from commercial suppliers and either used as received or purified according to *Purification of Common Laboratory Chemicals*.[1] Anhydrous tetrahydrofuran (THF) was obtained from an *inert* PS-MD-6 solvent purification system. All other solvents were dried using standard methods.[1] Yields refer to isolated yields of compounds estimated to be > 95 % pure as determined by ^{1}H-NMR spectroscopy.

Thin layer chromatography was performed on fluorescence indicator marked precoated silica gel 60 plates (*Macherey-Nagel*, ALUGRAM Xtra SIL G/UV_{254}) and visualized by UV light (254 nm/366 nm). Flash column chromatography was performed on silica gel (0.040 – 0.063 mm) with the solvents given in the procedures. Abbreviations for solvents used: CH = cyclohexane, EE = ethyl acetate. Retention factors were determined at chamber saturation at 25 °C. Developments were carried out between 3.0 – 3.5 cm.

NMR spectra were recorded on a *Bruker* Avance 360WB spectrometer at 23 °C. Chemical shifts for ^{1}H-NMR spectra are reported as δ (parts per million) relative to the residual proton signal of $CDCl_3$ at 7.26 ppm (s) or C_6D_6 at 7.16 ppm (s). Chemical shifts for ^{13}C-NMR spectra are reported as δ (parts per million) relative to the signal of $CDCl_3$ at 77.0 ppm (t) or C_6D_6 at 128.1 ppm (t). Chemical shifts for ^{77}Se-NMR spectra are reported as δ (parts per million) relative to the signal of $Se(CH_3)_2$ at 0.0 ppm. The following abbreviations are used to describe splitting patterns: br. = broad, s = singlet, d = doublet, dd = doublet of doublets, ddd = doublet of doublet of doublets, dt = doublet of triplets, t = triplet, td = triplet of doublets, m = multiplet. Coupling constants *J* are given in Hertz.

ESI and APCI mass spectra were recorded on an *Advion* Expression CMS^{L} *via* ASAP probe or direct inlet. High resolution (HR) EI mass spectra were recorded on a double focusing mass spectrometer ThermoQuest MAT 95 XL from *Finnigan MAT*. HR-ESI and HR-APCI mass spectra were recorded on a *Bruker* Impact II. All Signals are reported with the quotient from mass to charge m/z.

IR spectra were recorded on a *Nicolet* Thermo iS10 scientific spectrometer with a diamond ATR unit. The absorption bands are reported in cm^{-1} with indicated relative intensities: s (strong, 0 – 33 % T); m (medium, 34 – 66 % T), w (weak, 67 – 100 % T), and br (broad).

Melting points of solids, compounds that solidified after chromatography, were measured on a *Büchi* M-5600 Melting Point apparatus and are uncorrected. The measurements were performed with a heating rate of 5 °C/min and the melting points are reported in °C.

Low temperature reactions (–78 °C) were cooled using a *Julabo* FT902 cryostat. If not otherwise noted, solvents were removed on a *Büchi* Rotavapor R-300 with 40 °C water bath temperature.

Extinction spectra were recorded using an *Agilent* Cary 5000 spectrometer. Photoluminescence (PL) measurements were performed using either an *Edinburgh Instruments* FLS 1000 or a *Horiba* Fluoromax 4 spectrometer. The excitation wavelength was chosen to be at the respective absorption maximum of the sample. Time-resolved PL measurements were performed using an *Edinburgh Instruments* FLS 1000 with a pulsed 376 nm laser (*Edinburgh Instruments* EPL 375.5 MHz repetition rate, 60 ps pulse width) as excitation source or on a *Horiba* FluoroHub coupled with a Fluoromax 4 spectrometer using a pulsed 250 nm LED (*Horiba* NanoLED, 5 MHz repetition rate, 1.6 ns pulse width). The resulting PL decay curves were fitted by a mono- or bi-exponential decay using the equations:

$f(t) = A_1 e^{-\frac{t}{\tau_1}} + B$ and $f(t) = A_1 e^{-\frac{t}{\tau_1}} + A_2 e^{-\frac{t}{\tau_2}} + B$, respectively.

PL quantum yield was determined using an *Edinburgh Instruments* FLS 1000 with an integrating sphere via the intensity of the scattered excitation and the sample emission measured for the empty sphere and the sample. Temperature-dependent PL spectra were measured using an *Oxford Instruments* OptistatCF cryostat coupled with the FLS 1000. Solutions were measured in quartz cuvettes from *Hellma Analytics* with 10 mm path length, thin films of the samples were produced and measured by drop-casting the respective solutions in dichloromethane onto quartz glass slides, solid samples were measured in demountable cuvettes from *Hellma Analytics* and Teflon sample holders from *Edinburgh Instruments*. Color coordinates were generated within the *Edinburgh Fluoracle* software.

Single crystals were grown as described in the procedures below. Intensity data of suitable crystals were collected on a *Bruker* Venture D8 diffractometer at 100 K with Mo-Kα (0.71073 Å) radiation. All structures were solved by direct methods and refined based on F^2 by use of the SHELX program package as implemented in Olex2.[2] All non-hydrogen atoms were refined using anisotropic displacement parameters. Hydrogen atoms attached to carbon atoms were included in geometrically calculated positions using a rigid model. Crystal and refinement data are collected in Tables S4-48. Figures were created using *Crystal Impact's* DIAMOND Version 4.6.3. Crystallographic data for the structural analyses have been deposited with the Cambridge Crystallographic Data Centre. Copies of this information may be obtained free of charge from The Director, CCDC, 12 Union Road, Cambridge CB2 1EZ, UK (Fax: +44-1223-336033; e-mail: deposit@ccdc.cam.ac.uk or http://www.ccdc.cam.ac.uk)

2 Experimental Procedures

2.1 Preparation of Metalation Agents and Salt Solutions

2.1.1 Preparation of the Reagent *i*PrMgCl·LiCl

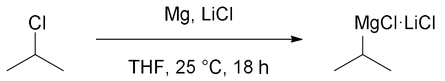

A slightly modified literature procedure was used.[3] LiCl (4.24 g, 100 mmol, 1.00 eq) was placed in a heat gun-dried and nitrogen-flushed *Schlenk* flask and heated *in vacuo* at 140 °C by heat gun for five hours. Magnesium turnings (2.67 g, 110 mmol, 1.10 eq) were placed in another heat gun-dried and nitrogen-flushed *Schlenk* flask and the dried LiCl and anhydrous THF (50 mL) were added. 2-Chloropropane (9.14 mL, 100 mmol, 1.00 eq) in anhydrous THF (50 mL) was slowly added at 25 °C through a dropping funnel. After approximately 1/5 of addition the mixture was slightly warmed with a heat gun until the reaction started (within ten minutes). When the reaction started the remaining solution was added dropwise and stirring was continued for 18 hours. After complete addition the temperature of the mixture rose until it started to boil. To remove excess of magnesium the grey solution was cannulated to another heat gun-dried and nitrogen-flushed *Schlenk* flask. The Grignard reagent was titrated[4] prior to use against I_2 (0.50 – 0.60 mmol) in anhydrous THF (2 mL) at 0 °C which resulted in a conversion of 92 – 96 %. Color change from dark violet to pale brown indicated the end of the titration.

2.1.2 Preparation of the Reagent TMPMgCl·LiCl

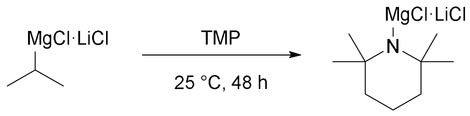

A slightly modified literature procedure was used.[5] A heat gun-dried and nitrogen-flushed *Schlenk* flask was charged with freshly titrated *i*PrMgCl·LiCl (75.0 mL, 90.0 mmol, 1.00 eq, 1.20 M). Freshly distilled TMP (16.0 mL, 94.5 mmol, 1.05 eq) was added through a rubber septum to the vigorously stirred *Grignard* solution *via* syringe pump (0.5 mL/min) at 25 °C. The reaction mixture was stirred at 25 °C for 48 hours, while the solution turned dark green. The base was titrated[6] prior to use against benzoic acid (122 mg, 1.00 mmol) using (4-phenylazo)diphenylamine (3 mg) as indicator in anhydrous THF (2.00 mL) at 0 °C which resulted in a conversion of 96 – 99 %. Color change from orange to dark violet indicated the end of the titration.

2.2 General Procedure for *ortho*-Hydroxylations

2.2.1 General Procedure for the *ortho*-Hydroxylation of Fluorenes using O_2 (GP 1)

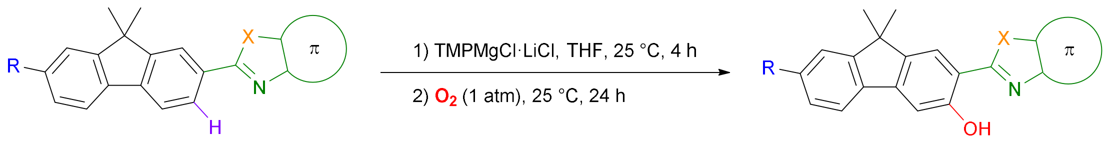

A literature method was used.[7] A heat gun-dried and nitrogen-flushed *Schlenk* tube, equipped with a magnetic stirring bar and a rubber septum, was charged with fluorene (1.00 eq) and anhydrous THF (0.2 – 0.4 M) was added. Dropwise addition of TMPMgCl·LiCl (3.00 eq) *via* syringe through the rubber septum, if not otherwise noted at 25 °C, was followed by stirring for the indicated time under the same conditions, while the mixture discolored pale red to dark red/brown, depending on the substrate. Then the nitrogen atmosphere was replaced by an oxygen atmosphere by flushing the reaction system using an oxygen filled balloon and the mixture was stirred for an additional 24 hours, if not otherwise noted at 25 °C. The clear, pale red to yellow, mixture was subjected to aqueous workup and the organic layer was dried over anhydrous Na_2SO_4, filtered and concentrated under reduced pressure on a rotary evaporator. Purification of the crude product was conducted by flash column chromatography using the given eluent.

2.2.2 General Procedure for the *ortho*-Hydroxylation of Fluorenes using $Na_2S_2O_8$ and Dioxane (GP 2)

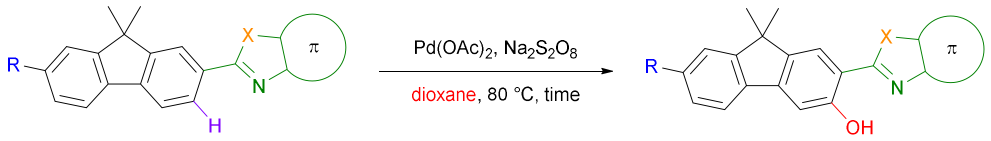

A literature method was used.[8] A heat gun-dried and nitrogen-flushed *Schlenk* tube, equipped with a magnetic stirring bar and a rubber septum, was charged with modified fluorene (1.00 eq) and anhydrous 1,4-dioxane (0.1 M) was added. $Pd(OAc)_2$ (3 mol%) and $Na_2S_2O_8$ (1.25 eq) were added successively and the mixture was stirred at 80 °C (oil bath temperature) for the indicated time. The turbid, brown mixture was subjected to aqueous workup and the organic layer was dried over anhydrous Na_2SO_4, filtered and concentrated under reduced pressure on a rotary evaporator. Purification of the crude product was conducted by flash column chromatography using the given eluent.

2.3 Preparation of Oxazoline bearing Bromofluorene (Oxa-OH)

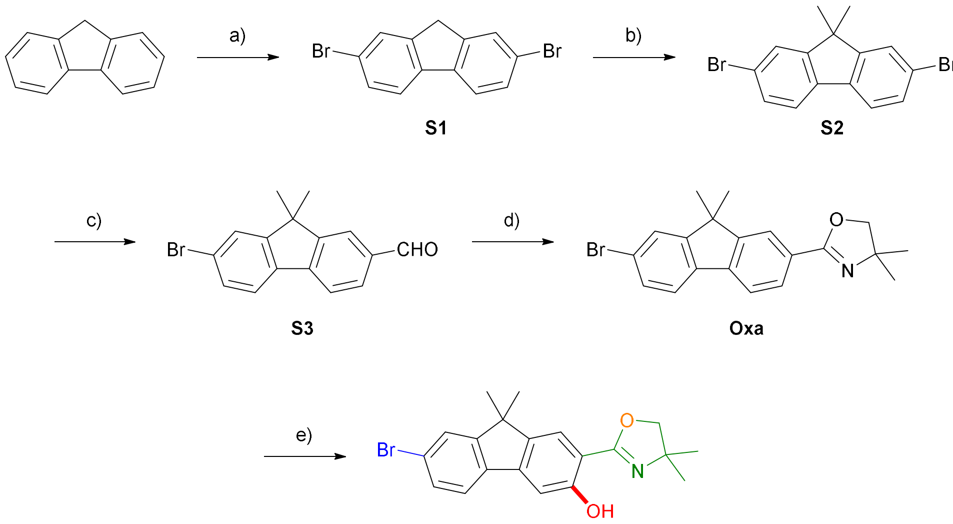

Reaction conditions: a) Br_2, Fe-powder, $CHCl_3$, 0 °C, 4 h, 99 %; b) KOH, KI, MeI, DMSO, 25 °C, 18 h, 92 %; c) 1) *n*BuLi, THF, –78 °C, 1 h; 2) DMF, THF, –78 °C to 25 °C, 10 h, 90 %; d) 1) 2-amino-2-methylpropan-1-ol, 4 Å MS, CH_2Cl_2, 25 °C, 18 h; 2) NBS, CH_2Cl_2, 25 °C, 3 h, 92 %; e) 1) TMPMgCl·LiCl, THF, 25 °C, 4 h; 2) O_2, 25 °C, 24 h, 86 %.

2.3.1 2,7-Dibromo-9*H*-fluorene (S1)

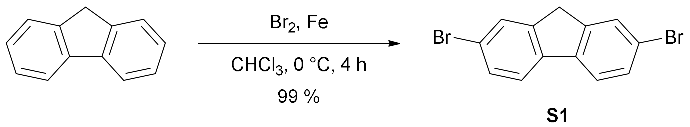

A modified literature procedure was used.[9] Fluorene (8.31 g, 50.0 mmol, 1.00 eq) was dissolved in $CHCl_3$ (83 mL, 0.6 M) and Fe-powder (279 mg, 5.00 mmol, 0.10 eq) was added. The solution was cooled to 0 °C in a water/ice bath. Br_2 (5.38 mL, 105 mmol, 2.10 eq) in $CHCl_3$ (42 mL) was added through a dropping funnel over one hour in the dark to the vigorously stirred mixture. After complete addition the mixture was stirred for an additional three hours at 0 °C. Saturated $Na_2S_2O_5$ solution (100 mL) was slowly added at the same temperature and stirring was continued for 30 minutes. $CHCl_3$ (100 mL) was added, the phases were separated and the aqueous layer was extracted with $CHCl_3$ (2 × 100 mL). The combined organic layers were dried over anhydrous Na_2SO_4, filtered and concentrated under reduced pressure on a rotary evaporator. **S1** (16.1 g, 49.7 mmol, 99 %) was isolated as a colorless solid. If desired the product can be recrystallized from chloroform.

R_f = 0.61 (SiO_2, CH). **Mp.:** 164 – 166 °C. **^{1}H-NMR (360 MHz, $CDCl_3$):** δ = 7.64 (d, $^4J_{HH}$ = 1.6 Hz, 2H), 7.57 (d, $^3J_{HH}$ = 8.1 Hz, 2H), 7.49 (dd, $^3J_{HH}$ = 8.2 Hz, $^4J_{HH}$ = 1.8 Hz, 2H), 3.83 (s, 2H) ppm. **^{13}C{^{1}H}-NMR (91 MHz, $CDCl_3$):** δ = 144.9 (2x), 139.8 (2x), 130.2 (2x), 128.4 (2x), 121.3 (2x), 121.1 (2x), 36.7

ppm. **IR (ATR, neat):** $\tilde{\nu}$ = 3049 (w), 2919 (w), 1881 (w), 1760 (w), 1568 (w), 1453 (w), 1391 (m), 1159 (w), 1054 (m), 1005 (w), 952 (w), 931 (w), 807 (s), 685 (m), 662 (m) cm^{-1}. **MS (EI, 70 eV):** m/z = 321.8 $[C_{13}H_8{}^{79}Br_2]^{+\cdot}$. The analytical data are in accordance with the literature.[10,11]

2.3.2 2,7-Dibromo-9,9-dimethyl-9*H*-fluorene (S2)

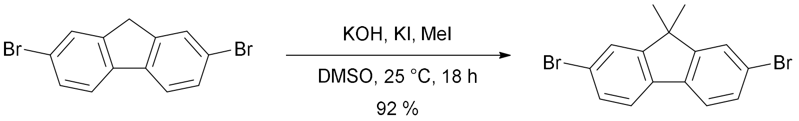

S1 **S2**

A modified literature procedure was used.[12] **S1** (16.2 g, 50.0 mmol, 1.00 eq) was suspended in DMSO (83 mL, 0.6 M) and KI (830 mg, 5.00 mmol, 0.10 eq) was added. To the water bath-cooled and vigorously stirred mixture were added KOH pellets (11.2 g, 200 mmol, 4.00 eq). The reaction was stirred for one hour, while the solution turned intensive red. MeI (7.78 mL, 125 mmol, 2.50 eq) was added *via* syringe pump (0.15 mL/min) through a rubber septum and stirring was continued at 25 °C for 18 hours. Excess of MeI was quenched by addition of NEt_3 (13.9 mL, 100 mmol, 2.00 eq). The mixture was stirred for 30 minutes, poured into water (500 mL) and extracted with CH_2Cl_2 (4 × 100 mL). The combined organic layers were dried over anhydrous $NaSO_4$, filtered and concentrated under reduced pressure on a rotary evaporator. Purification by flash column chromatography (SiO_2, CH) afforded **S2** (16.2 g, 46.0 mmol, 92 %) as a colorless solid. If desired the product can be recrystallized from cyclohexane.

$\mathbf{R_f}$ = 0.68 (SiO_2, CH). **Mp.:** 177 – 179 °C. **^{1}H-NMR (360 MHz, CDCl$_3$):** δ = 7.55 (d, $^4J_{HH}$ = 1.8 Hz, 2H), 7.54 (d, $^3J_{HH}$ = 8.1 Hz, 2H), 7.46 (dd, $^3J_{HH}$ = 8.1 Hz, $^4J_{HH}$ = 1.8 Hz, 2H), 1.47 (s, 6H) ppm. **^{13}C{^{1}H}-NMR (91 MHz, CDCl$_3$):** δ = 155.4 (2x), 137.3 (2x), 130.5 (2x), 126.3 (2x), 121.6 (4x), 47.4, 27.0 (2x) ppm. **IR (ATR, neat):** $\tilde{\nu}$ = 2962 (m), 2920 (w), 2856 (w), 1861 (w), 1727 (w), 1597 (w), 1577 (w), 1447 (m), 1397 (m), 1259 (m), 1083 (m), 1058 (m), 1001 (m), 865 (m), 824 (m), 791 (s), 729 (m), 667 (m) cm^{-1}. **MS (EI, 70 eV):** m/z = 349.9 $[C_{15}H_{12}{}^{79}Br_2]^{+\cdot}$. The analytical data are in accordance with the literature.[11,13]

2.3.3 7-Bromo-9,9-dimethyl-9*H*-fluorene-2-carbaldehyde (S3)

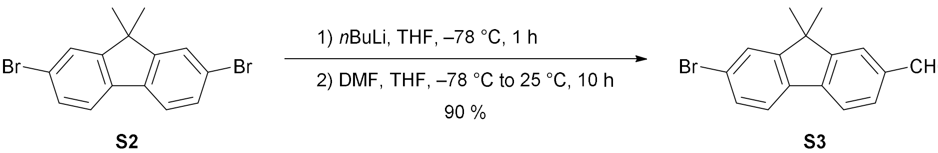

S2 **S3**

A modified literature procedure was used.[12] A heat gun-dried and nitrogen-flushed *Schlenk* flask was charged with **S2** (14.1 g, 40.0 mmol, 1.00 eq) and anhydrous THF (200 mL, 0.2 M) was added. The solution was cooled to –78 °C and *n*BuLi (16.8 mL, 42.0 mmol, 1.05 eq, 2.5 M in hexane) was added

via syringe pump (0.2 mL/min) through a rubber septum to the vigorously stirred mixture. The intensive red solution was stirred for one hour at –78 °C and anhydrous DMF (5.85 g, 6.19 mL, 80.0 mmol, 2.00 eq) in anhydrous THF (8 mL) was added *via* syringe pump (0.4 mL/min) through a rubber septum. Stirring was continued for 10 hours while the mixture was allowed to warm slowly to 25 °C. HCl (100 mL, 1.0 M) was poured into the reaction in small portions and the mixture was stirred rapidly for 30 minutes. The phases were separated and the aqueous layer was extracted with ethyl acetate (3 × 100 mL). The combined organic layers were dried over anhydrous Na_2SO_4, filtered and concentrated under reduced pressure on a rotary evaporator. Purification by flash column chromatography (SiO_2, CH:EE 20:1 v:v) afforded **S3** (10.9 g, 36.0 mmol, 90 %) as a colorless solid.

R_f = 0.26 (SiO_2, CH:EE 20:1 v:v). **Mp.:** 147 – 149 °C. **^{1}H-NMR (360 MHz, $CDCl_3$):** δ = 10.06 (s, 1H), 7.96 (d, $^4J_{HH}$ = 1.5 Hz, 1H), 7.87 (dd, $^3J_{HH}$ = 7.8 Hz, $^4J_{HH}$ = 1.3 Hz, 1H), 7.82 (d, $^3J_{HH}$ = 7.8 Hz, 1H), 7.65 (d, $^3J_{HH}$ = 8.1 Hz, 1H), 7.61 (d, $^4J_{HH}$ = 1.6 Hz, 1H), 7.51 (dd, $^3J_{HH}$ = 8.1 Hz, $^4J_{HH}$ = 1.7 Hz, 1H), 1.52 (s, 6H) ppm. **^{13}C{^{1}H}-NMR (91 MHz, $CDCl_3$):** δ = 192.2, 157.0, 154.1, 144.6, 136.8, 135.9, 130.8, 130.7, 126.6, 123.2, 122.7, 120.5, 47.4, 26.9 (2x) ppm. **IR (ATR, neat):** $\tilde{\nu}$ = 2963 (w), 2924 (w), 2814 (w), 2783 (w), 2708 (w), 1695 (s), 1683 (s), 1605 (m), 1405 (m), 1247 (m), 1175 (s), 1060 (m), 883 (m), 810 (s), 795 (s), 755 (s), 733 (s), 658 (m) cm^{-1}. **MS (APCI):** m/z = 301.1 [$C_{16}H_{13}{}^{79}BrO+H$]$^+$. The analytical data are in accordance with the literature.[11,14]

2.3.4 2-(7-Bromo-9,9-dimethyl-9*H*-fluoren-2-yl)-4,4-dimethyl-4,5-dihydrooxazole (Oxa)

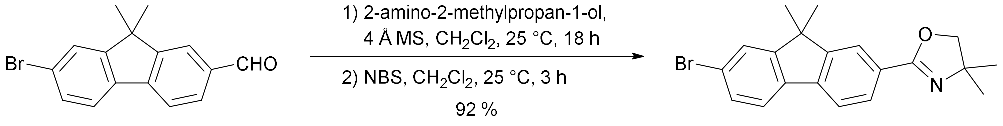

S3 **Oxa**

A modified literature procedure was used.[15] **S3** (3.01 g, 10.0 mmol, 1.00 eq) and 2-amino-2-methylpropan-1-ol (1.78 g, 1.91 mL, 20.0 mmol, 2.00 eq) were dissolved in CH_2Cl_2 (40 mL, 0.25 M) and 4 Å MS (5.00 g) was added. The mixture was slowly stirred for 18 hours at 25 °C whereupon NBS (3.56 g, 20.0 mmol, 2.00 eq) was added in one portion and stirring was continued for another three hours at the same temperature. All solids were filtered off and the organic phase was washed with saturated $NaHCO_3$ solution (2 × 50 mL). The combined aqueous phases were extracted with CH_2Cl_2 (2 × 50 mL). All organic phases were combined and washed with saturated $Na_2S_2O_3$ solution (50 mL) and the aqueous phase was extracted with CH_2Cl_2 (20 mL). The combined organic phases were dried over anhydrous $MgSO_4$, filtered and concentrated under reduced pressure on a rotary evaporator. Purification by flash column chromatography (SiO_2, CH:EE 15:1 v:v) afforded **Oxa** (3.41 g, 9.21 mmol, 92 %) as a colorless foam.

R_f = 0.17 (SiO_2, CH:EE 15:1 v:v). **Mp.:** 62 – 64 °C. **^{1}H-NMR (360 MHz, $CDCl_3$):** δ = 8.02 (d, $^4J_{HH}$ = 1.4 Hz, 1H), 7.92 (dd, $^3J_{HH}$ = 7.9 Hz, $^4J_{HH}$ = 1.5 Hz, 1H), 7.69 (d, $^3J_{HH}$ = 7.9 Hz, 1H), 7.58 (d, $^3J_{HH}$ = 8.0 Hz, 1H), 7.56 (d, $^4J_{HH}$ = 1.8 Hz, 1H), 7.46 (dd, $^3J_{HH}$ = 8.0 Hz, $^4J_{HH}$ = 1.9 Hz, 1H), 4.12 (s, 2H), 1.48 (s, 6H), 1.41 (s, 6H) ppm. **^{13}C{^{1}H}-NMR (91 MHz, $CDCl_3$):** δ = 162.3, 156.4, 153.3, 141.2, 137.4, 130.4, 127.8, 127.2, 126.4, 122.7, 122.1, 122.0, 119.9, 79.2, 67.7, 47.4, 28.6 (2x), 26.9 (2x) ppm. **IR (ATR, neat):** $\tilde{\nu}$ = 2962 (w), 2924 (w), 2359 (w), 1641 (s), 1452 (m), 1403 (m), 1355 (m), 1308 (s), 1262 (s), 1202 (s), 1086 (m), 1060 (s), 968 (m), 815 (s), 774 (m), 738 (s), 716 (s) cm^{-1}. **MS (APCI):** m/z = 370.2 [$C_{20}H_{20}{}^{79}BrNO+H$]$^+$. **HR-MS (EI, 70 eV):** calculated for $C_{20}H_{20}{}^{79}BrNO^{+\cdot}$ [M]$^{+\cdot}$: m/z = 369.07228, found: 369.07227 (Dev.: –0.01 mu; –0.02 ppm). The analytical data are in accordance with the literature.[11]

2.3.5 7-Bromo-2-(4,4-dimethyl-4,5-dihydrooxazol-2-yl)-9,9-dimethyl-9*H*-fluoren-3-ol (Oxa-OH)

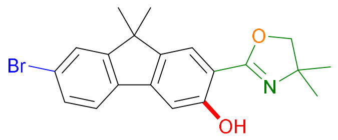

Oxa-OH

Prepared according to **GP 1** from **Oxa** (370 mg, 1.00 mmol, 1.00 eq) in THF (2.50 mL, 0.4 M) and TMPMgCl·LiCl (2.50 mL, 3.00 mmol, 3.00 eq, 1.20 M in THF). Stirring for four hours was followed by flushing with oxygen and stirring for another 24 hours. Saturated NH_4Cl solution (10 mL), water (10 mL) and CH_2Cl_2 (20 mL) were added and the phases were separated. The aqueous phase was extracted with CH_2Cl_2 (4 × 20 mL). Purification by flash column chromatography (SiO_2, CH:EE 20:1 v:v) afforded **Oxa-OH** (332 mg, 859 µmol, 86 %) as a colorless solid. Single crystals suitable for X-ray analysis were grown from MeCN solution by slow solvent evaporation.

R_f = 0.48 (SiO_2, CH:EE 20:1 v:v). **Mp.:** 150 – 152 °C. **^{1}H-NMR (360 MHz, $CDCl_3$):** δ = 12.37 (br. s, 1H), 7.66 (s, 1H), 7.57 (d, $^3J_{HH}$ = 8.0 Hz, 1H), 7.55 (d, $^4J_{HH}$ = 1.8 Hz, 1H), 7.47 (dd, $^3J_{HH}$ = 8.1 Hz, $^4J_{HH}$ = 1.8 Hz, 1H), 7.31 (s, 1H), 4.13 (s, 2H), 1.46 (s, 6H), 1.42 (s, 6H) ppm. **^{13}C{^{1}H}-NMR (91 MHz, $CDCl_3$):** δ = 163.8, 160.0, 157.1, 143.4, 143.3, 137.5, 130.4, 126.4, 122.4, 122.4, 121.7, 109.9, 107.9, 78.5, 67.3, 46.7, 28.7 (2x), 27.4 (2x) ppm. **IR (ATR, neat):** $\tilde{\nu}$ = 2959 (w), 2922 (w), 2893 (w), 2862 (w), 1734 (w), 1635 (s), 1584 (m), 1454 (m), 1389 (m), 1375 (m), 1310 (m), 1269 (s), 1218 (m), 1200 (s), 1129 (w), 1096 (m), 1063 (m), 1052 (s), 975 (m), 949 (m), 916 (w), 896 (m), 866 (m), 827 (m), 819 (s), 804 (s), 757 (m), 726 (m), 656 (s) cm^{-1}. **MS (APCI):** m/z = 386.0 [$C_{20}H_{20}{}^{79}BrNO_2+H$]$^+$. **HR-MS (APCI):** calculated for $C_{20}H_{21}{}^{79}BrNO_2{}^+$ [M+H]$^+$: m/z = 386.07502, found: 386.07565 (Dev.: 0.63 mu; 1.63 ppm). The analytical data are in accordance with the literature.[11]

2.4 Preparation of Dioxazoline bearing Fluorenes ($(Oxa\text{-}OH)_2$) and ($(Oxa)_2\text{-}OH$)

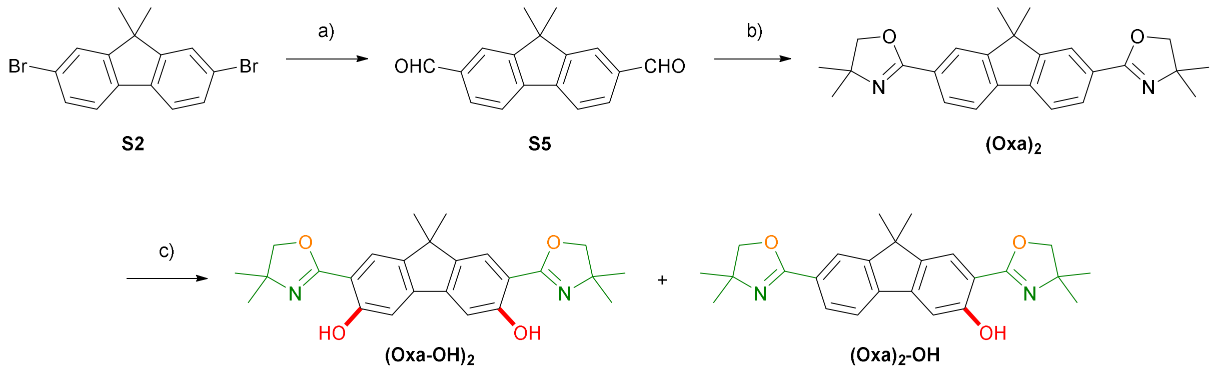

Reaction conditions: a) 1) *n*BuLi, THF, –78 °C, 1 h; 2) DMF, THF, –78 °C to 25 °C, 10 h, 82 %; f) 1) 2-amino-2-methylpropan-1-ol, 4 Å MS, CH_2Cl_2, 25 °C, 18 h; 2) NBS, CH_2Cl_2, 25 °C, 3 h, 97 %; c) 1) TMPMgCl·LiCl, THF, 25 °C, 4 h; 2) O_2, 25 °C, 24 h, 9 – 85 %.

2.4.1 9,9-Dimethyl-9*H*-fluorene-2,7-dicarbaldehyde (S5)

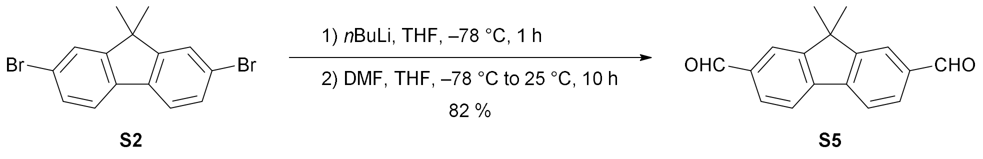

A modified literature procedure was used.[12] A heat gun-dried and nitrogen-flushed *Schlenk* flask was charged with **S2** (7.04 g, 20.0 mmol, 1.00 eq) and anhydrous THF (200 mL, 0.1 M) was added. The solution was cooled to –78 °C and *n*BuLi (17.6 mL, 44.0 mmol, 2.20 eq, 2.5 M in hexane) was added *via* syringe pump (0.6 mL/min) through a rubber septum to the vigorously stirred mixture. The intensive red solution was stirred for one hour at –78 °C, while the dilithiated species precipitated from the mixture. Anhydrous DMF (4.39 g, 4.65 mL, 60.0 mmol, 3.00 eq) in anhydrous THF (5.0 mL) was added *via* syringe pump (0.6 mL/min) through a rubber septum. Stirring was continued for 10 hours while the mixture was allowed to warm slowly to 25 °C. HCl (100 mL, 0.5 M) was poured in small portions to the reaction and the mixture was stirred rapidly for 30 minutes. The phases were separated and the aqueous layer was extracted with ethyl acetate (3 × 100 mL). The combined organic layers were dried over anhydrous Na_2SO_4, filtered and concentrated under reduced pressure on a rotary evaporator. Purification by flash column chromatography (SiO_2, CH:EE 6:1 to 4:1 v:v) afforded **S5** (4.08 g, 16.3 mmol, 82 %) as a pale yellow solid.

R_f = 0.21 (SiO_2, CH:EE 6:1 v:v). **R_f** = 0.34 (SiO_2, CH:EE 4:1 v:v). **Mp.:** 133 – 135 °C. **^{1}H-NMR (360 MHz, $CDCl_3$):** δ = 10.09 (s, 2H), 8.01 (dd, $^4J_{HH}$ = 1.4 Hz, $^5J_{HH}$ = 0.8 Hz, 2H), 7.95 (dd, $^3J_{HH}$ = 7.8 Hz, $^5J_{HH}$ = 0.8 Hz, 2H), 7.91 (dd, $^3J_{HH}$ = 7.8 Hz, $^4J_{HH}$ = 1.4 Hz, 2H), 1.57 (s, 6H) ppm. **^{13}C{^{1}H}-NMR**

(91 MHz, $CDCl_3$): *δ* = 192.1 (2x), 155.7 (2x), 143.9 (2x), 136.7 (2x), 130.5 (2x), 123.5 (2x), 121.7 (2x), 47.3, 26.8 (2x) ppm. **IR (ATR, neat):** $\tilde{\nu}$ = 3046 (w), 2952 (w), 2857 (w), 2757 (w), 1684 (s), 1602 (m), 1473 (w), 1461 (m), 1436 (m), 1420 (m), 1402 (m), 1362 (w), 1346 (w), 1316 (m), 1293 (w), 1271 (w), 1259 (w), 1230 (w), 1206 (m), 1172 (s), 1130 (m), 1077 (w), 1004 (m), 952 (w), 931 (w), 920 (m), 845 (m), 822 (s), 742 (s), 661 (m) cm^{-1}. **MS (APCI):** m/z = 251.2 $[C_{17}H_{14}O_2+H]^+$. **HR-MS (ESI):** calculated for $C_{17}H_{15}O_2^+$ $[M+H]^+$: m/z = 251.10666, found: 251.10682 (Dev.: 0.17 mu; 0.67 ppm); calculated for $C_{17}H_{14}NaO_2^+$ $[M+Na]^+$: m/z = 273.08860, found: 273.08885 (Dev.: 0.25 mu; 0.90 ppm). The analytical data are in accordance with the literature.[16]

2.4.2 2,2'-(9,9-Dimethyl-9*H*-fluorene-2,7-diyl)bis(4,4-dimethyl-4,5-dihydrooxazole) ((Oxa)₂)

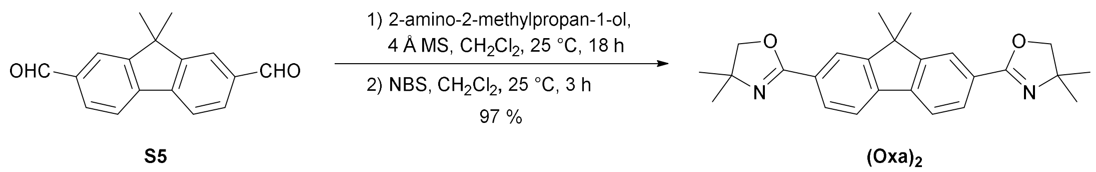

A modified literature procedure was used.[15] **S5** (501 mg, 2.00 mmol, 1.00 eq) and 2-amino-2-methylpropan-1-ol (713 mg, 763 µL, 8.00 mmol, 4.00 eq) were dissolved in CH_2Cl_2 (6.7 mL, 0.3 M) and 4 Å MS (2.00 g) was added. The mixture was slowly stirred for 18 hours at 25 °C whereupon NBS (1.42 g, 8.00 mmol, 4.00 eq) was added in one portion and stirring was continued for another three hours at the same temperature. All solids were filtered off and the organic phase was washed with saturated $NaHCO_3$ solution (3 × 20 mL). The combined aqueous phases were extracted with CH_2Cl_2 (2 × 20 mL). All organic phases were combined and washed with saturated $Na_2S_2O_3$ solution (20 mL) and the aqueous phase was extracted with CH_2Cl_2 (20 mL). The combined organic phases were dried over anhydrous $MgSO_4$, filtered and concentrated under reduced pressure on a rotary evaporator. Purification by flash column chromatography (SiO_2, CH:EE 1:1 v:v) afforded **$(Oxa)_2$** (752 mg, 1.94 mmol, 97 %) as a colorless foam.

R_f = 0.46 (SiO_2, CH:EE 1:1 v:v). **Mp.:** 175 – 177 °C. **^{1}H-NMR (360 MHz, $CDCl_3$):** *δ* = 8.03 (dd, $^4J_{HH}$ = 1.5 Hz, $^5J_{HH}$ = 0.6 Hz, 2H), 7.94 (dd, $^3J_{HH}$ = 8.0 Hz, $^4J_{HH}$ = 1.5 Hz, 2H), 7.75 (dd, $^3J_{HH}$ = 7.9 Hz, $^5J_{HH}$ = 0.7 Hz, 2H), 4.12 (s, 4H), 1.51 (s, 6H), 1.40 (s, 12H) ppm. **$^{13}C\{^1H\}$-NMR (91 MHz, $CDCl_3$):** *δ* = 162.4 (2x), 154.5 (2x), 141.4 (2x), 127.7 (2x), 127.5 (2x), 122.8 (2x), 120.4 (2x), 79.2 (2x), 67.7 (2x), 47.3, 28.6 (4x), 26.9 (2x) ppm. **IR (ATR, neat):** $\tilde{\nu}$ = 2962 (w), 2926 (w), 2889 (w), 2864 (w), 1645 (s), 1615 (w), 1574 (w), 1460 (m), 1436 (w), 1421 (m), 1381 (w), 1362 (w), 1348 (m), 1305 (s), 1267 (s), 1246 (m), 1196 (m), 1135 (w), 1087 (m), 1065 (s), 1058 (s), 991 (m), 968 (s), 938 (m), 921 (m), 900 (m), 880 (m), 853 (w), 834 (s), 789 (w), 742 (s), 719 (s) cm^{-1}. **MS (APCI):** m/z = 389.3 $[C_{25}H_{28}N_2O_2+H]^+$. **HR-MS (ESI):** calculated for $C_{25}H_{29}N_2O_2^+$ $[M+H]^+$: m/z = 389.22235, found: 389.22194 (Dev.: –0.41 mu; –1.05 ppm). The analytical data are in accordance with the literature.[7]

2.4.3 2,7-Bis(4,4-dimethyl-4,5-dihydrooxazol-2-yl)-9,9-dimethyl-9*H*-fluorene-3,6-diol ((Oxa-OH)$_2$) and 2,7-Bis(4,4-dimethyl-4,5-dihydrooxazol-2-yl)-9,9-dimethyl-9*H*-fluoren-3-ol ((Oxa)$_2$-OH)

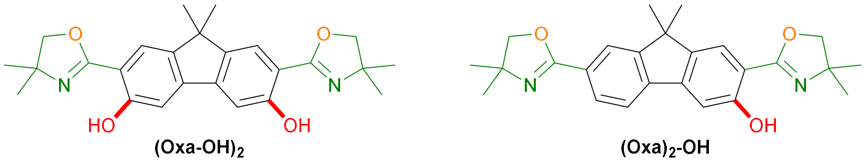

Prepared according to **GP 1** from **(Oxa)$_2$** (194 mg, 500 µmol, 1.00 eq) in THF (1.25 mL, 0.4 M) and TMPMgCl·LiCl (X mL, Y mmol, Z eq, 1.20 M in THF). Stirring for four hours was followed by flushing with oxygen and stirring for another 24 hours. Saturated NH_4Cl solution (10 mL), water (10 mL) and CH_2Cl_2 (20 mL) were added and the phases were separated. The aqueous phase was extracted with CH_2Cl_2 (4 × 20 mL). Purification by flash column chromatography (SiO_2, CH:EE 30:1 to 15:1 v:v) afforded **(Oxa-OH)$_2$** (X mg, Y µmol, Z %) as a colorless solid and **(Oxa)$_2$-OH** (X mg, Y µmol, Z %) as an off-white solid. **(Oxa-OH)$_2$**: Single crystals suitable for X-ray analysis were obtained from very slow diffusion of a cyclohexane layer into a CH_2Cl_2 solution of **(Oxa-OH)$_2$** and concomitant slow solvent evaporation. **(Oxa)$_2$-OH**: Single crystals suitable for X-ray analysis were grown from CH_2Cl_2 solution by slow solvent evaporation.

Table S1: Different conditions for the hydroxylation of 2,7-bis(oxazolinyl)-fluorene **(Oxa)$_2$**.

entry[a]	TMPMgCl·LiCl (X mL, Y mmol, Z eq)	products (X mg, Y µmol, Z %)[b] **(Oxa-OH)$_2$**	**(Oxa)$_2$-OH**
1	2.50 mL, 3.00 mmol, 6.00 eq	75.2 mg, 179 µmol, 36 %	79.5 mg, 197 µmol, 39 %
2	5.00 mL, 6.00 mmol, 12.0 eq	179 mg, 426 µmol, 85 %	18.0 mg, 44.5 µmol, 9 %

[a] Reactions were conducted with **(Oxa)$_2$** (0.5 mmol) in THF (0.4 M) and metalation agent as described. [b] Isolated yields after column chromatography. Highlighted in green are the preferred structures.

(Oxa-OH)$_2$: R$_f$ = 0.20 (SiO_2, CH:EE 30:1 v:v). **R$_f$** = 0.58 (SiO_2, CH:EE 15:1 v:v). **Mp.:** 285 – 287 °C. **^{1}H-NMR (360 MHz, $CDCl_3$):** δ = 12.33 (br. s, 2H), 7.66 (s, 2H), 7.34 (s, 2H), 4.14 (s, 4H), 1.46 (s, 6H), 1.42 (s, 12H). ppm. **^{13}C{^{1}H}-NMR (91 MHz, $CDCl_3$):** δ = 163.9 (2x), 159.8 (2x), 145.2 (2x), 143.5 (2x), 121.7 (2x), 110.4 (2x), 108.7 (2x), 78.5 (2x), 67.3 (2x), 45.9, 28.7 (4x), 27.9 (2x) ppm. **IR (ATR, neat):** $\tilde{\nu}$ = 2970 (w), 2919 (w), 2846 (w), 1634 (s), 1586 (m), 1458 (w), 1410 (s), 1381 (w), 1365 (m), 1334 (w), 1290 (m), 1274 (s), 1226 (w), 1203 (s), 1175 (s), 1099 (s), 1060 (m), 1028 (s), 965 (s), 945 (m), 922 (m), 899 (m), 865 (m), 803 (s), 780 (s), 734 (w), 705 (m), 679 (w) cm^{-1}. **MS (APCI):** m/z = 421.3 [$C_{25}H_{28}N_2O_4$+H]$^+$. **HR-MS (ESI):** calculated for $C_{25}H_{30}N_2O_4^+$ [M+2H]$^{2+}$: m/z = 211.10973, found: 211.10933 (Dev.: –0.40 mu; –1.89 ppm); calculated for $C_{25}H_{29}N_2O_2^+$ [M+H]$^+$: m/z = 421.21218, found: 421.21132 (Dev.: –0.86 mu; –2.04 ppm). The analytical data are in accordance with the literature.[7]

(Oxa)$_2$-OH: R$_f$ = 0.06 (SiO_2, CH:EE 30:1 v:v). **R$_f$** = 0.17 (SiO_2, CH:EE 15:1 v:v). **Mp.:** 235 – 237 °C. **^{1}H-NMR (360 MHz, $CDCl_3$):** δ = 12.35 (br. s, 1H), 8.05 – 8.02 (m, 1H), 7.93 (dt, $^3J_{HH}$ = 7.9 Hz, $^4J_{HH}$ = 1.5 Hz, 1H), 7.72 (dt, $^3J_{HH}$ = 8.0 Hz, $^5J_{HH}$ = 0.8 Hz, 1H), 7.68 (s, 1H), 7.35 (s, 1H), 4.14 (d, $^4J_{HH}$ = 3.2 Hz, 2H), 4.12 (d, $^4J_{HH}$ = 1.9 Hz, 2H), 1.49 (s, 6H), 1.42 – 1.40 (m, 12H) ppm. **^{1}H-NMR (360 MHz, C_6D_6):** δ = 12.73 (s, 1H), 8.40 (d, $^4J_{HH}$ = 1.5 Hz, 1H), 8.21 (dd, $^3J_{HH}$ = 7.9 Hz, $^4J_{HH}$ = 1.4 Hz, 1H), 7.88 (s, 1H), 7.55 (s, 1H), 7.40 (dd, $^3J_{HH}$ = 8.0 Hz, $^5J_{HH}$ = 0.7 Hz, 1H), 3.76 (s, 2H), 3.49 (s, 2H), 1.30 (s, 6H), 1.23 (s, 6H), 0.98 (s, 6H) ppm. **^{13}C{^{1}H}-NMR (91 MHz, $CDCl_3$):** δ = 163.8, 162.5, 159.9, 155.1, 144.5, 143.3, 141.5, 141.5, 127.7, 122.8, 121.7, 120.7, 110.3, 108.4, 79.3, 78.5, 67.7, 67.3, 46.6, 28.7 (2x), 28.5 (2x), 27.4 (2x) ppm. **^{13}C{^{1}H}-NMR (91 MHz, C_6D_6):** δ = 164.4, 162.0, 161.0, 155.4, 144.7, 144.1, 141.6, 128.9, 123.3, 122.1, 121.3, 110.6, 109.1, 79.0, 78.0, 68.0, 67.1, 46.6, 28.6 (2x), 28.2 (2x), 27.3 (2x) ppm. **IR (ATR, neat):** $\tilde{\nu}$ = 2960 (w), 2930 (w), 2889 (w), 1638 (s), 1618 (m), 1594 (m), 1466 (m), 1406 (m), 1389 (m), 1378 (w), 1365 (m), 1348 (m), 1322 (w), 1307 (m), 1276 (s), 1253 (m), 1222 (m), 1203 (s), 1090 (m), 1064 (s), 993 (m), 969 (s), 952 (m), 914 (m), 899 (m), 887 (m), 877 (m), 866 (m), 844 (s), 795 (s), 776 (s), 729 (m), 718 (s), 658 (m) cm^{-1}. **MS (APCI):** m/z = 405.3 [$C_{25}H_{28}N_2O_3$+H]$^+$. **HR-MS (ESI):** calculated for $C_{25}H_{29}N_2O_3^+$ [M+H]$^+$: m/z = 405.21727, found: 405.21693 (Dev.: –0.34 mu; –0.83 ppm). The analytical data are in accordance with the literature.[7]

2.5 Preparation of 2-(Benzo[*d*]oxazol-2-yl)-7-bromo-9,9-dimethyl-9*H*-fluoren-3-ol (BO-OH)

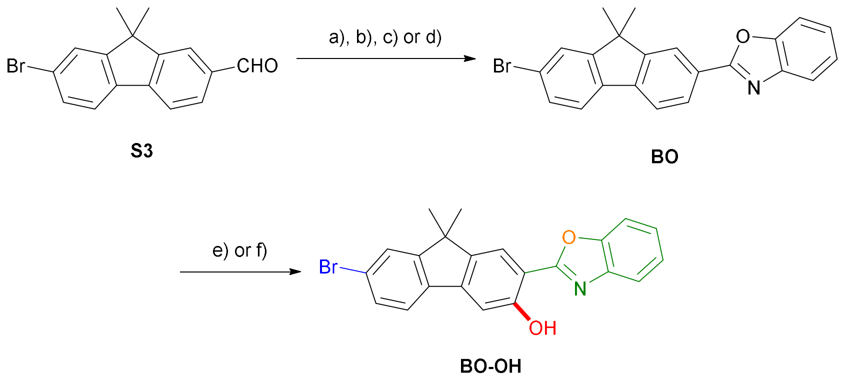

Reaction conditions: a) 1) 2-aminophenol, 4 Å MS, DMSO, 100 °C, 4 h; 2) NBS, CH_2Cl_2, 25 °C, 2 h, 24 %; b) 2-aminophenol, I_2, K_2CO_3, MeCN, 25 °C, 18 h; then 60 °C, 4 h, 28 %; c) 2-aminophenol, MnO_2 (act.), MeCN, 25 °C, 48 h; then 60 °C, 4 h, traces; d) 1) 2-aminophenol, EtOH, 90 °C, 4 h; 2) DDQ, CH_2Cl_2, 25 °C, 1 h, 93 %; e) 1) TMPMgCl·LiCl, THF, 25 °C, 4 h; 2) O_2, 25 °C, 24 h, 35 % (61 % BRSM); f) $Pd(OAc)_2$, $Na_2S_2O_8$, 1,4-dioxane, 80 °C, 16 h, 50 % (79 % BRSM).

2.5.1 2-(7-Bromo-9,9-dimethyl-9*H*-fluoren-2-yl)benzo[*d*]oxazole (BO)

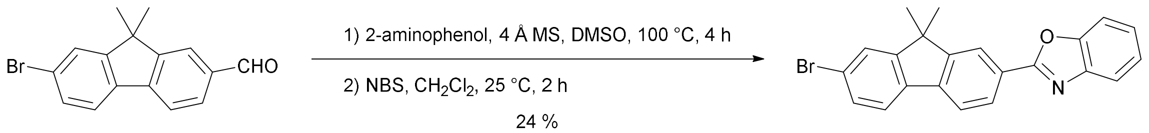

S3 **BO**

A modified literature procedure was used.[15] **S3** (1.51 g, 5.00 mmol, 1.00 eq) and 2-aminophenol (818 mg, 615 µL, 7.50 mmol, 1.50 eq) were dissolved in DMSO (12.5 mL, 0.4 M) and 4 Å MS (5.00 g) was added. The mixture was heated to 100 °C (oil bath temperature) and slowly stirred under these conditions for four hours. After cooling to ambient temperature, the solvent was lyophilized and the residue redissolved in CH_2Cl_2 (12.5 mL, 0.4 M). NBS (1.33 g, 7.50 mmol, 1.50 eq) was added in one portion and stirring was continued for another two hours at 25 °C. All solids were filtered off and the organic phase was washed with saturated $NaHCO_3$ solution (3 × 20 mL). The combined aqueous phases were extracted with CH_2Cl_2 (2 × 20 mL). All organic phases were combined and washed with saturated $Na_2S_2O_3$ solution (20 mL) and the aqueous phase was extracted with CH_2Cl_2 (20 mL). The combined organic phases were dried over anhydrous $MgSO_4$, filtered and concentrated under reduced pressure on a rotary evaporator. Purification by flash column chromatography (SiO_2, CH:EE 40:1 to 20:1 v:v) afforded **BO** (468 mg, 1.20 mmol, 24 %) as a yellowish solid.

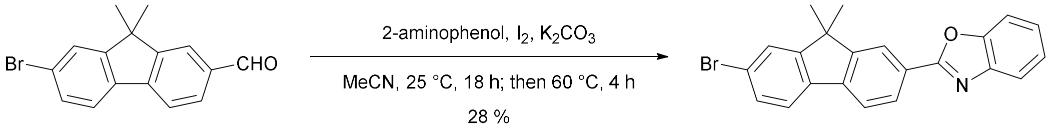

S3 **BO**

A modified literature procedure was used.[17] **S3** (301 mg, 1.00 mmol, 1.00 eq) and 2-aminophenol (120 mg, 90.3 µL, 1.10 mmol, 1.10 eq) were dissolved in MeCN (5.0 mL, 0.2 M). K_2CO_3 (415 mg, 3.00 mmol, 3.00 eq) and I_2 (508 mg, 2.00 mmol, 2.00 eq) were added successively and the mixture was stirred at 25 °C for 18 hours. Due to incomplete conversion of the intermediately formed *N*,*O*-acetale the mixture was heated to 60 °C for four hours. After cooling to ambient temperature solids were filtered off and the organic phase was diluted with CH_2Cl_2 (30 mL). Washing with saturated $Na_2S_2O_3$ solution (30 mL) was followed by extraction of the aqueous phase with CH_2Cl_2 (4 × 20 mL). The combined organic phases were dried over anhydrous $MgSO_4$, filtered and concentrated under reduced pressure on a rotary evaporator. Purification by flash column chromatography (SiO_2, CH:EE 30:1 v:v) afforded **BO** (282 mg, 282 µmol, 28 %) as a colorless solid.

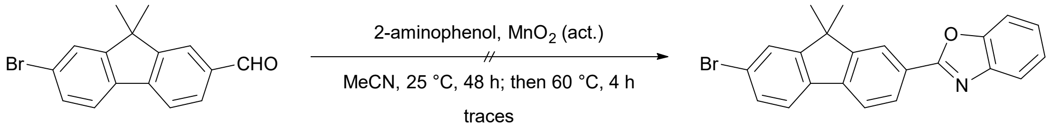

S3 **BO**

A modified literature procedure was used.[18] **S3** (301 mg, 1.00 mmol, 1.00 eq) and 2-aminophenol (120 mg, 90.3 µL, 1.10 mmol, 1.10 eq) were dissolved in MeCN (5.0 mL, 0.2 M). MnO_2 (174 mg, 2.00 mmol, 2.00 eq) was added and the mixture was stirred at 25 °C for three days. Due to low conversion to the desired product the mixture was heated to 60 °C for four hours, but the product formation remained very low, while no other side products were formed.

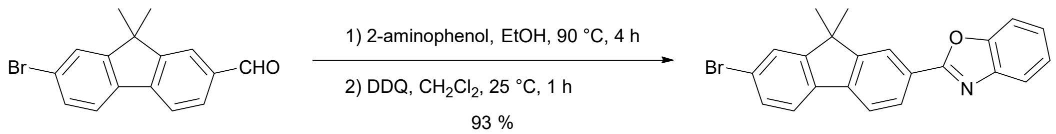

S3 **BO**

A modified literature procedure was used.[19] **S3** (602 mg, 2.00 mmol, 1.00 eq) and 2-aminophenol (218 mg, 164 µL, 2.00 mmol, 1.00 eq) were dissolved in EtOH (10 mL, 0.2 M). The mixture was heated to 90 °C (oil bath temperature) and stirred under these conditions for four hours to give a red/orange solution. After cooling to ambient temperature, the solvent was removed on a rotary evaporator and the residue redissolved in CH_2Cl_2 (10 mL, 0.2 M). DDQ (499 mg, 2.20 mmol, 1.10 eq) suspended in CH_2Cl_2 (10 mL) was added in one portion and stirring was continued for another one hour at 25 °C. Dilution with CH_2Cl_2 (20 mL) was followed by washing with saturated $NaHCO_3$ solution (2 × 20 mL). The combined aqueous phases were extracted with CH_2Cl_2 (2 × 10 mL). The combined organic phases were dried over anhydrous Na_2SO_4, filtered and concentrated under reduced pressure on a rotary evaporator. Purification by flash column chromatography (SiO_2, CH:EE 20:1 v:v) afforded **BO** (723 mg, 1.85 mmol, 93 %) as a colorless solid. Additionally, purification by recrystallization in hot methanol can be conducted to obtain **BO** as a colorless solid.

$\mathbf{R}_f$ = 0.08 (SiO_2, CH:EE 40:1 v:v). $\mathbf{R}_f$ = 0.12 (SiO_2, CH:EE 30:1 v:v). $\mathbf{R}_f$ = 0.28 (SiO_2, CH:EE 20:1 v:v). **Mp.:** 143 – 145 °C. **^{1}H-NMR (360 MHz, $CDCl_3$):** δ = 8.35 – 8.32 (m, 1H), 8.26 (dt, $^3J_{HH}$ = 8.0 Hz, $^4J_{HH}$ = 1.2 Hz, 1H), 7.82 (d, $^3J_{HH}$ = 8.1 Hz, 1H), 7.81 – 7.77 (m, 1H), 7.64 (d, $^3J_{HH}$ = 8.1 Hz, 1H), 7.62 – 7.58 (m, 2H), 7.53 – 7.49 (m, 1H), 7.40 – 7.34 (m, 2H), 1.57 (s, 6H) ppm. **^{13}C{^{1}H}-NMR (91 MHz, $CDCl_3$):** δ = 163.5, 156.5, 154.0, 150.9, 142.3, 141.7, 137.3, 130.6, 127.2, 126.5, 126.3, 125.2, 124.8, 122.4, 122.2, 122.2, 120.6, 120.0, 110.7, 47.5, 27.0 (2x) ppm. **IR (ATR, neat):** $\tilde{\nu}$ = 2957 (w), 2919 (w), 2898 (w), 2856 (w), 1615 (w), 1600 (w), 1558 (m), 1548 (m), 1469 (w), 1449 (m), 1412 (m), 1401 (m), 1360 (w), 1352 (w), 1335 (w), 1310 (w), 1297 (w), 1261 (w), 1241 (m), 1192 (m), 1181 (m), 1139 (w), 1107 (w), 1084 (w), 1060 (m), 1048 (m), 1001 (m), 938 (w), 924 (m), 906 (w), 898 (w), 892 (w), 879 (w), 847 (m), 821 (s), 775 (m), 761 (s), 745 (s), 738 (s), 674 (m) cm^{-1}. **MS (APCI):** m/z = 390.1 $[C_{22}H_{16}{}^{79}BrNO+H]^+$. **HR-MS (ESI):** calculated for $C_{22}H_{17}{}^{79}BrNO^+$ $[M+H]^+$: m/z = 390.04880, found: 390.04883 (Dev.: 0.03 mu; 0.08 ppm).

2.5.2 2-(Benzo[*d*]oxazol-2-yl)-7-bromo-9,9-dimethyl-9*H*-fluoren-3-ol (BO-OH)

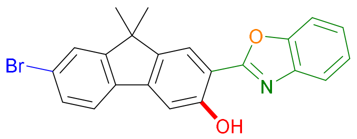

BO-OH

Prepared according to **GP 1** from **BO** (195 mg, 500 µmol, 1.00 eq) in THF (1.25 mL, 0.4 M) and TMPMgCl·LiCl (1.25 mL, 1.50 mmol, 3.00 eq, 1.20 M in THF). Stirring for four hours was followed by flushing with oxygen and stirring for another 24 hours. Saturated NH_4Cl solution (10 mL), water (10 mL) and CH_2Cl_2 (20 mL) were added and the phases were separated. The aqueous phase was extracted with CH_2Cl_2 (4 × 20 mL). Purification by flash column chromatography (SiO_2, CH:EE 1:0 to 40:1 v:v) afforded **BO-OH** (71.9 mg, 177 µmol, 35 %) as a slightly yellow solid. Due to recovered starting material (81.6 mg, 209 µmol) the BRSM yield is determined to be 61 %. Single crystals suitable for X-ray analysis were grown from CH_2Cl_2 solution by slow solvent evaporation.

Prepared according to **GP 2** from **BO** (78.1 mg, 200 µmol, 1.00 eq) in 1,4-dioxane (2.00 mL, 0.1 M), $Pd(OAc)_2$ (1.4 mg, 6.0 µmol, 3 mol%) and $Na_2S_2O_8$ (59.5 mg, 250 µmol, 1.25 eq). Stirring for 16 hours was followed by cooling to ambient temperature and dilution with ethyl acetate (10 mL). Saturated NH_4Cl solution (10 mL) and water (10 mL) were added and the phases were separated. The aqueous phase was extracted with ethyl acetate (4 × 20 mL). Purification by flash column chromatography (SiO_2, CH:EE 1:0 to 40:1 v:v) afforded **BO-OH** (40.7 mg, 100 µmol, 50 %) as a slightly yellow solid. Due to recovered starting material (28.6 mg, 73.4 µmol) the BRSM yield is determined to be 79 %.

R*f* = 0.04 (SiO_2, CH). **R*f*** = 0.34 (SiO_2, CH:EE 20:1 v:v). **Mp.:** 202 – 204 °C. **^{1}H-NMR (360 MHz, $CDCl_3$):** δ = 11.64 (br. s, 1H), 8.03 (s, 1H), 7.75 – 7.70 (m, 1H), 7.64 – 7.57 (m, 3H), 7.49 (dd, $^3J_{HH}$ = 8.2 Hz, $^4J_{HH}$ = 1.8 Hz, 1H), 7.41 – 7.35 (m, 3H), 1.55 (s, 6H) ppm. **^{13}C{^{1}H}-NMR (91 MHz, $CDCl_3$):** δ = 163.4, 158.9, 157.1, 149.2, 144.4, 143.8, 140.2, 137.2, 130.6, 126.5, 125.4, 125.2, 122.8, 122.5, 120.9, 119.3, 110.7, 109.5, 108.6, 46.8, 27.5 (2x) ppm. **IR (ATR, neat):** $\tilde{\nu}$ = 3060 (w), 2957 (w), 2921 (w), 2857 (w), 2811 (br), 1635 (m), 1590 (m), 1550 (m), 1471 (m), 1451 (m), 1406 (w), 1390 (m), 1359 (w), 1339 (w), 1306 (w), 1287 (w), 1279 (w), 1262 (m), 1246 (m), 1205 (m), 1142 (w), 1130 (w), 1109 (w), 1091 (m), 1061 (m), 1047 (m), 1001 (w), 963 (w), 948 (w), 926 (w), 911 (m), 897 (m), 879 (m), 869 (m), 825 (s), 800 (m), 762 (s), 739 (s), 725 (m), 696 (s), 672 (m) cm^{-1}. **MS (APCI):** m/z = 406.1 [$C_{22}H_{16}{}^{79}BrNO_2$+H]$^+$. **HR-MS (ESI):** calculated for $C_{22}H_{17}{}^{79}BrNO_2^+$ [M+H]$^+$: m/z = 406.04372, found: 406.04351 (Dev.: –0.20 mu; –0.50 ppm).

2.6 Preparation of 7-Bromo-9,9-dimethyl-2-(naphtho[2,3-*d*]oxazol-2-yl)-9*H*-fluoren-3-ol (NO-OH)

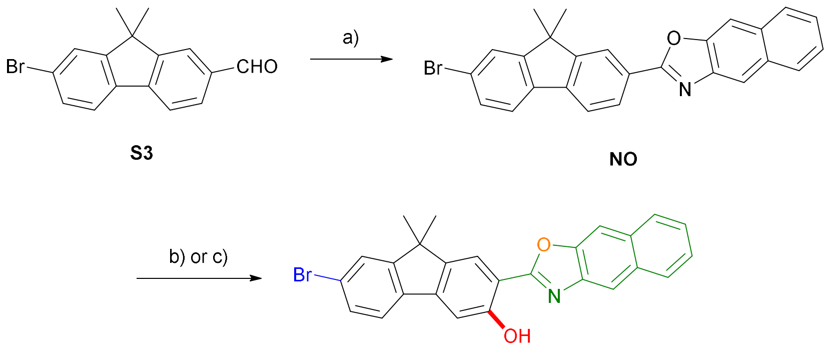

NO-OH

Reaction conditions: a) 1) 3-amino-2-naphthol, EtOH, 90 °C, 6 h; 2) DDQ, CH_2Cl_2, 25 °C, 1 h, 45 %; b) 1) TMPMgCl·LiCl, THF, 25 °C, 4 h; 2) O_2, 25 °C, 24 h, 29 % (51 % BRSM); c) $Pd(OAc)_2$, $Na_2S_2O_8$, 1,4-dioxane, 80 °C, 16 h, 33 % (71 % BRSM).

2.6.1 2-(7-Bromo-9,9-dimethyl-9*H*-fluoren-2-yl)naphtho[2,3-*d*]oxazole (NO)

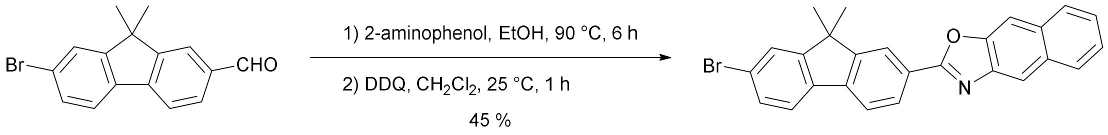

S3 **NO**

A modified literature procedure was used.[19] **S3** (602 mg, 2.00 mmol, 1.00 eq) and 3-amino-2-naphthol (318 mg, 2.00 mmol, 1.00 eq) were dissolved in EtOH (10 mL, 0.2 M). The mixture was heated to 90 °C (oil bath temperature) and stirred under these conditions for six hours to give a dark red solution. After cooling to ambient temperature, the solvent was removed on a rotary evaporator and the residue redissolved in CH_2Cl_2 (10 mL, 0.2 M). DDQ (499 mg, 2.20 mmol, 1.10 eq) suspended in CH_2Cl_2 (10 mL) was added in one portion and stirring was continued for another one hour at 25 °C. Dilution with CH_2Cl_2 (20 mL) was followed by washing with saturated $NaHCO_3$ solution (2 × 20 mL). The combined aqueous phases were extracted with CH_2Cl_2 (2 × 10 mL). The combined organic phases were dried over anhydrous Na_2SO_4, filtered and concentrated under reduced pressure on a rotary evaporator. Purification by flash column chromatography (SiO_2, CH:EE 25:1 v:v) afforded **NO** (394 mg, 895 µmol, 45 %) as a colorless solid.

R_f = 0.24 (SiO_2, CH:EE 25:1 v:v). **Mp.:** 232 – 234 °C. **^{1}H-NMR (360 MHz, $CDCl_3$):** δ = 8.40 (d, $^4J_{HH}$ = 1.5 Hz, 1H), 8.32 (dd, $^3J_{HH}$ = 8.0 Hz, $^4J_{HH}$ = 1.6 Hz, 1H), 8.21 (s, 1H), 8.05 – 8.00 (m, 1H), 7.99 – 7.94 (m, 2H), 7.84 (d, $^3J_{HH}$ = 8.0 Hz, 1H), 7.66 – 7.60 (m, 2H), 7.54 – 7.45 (m, 3H), 1.59 (s, 6H) ppm. **$^{13}C\{^1H\}$-NMR (91 MHz, $CDCl_3$):** δ = 165.5, 156.6, 154.0, 149.8, 142.3, 142.2, 137.2, 131.9, 131.7, 130.6, 128.7, 128.1, 127.8, 126.5, 125.9, 125.6, 124.9, 122.6 (2x), 122.3, 120.7, 117.2, 106.4, 47.6, 27.0

(2x) ppm. **IR (ATR, neat):** $\tilde{\nu}$ = 3051 (w), 2965 (w), 2923 (w), 2859 (w), 1694 (w), 1615 (w), 1557 (m), 1505 (w), 1463 (w), 1443 (w), 1401 (m), 1360 (w), 1349 (w), 1315 (w), 1306 (w), 1259 (w), 1246 (m), 1210 (w), 1153 (w), 1135 (w), 1105 (w), 1083 (w), 1059 (w), 1046 (m), 1016 (w), 1005 (w), 968 (w), 938 (w), 923 (w), 909 (w), 899 (w), 858 (s), 814 (s), 796 (m), 771 (m), 761 (w), 745 (m), 733 (s), 716 (s), 675 (w) cm^{-1}. **MS (APCI):** m/z = 440.1 [$C_{26}H_{18}{}^{79}BrNO+H]^+$. **HR-MS (ESI):** calculated for $C_{26}H_{19}{}^{79}BrNO^+$ [M+H]$^+$: m/z = 440.06445, found: 440.06495 (Dev.: 0.50 mu; 1.13 ppm).

2.6.2 7-Bromo-9,9-dimethyl-2-(naphtho[2,3-*d*]oxazol-2-yl)-9*H*-fluoren-3-ol (NO-OH)

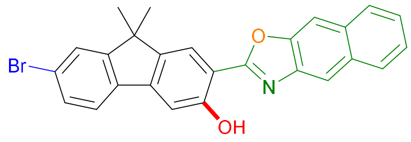

NO-OH

Prepared according to **GP 1** from **NO** (88.1 mg, 200 µmol, 1.00 eq) in THF (500 µL, 0.4 M) and TMPMgCl·LiCl (500 µL, 600 µmol, 3.00 eq, 1.20 M in THF). Stirring for four hours was followed by flushing with oxygen and stirring for another 24 hours. Saturated NH_4Cl solution (10 mL), water (10 mL) and CH_2Cl_2 (20 mL) were added and the phases were separated. The aqueous phase was extracted with CH_2Cl_2 (4 × 20 mL). Purification by flash column chromatography (SiO_2, CH:EE 1:0 to 40:1 to 20:1 v:v) afforded **NO-OH** (26.3 mg, 57.6 µmol, 29 %) as a yellow solid. Due to recovered starting material (38.0 mg, 86.2 µmol) the BRSM yield is determined to be 51 %. Single crystals suitable for X-ray analysis were grown from $CHCl_3$ solution by slow solvent evaporation.

Prepared according to **GP 2** from **NO** (88.1 mg, 200 µmol, 1.00 eq) in 1,4-dioxane (2.00 mL, 0.1 M), $Pd(OAc)_2$ (1.4 mg, 6.0 µmol, 3 mol%) and $Na_2S_2O_8$ (59.5 mg, 250 µmol, 1.25 eq). Stirring for 16 hours was followed by cooling to ambient temperature and dilution with ethyl acetate (10 mL). Saturated NH_4Cl solution (10 mL) and water (10 mL) were added and the phases were separated. The aqueous phase was extracted with ethyl acetate (4 × 20 mL). Purification by flash column chromatography (SiO_2, CH:EE 1:0 to 40:1 v:v) afforded **NO-OH** (30.5 mg, 66.8 µmol, 33 %) as a yellow solid. Due to recovered starting material (46.7 mg, 106 µmol) the BRSM yield is determined to be 71 %.

R*f* = 0.03 (SiO_2, CH). **R*f*** = 0.14 (SiO_2, CH:EE 40:1 v:v). **R*f*** = 0.26 (SiO_2, CH:EE 20:1 v:v). **Mp.:** 248 – 258 °C (long melting). **^{1}H-NMR (360 MHz, CDCl$_3$):** δ = 11.67 (br. s, 1H), 8.12 (s, 1H), 8.08 (s, 1H), 8.03 – 7.94 (m, 3H), 7.62 – 7.42 (m, 5H), 7.39 (s, 1H), 1.57 (s, 6H) ppm. **^{13}C{^{1}H}-NMR (91 MHz, CDCl$_3$):** δ = 165.4, 159.6, 157.2, 148.1, 144.5 (2x), 140.0, 137.1, 131.8 (2x), 130.6, 128.6, 128.1, 126.5, 125.8, 125.2, 123.0, 122.6, 121.3, 116.5, 109.2, 108.7, 106.5, 46.9, 27.5 (2x) ppm. **IR (ATR, neat):** $\tilde{\nu}$ = 3026 (w), 2955 (w), 2917 (m), 2849 (w), 2660 (br), 1644 (w), 1628 (w), 1592 (m), 1556 (m), 1461 (m), 1439 (m), 1404 (m), 1391 (m), 1308 (w), 1284 (w), 1270 (m), 1249 (m), 1206 (m), 1166 (w), 1154 (w), 1135 (m), 1089 (m), 1061 (m), 1040 (m), 961 (m), 943 (w), 929 (m), 914 (w), 895 (w), 866 (m),

849 (s), 821 (m), 807 (s), 752 (s), 736 (s), 702 (s), 666 (s) cm^{-1}. **MS (APCI):** m/z = 456.1 $[C_{26}H_{18}{}^{79}BrNO_2+H]^+$. **HR-MS (ESI):** calculated for $C_{26}H_{19}{}^{79}BrNO_2^+$ $[M+H]^+$: m/z = 456.05937, found: 456.05852 (Dev.: –0.84 mu; –1.85 ppm).

2.7 Preparation of 2-(Benzo[*d*]thiazol-2-yl)-7-bromo-9,9-dimethyl-9*H*-fluoren-3-ol (BS-OH)

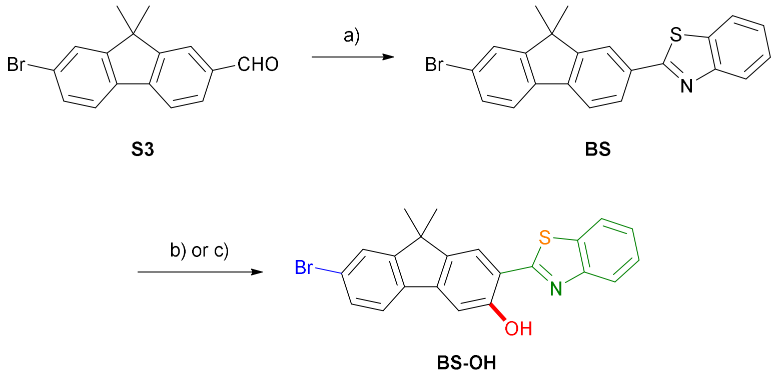

Reaction conditions: a) 2-aminothiophenol, DMSO, 150 °C, 1 h, 83 %; b) 1) TMPMgCl·LiCl, THF, 25 °C, 4 h; 2) O_2, 25 °C, 24 h, 27 % (52 % BRSM); c) $Pd(OAc)_2$, $Na_2S_2O_8$, 1,4-dioxane, 80 °C, 16 h, 42 % (82 % BRSM).

2.7.1 2-(7-Bromo-9,9-dimethyl-9*H*-fluoren-2-yl)benzo[*d*]thiazole (BS)

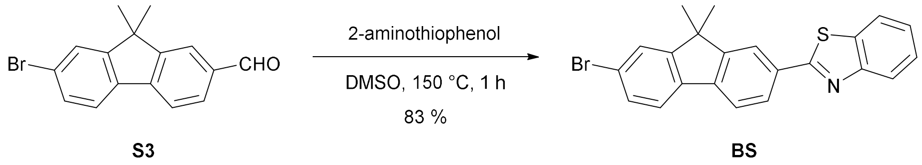

A modified literature procedure was used.[20] **S3** (2.41 g, 8.00 mmol, 1.00 eq) and 2-aminothiophenol (1.30 g, 1.11 mL, 10.4 mmol, 1.30 eq) were dissolved in DMSO (8.0 mL, 1.0 M). The mixture was heated to 150 °C (oil bath temperature) and stirred under these conditions for one hour to give a dark green solution. After cooling to ambient temperature, the solvent was lyophilized and the residue was diluted in CH_2Cl_2 (50 mL). Water (50 mL) was added and the phases were separated. The aqueous phase was extracted with CH_2Cl_2 (3 × 30 mL). The combined organic phases were dried over anhydrous Na_2SO_4, filtered and concentrated under reduced pressure on a rotary evaporator. Purification by flash column chromatography (SiO_2, CH:EE 30:1 v:v) afforded **BS** (2.70 g, 6.64 mmol, 83 %) as a colorless solid. Additionally, purification by recrystallization in hot cyclohexane can be conducted to obtain **BS** as a colorless solid.

R_f = 0.36 (SiO_2, CH:EE 30:1 v:v). **Mp.:** 166 – 168 °C. **^{1}H-NMR (360 MHz, $CDCl_3$):** δ = 8.21 (d, $^4J_{HH}$ = 1.6 Hz, 1H), 8.10 (dt, $^3J_{HH}$ = 8.3 Hz, $^4J_{HH}$ = 0.9 Hz, 1H), 8.04 (dd, $^3J_{HH}$ = 7.9 Hz, $^4J_{HH}$ = 1.6 Hz, 1H), 7.96 – 7.88 (m, 1H), 7.78 (d, $^3J_{HH}$ = 7.9 Hz, 1H), 7.63 (d, $^3J_{HH}$ = 8.1 Hz, 1H), 7.60 (d, $^4J_{HH}$ = 1.8 Hz, 1H), 7.54 – 7.48 (m, 2H), 7.43 – 7.38 (m, 1H), 1.57 (s, 6H) ppm. **^{13}C{^{1}H}-NMR (91 MHz, $CDCl_3$):**

δ = 168.4, 156.5, 154.3, 154.2, 141.2, 137.3, 135.1, 133.0, 130.5, 127.5, 126.5, 126.5, 125.3, 123.2, 122.2, 122.1, 121.7, 121.7, 120.6, 47.5, 27.1 (2x) ppm. **IR (ATR, neat):** $\tilde{\nu}$ = 2953 (w), 2916 (w), 2902 (w), 2858 (w), 1600 (w), 1568 (w), 1509 (m), 1475 (m), 1455 (m), 1434 (m), 1404 (m), 1381 (m), 1361 (w), 1339 (w), 1315 (m), 1287 (m), 1274 (w), 1252 (m), 1217 (m), 1203 (m), 1154 (m), 1135 (m), 1127 (w), 1119 (w), 1092 (m), 1084 (m), 1060 (m), 1016 (w), 1006 (m), 976 (s), 927 (m), 880 (m), 869 (m), 858 (m), 834 (m), 822 (m), 801 (s), 790 (m), 763 (m), 751 (s), 730 (s), 715 (s), 681 (s), 667 (m) cm^{-1}. **MS (APCI):** m/z = 406.1 $[C_{22}H_{16}{}^{79}BrNS+H]^+$. **HR-MS (ESI):** calculated for $C_{22}H_{17}{}^{79}BrNS^+$ $[M+H]^+$: m/z = 406.02596, found: 406.02559 (Dev.: –0.36 mu; –0.90 ppm); calculated for $C_{44}H_{32}{}^{79}Br_2N_2NaS_2{}^+$ $[2M+Na]^+$: m/z = 833.02659, found: 833.02538 (Dev.: –1.20 mu; –1.44 ppm).

2.7.2 2-(Benzo[*d*]thiazol-2-yl)-7-bromo-9,9-dimethyl-9*H*-fluoren-3-ol (BS-OH)

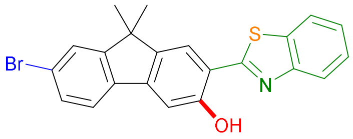

BS-OH

Prepared according to **GP 1** from **BS** (203 mg, 500 µmol, 1.00 eq) in THF (1.25 mL, 0.4 M) and TMPMgCl·LiCl (1.25 mL, 1.50 mmol, 3.00 eq, 1.20 M in THF). Stirring for four hours was followed by flushing with oxygen and stirring for another 24 hours. Saturated NH_4Cl solution (10 mL), water (10 mL) and CH_2Cl_2 (20 mL) were added and the phases were separated. The aqueous phase was extracted with CH_2Cl_2 (4 × 20 mL). Purification by flash column chromatography (SiO_2, CH:EE 1:0 to 80:1 v:v) afforded **BS-OH** (56.4 mg, 134 µmol, 27 %) as a yellow solid. Due to recovered starting material (97.9 mg, 241 µmol) the BRSM yield is determined to be 52 %. Single crystals suitable for X-ray analysis were grown from CH_2Cl_2 solution by slow solvent evaporation.

Prepared according to **GP 2** from **BS** (81.3 mg, 200 µmol, 1.00 eq) in 1,4-dioxane (2.00 mL, 0.1 M), $Pd(OAc)_2$ (1.4 mg, 6.0 µmol, 3 mol%) and $Na_2S_2O_8$ (59.5 mg, 250 µmol, 1.25 eq). Stirring for 16 hours was followed by cooling to ambient temperature and dilution with ethyl acetate (10 mL). Saturated NH_4Cl solution (10 mL) and water (10 mL) were added and the phases were separated. The aqueous phase was extracted with ethyl acetate (4 × 20 mL). Purification by flash column chromatography (SiO_2, CH:EE 1:0 to 80:1 v:v) afforded **BS-OH** (35.8 mg, 84.8 µmol, 42 %) as a yellow solid. Due to recovered starting material (39.0 mg, 96.1 µmol) the BRSM yield is determined to be 82 %.

R_f = 0.16 (SiO_2, CH). **R_f** = 0.31 (SiO_2, CH:EE 80:1 v:v). **Mp.:** 198 – 200 °C. **^{1}H-NMR (360 MHz, $CDCl_3$):** δ = 12.74 (s, 1H), 7.99 (ddd, $^3J_{HH}$ = 8.1 Hz, $^4J_{HH}$ = 1.2 Hz, $^5J_{HH}$ = 0.7 Hz, 1H), 7.91 (ddd, $^3J_{HH}$ = 8.0 Hz, $^4J_{HH}$ = 1.3 Hz, $^5J_{HH}$ = 0.6 Hz, 1H), 7.64 (s, 1H), 7.59 (d, $^3J_{HH}$ = 6.6 Hz, 1H), 7.57 (s, 1H), 7.54 – 7.47 (m, 2H), 7.41 (ddd, $^3J_{HH}$ = 8.2 Hz, $^3J_{HH}$ = 7.2 Hz, $^4J_{HH}$ = 1.2 Hz, 1H), 7.38 (s, 1H), 1.54 (s, 6H) ppm. **^{13}C{^{1}H}-NMR (91 MHz, $CDCl_3$):** δ = 169.7, 158.2, 157.0, 152.0, 144.4, 143.1, 137.2, 132.6,

130.6, 126.9, 126.4, 125.6, 122.6, 122.4, 122.2, 122.1, 121.6, 116.0, 109.0, 46.8, 27.6 (2x) ppm. **IR (ATR, neat):** $\tilde{\nu}$ = 2959 (w), 2919 (w), 2898 (w), 2859 (w), 2771 (br), 1633 (w), 1589 (m), 1506 (w), 1476 (m), 1455 (m), 1441 (m), 1408 (m), 1391 (m), 1359 (m), 1316 (w), 1301 (w), 1277 (w), 1247 (m), 1200 (s), 1160 (m), 1127 (m), 1085 (m), 1060 (m), 1020 (w), 992 (m), 937 (m), 910 (m), 867 (m), 855 (m), 820 (m), 810 (s), 757 (s), 743 (s), 722 (s), 696 (s), 686 (s) cm^{-1}. **MS (APCI):** m/z = 422.1 [$C_{22}H_{16}{}^{79}BrNOS+H]^+$. **HR-MS (ESI):** calculated for $C_{22}H_{17}{}^{79}BrNOS^+$ [M+H]$^+$: m/z = 422.02087, found: 422.02067 (Dev.: –0.20 mu; –0.48 ppm).

2.8 Preparation of 2-(Benzo[*d*][1,3]selenazol-2-yl)-7-bromo-9,9-dimethyl-9*H*-fluoren-3-ol (BSe-OH)

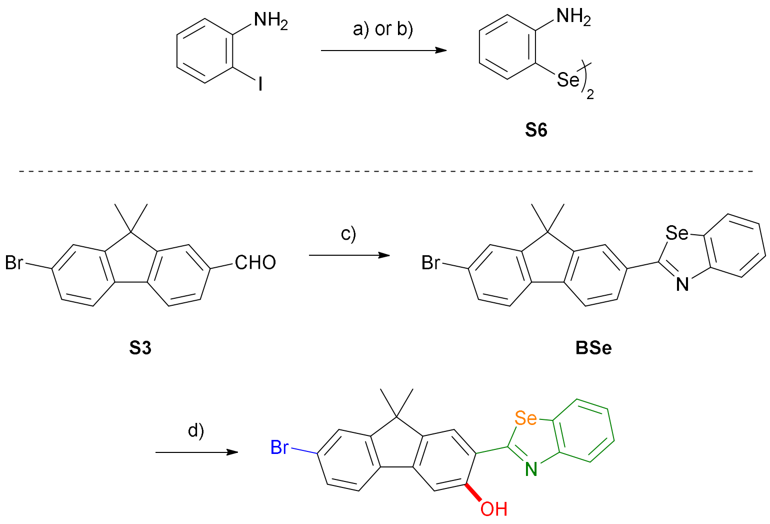

BSe-OH

Reaction conditions: a) 1) *n*BuLi, THF, –78 °C, 1 h; 2) Se^0, –78 °C to 25 °C, 12 h; 3) aq. $K_3[Fe(CN)_6]$, 25 °C, 2 h, 91 %; b) Se^0, CuO, KOH, DMSO, 90 °C, 24 h, 87 %; c) **S6**, $Na_2S_2O_5$, DMSO, 120 °C, 21 h, 70 %; d) $Pd(OAc)_2$, $Na_2S_2O_8$, 1,4-dioxane, 80 °C, 16 h, 25 % (61 % BRSM).

2.8.1 2,2'-Diselanediyldianiline (S6)

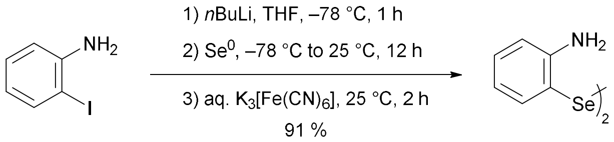

S6

A modified literature procedure was used.[21] A heat gun-dried and nitrogen-flushed *Schlenk* flask was charged with 2-iodoaniline (438 mg, 2.00 mmol, 1.00 eq) and anhydrous THF (8.0 mL, 0.25 M) was added. The solution was cooled to –78 °C and *n*BuLi (2.40 mL, 6.00 mmol, 3.00 eq, 2.5 M in hexane) was added *via* syringe pump (0.4 mL/min) through a rubber septum to the vigorously stirred mixture. The turbid, yellow solution was stirred for one hour at –78 °C and elemental selenium (158 mg, 2.00 mmol, 1.00 eq) was added in one portion. Stirring was continued for 12 hours while the mixture

was allowed to warm slowly to 25 °C. In a beaker, $K_3[Fe(CN)_6]$ (1.32 g, 4.00 mmol, 2.00 eq) was dissolved in water (60 mL) and the reaction mixture was poured into the aqueous solution. Stirring at 25 °C for two hours was followed by the addition of ethyl acetate (50 mL). The phases were separated and the aqueous layer was extracted with ethyl acetate (3 × 50 mL). The combined organic layers were dried over anhydrous Na_2SO_4, filtered and concentrated under reduced pressure on a rotary evaporator. Purification by flash column chromatography (SiO_2, CH:EE 6:1 v:v) afforded **S6** (312 mg, 912 µmol, 91 %) as a red oil, which solidified upon standing at 25 °C to give a red solid.

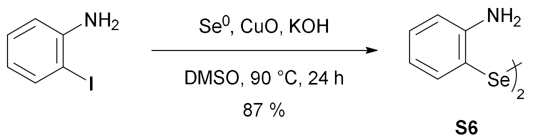

S6

A modified literature procedure was used.[22] A heat gun-dried and nitrogen-flushed *Schlenk* flask was charged with 2-iodoaniline (4.38 g, 20.0 mmol, 1.00 eq) and anhydrous DMSO (40 mL, 0.5 M) was added. Elemental selenium (3.16 g, 40.0 mmol, 2.00 eq), CuO (159 mg, 2.00 mmol, 0.10 eq) and KOH platelets (2.24 g, 40.0 mmol, 2.00 eq) were added successively at 25 °C and the mixture was heated to 90 °C (oil bath temperature) for 24 hours. After cooling to ambient temperature, the solvent was lyophilized and the residue was diluted in water (100 mL) and diethyl ether (200 mL) and insoluble solids were filtered off. The phases were separated and the aqueous layer was extracted with ethyl acetate (6 × 100 mL). The combined organic layers were dried over anhydrous Na_2SO_4, filtered and concentrated under reduced pressure on a rotary evaporator. Purification by flash column chromatography (SiO_2, CH:EE 6:1 v:v) afforded **S6** (2.97 g, 8.68 mmol, 87 %) as an orange/red solid.

R_f = 0.27 (SiO_2, CH:EE 6:1 v:v). **Mp.:** 72 – 74 °C. **^{1}H-NMR (360 MHz, $CDCl_3$):** δ = 7.35 (dd, $^3J_{HH}$ = 7.7 Hz, $^4J_{HH}$ = 1.4 Hz, 2H), 7.17 – 7.11 (m, 2H), 6.72 (dd, $^3J_{HH}$ = 8.0 Hz, $^4J_{HH}$ = 1.3 Hz, 2H), 6.56 (td, $^3J_{HH}$ = 7.7 Hz, $^4J_{HH}$ = 1.2 Hz, 2H), 4.25 (s, 4H) ppm. **^{13}C{^{1}H}-NMR (91 MHz, $CDCl_3$):** δ = 148.9 (2x), 138.4 (2x), 131.7 (2x), 118.6 (2x), 115.1 (2x), 114.9 (2x) ppm. **^{77}Se-NMR (69 MHz, $CDCl_3$):** δ = 405.9 (s) ppm. **IR (ATR, neat):** $\tilde{\nu}$ = 3411 (w), 3307 (w), 3175 (w), 3054 (w), 3010 (w), 2953 (w), 2918 (w), 2850 (w), 2779 (w), 2606 (w), 1941 (w), 1907 (w), 1599 (m), 1558 (m), 1516 (w), 1471 (s), 1442 (m), 1307 (m), 1249 (m), 1185 (w), 1159 (m), 1139 (m), 1079 (w), 1044 (w), 1015 (m), 962 (w), 935 (m), 907 (w), 889 (w), 848 (m), 831 (w), 744 (s) cm^{-1}. **MS (EI, 70 eV):** m/z = 343.9 $[C_{12}H_{12}N_2{}^{80}Se_2]^{+\cdot}$. **HR-MS (ESI):** calculated for $C_{12}H_{13}N_2{}^{80}Se_2{}^+$ $[M+H]^+$: m/z = 344.94060, found: 344.94045 (Dev.: –0.15 mu; –0.44 ppm); calculated for $C_{12}H_{12}N_2Na^{80}Se_2{}^+$ $[M+Na]^+$: m/z = 366.92255, found: 366.92231 (Dev.: –0.24 mu; –0.64 ppm). The analytical data are in accordance with the literature.[23]

2.8.2 2-(7-Bromo-9,9-dimethyl-9*H*-fluoren-2-yl)benzo[*d*][1,3]selenazole (BSe)

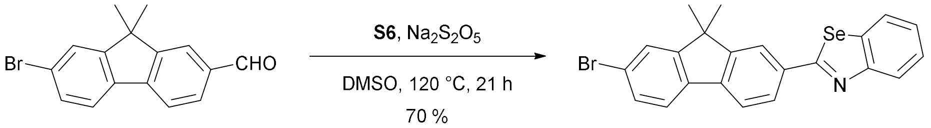

S3 **BSe**

A modified literature procedure was used.[24] A heat gun-dried and nitrogen-flushed *Schlenk* flask was charged with **S3** (1.81 g, 6.00 mmol, 1.00 eq) and anhydrous DMSO (20 mL, 0.3 M) was added. $Na_2S_2O_5$ (1.14 g, 6.00 mmol, 1.00 eq) and **S6** (1.03 g, 3.00 mmol, 0.50 eq) were added successively at 25 °C and the mixture was heated to 120 °C (oil bath temperature) for 21 hours. After cooling to ambient temperature, the solvent was lyophilized and the residue was diluted in water (100 mL) and CH_2Cl_2 (100 mL) and insoluble solids were filtered off. The phases were separated and the aqueous layer was extracted with CH_2Cl_2 (5 × 50 mL). The combined organic layers were dried over anhydrous Na_2SO_4, filtered and concentrated under reduced pressure on a rotary evaporator. Purification by flash column chromatography (SiO_2, CH:EE 50:1 v:v) afforded **BSe** (1.89 g, 4.17 mmol, 70 %) as a slightly yellow solid. Additionally, purification by recrystallization in hot methanol can be conducted to obtain **BSe** as a beige solid.

R_f = 0.30 (SiO_2, CH:EE 50:1 v:v). **Mp.:** 206 – 208 °C. **^{1}H-NMR (360 MHz, $CDCl_3$):** δ = 8.17 – 8.10 (m, 2H), 7.98 – 7.90 (m, 2H), 7.75 (dd, $^3J_{HH}$ = 8.1 Hz, $^4J_{HH}$ = 1.4 Hz, 1H), 7.63 (dd, $^3J_{HH}$ = 8.1 Hz, $^4J_{HH}$ = 1.3 Hz, 1H), 7.60 (d, $^4J_{HH}$ = 1.8 Hz, 1H), 7.53 – 7.47 (m, 2H), 7.33 (t, $^3J_{HH}$ = 7.6 Hz, 1H), 1.57 (s, 6H) ppm. **^{13}C{^{1}H}-NMR (91 MHz, $CDCl_3$):** δ = 172.8, 156.5, 155.9, 154.2, 141.4, 138.3, 137.3, 135.5, 130.6, 128.4, 126.6, 126.5, 125.4, 125.0, 124.8, 122.3, 122.1, 121.9, 120.7, 47.5, 27.1 (2x) ppm. **^{77}Se-NMR (69 MHz, $CDCl_3$):** δ = 616.3 (s) ppm. **IR (ATR, neat):** $\tilde{\nu}$ = 3058 (w), 3024 (w), 3001 (w), 2950 (w), 2914 (w), 2899 (w), 2856 (w), 1566 (w), 1517 (m), 1478 (w), 1458 (w), 1449 (w), 1435 (m), 1413 (m), 1402 (m), 1380 (w), 1360 (w), 1339 (w), 1307 (m), 1287 (w), 1272 (w), 1251 (m), 1217 (w), 1192 (m), 1153 (w), 1133 (w), 1116 (w), 1092 (w), 1082 (w), 1017 (w), 1004 (w), 960 (m), 933 (m), 926 (m), 874 (w), 865 (m), 859 (m), 843 (w), 834 (m), 801 (s), 778 (m), 763 (m), 749 (s), 730 (s), 708 (s), 671 (w), 664 (m) cm^{-1}. **MS (APCI):** m/z = 454.1 [$C_{22}H_{16}{}^{79}BrN^{80}Se$+H]$^+$. **HR-MS (ESI):** calculated for $C_{22}H_{17}{}^{79}BrN^{76}Se^+$ [M+H]$^+$: m/z = 449.97320, found: 449.97238 (Dev.: –0.82 mu; –1.82 ppm).

2.8.3 2-(Benzo[*d*][1,3]selenazol-2-yl)-7-bromo-9,9-dimethyl-9*H*-fluoren-3-ol (BSe-OH)

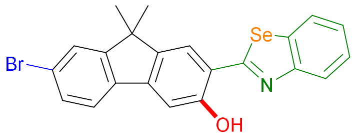

BSe-OH

Conducted according to **GP 1** from **BSe** (113 mg, 250 µmol, 1.00 eq) in THF (625 µL, 0.4 M) and TMPMgCl·LiCl (625 µL, 750 µmol, 3.00 eq, 1.20 M in THF). Stirring for four hours was followed by

flushing with oxygen and stirring for another 24 hours. No desired product was observed.

Prepared according to **GP 2** from **BSe** (90.7 mg, 200 µmol, 1.00 eq) in 1,4-dioxane (2.00 mL, 0.1 M), $Pd(OAc)_2$ (1.4 mg, 6.0 µmol, 3 mol%) and $Na_2S_2O_8$ (59.5 mg, 250 µmol, 1.25 eq). Stirring for 48 hours was followed by cooling to ambient temperature and dilution with ethyl acetate (10 mL). Saturated NH_4Cl solution (10 mL) and water (10 mL) were added and the phases were separated. The aqueous phase was extracted with ethyl acetate (4 × 20 mL). Purification by flash column chromatography (SiO_2, CH:EE 1:0 to 50:1 v:v) afforded **BSe-OH** (23.7 mg, 50.5 µmol, 25 %) as an orange solid. Due to recovered starting material (53.0 mg, 117 µmol) the BRSM yield is determined to be 61 %. Single crystals suitable for X-ray analysis were grown from CH_2Cl_2 solution by slow solvent evaporation.

R*f* = 0.22 (SiO_2, CH). **R*f*** = 0.38 (SiO_2, CH:EE 50:1 v:v). **Mp.:** 223 – 225 °C. **^{1}H-NMR (360 MHz, $CDCl_3$):** δ = 12.79 (br. s, 1H), 8.01 (d, $^3J_{HH}$ = 8.1 Hz, 1H), 7.93 (d, $^3J_{HH}$ = 7.9 Hz, 1H), 7.61 – 7.56 (m, 2H), 7.53 – 7.46 (m, 3H), 7.37 – 7.30 (m, 2H), 1.54 (s, 6H) ppm. **^{13}C{^{1}H}-NMR (91 MHz, $CDCl_3$):** δ = 174.2, 157.6, 157.1, 144.7, 137.4, 137.2, 131.8, 130.6, 127.0, 126.4, 125.7, 124.8, 123.7, 123.7, 123.6, 123.6, 122.7, 122.4, 108.9, 46.8, 27.6 (2x) ppm. **^{77}Se-NMR (69 MHz, $CDCl_3$):** δ = 602.3 (s) ppm. **IR (ATR, neat):** $\tilde{\nu}$ = 3058 (w), 3004 (w), 2958 (w), 2921 (w), 2858 (w), 2772 (br), 1723 (w), 1633 (w), 1590 (w), 1515 (w), 1478 (w), 1462 (w), 1453 (w), 1440 (m), 1410 (w), 1390 (m), 1358 (w), 1308 (w), 1287 (w), 1273 (w), 1257 (w), 1244 (w), 1224 (w), 1194 (m), 1173 (m), 1130 (w), 1119 (w), 1092 (w), 1084 (w), 1059 (w), 1049 (w), 1021 (w), 984 (m), 932 (w), 905 (m), 863 (m), 848 (m), 808 (s), 755 (s), 738 (s), 714 (s), 692 (s), 670 (m) cm^{-1}. **MS (APCI):** m/z = 470.1 [$C_{22}H_{16}{}^{79}BrNO^{80}Se+H$]$^+$. **HR-MS (ESI):** calculated for $C_{22}H_{17}{}^{79}BrNO^{76}Se^+$ [M+H]$^+$: m/z = 465.96812, found: 465.96825 (Dev.: 0.13 mu; 0.28 ppm).

3 Optical Properties

3.1 2-(7-Bromo-9,9-dimethyl-9*H*-fluoren-2-yl)-4,4-dimethyl-4,5-dihydrooxazole (Oxa)

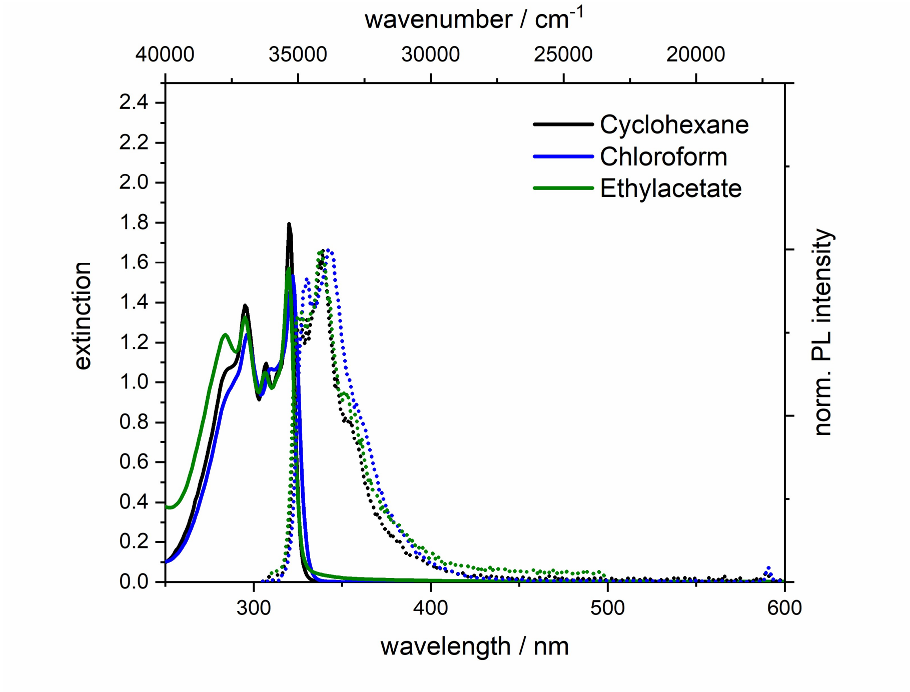

Figure S1: Optical Properties of compound **Oxa** in different solvents, extinction (solid line) and emission (dotted line).

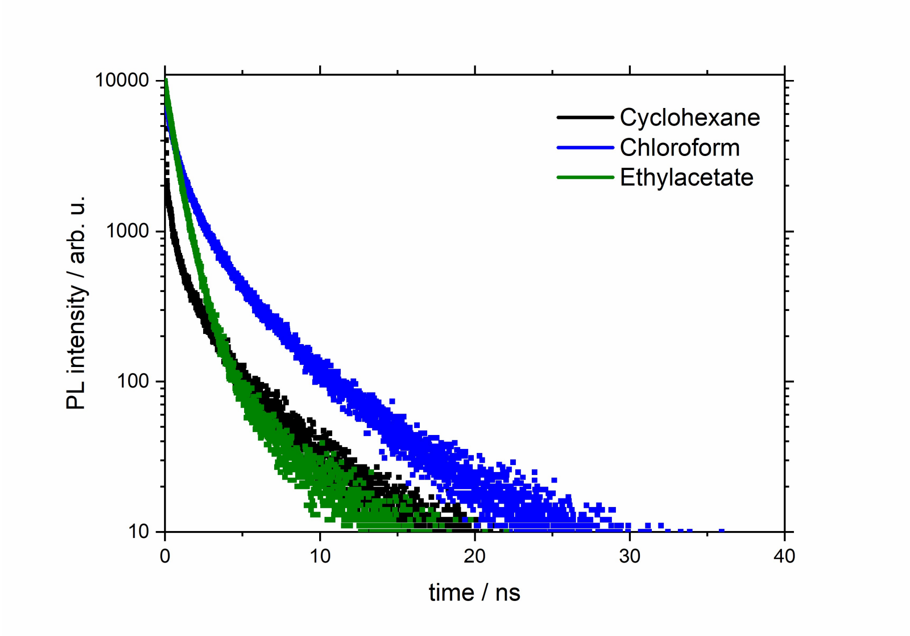

Figure S2: Fluorescence decay of compound **Oxa** in different solvents.

3.2 7-Bromo-2-(4,4-dimethyl-4,5-dihydrooxazol-2-yl)-9,9-dimethyl-9*H*-fluoren-3-ol (Oxa-OH)

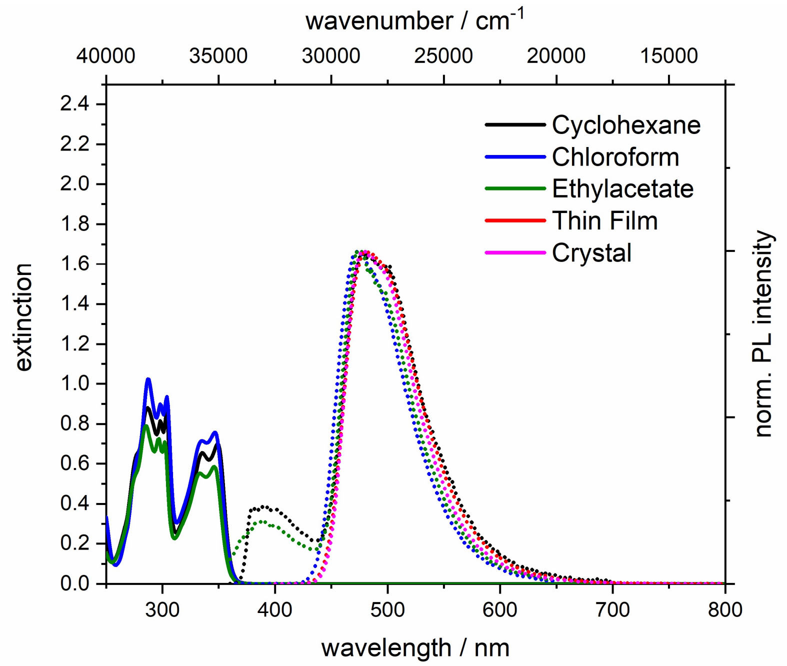

Figure S3: Optical Properties of compound **Oxa-OH** in different solvents and in solid state, extinction (solid line) and emission (dotted line).

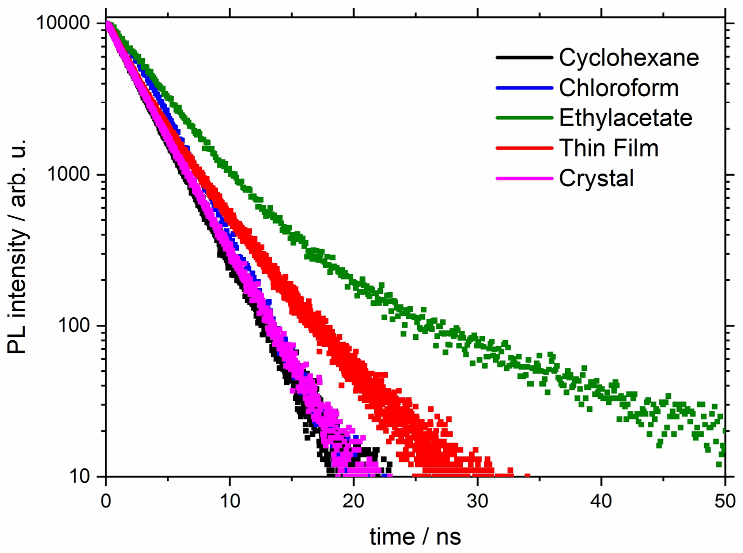

Figure S4: Fluorescence decay of compound **Oxa-OH** in different solvents and solid state.

3.3 2,2'-(9,9-Dimethyl-9*H*-fluorene-2,7-diyl)bis(4,4-dimethyl-4,5-dihydrooxazole) ((Oxa)$_2$)

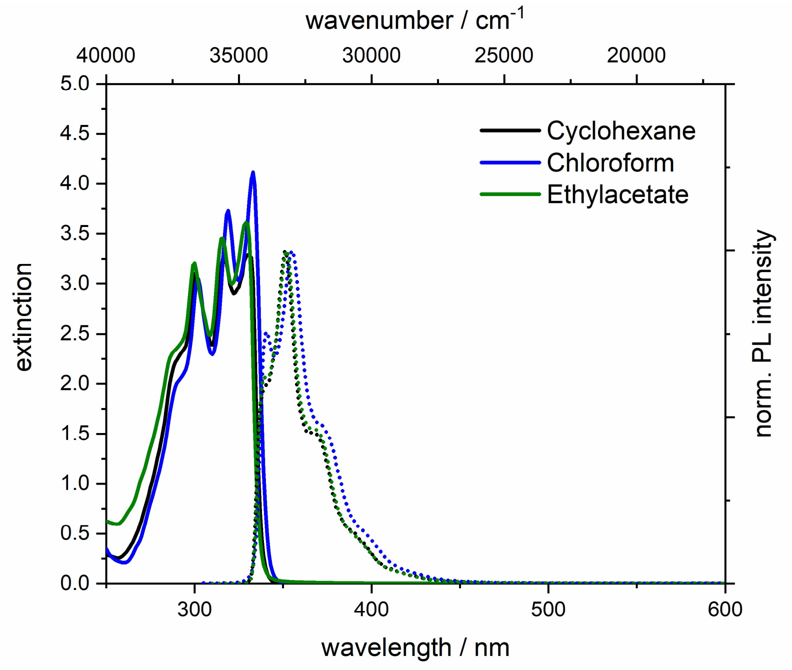

Figure S5: Optical Properties of compound **(Oxa)$_2$** in different solvents, extinction (solid line) and emission (dotted line).

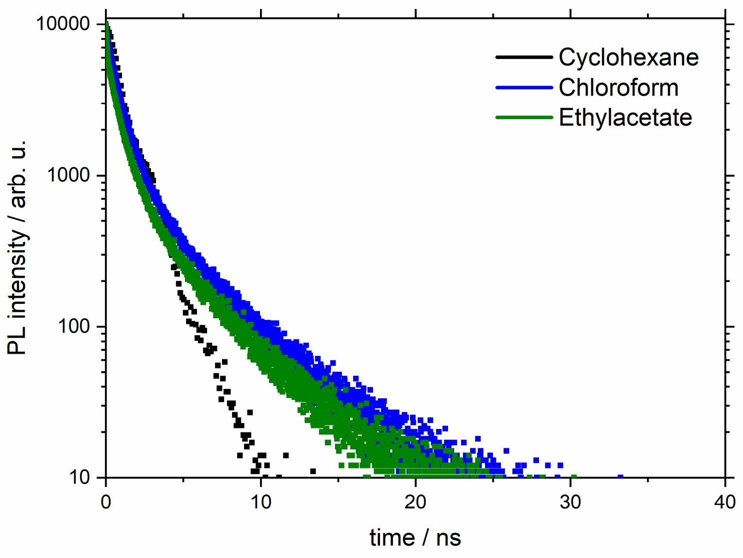

Figure S6: Fluorescence decay of compound **(Oxa)$_2$** in different solvents.

3.4 2,7-Bis(4,4-dimethyl-4,5-dihydrooxazol-2-yl)-9,9-dimethyl-9*H*-fluoren-3-ol ((Oxa)$_2$-OH)

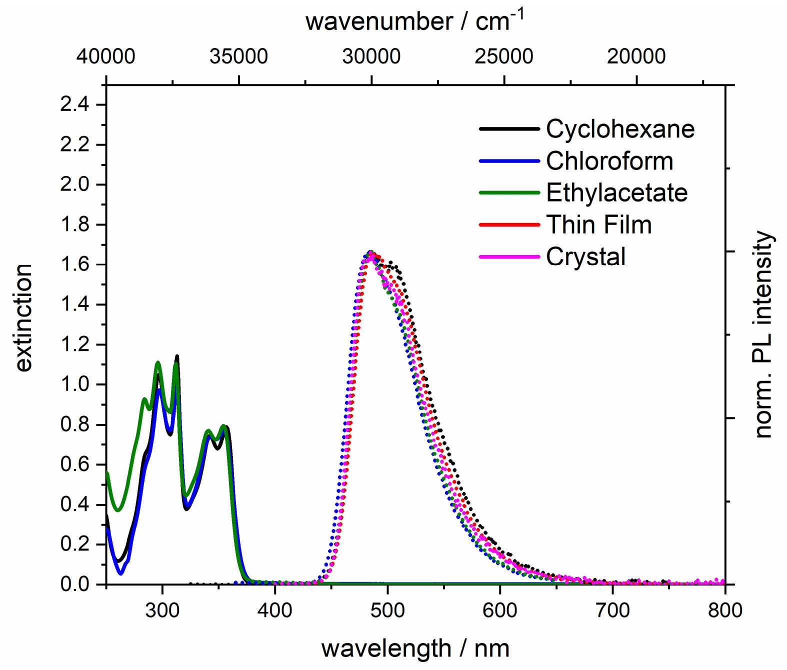

Figure S7: Optical Properties of compound **(Oxa)$_2$-OH** in different solvents and in solid state, extinction (solid line) and emission (dotted line).

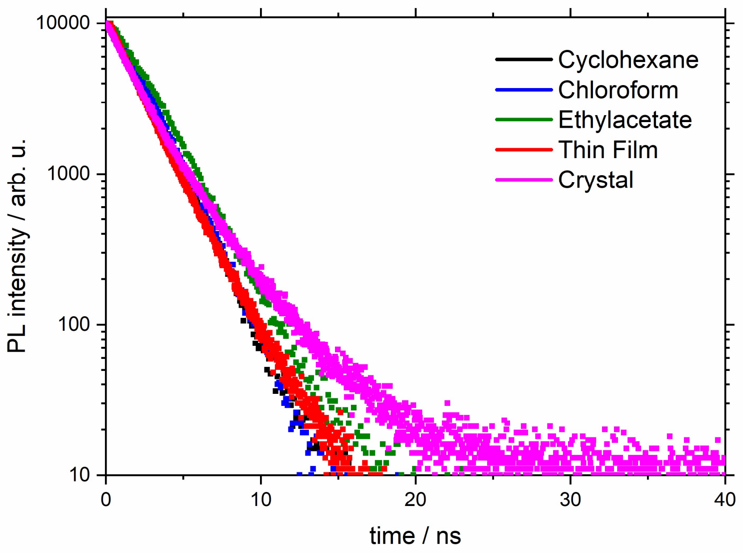

Figure S8: Fluorescence decay of compound **(Oxa)$_2$-OH** in different solvents and solid state.

3.5 2,7-Bis(4,4-dimethyl-4,5-dihydrooxazol-2-yl)-9,9-dimethyl-9*H*-fluorene-3,6-diol ((Oxa-OH)$_2$)

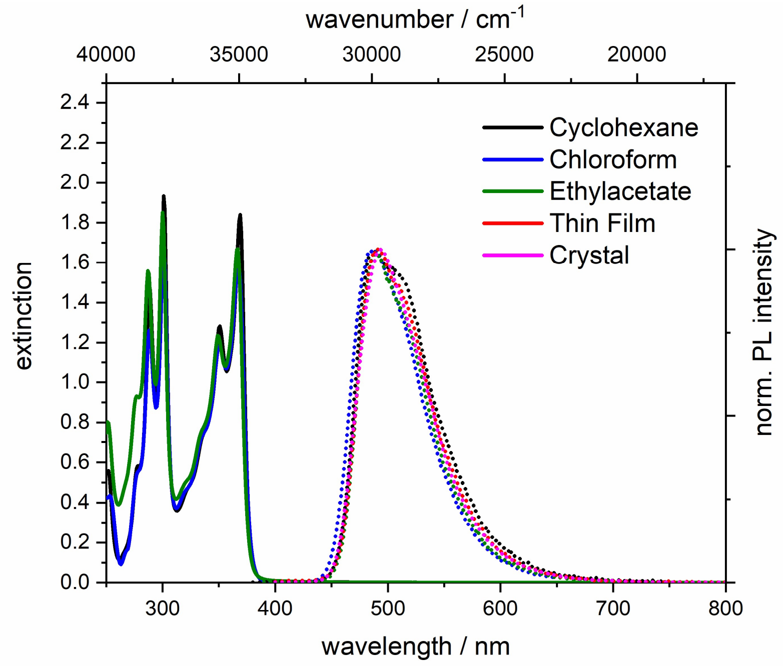

Figure S9: Optical Properties of compound **(Oxa-OH)$_2$** in different solvents and in solid state, extinction (solid line) and emission (dotted line).

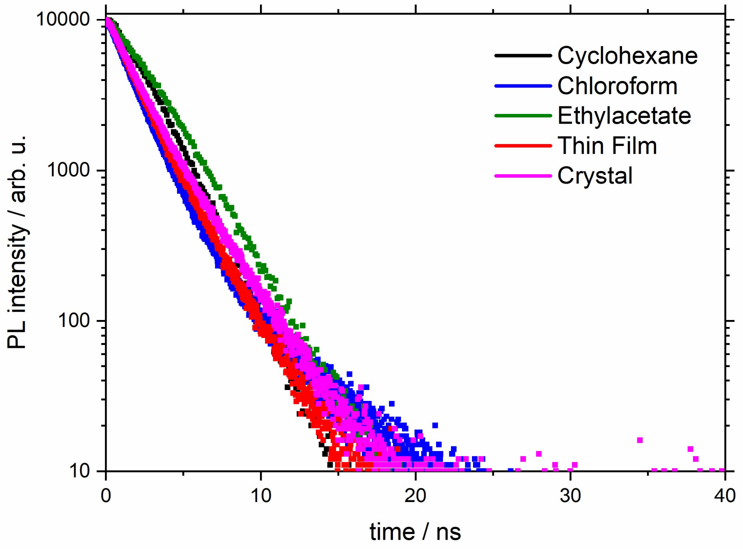

Figure S10: Fluorescence decay of compound **(Oxa-OH)$_2$** in different solvents and solid state.

3.6 2-(7-Bromo-9,9-dimethyl-9*H*-fluoren-2-yl)benzo[*d*]oxazole (BO)

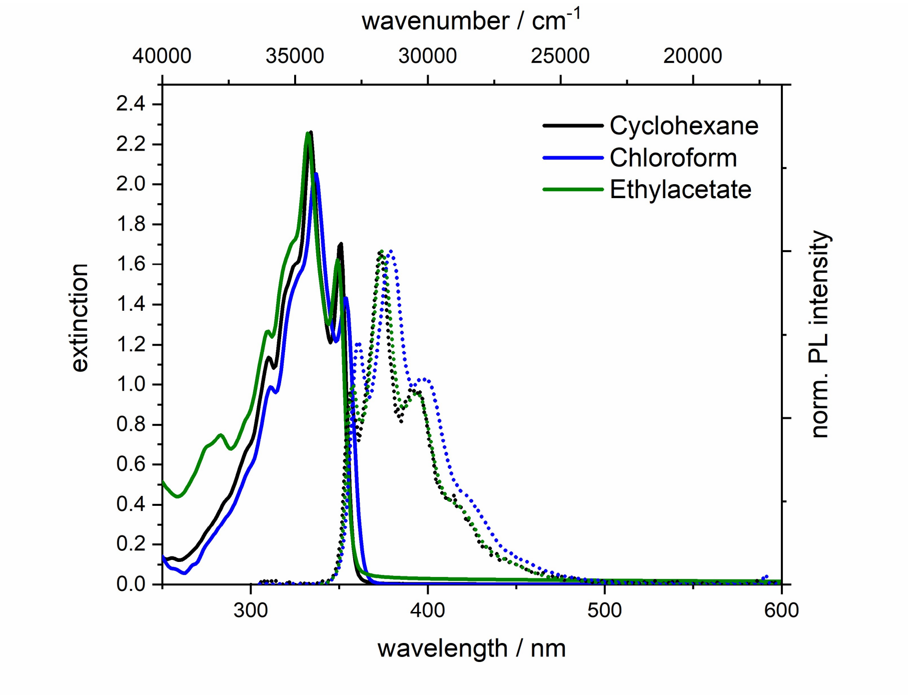

Figure S11: Optical Properties of compound **BO** in different solvents, extinction (solid line) and emission (dotted line).

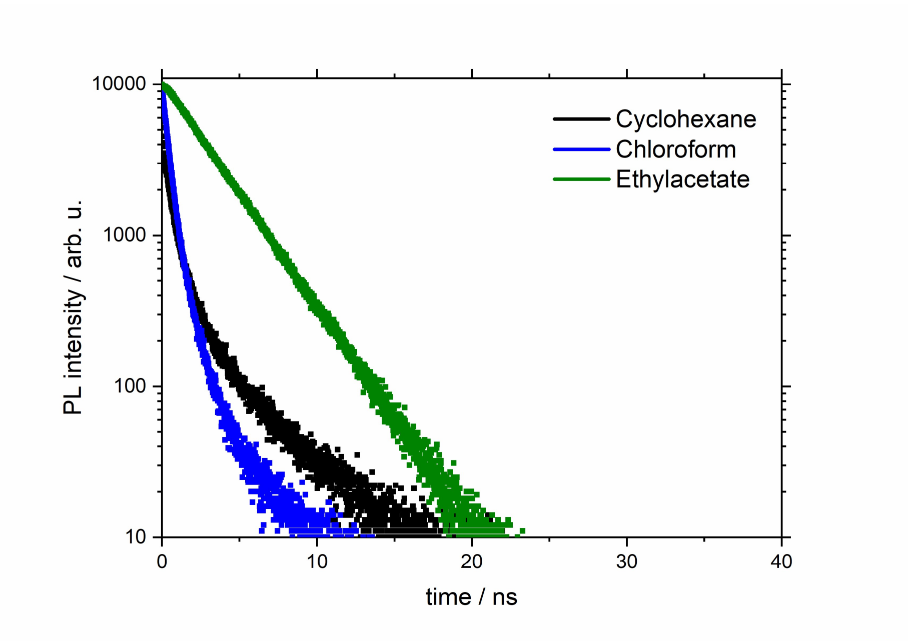

Figure S12: Fluorescence decay of compound **BO** in different solvents.

3.7 2-(Benzo[*d*]oxazol-2-yl)-7-bromo-9,9-dimethyl-9*H*-fluoren-3-ol (BO-OH)

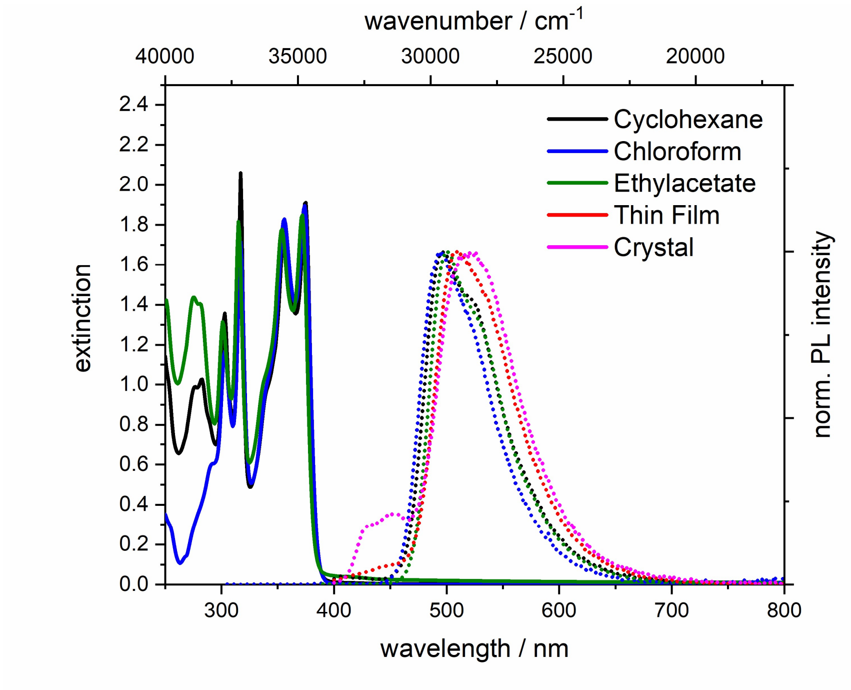

Figure S13: Optical Properties of compound **BO-OH** in different solvents and in solid state, extinction (solid line) and emission (dotted line).

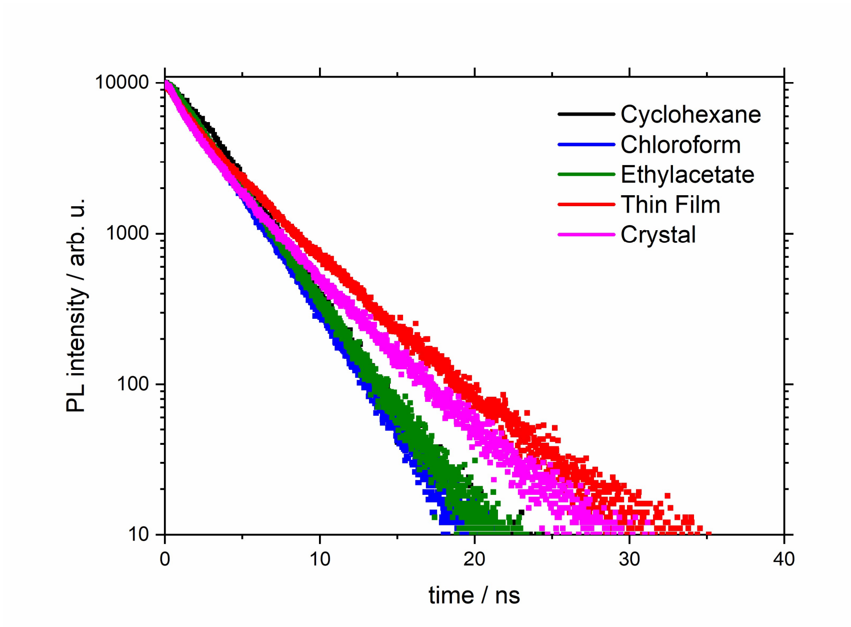

Figure S14: Fluorescence decay of compound **BO-OH** in different solvents and solid state.

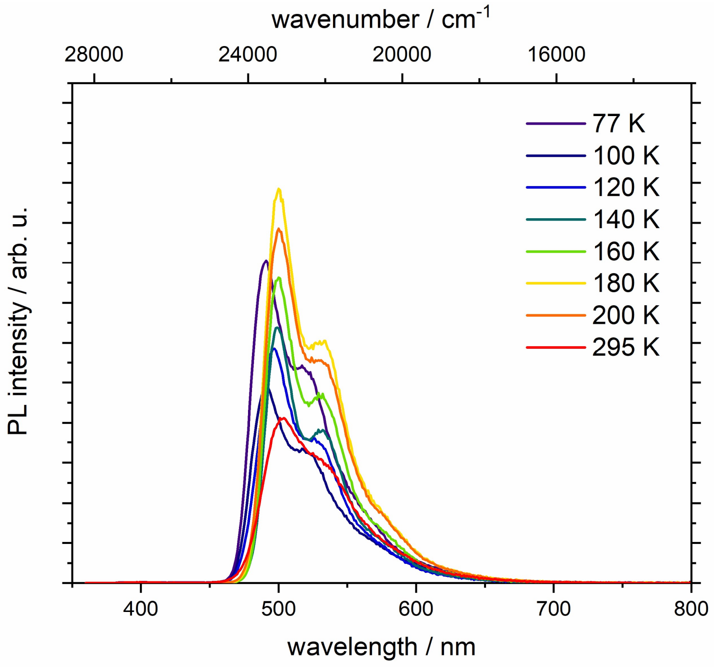

Figure S15: Temperature-dependent emission spectra of compound **BO-OH** in 2-methyltetrahydrofuran.

3.8 2-(7-Bromo-9,9-dimethyl-9*H*-fluoren-2-yl)naphtho[2,3-*d*]oxazole (NO)

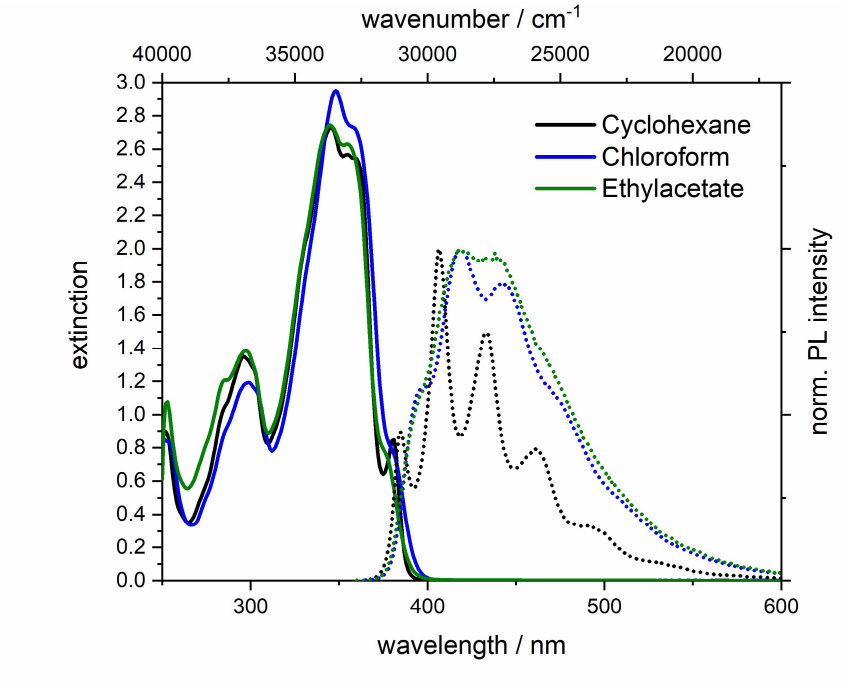

Figure S16: Optical Properties of compound **NO** in different solvents, extinction (solid line) and emission (dotted line).

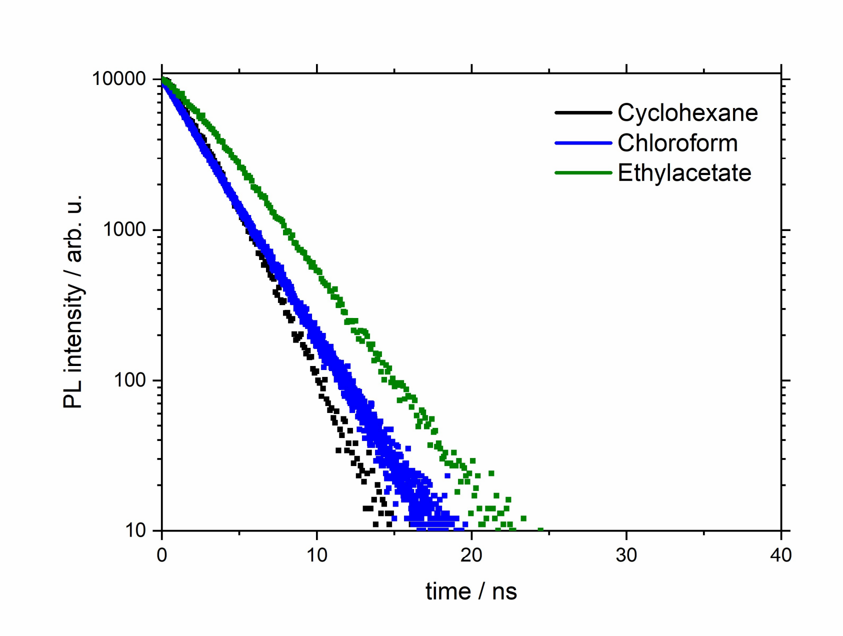

Figure S17: Fluorescence decay of compound **NO** in different solvents.

3.9 7-Bromo-9,9-dimethyl-2-(naphtho[2,3-*d*]oxazol-2-yl)-9*H*-fluoren-3-ol (NO-OH)

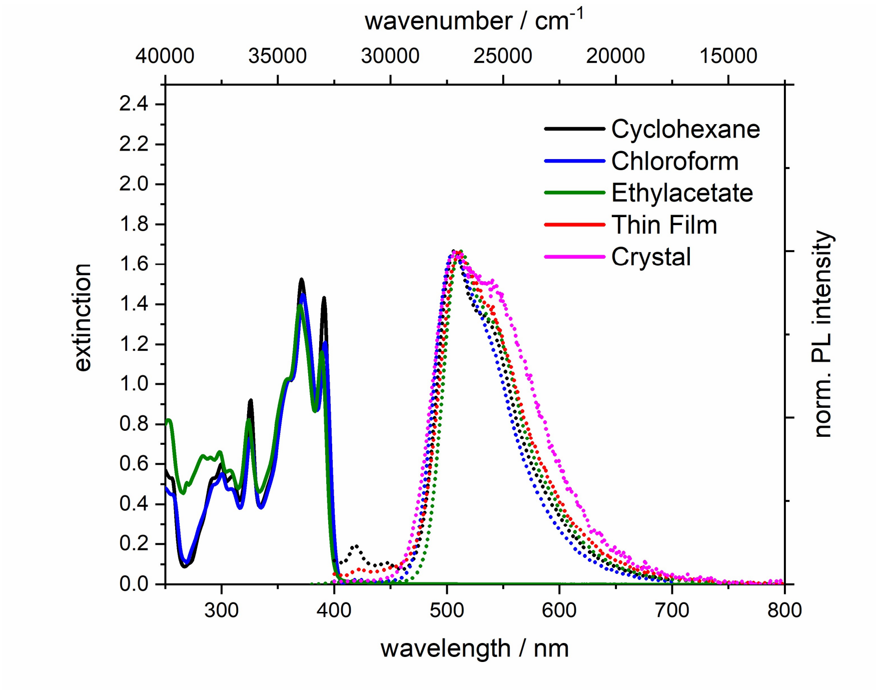

Figure S18: Optical Properties of compound **NO-OH** in different solvents and in solid state, extinction (solid line) and emission (dotted line).

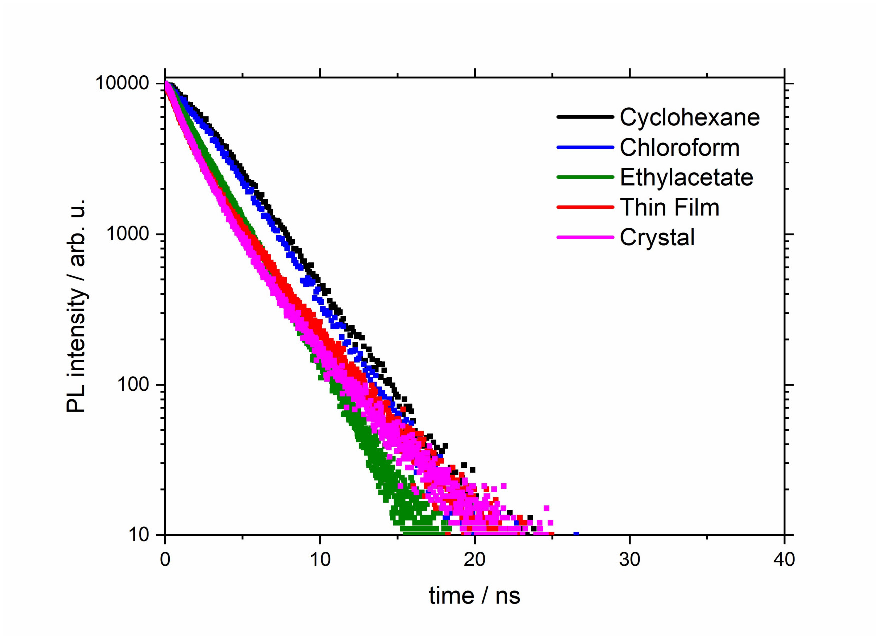

Figure S19: Fluorescence decay of compound **NO-OH** in different solvents and solid state.

3.10 2-(7-Bromo-9,9-dimethyl-9*H*-fluoren-2-yl)benzo[*d*]thiazole (BS)

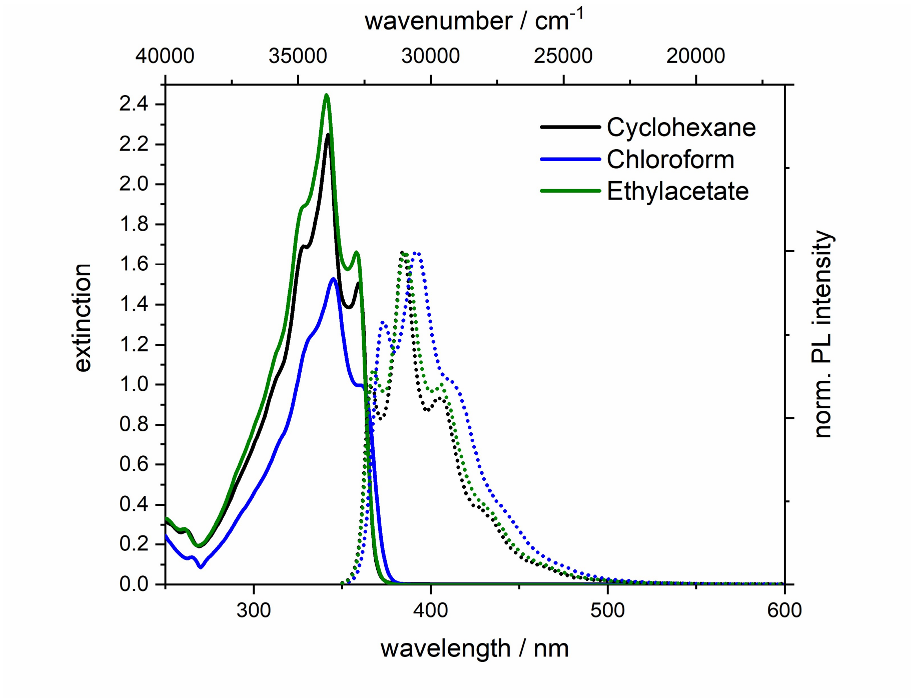

Figure S20: Optical Properties of compound **BS** in different solvents, extinction (solid line) and emission (dotted line).

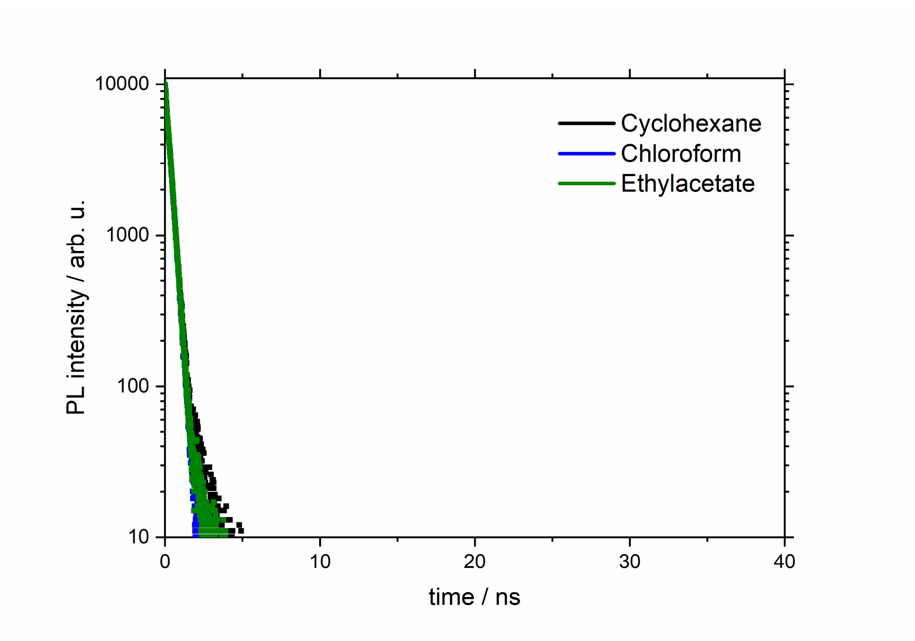

Figure S21: Fluorescence decay of compound **BS** in different solvents.

3.11 2-(Benzo[*d*]thiazol-2-yl)-7-bromo-9,9-dimethyl-9*H*-fluoren-3-ol (BS-OH)

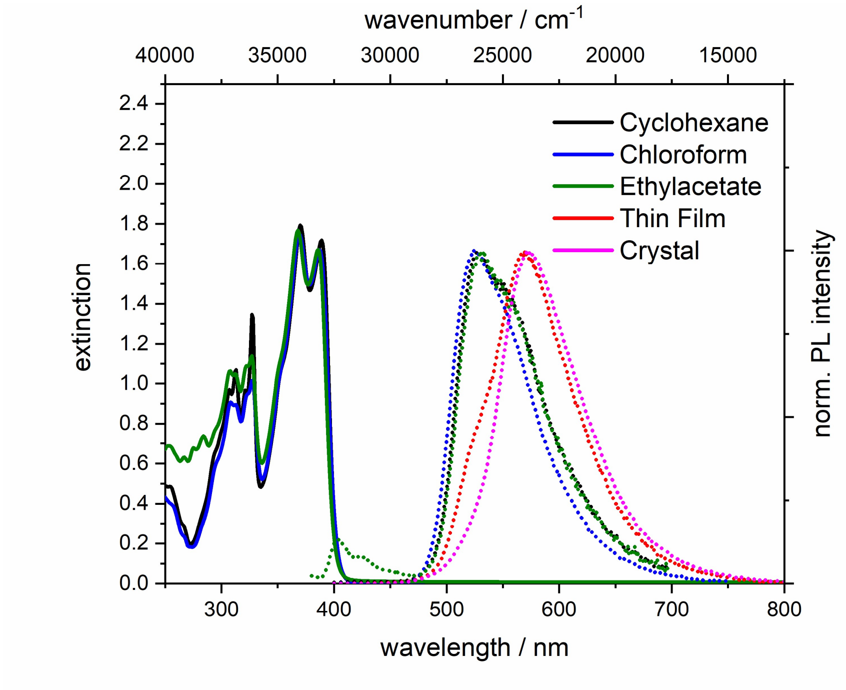

Figure S22: Optical Properties of compound **BS-OH** in different solvents and in solid state, extinction (solid line) and emission (dotted line).

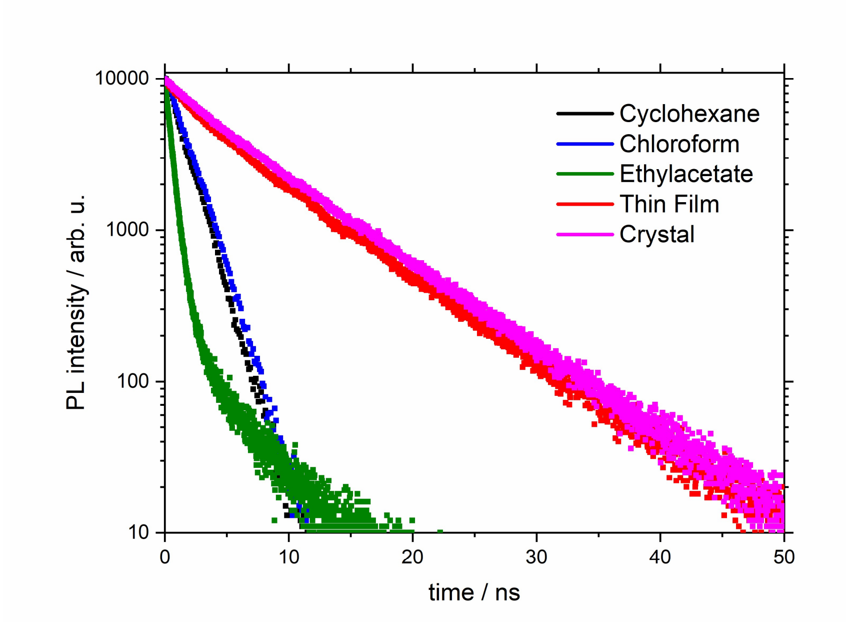

Figure S23: Fluorescence decay of compound **BS-OH** in different solvents and solid state.

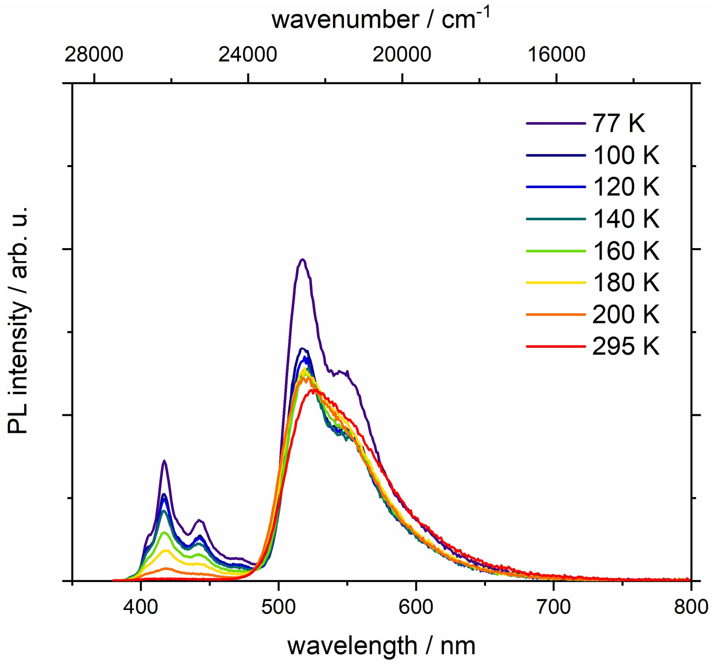

Figure S24: Temperature-dependent emission spectra of compound **BS-OH** in 2-methyltetrahydrofuran.

3.12 2-(7-Bromo-9,9-dimethyl-9*H*-fluoren-2-yl)benzo[*d*][1,3]selenazole (BSe)

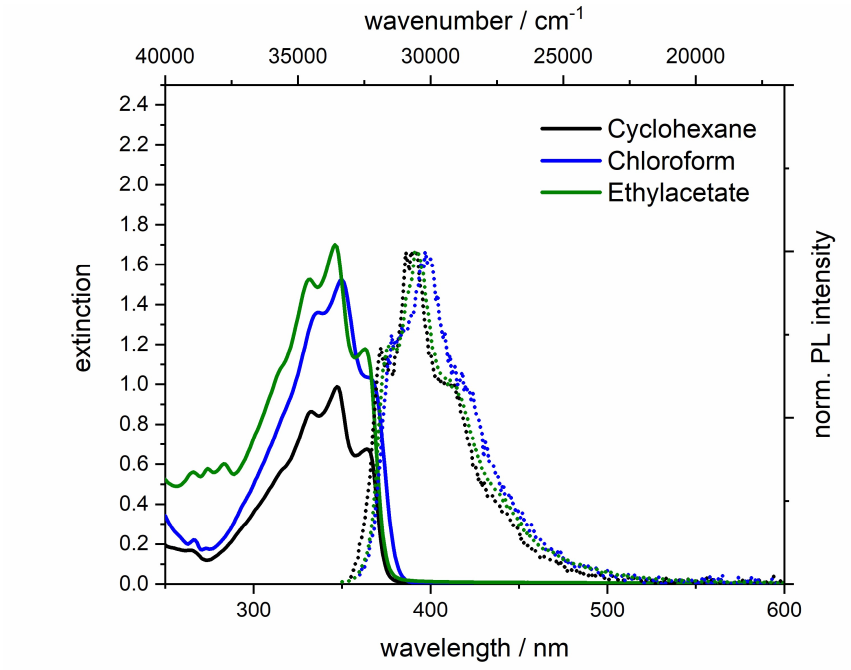

Figure S25: Optical Properties of compound **BSe** in different solvents, extinction (solid line) and emission (dotted line).

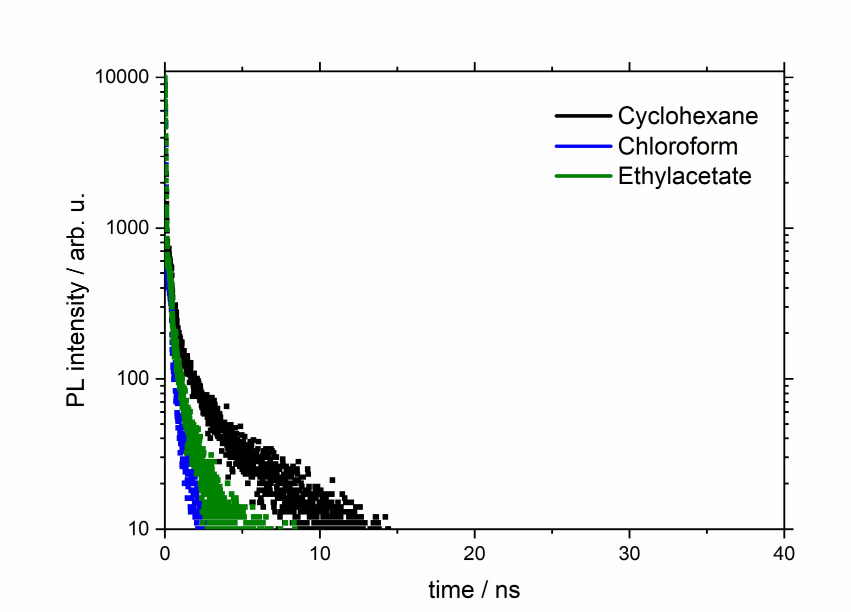

Figure S26: Fluorescence decay of compound **BSe** in different solvents.

3.13 2-(Benzo[*d*][1,3]selenazol-2-yl)-7-bromo-9,9-dimethyl-9*H*-fluoren-3-ol (BSe-OH)

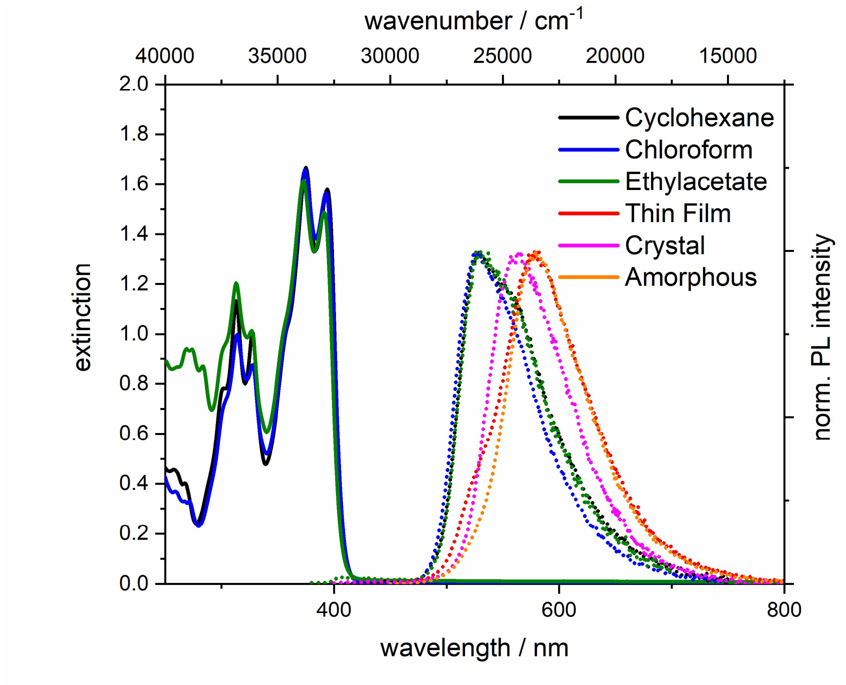

Figure S27: Optical Properties of compound **BSe-OH** in different solvents and in solid state, extinction (solid line) and emission (dotted line).

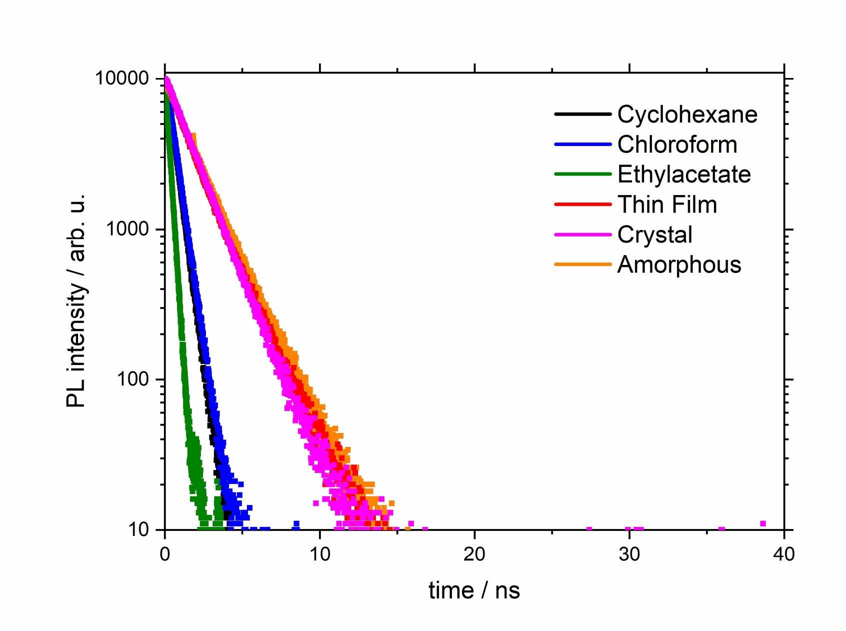

Figure S28: Fluorescence decay of compound **BSe-OH** in different solvents and solid state.

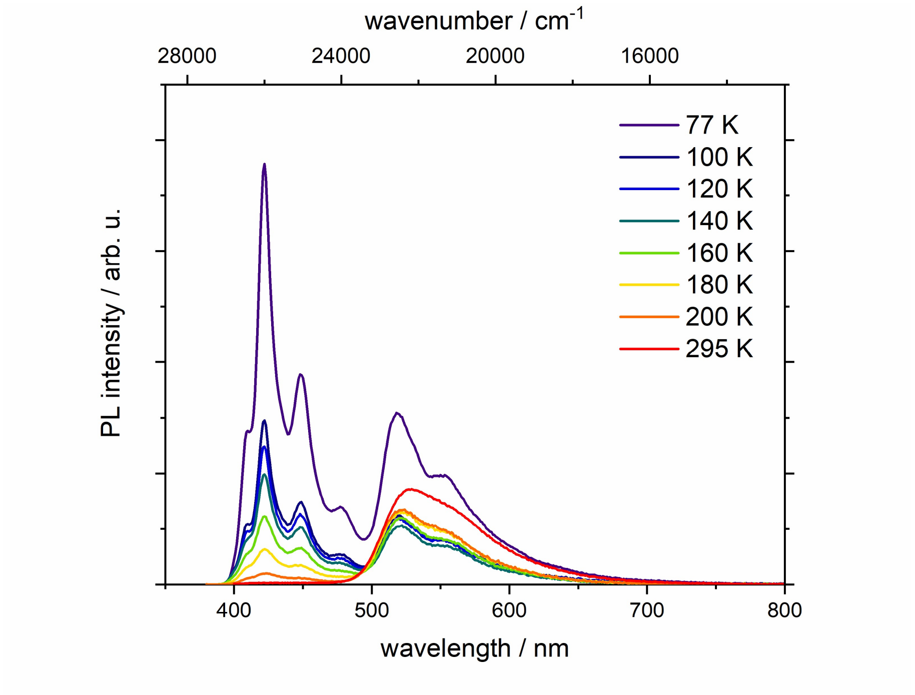

Figure S29: Temperature-dependent emission spectra of compound **BSe-OH** in 2-methyltetrahydrofuran.

3.14 Solid-State Spectra of Hydroxyfluorenes

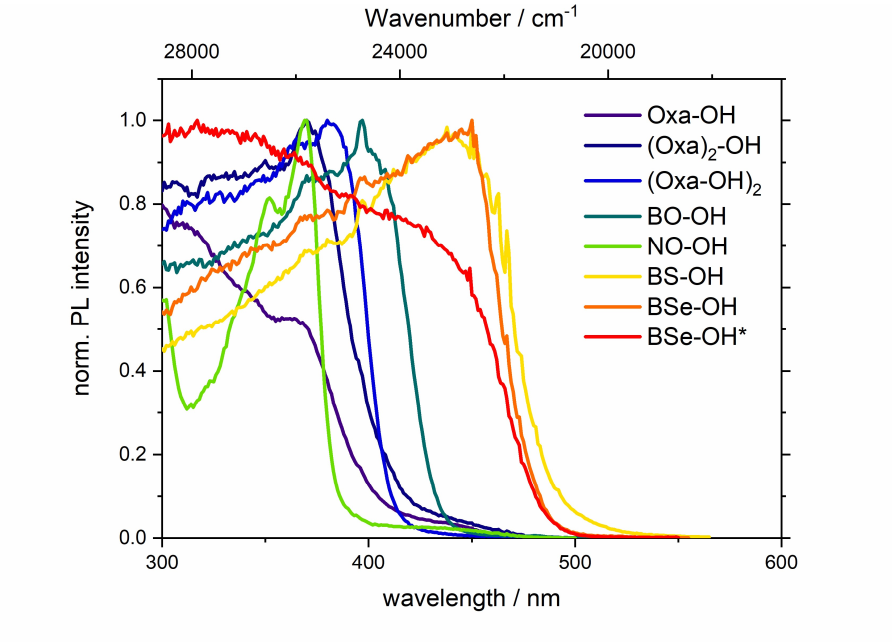

Figure S30: Excitation spectra of the crystalline oxazolinyl- and arylchalcogenazolyl-substituted hydroxyfluorenes.

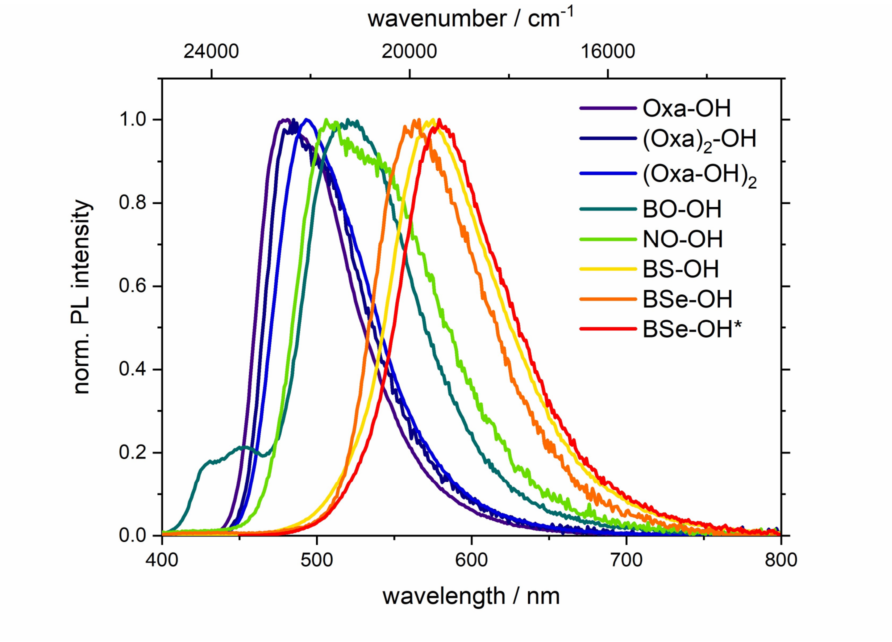

Figure S31: Emission spectra of the crystalline oxazolinyl- and arylchalcogenazolyl-substituted hydroxyfluorenes. The amorphous sample is marked with an asterisk.

3.15 Photophysical Data of non-hydroxylated Fluorenes

Table S2: Photophysical data of **Oxa**, **$(Oxa)_2$**, **BO**, **NO**, **BS** and **BSe** measured in aerated solution and thin film at 295 K.

Compd	Solvent	λ_{abs} [nm] (ε [mol L^{-1} cm^{-1}])	λ_{em} [nm]	$\Delta\tilde{\nu}$ [cm^{-1}]	Φ_F [%][a]	τ [ns] (Rel %)[b]	k_r [10^8 s^{-1}][c]	k_{nr} [10^8 s^{-1}][c]
**Oxa**	CH	320 (47,830)	339	1,750	18	0.2 (85), 6.9 (15)	1.50	6.80
	$CHCl_3$	322 (40,860)	343	1,900	<1	0.8 (47), 4.3 (53)	0.02	3.73
	EtOAc	320 (41,820)	338	1,660	<1	0.7 (85), 3.9 (15)	0.03	8.46
	Thin film	–	346	–	5	0.6 (93), 3.3 (7)	0.63	12.0
**$(Oxa)_2$**	CH	330 (70,120)	351	1,810	30	0.4	7.55	17.5
	$CHCl_3$	333 (79,970)	355	1,860	24	0.8 (59), 4.3 (41)	1.07	3.40
	EtOAc	329 (63,860)	352	1,990	28	0.7 (55), 3.9 (45)	1.31	3.36
	Thin film	–	357	–	2	0.8 (58), 4.3 (42)	0.10	4.30
**BO**	CH	334 (58,820)	373	3,130	32	0.6 (57), 4.0 (43)	1.54	3.31
	$CHCl_3$	337 (53,410)	379	3,290	18	0.5 (80), 2.0 (20)	2.29	2.41
	EtOAc	332 (58,660)	372	3,380	30	2.9	1.03	10.2
	Thin film	–	415	–	31	1.6 (40), 4.2 (60)	0.98	2.18
**NO**	CH	345 (51,420)	407	4,420	72	1.7	4.24	1.64
	$CHCl_3$	348 (55,620)	419	4,870	56	2.5	2.22	1.78
	EtOAc	345 (51,650)	417	5,010	56	2.9	1.92	1.53
	Thin film	–	433	–	13	0.5 (84), 1.8 (16)	1.79	12.3
**BS**	CH	342 (65,760)	384	3,200	20	0.3 (95), 1.5 (5)	5.50	22.3
	$CHCl_3$	345 (43,790)	392	3,470	15	0.3	5.02	28.3
	EtOAc	341 (65,040)	386	3,420	18	0.3	6.15	27.2
	Thin film	–	436	–	13	0.7 (41), 1.6 (59)	1.02	7.10
**BSe**	CH	347 (49,510)	390	3,180	<1	<0.2	–	–
	$CHCl_3$	350 (45,640)	397	3,380	<1	<0.2	–	–
	EtOAc	346 (51,350)	391	3,330	<1	<0.2	–	–
	Thin film	–	452	–	4	0.5	0.82	19.2

[a] Absolute quantum yields were determined by using an integration sphere. [b] Relative ratio of the species of a double exponential function are given in parentheses. [c] k_r (10^8 s^{-1}) and k_{nr} (10^8 s^{-1}) were calculated using the equations $k_r = \Phi_F/\tau$ and $k_{nr} = (1 - \Phi_F)/\tau$. [d] Thin film was prepared *via* drop-cast method of a dichloromethane solution ($c = 10^{-5}$ mol L^{-1}) on a glass slide.

3.16 Photophysical Data of hydroxylated Fluorenes

Table S3: Photophysical data of **Oxa-OH**, **(Oxa)$_2$-OH**, **(Oxa-OH)$_2$**, **BO-OH**, **NO-OH**, **BS-OH**, **BSe-OH** measured in aerated solution and solid-state at 295 K.

Compd	Solvent	λ_{abs} [nm] (ε [mol L^{-1} cm^{-1}])	λ_{em} [nm]	$\Delta\tilde{\nu}$ [cm^{-1}]	Φ_F [%][a]	τ [ns] (Rel %)[b]	k_r [10^8 s^{-1}][c]	k_{nr} [10^8 s^{-1}][c]
**Oxa-OH**	CH	346 (26,349)	480	7,820	53	2.7	1.96	1.74
	$CHCl_3$	347 (17,127)	475	7,770	17	2.6	0.64	3.21
	EtOAc	346 (22,049)	475	7,850	10	1.5 (41), 6.8 (59)	0.22	1.95
	Thin film	–	482	–	25	1.9 (32), 4.3 (68)	0.69	2.14
	Solid	364[d]	481	6,680	38	2.9	1.32	2.13
**(Oxa)$_2$-OH**	CH	357 (20,540)	485	7,370	8	1.6	0.52	5.73
	$CHCl_3$	355 (19,830)	485	7,470	10	1.7	0.61	5.27
	EtOAc	354 (20,630)	483	7,630	42	1.8 (50), 4.9 (50)	1.24	1.75
	Thin film	–	487	–	12	1.9 (68), 3.9 (32)	0.46	3.47
	Solid	370[d]	485	6,410	11	2.1	0.54	4.22
**(Oxa-OH)$_2$**	CH	369 (57,970)	488	6,610	10	2.2	0.47	4.07
	$CHCl_3$	367 (49,640)	488	6,760	11	1.6 (85), 4.8 (15)	0.54	4.27
	EtOAc	367 (52,590)	491	6,880	14	2.3	0.61	3.74
	Thin film	–	491	–	15	1.8 (80), 3.2 (20)	0.71	4.10
	Solid	380[d]	493	6,030	18	2.3	0.77	3.57
**BO-OH**	CH	375 (42,310)	496	6,510	31	2.9	1.06	2.39
	$CHCl_3$	374 (41,990)	494	6,500	34	2.8	1.21	2.36
	EtOAc	372 (40,950)	500	6,882	30	2.3	1.33	3.02
	Thin film	–	509	–	32	1.8 (21), 4.7 (79)	0.79	1.65
	Solid	397[d]	520	5,960	37	1.6 (28), 4.3 (72)	1.04	1.78
**NO-OH**	CH	371 (69,550)	506	7,190	38	2.8	1.34	2.23
	$CHCl_3$	372 (65,820)	507	7,160	38	2.6	1.46	2.39
	EtOAc	370 (63,590)	512	7,500	13	2.4	0.56	3.61
	Thin film	–	509	–	10	1.2 (31), 3.3 (69)	0.37	3.41
	Solid	369[d]	506	7,340	3	1.6 (62), 3.8 (38)	0.11	3.99
**BS-OH**	CH	370 (41,140)	528	8,090	12	1.3	0.91	6.78
	$CHCl_3$	369 (40,190)	530	7,980	16	1.2	1.36	6.97
	EtOAc	368 (40,670)	523	8,310	3	0.5 (83), 4.3 (17)	0.29	8.44
	Thin film	–	569	–	33	2.6 (14), 7.7 (86)	0.47	0.96
	Solid	450[d]	575	4,830	39	7.3	0.54	0.83
**BSe-OH**	CH	375 (47,730)	528	7,730	5	0.6	0.87	15.8
	$CHCl_3$	374 (46,510)	526	7,730	6	0.6	0.99	15.7
	EtOAc	373 (45,410)	530	7,980	2	0.3	0.68	32.7
	Thin film	–	580	–	9	1.3 (50), 2.1 (50)	0.51	5.37
	Solid	450[d]	566	4,550	7	1.7	0.41	5.47
	Amorphous	435[d]	579	5,720	7	1.8	0.39	5.04

[a] Absolute quantum yields were determined by using an integration sphere. [b] Relative ratio of the species of a double exponential function are given in parentheses. [c] k_r (10^8 s^{-1}) and k_{nr} (10^8 s^{-1}) were calculated using the equations $k_r = \Phi_F/\tau$ and $k_{nr} = (1 - \Phi_F)/\tau$. [d] Excitation maximum. [f] Thin film was prepared *via* drop-cast method of a dichloromethane solution ($c = 10^{-5}$ mol L^{-1}) on a glass slide.

3.17 Photographs of Luminophores

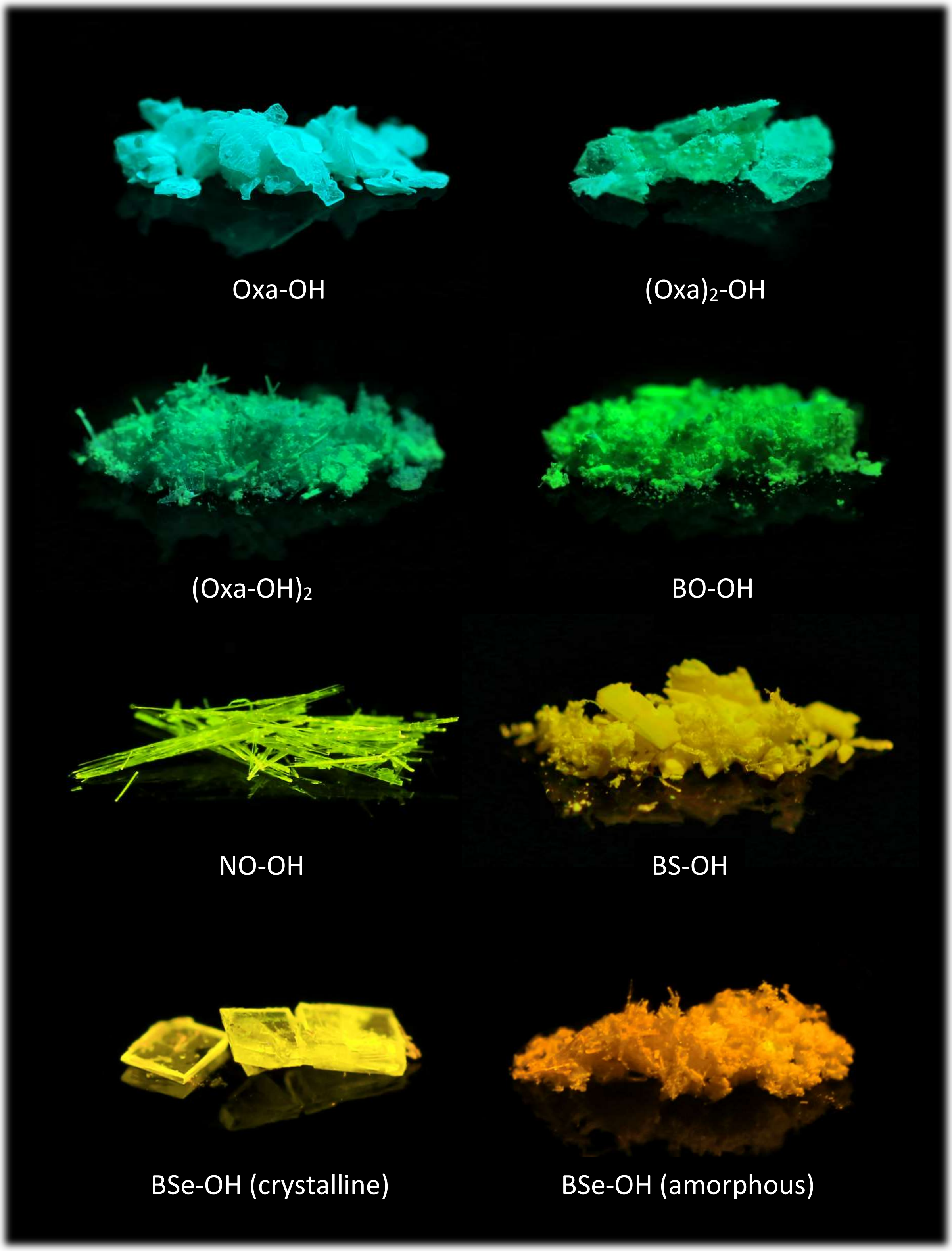

Figure S32: Representative images of hydroxylated fluorenes in the solid-state under 366 nm irradiation.

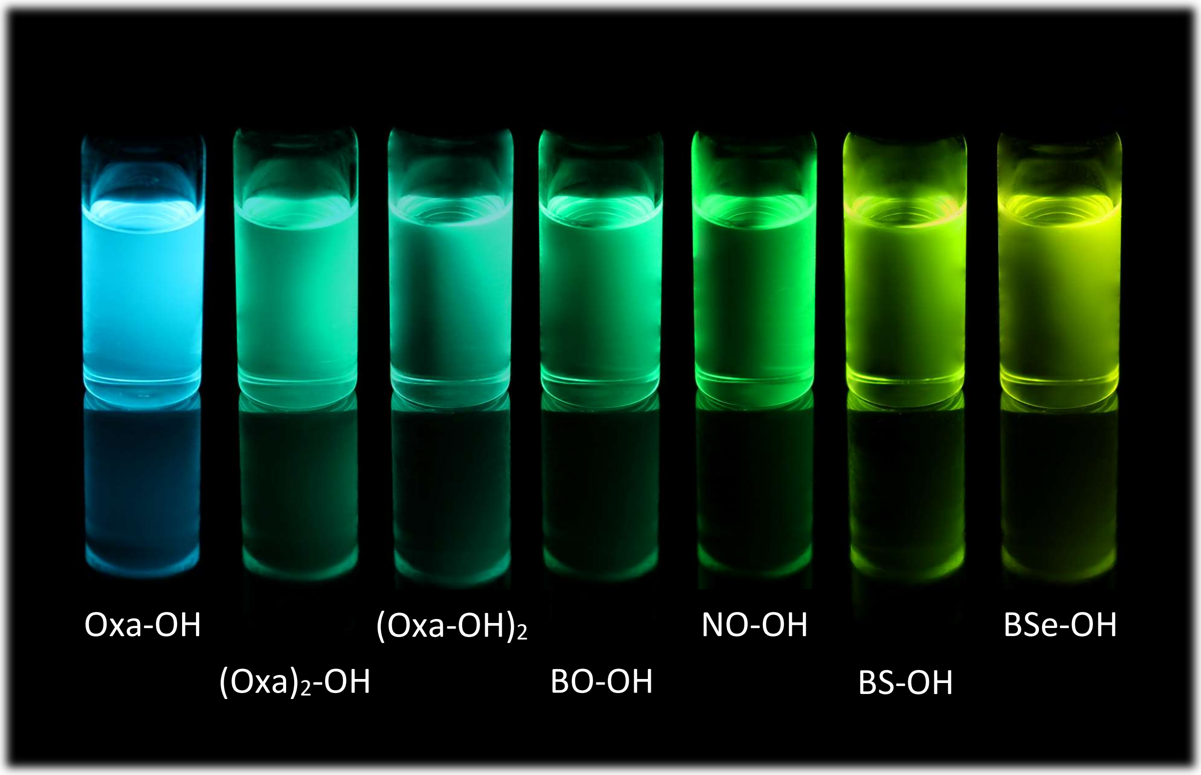

Figure S33: Representative images of hydroxylated fluorenes in dichloromethane solution ($c = 10^{-4}$ mol L^{-1}) under 366 nm irradiation.

4 Crystal Structures

4.1 7-Bromo-2-(4,4-dimethyl-4,5-dihydrooxazol-2-yl)-9,9-dimethyl-9*H*-fluoren-3-ol (Oxa-OH)

4.1.1 Crystal Data for Oxa-OH

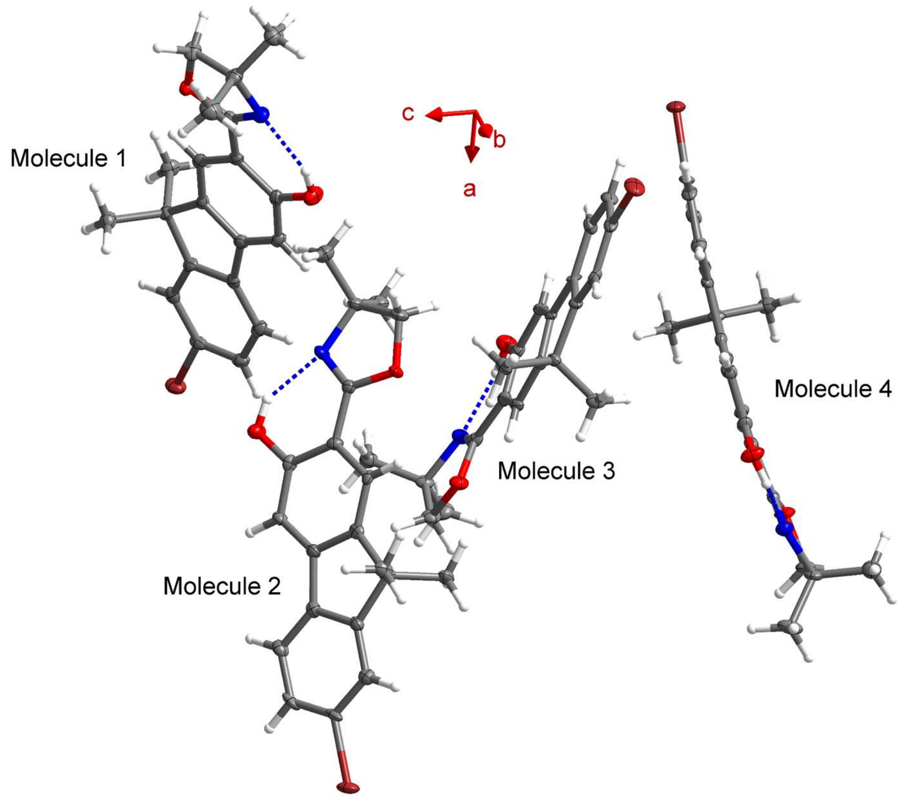

Figure S34: Asymmetric unit of **Oxa-OH**.

Table S4: Crystal data and structure refinement for **Oxa-OH**.

Empirical formula	$C_{20}H_{20}BrNO_2$
Formula weight/g mol^{-1}	386.28
Crystal system	Triclinic
Crystal size/mm^3	0.24 × 0.23 × 0.21
Space group	P–1
a/Å	9.6343(2)
b/Å	19.0413(5)
c/Å	19.4763(5)
α/°	83.018(2)
β/°	89.376(2)

$\gamma/°$	82.190(2)
Volume/Å^3	3513.47(15)
Z	8
$\rho_{calc,}$ g/cm^3	1.461
μ/mm^{-1}	2.351
F(000)	1584.0
2Θ range for data collection/°	4.52 to 50
Index ranges	$-11 \leq h \leq 11$, $-22 \leq k \leq 22$, $-23 \leq l \leq 23$
No. of reflections collected	85212
No. of independent reflections	12379 [R_{int} = 0.0727, R_{sigma} = 0.0411]
Data/restraints/parameters	12379/0/886
Goodness-of-fit on F^2	1.240
Final R indexes [I ≥ 2σ (I)]	R_1 = 0.0698, wR_2 = 0.1465
Final R indexes [all data]	R_1 = 0.0815, wR_2 = 0.1510
Largest diff. peak/hole / e Å^{-3}	1.71/–1.04
CCDC number	2018044

Table S5: Fractional Atomic Coordinates (×10^4) and Equivalent Isotropic Displacement Parameters (Å^2×10^3) for **Oxa-OH**. U_{eq} is defined as 1/3 of the trace of the orthogonalized U_{IJ} tensor.

Atom	*x*	*y*	*z*	U(eq)
O4	2876(4)	7174(2)	6567(2)	20.0(9)
N1	-1056(5)	3798(3)	7698(3)	19.1(11)
O1	1126(5)	4002(2)	6923(2)	24.8(10)
C1	1729(6)	2272(3)	8010(3)	15.4(12)
O2	-928(4)	2787(2)	8446(2)	20.6(9)
C2	1042(6)	2926(3)	7713(3)	14.8(12)
C3	1765(6)	3369(3)	7238(3)	16.3(12)
Br1	9611.7(6)	755.3(4)	7577.6(3)	24.51(17)
C4	3168(6)	3166(3)	7087(3)	15.8(12)
C4A	3831(6)	2516(3)	7401(3)	16.3(12)

C4B	5290(6)	2171(3)	7356(3)	15.9(12)
C5	6434(6)	2408(3)	7004(3)	18.6(13)
C6	7728(6)	1983(3)	7085(3)	19.0(13)
C7	7839(6)	1328(4)	7494(3)	19.1(13)
C8	6709(6)	1085(3)	7842(3)	17.6(13)
C8A	5418(6)	1521(3)	7770(3)	15.3(12)
C9	4052(6)	1380(3)	8128(3)	17.5(13)
C9A	3109(6)	2060(3)	7851(3)	13.9(12)
C10	4213(7)	1313(4)	8924(3)	22.6(14)
C11	3528(7)	709(3)	7912(3)	21.3(14)
C12	-372(6)	3193(3)	7927(3)	17.1(13)
C13	-2326(6)	3162(3)	8543(3)	22.8(14)
C14	-2298(7)	3909(3)	8159(3)	21.5(14)
C15	-2006(7)	4445(4)	8630(4)	29.5(16)
C16	-3644(7)	4172(4)	7738(4)	27.4(15)
N2	3263(5)	6162(3)	7306(3)	17.2(11)
N3	6047(5)	6051(3)	5202(3)	18.5(11)
N4	11635(5)	3928(3)	-193(3)	19.6(11)
C68A	6752(6)	1463(3)	458(3)	14.8(12)
C69	8258(6)	1334(3)	189(3)	14.8(12)
C32	3639(6)	6765(3)	7081(3)	14.5(12)
C24A	7448(6)	7444(3)	7598(3)	15.9(12)
C21	5232(6)	7696(3)	6998(3)	14.0(12)
C66	4104(6)	1935(3)	928(3)	18.0(13)
C49A	2159(6)	7927(3)	4778(3)	14.1(12)
C29	7063(6)	8596(3)	6892(3)	14.6(12)
C68	5772(6)	990(3)	528(3)	15.5(12)
C48A	-86(6)	8538(3)	4548(3)	14.9(12)
C27	10831(6)	8634(4)	7492(3)	19.5(13)
C64B	6425(6)	2158(3)	632(3)	14.1(12)
C28	9567(6)	8884(3)	7156(3)	17.7(13)
C47	-2483(6)	8788(4)	4231(3)	19.6(13)

C25	9986(6)	7559(3)	7981(3)	18.8(13)
Br4	3029.1(6)	645.3(4)	806.3(3)	22.62(17)
C22	4908(6)	7030(3)	7294(3)	15.2(12)
C23	5845(6)	6582(3)	7761(3)	15.1(12)
C31	7140(7)	8707(3)	6101(3)	21.8(14)
C69A	8716(6)	2055(3)	238(3)	16.1(12)
C64	7827(6)	3228(3)	563(3)	16.8(13)
C52	5489(6)	6695(3)	5208(3)	16.0(12)
C44B	-59(6)	7843(3)	4378(3)	15.3(12)
C67	4449(6)	1253(3)	758(3)	18.6(13)
C49	1333(6)	8658(3)	4819(3)	14.6(12)
C24	7141(6)	6792(3)	7902(3)	16.1(12)
C44	1893(6)	6759(3)	4457(3)	15.3(12)
C61	9979(6)	2293(3)	30(3)	14.3(12)
C28A	8517(6)	8454(3)	7223(3)	15.9(12)
C64A	7647(6)	2536(3)	494(3)	15.8(12)
C48	-1302(6)	9022(3)	4468(3)	16.5(13)
C65	5104(6)	2395(3)	878(3)	18.3(13)
C43	3275(6)	6518(3)	4659(3)	16.0(12)
C26	11050(6)	7979(4)	7900(3)	21.5(14)
C29A	6491(6)	7904(3)	7162(3)	15.0(12)
Br2	12304.7(6)	9202.5(4)	7384.5(4)	24.82(17)
C53	7512(6)	6727(3)	5743(3)	20.6(14)
C46	-2498(6)	8111(4)	4064(3)	22.8(14)
C54	7347(6)	5951(3)	5623(3)	17.8(13)
C76	12383(7)	4490(4)	-1291(3)	25.0(15)
C45	-1253(6)	7619(3)	4139(3)	20.1(13)
C55	7082(7)	5497(3)	6297(3)	21.1(14)
C73	13467(6)	3264(3)	-751(3)	21.6(14)
C75	13923(7)	4391(4)	-241(4)	31.3(17)
C35	884(7)	5782(4)	7273(4)	26.4(15)
C36	2685(7)	5513(4)	6365(4)	25.7(15)

C56	8566(7)	5600(4)	5230(4)	29.7(16)
Br3	-4175.0(7)	9434.2(4)	4153.2(4)	29.23(18)
O3	5542(4)	5948(2)	8065(2)	21.6(10)
O8	12353(4)	2843(2)	-527(2)	19.1(9)
O7	9246(5)	4148(2)	427(2)	23.8(10)
O5	3786(5)	5836(2)	4590(2)	23.2(10)
O6	6181(4)	7141(2)	5524(2)	19.1(9)
C44A	1352(6)	7454(3)	4529(3)	14.5(12)
C72	11407(6)	3286(3)	-202(3)	14.8(12)
C62	10167(6)	2996(3)	82(3)	14.7(12)
C70	9133(6)	726(3)	642(3)	20.7(14)
C63	9099(6)	3472(3)	356(3)	16.9(13)
C51	1937(6)	9261(3)	4372(3)	19.7(13)
C24B	8722(6)	7784(3)	7638(3)	15.3(12)
C41	3535(6)	7690(3)	4980(3)	16.0(12)
C30	6209(6)	9235(3)	7167(3)	20.5(13)
C74	12897(6)	4035(3)	-630(3)	19.6(13)
C33	1685(6)	6804(3)	6467(3)	22.4(14)
C34	2090(6)	6052(3)	6851(3)	18.2(13)
C71	8259(7)	1171(3)	-563(3)	22.6(14)
C50	1212(6)	8821(3)	5574(3)	18.9(13)
C42	4087(6)	6987(3)	4933(3)	15.6(12)

Table S6: Bond Lengths for **Oxa-OH**.

Atom	Atom	Length/Å	Atom	Atom	Length/Å
O4	C32	1.349(7)	C29	C29A	1.529(8)
O4	C33	1.452(7)	C29	C30	1.526(8)
N1	C12	1.280(8)	C68	C67	1.395(8)
N1	C14	1.495(8)	C48A	C44B	1.398(9)
O1	C3	1.355(7)	C48A	C49	1.527(8)
C1	C2	1.390(8)	C48A	C48	1.387(8)
C1	C9A	1.380(8)	C27	C28	1.386(8)

O2	C12	1.348(7)	C27	C26	1.386(9)
O2	C13	1.457(7)	C27	Br2	1.895(6)
C2	C3	1.417(8)	C64B	C64A	1.468(8)
C2	C12	1.462(8)	C64B	C65	1.391(8)
C3	C4	1.393(8)	C28	C28A	1.381(8)
Br1	C7	1.894(6)	C47	C48	1.381(9)
C4	C4A	1.388(8)	C47	C46	1.370(10)
C4A	C4B	1.474(8)	C47	Br3	1.899(6)
C4A	C9A	1.403(8)	C25	C26	1.380(9)
C4B	C5	1.391(8)	C25	C24B	1.385(8)
C4B	C8A	1.384(8)	Br4	C67	1.903(6)
C5	C6	1.390(9)	C22	C23	1.412(8)
C6	C7	1.389(9)	C23	C24	1.402(8)
C7	C8	1.379(9)	C23	O3	1.346(7)
C8	C8A	1.397(8)	C69A	C61	1.394(8)
C8A	C9	1.524(8)	C69A	C64A	1.411(8)
C9	C9A	1.522(8)	C64	C64A	1.374(9)
C9	C10	1.547(8)	C64	C63	1.407(8)
C9	C11	1.540(9)	C52	O6	1.354(7)
C13	C14	1.528(9)	C52	C42	1.470(8)
C14	C15	1.508(10)	C44B	C45	1.384(8)
C14	C16	1.534(9)	C44B	C44A	1.471(8)
N2	C32	1.276(8)	C49	C51	1.530(8)
N2	C34	1.496(7)	C49	C50	1.540(8)
N3	C52	1.270(8)	C44	C43	1.392(8)
N3	C54	1.483(8)	C44	C44A	1.377(8)
N4	C72	1.273(8)	C61	C62	1.390(8)
N4	C74	1.498(8)	C28A	C24B	1.415(8)
C68A	C69	1.535(8)	C43	O5	1.348(7)
C68A	C68	1.387(8)	C43	C42	1.417(8)
C68A	C64B	1.399(8)	C53	C54	1.553(9)
C69	C69A	1.513(8)	C53	O6	1.451(7)

C69	C70	1.527(8)	C46	C45	1.415(9)
C69	C71	1.533(8)	C54	C55	1.520(9)
C32	C22	1.467(8)	C54	C56	1.518(9)
C24A	C24	1.378(8)	C76	C74	1.510(9)
C24A	C29A	1.398(8)	C73	O8	1.458(7)
C24A	C24B	1.471(8)	C73	C74	1.540(9)
C21	C22	1.402(8)	C75	C74	1.528(9)
C21	C29A	1.378(8)	C35	C34	1.525(8)
C66	C67	1.375(9)	C36	C34	1.529(9)
C66	C65	1.383(9)	O8	C72	1.361(7)
C49A	C49	1.517(8)	O7	C63	1.338(7)
C49A	C44A	1.399(8)	C72	C62	1.459(8)
C49A	C41	1.386(8)	C62	C63	1.420(8)
C29	C31	1.530(8)	C41	C42	1.386(8)
C29	C28A	1.525(8)	C33	C34	1.535(9)

Table S7: Bond Angles for **Oxa-OH**.

Atom	Atom	Atom	Angle/°	Atom	Atom	Atom	Angle/°
C32	O4	C33	105.4(4)	C26	C25	C24B	119.7(6)
C12	N1	C14	106.4(5)	C21	C22	C32	120.3(5)
C9A	C1	C2	120.2(5)	C21	C22	C23	120.7(5)
C12	O2	C13	104.9(5)	C23	C22	C32	118.9(5)
C1	C2	C3	119.5(5)	C24	C23	C22	119.4(5)
C1	C2	C12	121.1(5)	O3	C23	C22	121.8(5)
C3	C2	C12	119.2(5)	O3	C23	C24	118.8(5)
O1	C3	C2	121.5(5)	C61	C69A	C69	128.5(5)
O1	C3	C4	118.0(5)	C61	C69A	C64A	119.1(5)
C4	C3	C2	120.6(5)	C64A	C69A	C69	112.3(5)
C4A	C4	C3	118.5(5)	C64A	C64	C63	119.2(5)
C4	C4A	C4B	130.8(5)	N3	C52	O6	118.8(5)
C4	C4A	C9A	121.3(5)	N3	C52	C42	124.5(5)
C9A	C4A	C4B	107.9(5)	O6	C52	C42	116.6(5)

C5	C4B	C4A	130.8(6)	C48A	C44B	C44A	108.4(5)
C8A	C4B	C4A	108.0(5)	C45	C44B	C48A	120.8(6)
C8A	C4B	C5	121.2(6)	C45	C44B	C44A	130.8(6)
C6	C5	C4B	118.6(6)	C66	C67	C68	123.5(5)
C7	C6	C5	119.7(6)	C66	C67	Br4	118.4(4)
C6	C7	Br1	118.4(4)	C68	C67	Br4	118.0(5)
C8	C7	Br1	119.3(5)	C49A	C49	C48A	100.0(5)
C8	C7	C6	122.3(6)	C49A	C49	C51	113.4(5)
C7	C8	C8A	117.8(6)	C49A	C49	C50	110.7(5)
C4B	C8A	C8	120.5(5)	C48A	C49	C51	111.9(5)
C4B	C8A	C9	112.5(5)	C48A	C49	C50	110.5(5)
C8	C8A	C9	127.0(5)	C51	C49	C50	110.0(5)
C8A	C9	C10	111.0(5)	C24A	C24	C23	119.0(5)
C8A	C9	C11	111.7(5)	C44A	C44	C43	118.7(5)
C9A	C9	C8A	100.0(5)	C62	C61	C69A	119.6(5)
C9A	C9	C10	111.5(5)	C28	C28A	C29	128.2(5)
C9A	C9	C11	112.0(5)	C28	C28A	C24B	120.6(5)
C11	C9	C10	110.4(5)	C24B	C28A	C29	111.2(5)
C1	C9A	C4A	119.8(5)	C69A	C64A	C64B	107.4(5)
C1	C9A	C9	128.5(5)	C64	C64A	C64B	130.6(6)
C4A	C9A	C9	111.7(5)	C64	C64A	C69A	122.0(6)
N1	C12	O2	118.5(5)	C47	C48	C48A	117.6(6)
N1	C12	C2	125.0(6)	C66	C65	C64B	118.5(6)
O2	C12	C2	116.2(5)	C44	C43	C42	119.7(5)
O2	C13	C14	104.5(5)	O5	C43	C44	118.0(5)
N1	C14	C13	102.3(5)	O5	C43	C42	122.3(5)
N1	C14	C15	107.3(5)	C25	C26	C27	119.7(6)
N1	C14	C16	111.3(5)	C24A	C29A	C29	112.0(5)
C13	C14	C16	111.7(5)	C21	C29A	C24A	120.2(6)
C15	C14	C13	112.9(6)	C21	C29A	C29	127.7(5)
C15	C14	C16	110.9(5)	O6	C53	C54	104.4(5)
C32	N2	C34	107.1(5)	C47	C46	C45	119.2(6)

C52	N3	C54	107.2(5)	N3	C54	C53	102.6(5)
C72	N4	C74	107.7(5)	N3	C54	C55	107.5(5)
C68	C68A	C69	127.8(5)	N3	C54	C56	110.2(5)
C68	C68A	C64B	121.0(5)	C55	C54	C53	111.5(5)
C64B	C68A	C69	111.2(5)	C56	C54	C53	112.8(5)
C69A	C69	C68A	100.4(5)	C56	C54	C55	111.6(5)
C69A	C69	C70	113.1(5)	C44B	C45	C46	118.5(6)
C69A	C69	C71	111.2(5)	O8	C73	C74	105.2(5)
C70	C69	C68A	111.2(5)	C72	O8	C73	105.3(4)
C70	C69	C71	110.2(5)	C52	O6	C53	105.4(5)
C71	C69	C68A	110.5(5)	C49A	C44A	C44B	107.5(5)
O4	C32	C22	116.4(5)	C44	C44A	C49A	122.0(5)
N2	C32	O4	118.0(5)	C44	C44A	C44B	130.5(5)
N2	C32	C22	125.4(5)	N4	C72	O8	118.0(5)
C24	C24A	C29A	121.6(5)	N4	C72	C62	124.9(5)
C24	C24A	C24B	130.3(5)	O8	C72	C62	117.1(5)
C29A	C24A	C24B	108.1(5)	C61	C62	C72	121.1(5)
C29A	C21	C22	119.0(5)	C61	C62	C63	120.9(5)
C67	C66	C65	119.6(5)	C63	C62	C72	117.8(5)
C44A	C49A	C49	112.5(5)	C64	C63	C62	119.1(5)
C41	C49A	C49	127.9(5)	O7	C63	C64	118.0(5)
C41	C49A	C44A	119.6(6)	O7	C63	C62	122.8(5)
C28A	C29	C31	111.8(5)	C25	C24B	C24A	132.0(6)
C28A	C29	C29A	100.5(5)	C25	C24B	C28A	119.8(6)
C28A	C29	C30	110.0(5)	C28A	C24B	C24A	108.3(5)
C29A	C29	C31	112.3(5)	C42	C41	C49A	119.3(5)
C30	C29	C31	111.1(5)	N4	C74	C76	107.1(5)
C30	C29	C29A	110.8(5)	N4	C74	C73	102.0(5)
C68A	C68	C67	116.3(5)	N4	C74	C75	110.0(5)
C44B	C48A	C49	111.6(5)	C76	C74	C73	112.2(5)
C48	C48A	C44B	120.8(5)	C76	C74	C75	111.7(6)
C48	C48A	C49	127.6(5)	C75	C74	C73	113.2(5)

C28	C27	Br2	118.9(5)	O4	C33	C34	104.6(5)
C26	C27	C28	122.0(6)	N2	C34	C35	111.6(5)
C26	C27	Br2	119.1(4)	N2	C34	C36	107.5(5)
C68A	C64B	C64A	108.8(5)	N2	C34	C33	101.8(5)
C65	C64B	C68A	121.0(5)	C35	C34	C36	110.6(5)
C65	C64B	C64A	130.1(6)	C35	C34	C33	112.6(5)
C28A	C28	C27	118.2(6)	C36	C34	C33	112.3(5)
C48	C47	Br3	118.1(5)	C43	C42	C52	118.3(5)
C46	C47	C48	123.1(6)	C41	C42	C52	120.9(5)
C46	C47	Br3	118.7(5)	C41	C42	C43	120.7(5)

Table S8: Torsion Angles for **Oxa-OH**.

A	B	C	D	Angle/°	A	B	C	D	Angle/°
O4	C32	C22	C21	3.8(8)	C31	C29	C29A	C24A	120.6(6)
O4	C32	C22	C23	-171.5(5)	C31	C29	C29A	C21	-56.4(8)
O4	C33	C34	N2	17.3(6)	C69A	C61	C62	C72	-173.0(5)
O4	C33	C34	C35	136.9(5)	C69A	C61	C62	C63	1.1(9)
O4	C33	C34	C36	-97.4(6)	C52	N3	C54	C53	-10.3(6)
O1	C3	C4	C4A	-179.4(5)	C52	N3	C54	C55	107.4(6)
C1	C2	C3	O1	178.3(5)	C52	N3	C54	C56	-130.7(6)
C1	C2	C3	C4	-2.5(9)	C44B	C48A	C49	C49A	-0.1(6)
C1	C2	C12	N1	177.4(6)	C44B	C48A	C49	C51	120.2(6)
C1	C2	C12	O2	3.2(8)	C44B	C48A	C49	C50	-116.8(6)
O2	C13	C14	N1	17.9(6)	C44B	C48A	C48	C47	1.5(9)
O2	C13	C14	C15	-97.0(6)	C67	C66	C65	C64B	2.1(9)
O2	C13	C14	C16	137.1(5)	C49	C49A	C44A	C44B	0.9(7)
C2	C1	C9A	C4A	1.6(9)	C49	C49A	C44A	C44	-179.3(5)
C2	C1	C9A	C9	178.6(6)	C49	C49A	C41	C42	176.8(5)
C2	C3	C4	C4A	1.4(9)	C49	C48A	C44B	C45	178.3(5)
C3	C2	C12	N1	2.7(9)	C49	C48A	C44B	C44A	0.6(7)
C3	C2	C12	O2	-171.5(5)	C49	C48A	C48	C47	-178.1(6)
C3	C4	C4A	C4B	-178.4(6)	C24	C24A	C29A	C21	-3.1(9)

C3	C4	C4A	C9A	1.2(9)	C24	C24A	C29A	C29	179.7(5)
Br1	C7	C8	C8A	179.9(4)	C24	C24A	C24B	C25	0.5(11)
C4	C4A	C4B	C5	2.3(11)	C24	C24A	C24B	C28A	179.2(6)
C4	C4A	C4B	C8A	-179.9(6)	C44	C43	C42	C52	173.6(5)
C4	C4A	C9A	C1	-2.7(9)	C44	C43	C42	C41	-2.3(9)
C4	C4A	C9A	C9	179.8(5)	C61	C69A	C64A	C64B	176.7(5)
C4A	C4B	C5	C6	176.4(6)	C61	C69A	C64A	C64	-2.0(9)
C4A	C4B	C8A	C8	-178.2(5)	C61	C62	C63	C64	-1.2(9)
C4A	C4B	C8A	C9	-0.2(7)	C61	C62	C63	O7	178.7(6)
C4B	C4A	C9A	C1	177.0(5)	C28A	C29	C29A	C24A	1.7(6)
C4B	C4A	C9A	C9	-0.5(7)	C28A	C29	C29A	C21	-175.3(6)
C4B	C5	C6	C7	2.0(9)	C64A	C64B	C65	C66	176.6(6)
C4B	C8A	C9	C9A	-0.1(6)	C64A	C69A	C61	C62	0.5(9)
C4B	C8A	C9	C10	-117.8(6)	C64A	C64	C63	O7	179.8(5)
C4B	C8A	C9	C11	118.5(6)	C64A	C64	C63	C62	-0.2(9)
C5	C4B	C8A	C8	-0.1(9)	C48	C48A	C44B	C45	-1.3(9)
C5	C4B	C8A	C9	177.9(5)	C48	C48A	C44B	C44A	-179.0(5)
C5	C6	C7	Br1	178.8(5)	C48	C48A	C49	C49A	179.4(6)
C5	C6	C7	C8	-1.6(9)	C48	C48A	C49	C51	-60.2(8)
C6	C7	C8	C8A	0.3(9)	C48	C48A	C49	C50	62.7(8)
C7	C8	C8A	C4B	0.6(9)	C48	C47	C46	C45	0.4(9)
C7	C8	C8A	C9	-177.1(6)	C65	C66	C67	C68	-0.5(9)
C8	C8A	C9	C9A	177.8(6)	C65	C66	C67	Br4	-178.0(5)
C8	C8A	C9	C10	60.0(8)	C65	C64B	C64A	C69A	-178.9(6)
C8	C8A	C9	C11	-63.6(8)	C65	C64B	C64A	C64	-0.4(11)
C8A	C4B	C5	C6	-1.2(9)	C43	C44	C44A	C49A	1.9(9)
C8A	C9	C9A	C1	-176.9(6)	C43	C44	C44A	C44B	-178.3(6)
C8A	C9	C9A	C4A	0.4(6)	C26	C27	C28	C28A	-1.5(9)
C9A	C1	C2	C3	1.0(9)	C26	C25	C24B	C24A	176.7(6)
C9A	C1	C2	C12	-173.7(5)	C26	C25	C24B	C28A	-1.9(9)
C9A	C4A	C4B	C5	-177.4(6)	C29A	C24A	C24	C23	0.8(9)
C9A	C4A	C4B	C8A	0.5(7)	C29A	C24A	C24B	C25	-178.5(6)

C10	C9	C9A	C1	-59.5(8)	C29A	C24A	C24B	C28A	0.2(7)
C10	C9	C9A	C4A	117.7(6)	C29A	C21	C22	C32	-174.3(5)
C11	C9	C9A	C1	64.7(8)	C29A	C21	C22	C23	1.0(9)
C11	C9	C9A	C4A	-118.1(6)	C29A	C29	C28A	C28	178.2(6)
C12	N1	C14	C13	-14.6(6)	C29A	C29	C28A	C24B	-1.5(6)
C12	N1	C14	C15	104.4(6)	Br2	C27	C28	C28A	178.1(4)
C12	N1	C14	C16	-134.0(6)	Br2	C27	C26	C25	-179.9(5)
C12	O2	C13	C14	-15.3(6)	C46	C47	C48	C48A	-1.0(9)
C12	C2	C3	O1	-6.9(9)	C54	N3	C52	O6	4.6(7)
C12	C2	C3	C4	172.3(5)	C54	N3	C52	C42	-171.2(5)
C13	O2	C12	N1	6.5(7)	C54	C53	O6	C52	-10.0(6)
C13	O2	C12	C2	-178.9(5)	C45	C44B	C44A	C49A	-178.3(6)
C14	N1	C12	O2	5.7(7)	C45	C44B	C44A	C44	1.9(11)
C14	N1	C12	C2	-168.4(6)	C73	O8	C72	N4	3.3(7)
N2	C32	C22	C21	178.5(6)	C73	O8	C72	C62	-179.7(5)
N2	C32	C22	C23	3.1(9)	Br3	C47	C48	C48A	177.6(4)
N3	C52	O6	C53	3.9(7)	Br3	C47	C46	C45	-178.2(5)
N3	C52	C42	C43	1.9(9)	O3	C23	C24	C24A	-179.2(5)
N3	C52	C42	C41	177.8(6)	O8	C73	C74	N4	12.7(6)
N4	C72	C62	C61	177.8(6)	O8	C73	C74	C76	-101.6(6)
N4	C72	C62	C63	3.5(9)	O8	C73	C74	C75	130.9(6)
C68A	C69	C69A	C61	-176.1(6)	O8	C72	C62	C61	1.0(8)
C68A	C69	C69A	C64A	-0.2(6)	O8	C72	C62	C63	-173.3(5)
C68A	C68	C67	C66	-1.5(9)	O5	C43	C42	C52	-5.6(8)
C68A	C68	C67	Br4	176.0(4)	O5	C43	C42	C41	178.5(5)
C68A	C64B	C64A	C69A	-0.4(7)	O6	C52	C42	C43	-174.0(5)
C68A	C64B	C64A	C64	178.1(6)	O6	C52	C42	C41	1.9(8)
C68A	C64B	C65	C66	-1.7(9)	O6	C53	C54	N3	12.2(6)
C69	C68A	C68	C67	-177.3(6)	O6	C53	C54	C55	-102.6(6)
C69	C68A	C64B	C64A	0.3(7)	O6	C53	C54	C56	130.8(5)
C69	C68A	C64B	C65	179.0(5)	C44A	C49A	C49	C48A	-0.5(6)
C69	C69A	C61	C62	176.1(6)	C44A	C49A	C49	C51	-119.7(6)

C69	C69A	C64A	C64B	0.4(7)	C44A	C49A	C49	C50	116.1(5)
C69	C69A	C64A	C64	-178.3(5)	C44A	C49A	C41	C42	0.0(8)
C32	O4	C33	C34	-14.6(6)	C44A	C44B	C45	C46	177.7(6)
C32	N2	C34	C35	-134.7(6)	C44A	C44	C43	O5	179.5(5)
C32	N2	C34	C36	103.9(6)	C44A	C44	C43	C42	0.3(8)
C32	N2	C34	C33	-14.3(6)	C72	N4	C74	C76	106.8(6)
C32	C22	C23	C24	172.1(5)	C72	N4	C74	C73	-11.2(6)
C32	C22	C23	O3	-6.4(9)	C72	N4	C74	C75	-131.6(6)
C21	C22	C23	C24	-3.2(9)	C72	C62	C63	C64	173.1(5)
C21	C22	C23	O3	178.3(5)	C72	C62	C63	O7	-7.0(9)
C49A	C41	C42	C52	-173.7(5)	C70	C69	C69A	C61	65.4(8)
C49A	C41	C42	C43	2.1(9)	C70	C69	C69A	C64A	-118.7(6)
C29	C28A	C24B	C24A	0.9(7)	C63	C64	C64A	C64B	-176.5(6)
C29	C28A	C24B	C25	179.8(5)	C63	C64	C64A	C69A	1.9(9)
C68	C68A	C69	C69A	179.1(6)	C24B	C24A	C24	C23	-178.0(6)
C68	C68A	C69	C70	-61.0(8)	C24B	C24A	C29A	C21	175.9(5)
C68	C68A	C69	C71	61.7(8)	C24B	C24A	C29A	C29	-1.2(7)
C68	C68A	C64B	C64A	-179.0(5)	C24B	C25	C26	C27	2.0(9)
C68	C68A	C64B	C65	-0.3(9)	C41	C49A	C49	C48A	-177.4(6)
C48A	C44B	C45	C46	0.7(9)	C41	C49A	C49	C51	63.3(8)
C48A	C44B	C44A	C49A	-0.9(6)	C41	C49A	C49	C50	-60.8(8)
C48A	C44B	C44A	C44	179.3(6)	C41	C49A	C44A	C44B	178.1(5)
C27	C28	C28A	C29	-178.1(6)	C41	C49A	C44A	C44	-2.1(9)
C27	C28	C28A	C24B	1.6(9)	C30	C29	C28A	C28	-65.0(8)
C64B	C68A	C69	C69A	-0.1(6)	C30	C29	C28A	C24B	115.3(6)
C64B	C68A	C69	C70	119.8(6)	C30	C29	C29A	C24A	-114.5(6)
C64B	C68A	C69	C71	-117.5(6)	C30	C29	C29A	C21	68.6(8)
C64B	C68A	C68	C67	1.9(8)	C74	N4	C72	O8	5.5(7)
C28	C27	C26	C25	-0.3(9)	C74	N4	C72	C62	-171.3(5)
C28	C28A	C24B	C24A	-178.8(5)	C74	C73	O8	C72	-10.2(6)
C28	C28A	C24B	C25	0.1(9)	C33	O4	C32	N2	6.0(7)
C47	C46	C45	C44B	-0.2(9)	C33	O4	C32	C22	-178.9(5)

C22	C21	C29A	C24A	2.2(8)	C34	N2	C32	O4	5.9(7)
C22	C21	C29A	C29	178.9(5)	C34	N2	C32	C22	-168.6(6)
C22	C23	C24	C24A	2.3(9)	C71	C69	C69A	C61	-59.2(8)
C31	C29	C28A	C28	58.9(8)	C71	C69	C69A	C64A	116.7(6)
C31	C29	C28A	C24B	-120.8(5)	C42	C52	O6	C53	-180.0(5)

Table S9: Hydrogen Atom Coordinates (Å×10^4) and Isotropic Displacement Parameters (Å^2×10^3) for **Oxa-OH**.

Atom	*x*	*y*	*z*	U(eq)
H1	323.92	4091.99	7091	37
H1A	1245.47	1969.4	8323.99	18
H4	3660.84	3466.6	6776.5	19
H5	6334.04	2850.96	6713.91	22
H6	8531.44	2140.22	6861.72	23
H8	6805.38	635.58	8121.6	21
H10A	4875.77	887.61	9081.47	34
H10B	3301.22	1270.1	9140.37	34
H10C	4565.96	1737.98	9052.76	34
H11A	4224.82	291.09	8044.92	32
H11B	3386.45	769.2	7409.43	32
H11C	2639.78	641.11	8143.42	32
H13A	-2522.07	3184.74	9040.48	27
H13B	-3048.03	2920.74	8344.83	27
H15A	-1201.85	4244.64	8928.6	44
H15B	-2830.27	4558.14	8916.33	44
H15C	-1797.45	4882.03	8350.9	44
H16A	-3573.78	4643.24	7484.12	41
H16B	-4447.15	4207.69	8050.86	41
H16C	-3771.06	3834.88	7409.43	41
H21	4592.88	8000.41	6688.98	17
H66	3184.46	2089.03	1079.11	22
H68	5989.16	512.29	424.08	19
H28	9426.61	9338.74	6886.72	21

H25	10119.25	7116.2	8271.86	23
H31A	7740.78	8302.94	5939.35	33
H31B	7530.76	9149.46	5952.48	33
H31C	6196.84	8739.63	5905.93	33
H64	7100.87	3537.64	749.56	20
H24	7798.81	6488.71	8202.45	19
H44	1335.16	6450.95	4274.29	18
H61	10706.52	1977.22	-146.45	17
H48	-1320.96	9497.66	4572.21	20
H65	4894.23	2862.95	1007.36	22
H26	11928.32	7820.04	8122	26
H53A	7696.54	6755.31	6237.59	25
H53B	8289.61	6899.7	5464	25
H46	-3332.93	7972.53	3899.93	27
H76A	13181.4	4564.25	-1594.67	38
H76B	11912.31	4952.27	-1182.94	38
H76C	11723.21	4247.86	-1524.59	38
H45	-1236.58	7145.47	4028.68	24
H55A	6812.67	5042.21	6195.41	32
H55B	6324.88	5750.64	6548.93	32
H55C	7936.78	5406.2	6579.65	32
H73A	13681.1	3236.65	-1245.7	26
H73B	14330.88	3091.24	-475.35	26
H75A	14754.32	4447.65	-525.21	47
H75B	14200.08	4092	194.75	47
H75C	13471.41	4860.37	-141.94	47
H35A	89.03	5774.66	6965.56	40
H35B	600.87	6100.46	7623.66	40
H35C	1188.54	5298.63	7500.06	40
H36A	3429.12	5704.74	6084.21	39
H36B	1937.48	5426.77	6061.7	39
H36C	3069.11	5063.93	6638.45	39

H56A	8670.4	5890.94	4785.89	45
H56B	8386.38	5123.05	5146.56	45
H56C	9428.04	5559.78	5502.41	45
H3	4740.37	5886.25	7940.42	32
H7	9981.4	4253	228.61	36
H5A	4646.38	5764.7	4684.89	35
H70A	9147.84	842.24	1118.55	31
H70B	10092.37	661.64	465.32	31
H70C	8721.32	283.97	633.88	31
H51A	2082.23	9134.96	3900.54	30
H51B	2834.19	9329.74	4567.62	30
H51C	1281.37	9703.15	4360.24	30
H41	4093.89	8006.44	5150.27	19
H30A	5244.92	9290.53	6993.06	31
H30B	6627.39	9668.35	7012.43	31
H30C	6204.9	9158.56	7674.22	31
H33A	1524.27	6783.17	5969.18	27
H33B	825.08	7046.33	6664.98	27
H71A	7868.5	725.36	-585	34
H71B	9221.83	1121.69	-735.97	34
H71C	7687.98	1562.56	-847.31	34
H50A	2136.98	8879.77	5745.48	28
H50B	855.83	8424.91	5860.25	28
H50C	566.09	9261.61	5598.29	28

4.1.2 Crystal Packing Views of Oxa-OH

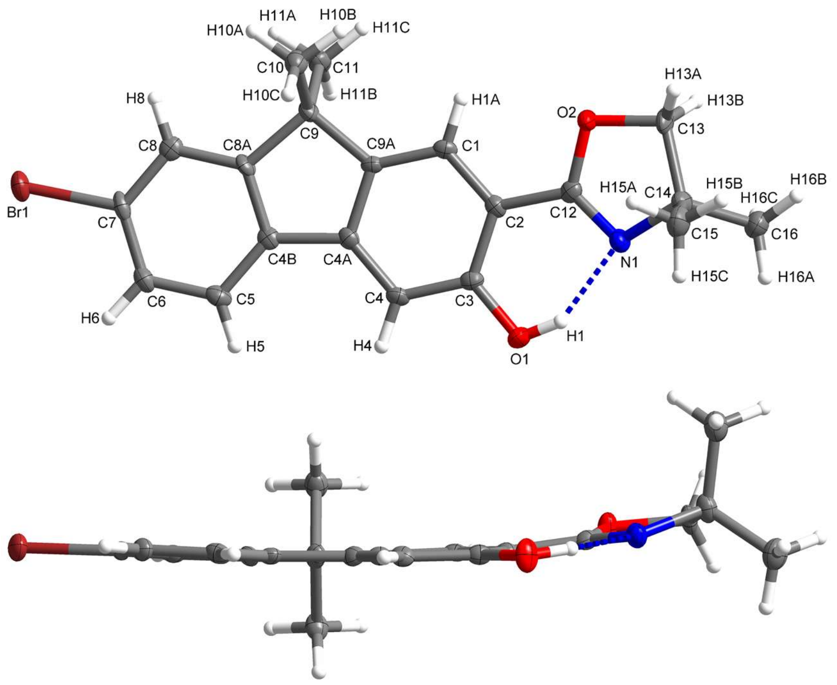

Figure S35: Top view with atom labels (top) and side view (bottom) of **Oxa-OH** (molecule 1).

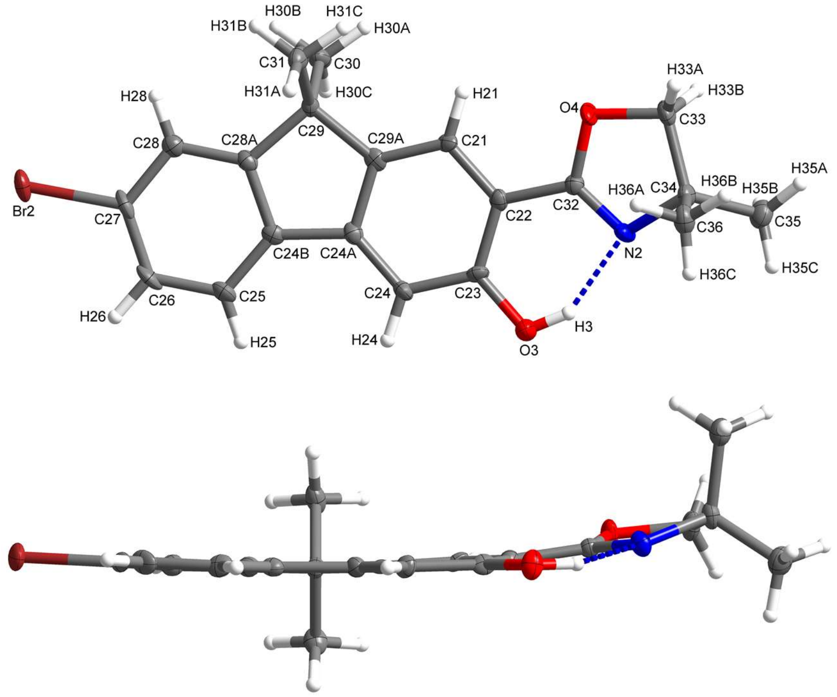

Figure S36: Top view with atom labels (top) and side view (bottom) of **Oxa-OH** (molecule 2).

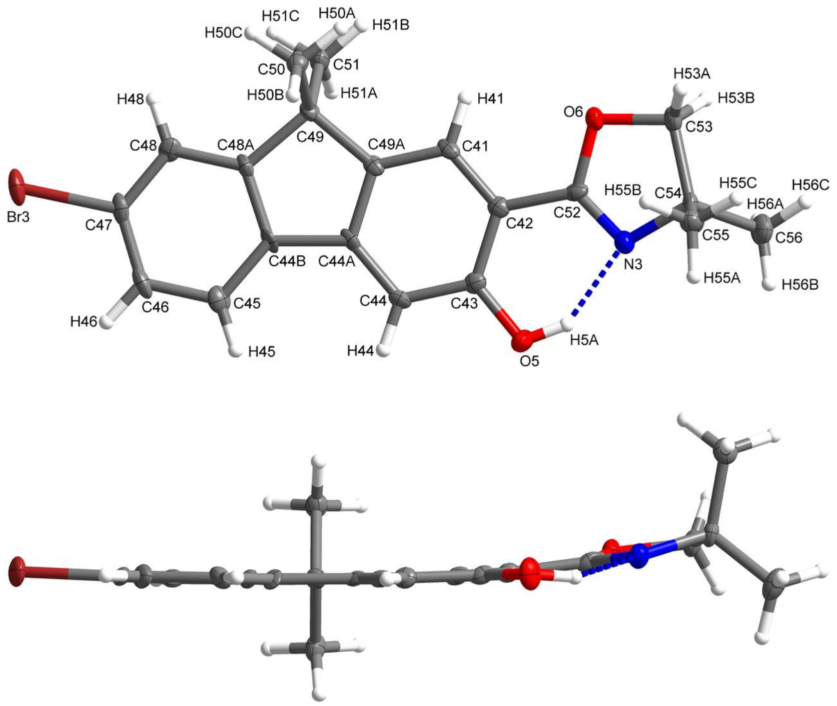

Figure S37: Top view with atom labels (top) and side view (bottom) of **Oxa-OH** (molecule 3).

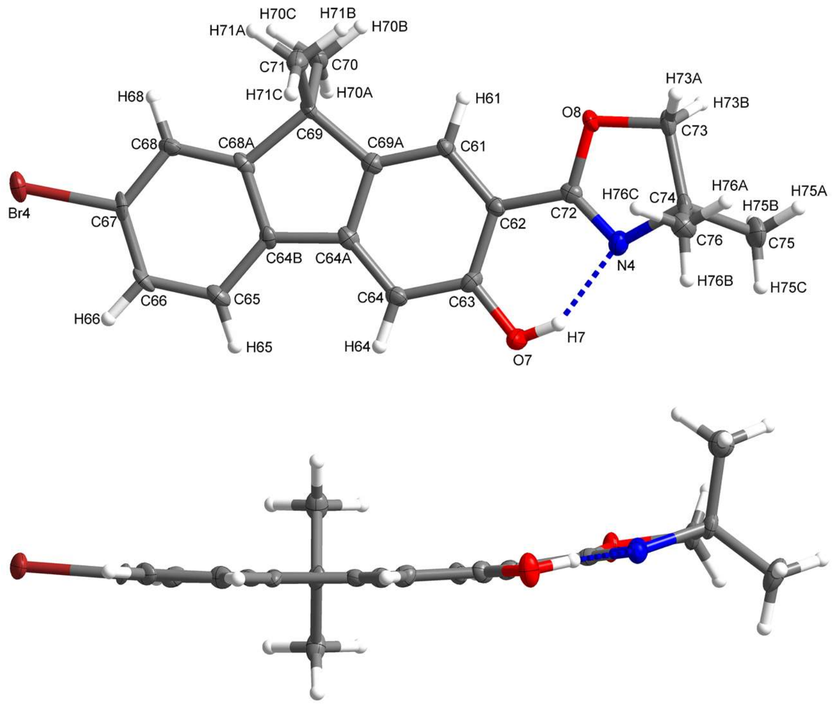

Figure S38: Top view with atom labels (top) and side view (bottom) of **Oxa-OH** (molecule 4).

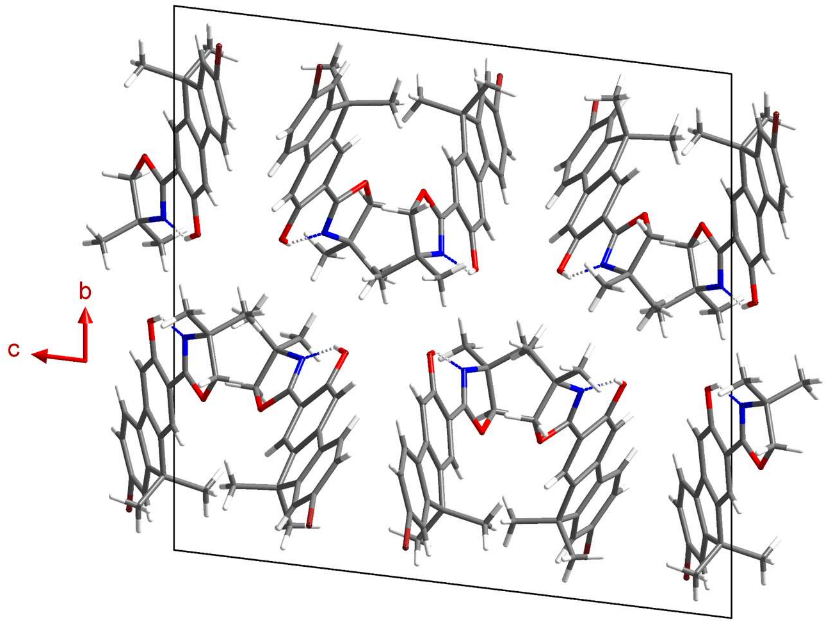

Figure S39: Unit cell packing view of an **Oxa-OH** crystal along the *a*-axis.

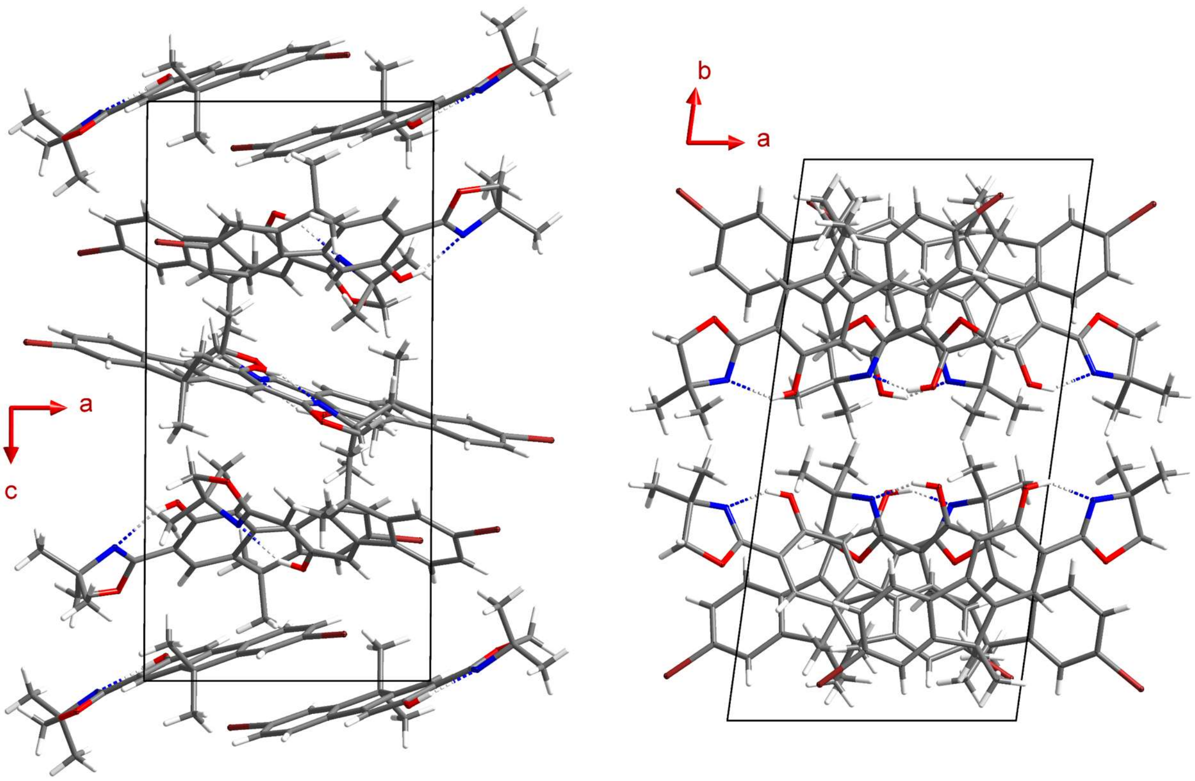

Figure S40: Unit cell packing view of an **Oxa-OH** crystal along the *b*-axis (left) and along the *c*-axis (right).

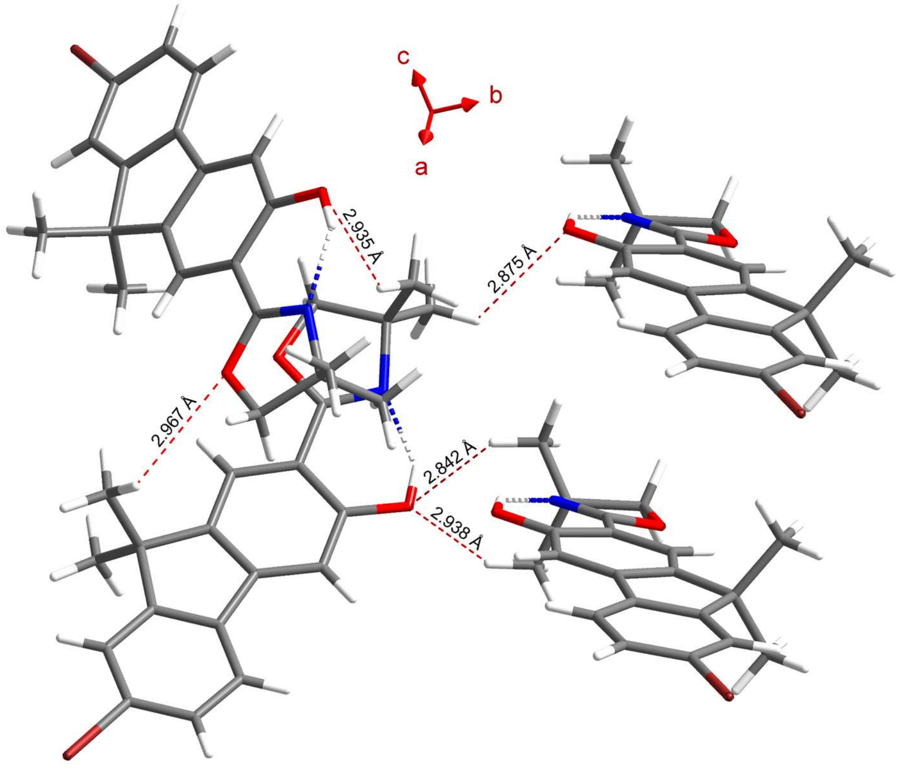

Figure S41: Various intermolecular interactions in **Oxa-OH** crystal lattice up to 3.000 Å distances, including C–H···O (red dashed lines) interactions. All distances are given in Å.

4.2 2,7-Bis(4,4-dimethyl-4,5-dihydrooxazol-2-yl)-9,9-dimethyl-9*H*-fluoren-3-ol ((Oxa)$_2$-OH)

4.2.1 Crystal Data for (Oxa)$_2$-OH

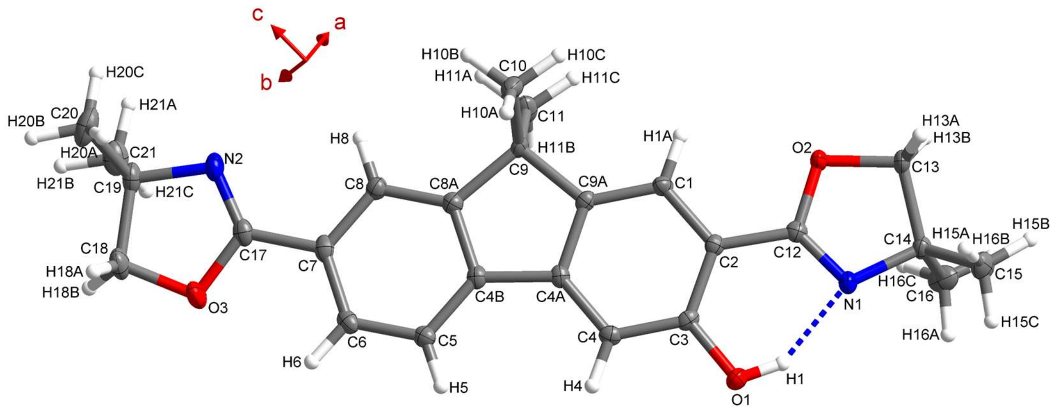

Figure S42: Asymmetric unit of **(Oxa)$_2$-OH** with atom labels.

Table S10: Crystal data and structure refinement for **(Oxa)$_2$-OH**.

Empirical formula	$C_{25}H_{28}N_2O_3$
Formula weight/g mol^{-1}	404.49
Crystal system	Orthorhombic
Crystal size/mm^3	0.28 × 0.13 × 0.11
Space group	$Pna2_1$
a/Å	10.1266(4)
b/Å	21.0008(9)
c/Å	10.2483(4)
α/°	90
β/°	90
γ/°	90
Volume/Å^3	2179.47(15)
Z	4
$\rho_{calc,}$ g/cm^3	1.233
μ/mm^{-1}	0.081
F(000)	864.0
2Θ range for data collection/°	3.878 to 56.564
Index ranges	$-13 \leq h \leq 12$, $-28 \leq k \leq 28$, $-13 \leq l \leq 13$
No. of reflections collected	35122
No. of independent reflections	5406 [R_{int} = 0.0468, R_{sigma} = 0.0356]
Data/restraints/parameters	5406/1/278
Goodness-of-fit on F^2	1.030
Final R indexes [I ≥ 2σ (I)]	R_1 = 0.0414, wR_2 = 0.0932
Final R indexes [all data]	R_1 = 0.0496, wR_2 = 0.0968
Largest diff. peak/hole / e Å^{-3}	0.29/–0.28
Flack parameter	0.3(3)
CCDC number	2018050

Table S11: Fractional Atomic Coordinates ($\times 10^4$) and Equivalent Isotropic Displacement Parameters ($Å^2 \times 10^3$) for **(Oxa)₂-OH**. U_{eq} is defined as 1/3 of the trace of the orthogonalized U_{IJ} tensor.

Atom	*x*	*y*	*z*	U(eq)
O1	-7244.7(15)	-6111.4(7)	-9594.1(16)	19.3(3)
O2	-3825.5(15)	-6942.2(7)	-8031.9(15)	17.4(3)
O3	-9784.6(17)	-3451.2(8)	-2265.4(18)	25.0(4)
N1	-5129.4(18)	-6816.1(8)	-9800.8(18)	15.8(4)
N2	-8361(2)	-3975.3(9)	-939(2)	22.1(4)
C1	-5472(2)	-6211.9(10)	-6450(2)	14.7(4)
C2	-5797(2)	-6321.7(10)	-7763(2)	13.7(4)
C3	-6915(2)	-6024.2(10)	-8322(2)	13.7(4)
C4	-7684(2)	-5611.8(10)	-7579(2)	14.3(4)
C4A	-7347(2)	-5511.4(9)	-6285(2)	13.1(4)
C4B	-7935(2)	-5083.5(9)	-5314(2)	13.8(4)
C5	-9018(2)	-4676.9(10)	-5395(2)	16.1(4)
C6	-9317(2)	-4294.0(10)	-4327(2)	16.9(4)
C7	-8553(2)	-4324.6(10)	-3195(2)	16.9(4)
C8	-7485(2)	-4746.3(10)	-3103(2)	16.9(4)
C8A	-7186(2)	-5124.1(10)	-4164(2)	15.0(4)
C9	-6077(2)	-5610.5(10)	-4296(2)	14.4(4)
C9A	-6249(2)	-5811.3(10)	-5712(2)	13.4(4)
C10	-6294(2)	-6173.1(11)	-3369(2)	21.5(5)
C11	-4715(2)	-5308.2(11)	-4060(3)	21.4(5)
C12	-4934(2)	-6707.2(10)	-8590(2)	14.1(4)
C13	-3228(2)	-7341.4(11)	-9034(2)	21.0(5)
C14	-3935(2)	-7143.9(10)	-10305(2)	16.5(4)
C15	-4326(3)	-7711.5(11)	-11140(3)	25.8(5)
C16	-3140(2)	-6661.9(13)	-11081(3)	26.5(5)
C17	-8862(2)	-3919.9(10)	-2058(2)	17.8(5)
C18	-10071(2)	-3200.8(12)	-974(3)	26.8(6)
C19	-8954(2)	-3465.1(11)	-115(2)	22.0(5)
C20	-9462(3)	-3768.9(13)	1144(3)	32.6(6)

C21	-7899(3)	-2964.2(12)	172(3)	28.6(6)

Table S12: Bond Lengths for **(Oxa)$_2$-OH**.

Atom	Atom	Length/Å	Atom	Atom	Length/Å
O1	C3	1.359(3)	C4B	C5	1.393(3)
O2	C12	1.353(3)	C4B	C8A	1.404(3)
O2	C13	1.458(3)	C5	C6	1.392(3)
O3	C17	1.373(3)	C6	C7	1.396(3)
O3	C18	1.453(3)	C7	C8	1.401(3)
N1	C12	1.277(3)	C7	C17	1.476(3)
N1	C14	1.485(3)	C8	C8A	1.379(3)
N2	C17	1.259(3)	C8A	C9	1.524(3)
N2	C19	1.490(3)	C9	C9A	1.521(3)
C1	C2	1.404(3)	C9	C10	1.532(3)
C1	C9A	1.379(3)	C9	C11	1.538(3)
C2	C3	1.414(3)	C13	C14	1.543(3)
C2	C12	1.462(3)	C14	C15	1.520(3)
C3	C4	1.391(3)	C14	C16	1.518(3)
C4	C4A	1.385(3)	C18	C19	1.537(4)
C4A	C4B	1.467(3)	C19	C20	1.528(4)
C4A	C9A	1.406(3)	C19	C21	1.528(3)

Table S13: Bond Angles for **(Oxa)$_2$-OH**.

Atom	Atom	Atom	Angle/°	Atom	Atom	Atom	Angle/°
C12	O2	C13	104.85(17)	C8A	C9	C11	111.75(17)
C17	O3	C18	104.70(18)	C9A	C9	C8A	100.72(17)
C12	N1	C14	107.15(18)	C9A	C9	C10	111.19(17)
C17	N2	C19	106.69(19)	C9A	C9	C11	111.50(18)
C9A	C1	C2	119.52(19)	C10	C9	C11	110.45(19)
C1	C2	C3	120.19(19)	C1	C9A	C4A	119.7(2)
C1	C2	C12	120.44(19)	C1	C9A	C9	128.90(19)
C3	C2	C12	119.23(19)	C4A	C9A	C9	111.40(18)

O1	C3	C2	121.73(19)	O2	C12	C2	116.89(19)
O1	C3	C4	118.11(19)	N1	C12	O2	118.3(2)
C4	C3	C2	120.1(2)	N1	C12	C2	124.8(2)
C4A	C4	C3	118.70(19)	O2	C13	C14	104.32(16)
C4	C4A	C4B	130.0(2)	N1	C14	C13	102.08(18)
C4	C4A	C9A	121.78(19)	N1	C14	C15	110.30(18)
C9A	C4A	C4B	108.16(19)	N1	C14	C16	107.75(18)
C5	C4B	C4A	130.9(2)	C15	C14	C13	112.68(19)
C5	C4B	C8A	120.8(2)	C16	C14	C13	112.06(19)
C8A	C4B	C4A	108.24(18)	C16	C14	C15	111.5(2)
C6	C5	C4B	118.6(2)	O3	C17	C7	115.8(2)
C5	C6	C7	120.5(2)	N2	C17	O3	118.8(2)
C6	C7	C8	120.8(2)	N2	C17	C7	125.5(2)
C6	C7	C17	120.8(2)	O3	C18	C19	104.15(18)
C8	C7	C17	118.4(2)	N2	C19	C18	103.4(2)
C8A	C8	C7	118.7(2)	N2	C19	C20	108.27(19)
C4B	C8A	C9	111.38(19)	N2	C19	C21	108.80(19)
C8	C8A	C4B	120.56(19)	C20	C19	C18	112.8(2)
C8	C8A	C9	128.1(2)	C21	C19	C18	112.1(2)
C8A	C9	C10	110.90(18)	C21	C19	C20	111.1(2)

Table S14: Torsion Angles for **$(Oxa)_2$-OH**.

A	B	C	D	Angle/°	A	B	C	D	Angle/°
O1	C3	C4	C4A	-179.06(18)	C7	C8	C8A	C4B	0.4(3)
O2	C13	C14	N1	-17.8(2)	C7	C8	C8A	C9	179.6(2)
O2	C13	C14	C15	-136.1(2)	C8	C7	C17	O3	170.58(19)
O2	C13	C14	C16	97.2(2)	C8	C7	C17	N2	-9.9(3)
O3	C18	C19	N2	-14.6(2)	C8	C8A	C9	C9A	-176.0(2)
O3	C18	C19	C20	-131.3(2)	C8	C8A	C9	C10	66.2(3)
O3	C18	C19	C21	102.4(2)	C8	C8A	C9	C11	-57.5(3)
C1	C2	C3	O1	178.87(19)	C8A	C4B	C5	C6	2.3(3)
C1	C2	C3	C4	1.2(3)	C8A	C9	C9A	C1	174.7(2)

C1	C2	C12	O2	-0.5(3)	C8A	C9	C9A	C4A	-3.1(2)
C1	C2	C12	N1	-177.8(2)	C9A	C1	C2	C3	-0.1(3)
C2	C1	C9A	C4A	-0.8(3)	C9A	C1	C2	C12	175.5(2)
C2	C1	C9A	C9	-178.5(2)	C9A	C4A	C4B	C5	179.2(2)
C2	C3	C4	C4A	-1.3(3)	C9A	C4A	C4B	C8A	0.4(2)
C3	C2	C12	O2	175.20(18)	C10	C9	C9A	C1	-67.7(3)
C3	C2	C12	N1	-2.1(3)	C10	C9	C9A	C4A	114.5(2)
C3	C4	C4A	C4B	176.5(2)	C11	C9	C9A	C1	56.1(3)
C3	C4	C4A	C9A	0.4(3)	C11	C9	C9A	C4A	-121.77(19)
C4	C4A	C4B	C5	2.7(4)	C12	O2	C13	C14	15.3(2)
C4	C4A	C4B	C8A	-176.2(2)	C12	N1	C14	C13	14.3(2)
C4	C4A	C9A	C1	0.7(3)	C12	N1	C14	C15	134.3(2)
C4	C4A	C9A	C9	178.75(18)	C12	N1	C14	C16	-103.8(2)
C4A	C4B	C5	C6	-176.5(2)	C12	C2	C3	O1	3.2(3)
C4A	C4B	C8A	C8	176.91(18)	C12	C2	C3	C4	-174.50(18)
C4A	C4B	C8A	C9	-2.4(2)	C13	O2	C12	N1	-6.9(3)
C4B	C4A	C9A	C1	-176.19(19)	C13	O2	C12	C2	175.57(18)
C4B	C4A	C9A	C9	1.9(2)	C14	N1	C12	O2	-5.3(3)
C4B	C5	C6	C7	-0.8(3)	C14	N1	C12	C2	171.94(19)
C4B	C8A	C9	C9A	3.3(2)	C17	O3	C18	C19	14.0(2)
C4B	C8A	C9	C10	-114.5(2)	C17	N2	C19	C18	9.9(2)
C4B	C8A	C9	C11	121.8(2)	C17	N2	C19	C20	129.7(2)
C5	C4B	C8A	C8	-2.1(3)	C17	N2	C19	C21	-109.4(2)
C5	C4B	C8A	C9	178.57(19)	C17	C7	C8	C8A	-179.74(19)
C5	C6	C7	C8	-0.9(3)	C18	O3	C17	N2	-8.9(3)
C5	C6	C7	C17	179.97(19)	C18	O3	C17	C7	170.60(19)
C6	C7	C8	C8A	1.1(3)	C19	N2	C17	O3	-1.0(3)
C6	C7	C17	O3	-10.3(3)	C19	N2	C17	C7	179.5(2)
C6	C7	C17	N2	169.2(2)					

Table S15: Hydrogen Atom Coordinates (Å×10^4) and Isotropic Displacement Parameters (Å^2×10^3) for **(Oxa)$_2$-OH**.

Atom	*x*	*y*	*z*	U(eq)
H1	-6685.37	-6349.67	-9951.81	29
H1A	-4721.65	-6412.11	-6074.16	18
H4	-8425.81	-5403.25	-7951.86	17
H5	-9542.24	-4661.34	-6163.32	19
H6	-10045.1	-4009.67	-4368.39	20
H8	-6975.73	-4771.48	-2326.34	20
H10A	-7174.15	-6353.26	-3514.17	32
H10B	-6221.67	-6026.21	-2463.69	32
H10C	-5623.49	-6499.88	-3534.65	32
H11A	-4646.69	-5171.81	-3148.05	32
H11B	-4606.16	-4938.66	-4634.18	32
H11C	-4023.05	-5621.39	-4248.04	32
H13A	-3374.25	-7798.19	-8844.86	25
H13B	-2266.09	-7261.58	-9096.74	25
H15A	-4897.02	-7995.9	-10633.83	39
H15B	-3531.25	-7942.31	-11411.73	39
H15C	-4802.88	-7562.1	-11914.3	39
H16A	-3675.77	-6501.94	-11806.81	40
H16B	-2342.54	-6865.97	-11424.1	40
H16C	-2890.33	-6306.57	-10512.26	40
H18A	-10942.67	-3349.83	-662.35	32
H18B	-10062.47	-2729.36	-977.47	32
H20A	-10058.87	-4121.04	929.36	49
H20B	-9939.08	-3449.33	1657.23	49
H20C	-8714.43	-3930.97	1651.81	49
H21A	-7165.74	-3162.04	646.23	43
H21B	-8281.72	-2623.23	703.74	43
H21C	-7572.38	-2786.04	-651.2	43

4.2.2 Crystal Packing Views of (Oxa)$_2$-OH

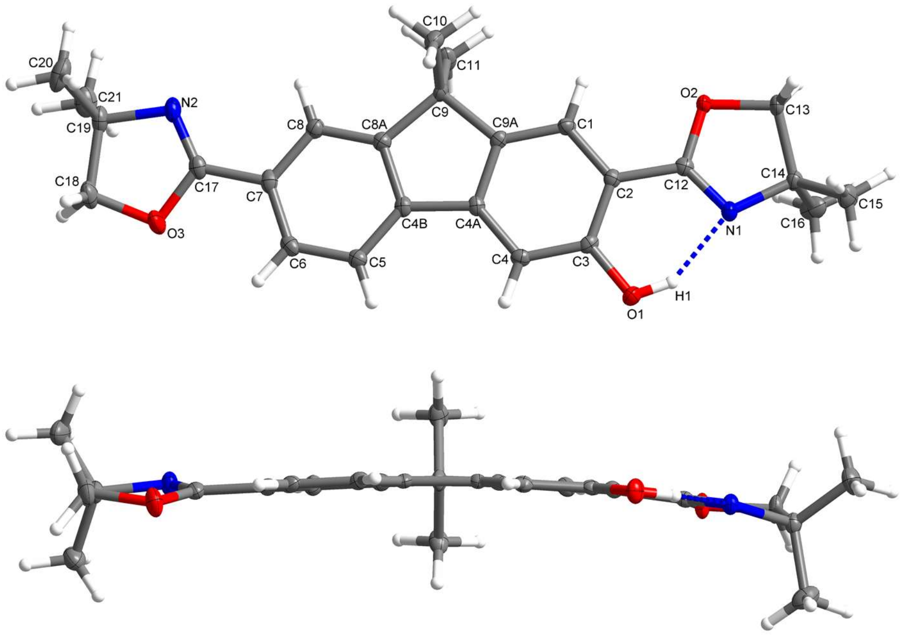

Figure S43: Top view with atom labels (top) and side view (bottom) of **(Oxa)$_2$-OH**.

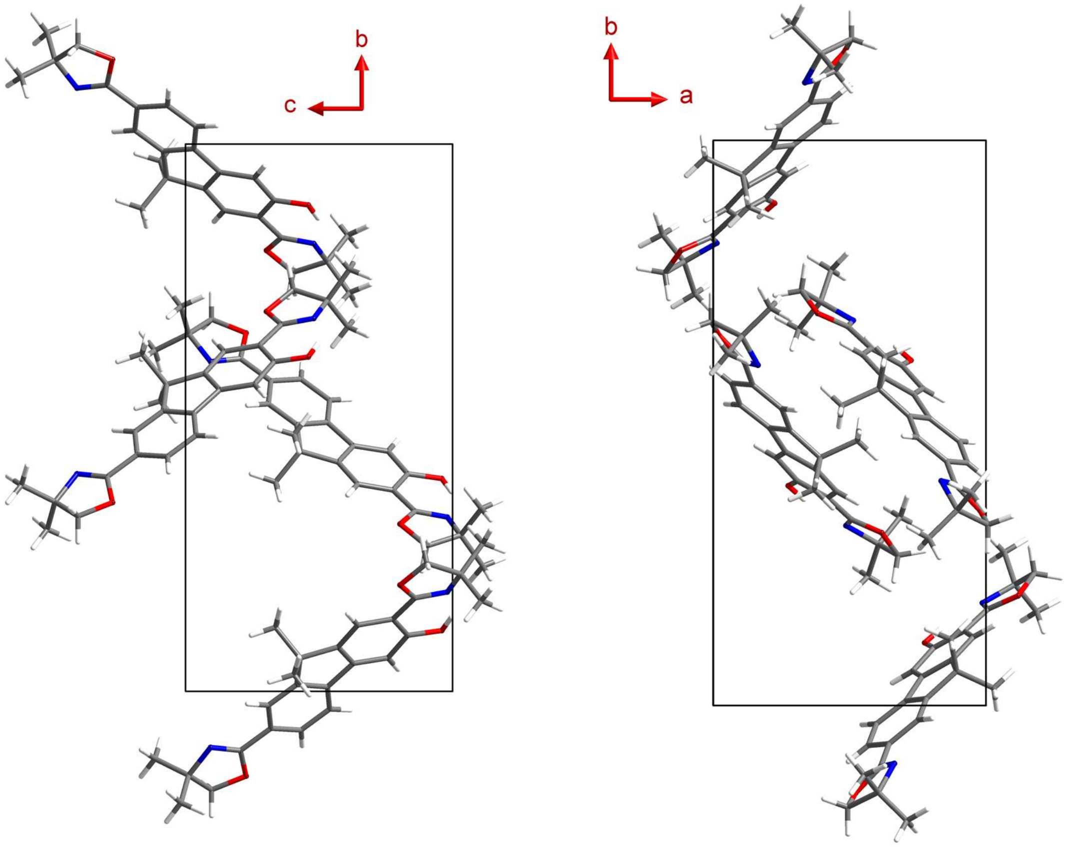

Figure S44: Unit cell packing view of an **(Oxa)$_2$-OH** crystal along the *a*-axis (left) and the *c*-axis (right).

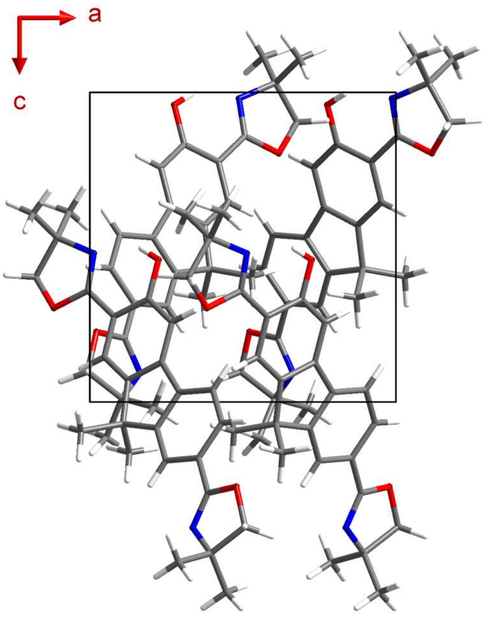

Figure S45: Unit cell packing view of an **(Oxa)$_2$-OH** crystal along the *b*-axis.

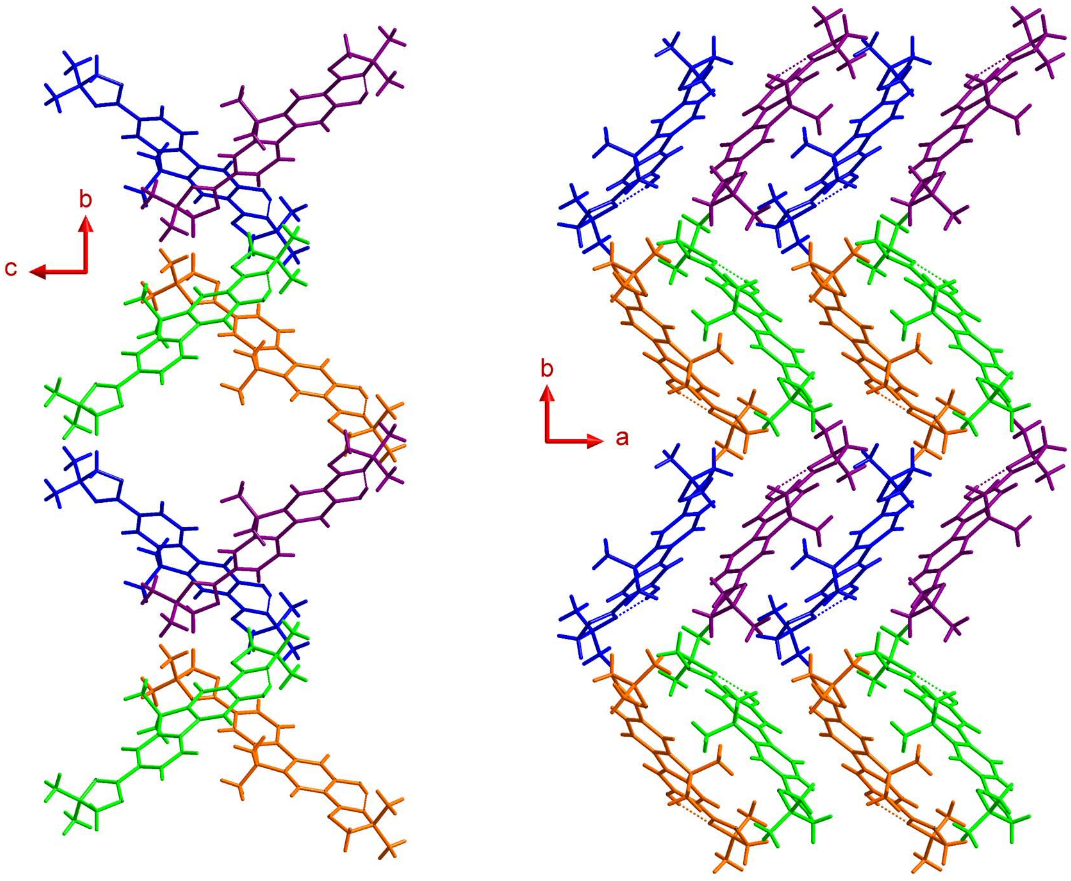

Figure S46: Crystal packing of **(Oxa)$_2$-OH** along the *a*-axis (left) and the *c*-axis (right). For a greater clarity, identical molecules are colored violet, blue, green and orange.

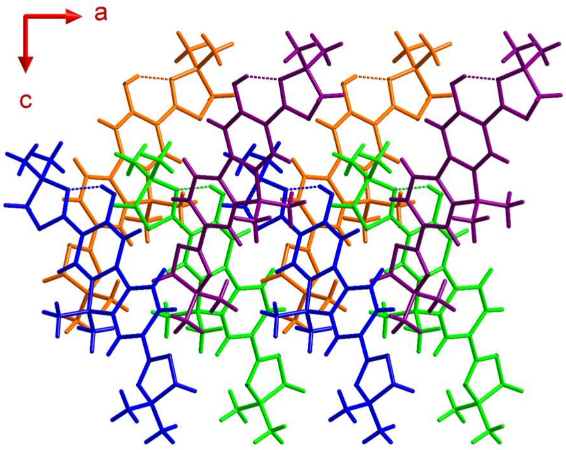

Figure S47: Crystal packing of **(Oxa)₂-OH** along the *b*-axis. For a greater clarity, identical molecules are colored violet, blue, green and orange.

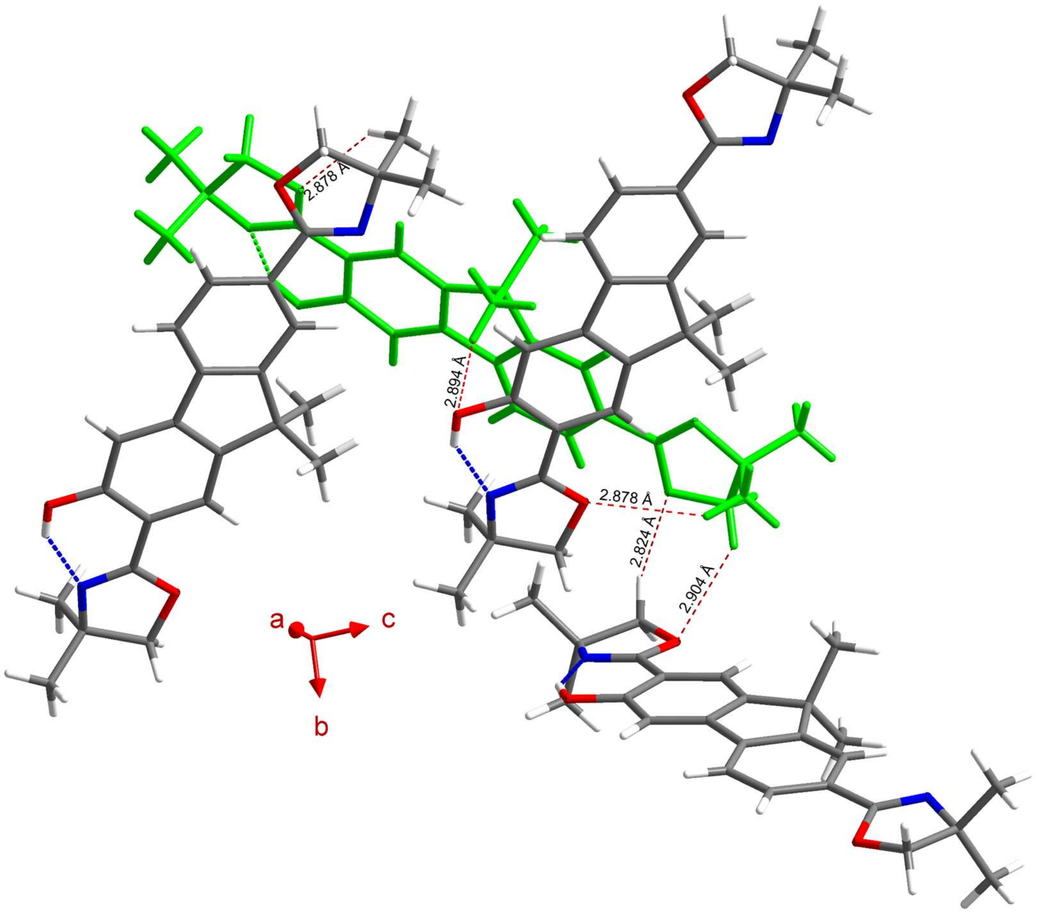

Figure S48: Various intermolecular interactions in **(Oxa)₂-OH** crystal lattice up to 3.000 Å distances, including C–H···O (red dotted lines) interactions. All distances are given in Å. For a greater clarity, one definite molecule is colored green.

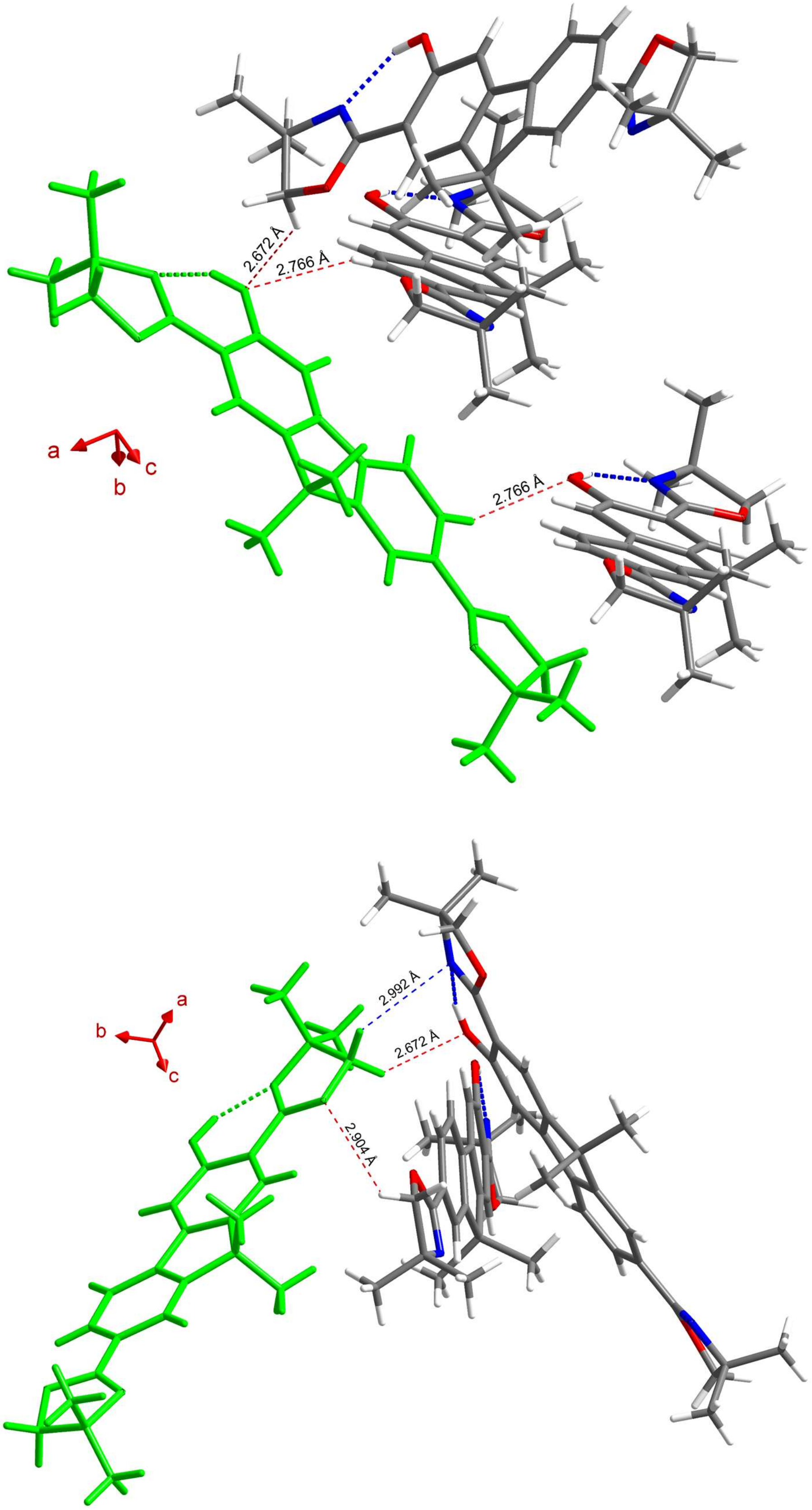

Figure S49: Various intermolecular interactions in **(Oxa)$_2$-OH** crystal lattice up to 3.000 Å distances, including C–H···O (red dashed lines) and C–H···N (blue dashed lines) interactions. All distances are given in Å. For a greater clarity, one definite molecule is colored green.

4.3 2,7-Bis(4,4-dimethyl-4,5-dihydrooxazol-2-yl)-9,9-dimethyl-9*H*-fluorene-3,6-diol ((Oxa-OH)$_2$)

4.3.1 Crystal Data for (Oxa-OH)$_2$

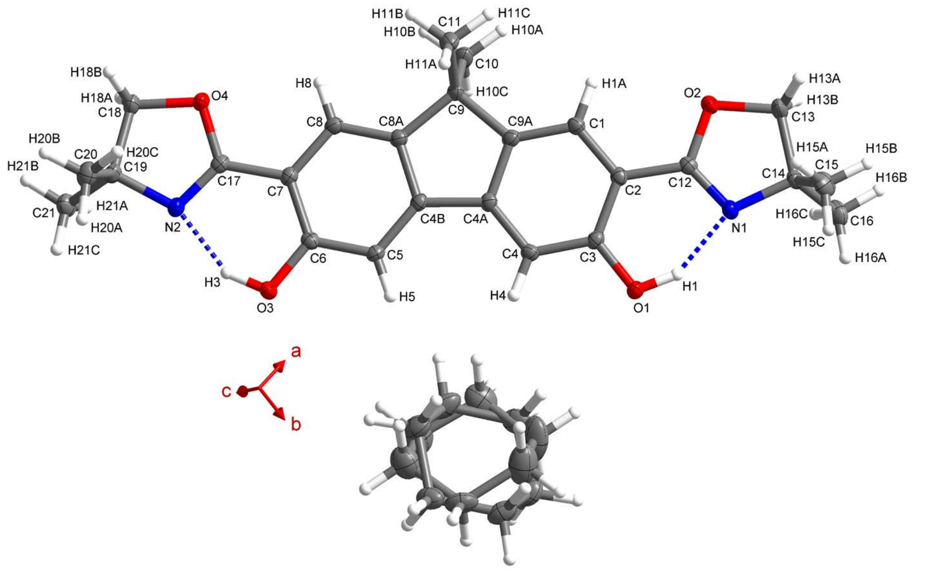

Figure S50: Asymmetric unit of **(Oxa-OH)$_2$** with atom labels.

Table S16: Crystal data and structure refinement for **(Oxa-OH)$_2$**.

Empirical formula	$C_{31}H_{40}N_2O_4$
Formula weight/g mol^{-1}	504.65
Crystal system	Orthorhombic
Crystal size/mm^3	0.5 × 0.3 × 0.3
Space group	Pccn
a/Å	20.5800(6)
b/Å	24.0995(7)
c/Å	11.4414(3)
α/°	90
β/°	90
γ/°	90
Volume/Å^3	5674.6(3)
Z	8
ρ_{calc}, g/cm^3	1.181
μ/mm^{-1}	0.078

F(000)	2176.0
2Θ range for data collection/°	4.41 to 56.56
Index ranges	$-25 \leq h \leq 27$, $-32 \leq k \leq 29$, $-15 \leq l \leq 12$
No. of reflections collected	61897
No. of independent reflections	7040 [R_{int} = 0.0434, R_{sigma} = 0.0258]
Data/restraints/parameters	7040/36/404
Goodness-of-fit on F^2	1.029
Final R indexes [$I \geq 2\sigma$ (I)]	R_1 = 0.0425, wR_2 = 0.0960
Final R indexes [all data]	R_1 = 0.0592, wR_2 = 0.1052
Largest diff. peak/hole / e Å^{-3}	0.32/–0.21
CCDC number	2018045

Table S17: Fractional Atomic Coordinates (×10^4) and Equivalent Isotropic Displacement Parameters (Å^2×10^3) for **(Oxa-OH)$_2$**. U_{eq} is defined as 1/3 of the trace of the orthogonalized U_{IJ} tensor.

Atom	*x*	*y*	*z*	U(eq)
O2	7760.5(4)	4739.4(4)	4079.5(8)	21.9(2)
O1	5962.7(4)	5487.9(3)	4729.7(8)	18.92(19)
O4	4896.3(4)	2207.0(3)	9604.4(8)	19.93(19)
O3	3955.7(4)	3715.6(4)	8966.4(8)	20.06(19)
N1	7104.6(5)	5477.1(4)	3788.7(8)	16.7(2)
N2	4008.4(5)	2737.8(4)	9908.0(9)	17.2(2)
C1	6840.9(6)	4203.7(5)	5501.3(10)	16.7(2)
C2	6706.2(5)	4719.9(5)	4986.0(10)	15.3(2)
C3	6110.3(5)	4991.1(5)	5212.5(10)	15.2(2)
C4B	5429.1(5)	3911.2(5)	7301.8(10)	14.7(2)
C4A	5806.8(5)	4244.9(5)	6474.7(10)	14.8(2)
C4	5658.9(5)	4751.1(5)	5964.9(10)	15.6(2)
C5	4823.6(6)	4013.8(5)	7782.0(10)	15.5(2)
C6	4550.9(5)	3615.6(5)	8515.4(10)	15.8(2)
C7	4891.8(6)	3117.7(5)	8749.9(10)	15.5(2)
C8	5510.1(5)	3028.1(5)	8268.2(10)	16.5(2)

C8A	5778.6(5)	3422.0(5)	7543.7(10)	15.9(2)
C9	6424.9(5)	3411.9(5)	6891.6(10)	17.4(2)
C9A	6392.9(5)	3964.5(5)	6241.6(10)	16.2(2)
C10	7000.9(6)	3386.2(6)	7743.9(13)	25.8(3)
C11	6459.1(6)	2924.3(5)	6026.8(12)	24.5(3)
C12	7185.5(5)	4998.9(5)	4254.7(10)	15.6(2)
C13	8156.5(6)	5129.0(6)	3422.1(13)	30.0(3)
C14	7699.1(5)	5616.1(5)	3120.8(10)	17.3(2)
C15	7527.3(7)	5636.8(6)	1829.3(10)	25.5(3)
C16	7969.8(6)	6169.7(6)	3529.4(12)	27.0(3)
C17	4574.4(6)	2693.0(5)	9456.1(10)	15.8(2)
C18	4503.1(6)	1894.8(5)	10437.3(11)	21.0(3)
C19	3841.2(6)	2192.6(5)	10436.5(10)	17.5(2)
C20	3351.9(6)	1902.8(5)	9646.4(11)	24.8(3)
C21	3574.0(6)	2272.7(5)	11662.4(11)	23.9(3)
C30	4082.2(12)	5424.2(8)	8190(2)	33.2(5)
C31	4697.6(15)	5584.4(9)	8852(2)	36.8(6)
C32	5050.7(11)	6060.9(11)	8259(3)	33.1(6)
C33	4602.6(10)	6557.7(8)	8068.8(17)	31.8(5)
C34	3996.1(11)	6391.0(9)	7393.2(19)	31.7(5)
C35	3638.9(12)	5923.0(11)	8014(3)	30.4(6)
C40	3784(5)	6106(5)	7686(9)	33(2)
C41	3810(5)	5607(5)	8471(9)	46(2)
C42	4377(6)	5658(5)	9328(9)	54(3)
C43	5007(5)	5750(6)	8655(9)	42(2)
C44	4957(6)	6223(7)	7849(12)	60(4)
C45	4406(5)	6169(5)	7000(9)	56(3)

Table S18: Bond Lengths for **(Oxa-OH)₂**.

Atom	Atom	Length/Å	Atom	Atom	Length/Å
O2	C12	1.3534(14)	C2	C3	1.4137(16)
O2	C13	1.4531(15)	C2	C12	1.4579(16)

C30a	C31	1.525(3)	C3	C4	1.3923(16)
C31a	C32	1.519(4)	C4B	C4A	1.4650(15)
C32a	C33	1.527(3)	C4B	C5	1.3841(16)
C33a	C34	1.522(3)	C4B	C8A	1.4085(16)
C30a	C35	1.522(3)	C4A	C4	1.3860(16)
C34a	C35	1.522(3)	C4A	C9A	1.4081(16)
C40b	C41	1.502(14)	C5	C6	1.3928(16)
C41b	C42	1.529(14)	C6	C7	1.4156(16)
C42b	C43	1.525(14)	C7	C8	1.4034(16)
C43b	C44	1.471(15)	C7	C17	1.4584(15)
C40b	C45	1.510(14)	C8	C8A	1.3761(16)
C44b	C45	1.498(14)	C8A	C9	1.5251(16)
O1	C3	1.3530(14)	C9	C9A	1.5266(16)
O4	C17	1.3562(14)	C9	C10	1.5363(17)
O4	C18	1.4592(14)	C9	C11	1.5378(17)
O3	C6	1.3509(14)	C13	C14	1.5437(17)
N1	C12	1.2806(15)	C14	C15	1.5202(16)
N1	C14	1.4809(14)	C14	C16	1.5195(17)
N2	C17	1.2791(15)	C18	C19	1.5396(17)
N2	C19	1.4866(14)	C19	C20	1.5229(17)
C1	C2	1.4043(16)	C19	C21	1.5189(17)
C1	C9A	1.3783(16)			

Table S19: Bond Angles for **(Oxa-OH)$_2$**.

Atom	Atom	Atom	Angle/°	Atom	Atom	Atom	Angle/°
C12	O2	C13	105.58(9)	C8	C8A	C9	129.34(10)
C34a	C35a	C30	110.9(2)	C8A	C9	C9A	100.76(9)
C32a	C31a	C30	111.54(18)	C8A	C9	C10	111.29(10)
C17	O4	C18	104.85(9)	C8A	C9	C11	111.52(10)
C12	N1	C14	108.13(10)	C9A	C9	C10	112.19(10)
C17	N2	C19	107.50(9)	C9A	C9	C11	110.79(10)
C9A	C1	C2	119.76(11)	C10	C9	C11	110.01(10)

C1	C2	C3	120.26(10)	C1	C9A	C4A	119.25(10)
C1	C2	C12	121.06(10)	C1	C9A	C9	129.44(10)
C3	C2	C12	118.61(10)	C4A	C9A	C9	111.31(10)
O1	C3	C2	121.94(10)	O2	C12	C2	117.61(10)
O1	C3	C4	118.05(10)	N1	C12	O2	117.89(10)
C4	C3	C2	120.00(10)	N1	C12	C2	124.48(11)
C5	C4B	C4A	129.50(10)	O2	C13	C14	105.36(10)
C5	C4B	C8A	122.09(10)	N1	C14	C13	102.50(9)
C8A	C4B	C4A	108.39(10)	N1	C14	C15	108.48(10)
C4	C4A	C4B	129.68(11)	N1	C14	C16	110.04(10)
C35a	C30a	C31	111.32(17)	C15	C14	C13	112.58(11)
C34a	C33a	C32	111.14(17)	C16	C14	C13	112.05(11)
C31a	C32a	C33	111.56(19)	C16	C14	C15	110.82(10)
C35a	C34a	C33	110.78(18)	O4	C17	C7	117.17(10)
C44b	C45b	C40	108.3(9)	N2	C17	O4	117.84(10)
C4	C4A	C9A	122.06(10)	N2	C17	C7	124.94(10)
C9A	C4A	C4B	108.26(10)	O4	C18	C19	104.47(9)
C4A	C4	C3	118.64(10)	N2	C19	C18	101.98(9)
C4B	C5	C6	118.62(10)	N2	C19	C20	108.48(10)
O3	C6	C5	118.20(10)	N2	C19	C21	110.31(10)
O3	C6	C7	121.89(10)	C20	C19	C18	111.81(10)
C5	C6	C7	119.90(10)	C21	C19	C18	112.27(10)
C6	C7	C17	118.55(10)	C21	C19	C20	111.53(10)
C8	C7	C6	120.36(10)	C43b	C42b	C41	109.7(8)
C8	C7	C17	121.04(10)	C44b	C43b	C42	111.7(10)
C8A	C8	C7	119.64(10)	C40b	C41b	C42	110.3(8)
C4B	C8A	C9	111.25(10)	C43b	C44b	C45	113.2(10)
C8	C8A	C4B	119.39(10)	C41b	C40b	C45	111.3(8)

Table S20: Torsion Angles for **(Oxa-OH)$_2$**.

A	B	C	D	Angle/°	A	B	C	D	Angle/°
C40b	C41b	C42b	C43b	-55.1(13)	C5	C4B	C8A	C8	0.81(17)

C31a	C32a	C33a	C34a	-55.0(3)	C5	C4B	C8A	C9	179.29(10)
C35a	C30a	C31a	C32a	-54.5(3)	C5	C6	C7	C8	1.44(17)
C41b	C40b	C45b	C44b	-58.7(13)	C5	C6	C7	C17	-175.86(10)
C33a	C34a	C35a	C30a	-56.9(3)	C6	C7	C8	C8A	-1.44(17)
C45b	C40b	C41b	C42b	59.2(12)	C6	C7	C17	O4	175.97(10)
C30a	C31a	C32a	C33a	54.0(3)	C6	C7	C17	N2	-1.42(17)
C43b	C44b	C45b	C40b	57.6(16)	C7	C8	C8A	C4B	0.34(17)
C32a	C33a	C34a	C35a	56.3(3)	C7	C8	C8A	C9	-177.84(11)
C41b	C42b	C43b	C44b	53.6(13)	C8	C7	C17	O4	-1.31(16)
C31a	C30a	C35a	C34a	56.0(3)	C8	C7	C17	N2	-178.69(11)
C42b	C43b	C44b	C45b	-56.3(15)	C8	C8A	C9	C9A	178.16(12)
O2	C13	C14	N1	7.19(13)	C8	C8A	C9	C10	-62.71(16)
O2	C13	C14	C15	-109.16(12)	C8	C8A	C9	C11	60.55(16)
O2	C13	C14	C16	125.13(11)	C8A	C4B	C4A	C4	178.60(11)
O1	C3	C4	C4A	-179.35(10)	C8A	C4B	C4A	C9A	-1.60(13)
O4	C18	C19	N2	-17.86(12)	C8A	C4B	C5	C6	-0.81(17)
O4	C18	C19	C20	97.85(11)	C8A	C9	C9A	C1	179.23(12)
O4	C18	C19	C21	-135.91(10)	C8A	C9	C9A	C4A	-0.88(12)
O3	C6	C7	C8	-179.88(10)	C9A	C1	C2	C3	-0.78(17)
O3	C6	C7	C17	2.82(16)	C9A	C1	C2	C12	176.34(11)
C1	C2	C3	O1	-179.83(10)	C9A	C4A	C4	C3	-0.94(17)
C1	C2	C3	C4	1.12(17)	C10	C9	C9A	C1	60.76(16)
C1	C2	C12	O2	0.80(16)	C10	C9	C9A	C4A	-119.35(11)
C1	C2	C12	N1	-177.63(11)	C11	C9	C9A	C1	-62.62(16)
C2	C1	C9A	C4A	-0.39(17)	C11	C9	C9A	C4A	117.26(11)
C2	C1	C9A	C9	179.49(11)	C12	O2	C13	C14	-6.63(13)
C2	C3	C4	C4A	-0.26(16)	C12	N1	C14	C13	-5.31(13)
C3	C2	C12	O2	177.96(10)	C12	N1	C14	C15	113.95(11)
C3	C2	C12	N1	-0.46(17)	C12	N1	C14	C16	-124.66(11)
C4B	C4A	C4	C3	178.85(11)	C12	C2	C3	O1	2.98(16)
C4B	C4A	C9A	C1	-178.55(10)	C12	C2	C3	C4	-176.07(10)
C4B	C4A	C9A	C9	1.55(13)	C13	O2	C12	N1	3.64(14)

C4B	C5	C6	O3	-179.04(10)	C13	O2	C12	C2	-174.89(11)
C4B	C5	C6	C7	-0.31(16)	C14	N1	C12	O2	1.32(14)
C4B	C8A	C9	C9A	-0.13(12)	C14	N1	C12	C2	179.75(10)
C4B	C8A	C9	C10	119.00(11)	C17	O4	C18	C19	16.21(12)
C4B	C8A	C9	C11	-117.74(11)	C17	N2	C19	C18	13.50(12)
C4A	C4B	C5	C6	177.04(11)	C17	N2	C19	C20	-104.62(11)
C4A	C4B	C8A	C8	-177.44(10)	C17	N2	C19	C21	132.95(10)
C4A	C4B	C8A	C9	1.04(13)	C17	C7	C8	C8A	175.79(10)
C4	C4A	C9A	C1	1.27(17)	C18	O4	C17	N2	-8.52(14)
C4	C4A	C9A	C9	-178.63(10)	C18	O4	C17	C7	173.90(10)
C5	C4B	C4A	C4	0.5(2)	C19	N2	C17	O4	-3.76(14)
C5	C4B	C4A	C9A	-179.67(11)	C19	N2	C17	C7	173.61(10)

Table S21: Hydrogen Atom Coordinates (Å×10^4) and Isotropic Displacement Parameters (Å^2×10^3) for **(Oxa-OH)$_2$**.

Atom	*x*	*y*	*z*	U(eq)
H1A	7239.38	4019.97	5340.27	20
H4	5257.75	4930.63	6125.5	19
H5	4598.75	4348.78	7614.82	19
H8	5741.97	2697.33	8441.34	20
H10A	7408.95	3402.73	7303.64	39
H10B	6982.75	3038.5	8186.88	39
H10C	6978.57	3700.92	8284.79	39
H11A	6089.18	2943.05	5488.86	37
H11B	6444.83	2573.45	6458.97	37
H11C	6865.08	2945.76	5581.45	37
H13A	8325.85	4953.77	2701.24	36
H13B	8528.06	5259.22	3898.46	36
H15A	7352.39	5276.8	1585.17	38
H15B	7918.21	5721.08	1373.36	38
H15C	7200.69	5925.94	1696.65	38
H16A	7663.18	6466.33	3331.38	41

H16B	8386.58	6239.83	3142.48	41
H16C	8034.06	6159.99	4377.83	41
H18A	4701.4	1902.84	11225.43	25
H18B	4455.51	1503.77	10186.03	25
H20A	2962.78	2134.25	9568.45	37
H20B	3232.43	1544.19	9988.46	37
H20C	3545.55	1843.39	8873.99	37
H21A	3884.63	2487.31	12128.4	36
H21B	3504.93	1909.77	12028.04	36
H21C	3160.06	2472.79	11621.56	36
H30A	4201.96	5267.92	7419.31	40
H30B	3846.57	5134.2	8632.57	40
H31A	4581.81	5693.66	9659.2	44
H31B	4990.3	5259.12	8896.57	44
H32A	5221.5	5934.21	7495.21	40
H32B	5424.36	6175.58	8747.23	40
H33A	4474.73	6713.7	8835.48	38
H33B	4838.44	6849.11	7630.82	38
H34A	3704.3	6715.54	7317.04	38
H34B	4120.24	6269.05	6597.68	38
H35A	3256.5	5811	7544.56	37
H35B	3481.62	6055.54	8782.28	37
H40A	3711.24	6442.8	8162.86	40
H40B	3414.36	6067.69	7137.79	40
H41A	3862.95	5267.49	7993.92	55
H41B	3396.97	5575.39	8910.53	55
H42A	4299.91	5973.24	9865.08	65
H42B	4410.4	5315.12	9801.4	65
H43A	5365.23	5817.9	9215.69	50
H43B	5113.96	5410.51	8206.59	50
H44A	5368.77	6257.6	7407.56	72
H44B	4898.11	6567.23	8308.66	72

H45A	4473.56	5841.56	6492.32	67
H45B	4382.67	6503.37	6497.65	67
H3	3843(9)	3400(8)	9410(17)	52(5)
H1	6315(10)	5599(8)	4301(17)	54(6)

Table S22: Atomic Occupancy for **(Oxa-OH)$_2$**.

Atom	*Occupancy*	Atom	*Occupancy*	Atom	*Occupancy*
C30	0.794(4)	H30A	0.794(4)	H30B	0.794(4)
C31	0.794(4)	H31A	0.794(4)	H31B	0.794(4)
C32	0.794(4)	H32A	0.794(4)	H32B	0.794(4)
C33	0.794(4)	H33A	0.794(4)	H33B	0.794(4)
C34	0.794(4)	H34A	0.794(4)	H34B	0.794(4)
C35	0.794(4)	H35A	0.794(4)	H35B	0.794(4)
C40	0.206(4)	H40A	0.206(4)	H40B	0.206(4)
C41	0.206(4)	H41A	0.206(4)	H41B	0.206(4)
C42	0.206(4)	H42A	0.206(4)	H42B	0.206(4)
C43	0.206(4)	H43A	0.206(4)	H43B	0.206(4)
C44	0.206(4)	H44A	0.206(4)	H44B	0.206(4)
C45	0.206(4)	H45A	0.206(4)	H45B	0.206(4)

4.3.2 Crystal Packing Views of (Oxa-OH)$_2$

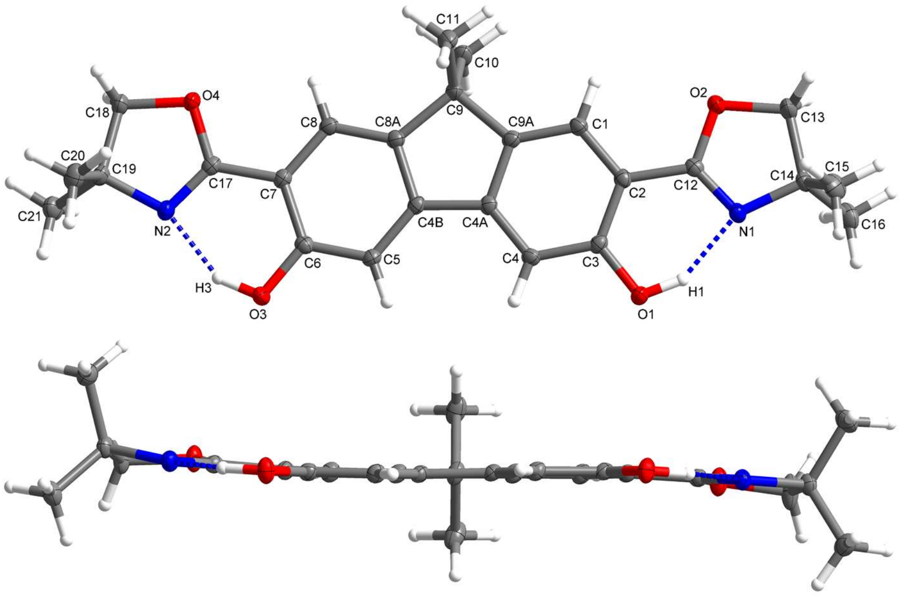

Figure S51: Top view with atom labels (top) and side view (bottom) of **(Oxa-OH)$_2$**.

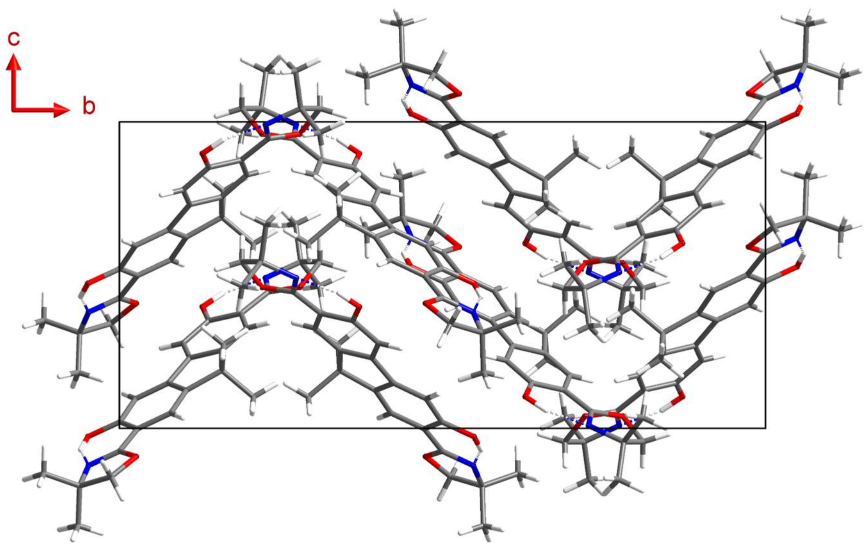

Figure S52: Unit cell packing view of an **(Oxa-OH)$_2$** crystal along the *a*-axis. For better clarity, disordered cyclohexane molecules are omitted.

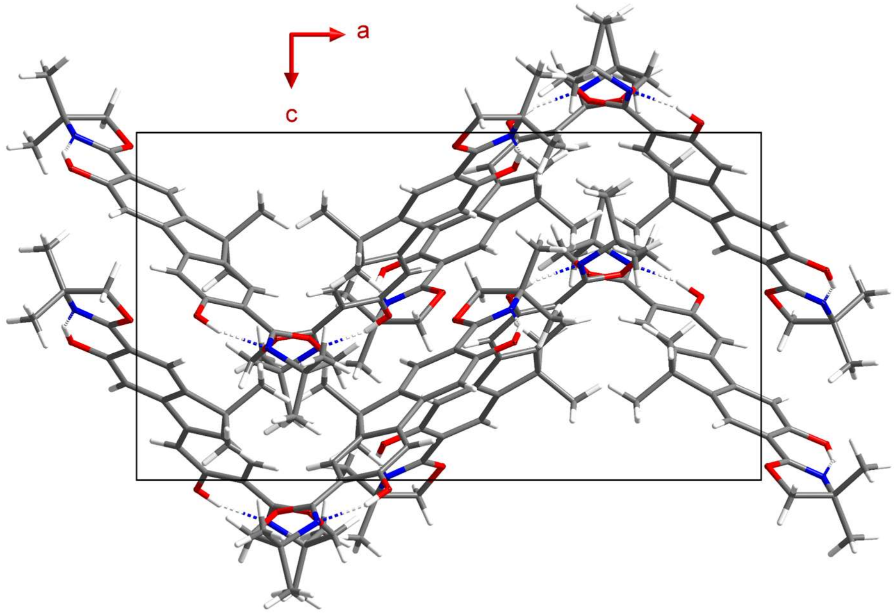

Figure S53: Unit cell packing view of an **(Oxa-OH)$_2$** crystal along the *b*-axis. For a greater clarity, disordered cyclohexane molecules are omitted.

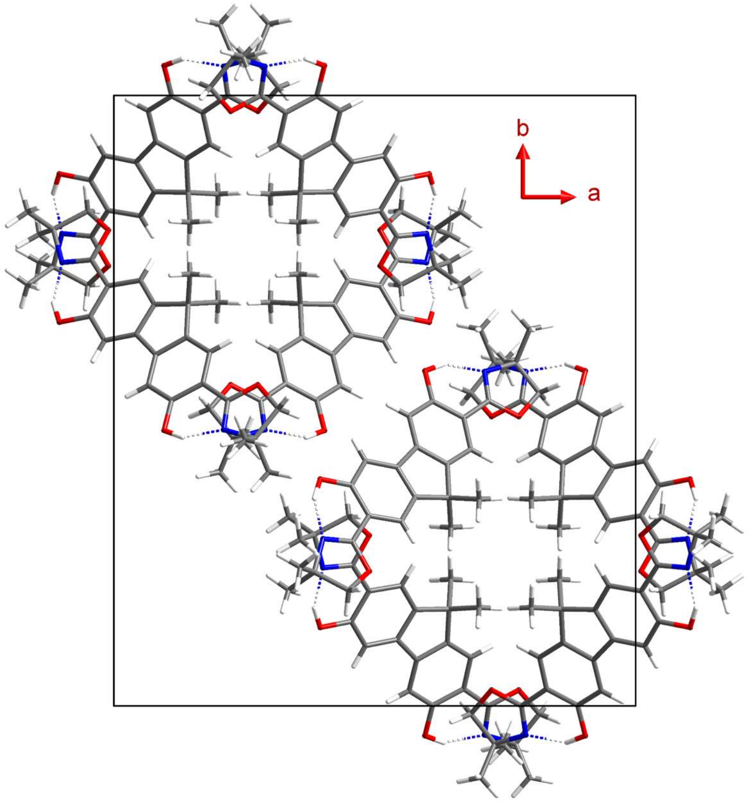

Figure S54: Unit cell packing view of an **(Oxa-OH)2** crystal along the *c*-axis. For a greater clarity, disordered cyclohexane molecules are omitted.

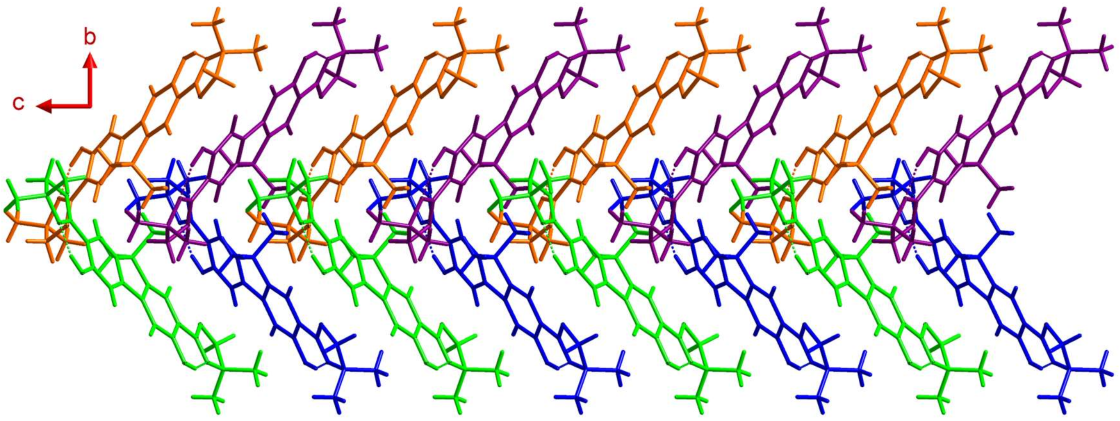

Figure S55: Crystal packing of **(Oxa-OH)2** along the *a*-axis. For a greater clarity, identical molecules are colored violet, blue, green and orange, and disordered cyclohexane molecules are omitted.

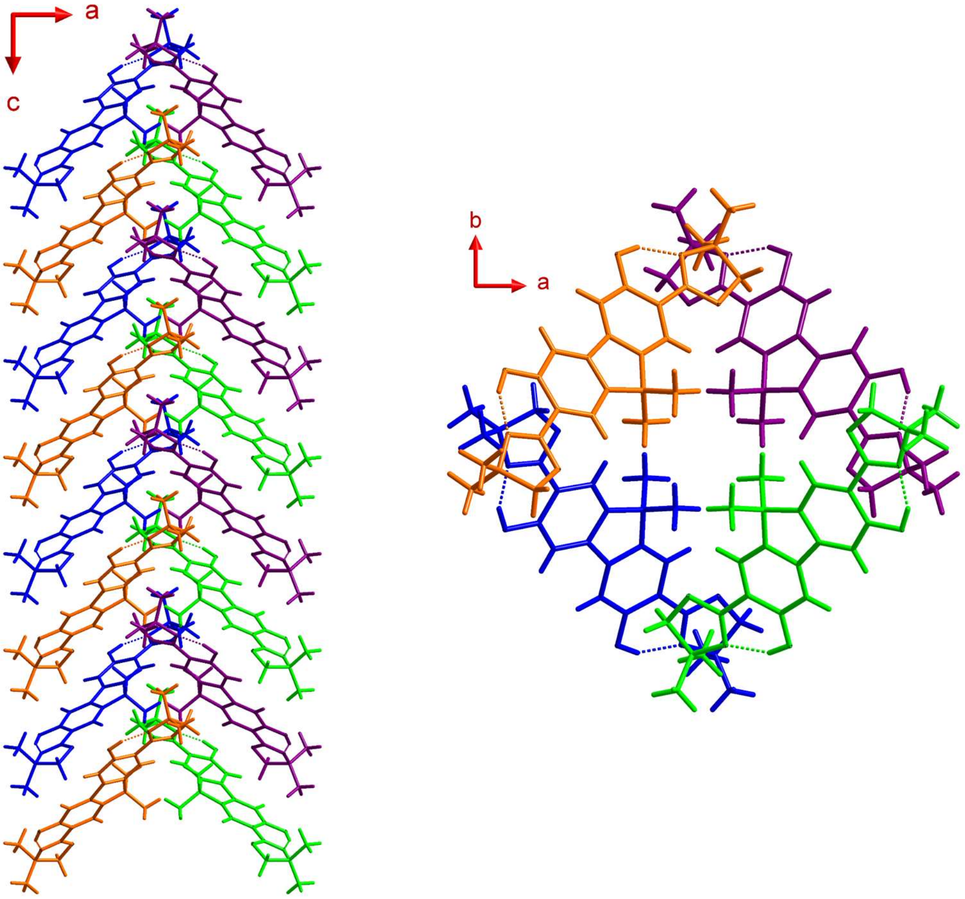

Figure S56: Crystal packing of **(Oxa-OH)₂** along the *b*-axis (left) and the *c*-axis (right). For a greater clarity, identical molecules are colored violet, blue, green and orange, and disordered cyclohexane molecules are omitted.

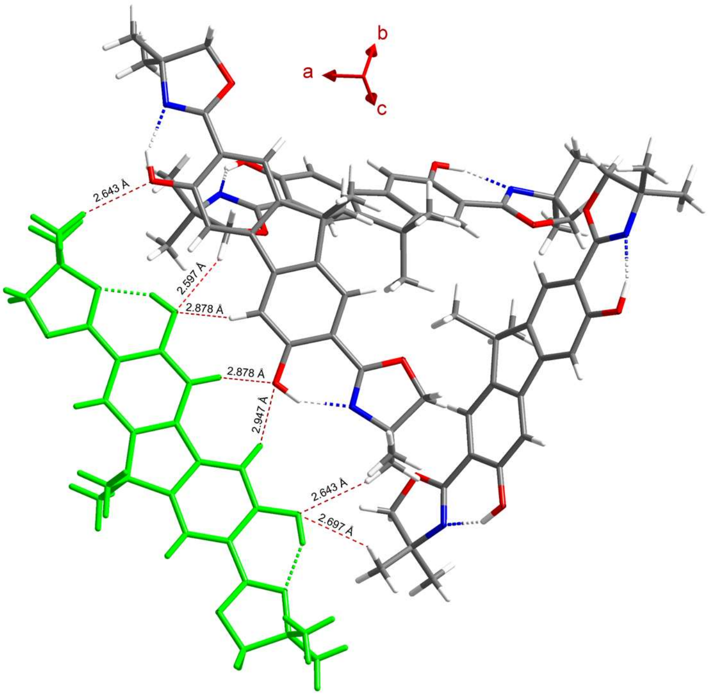

Figure S57: Various intermolecular interactions in **(Oxa-OH)₂** crystal lattice up to 3.000 Å distances, including C–H···O (red dotted lines) interactions. All distances are given in Å. For a greater clarity, one definite molecule is colored green and disordered cyclohexane molecules are omitted.

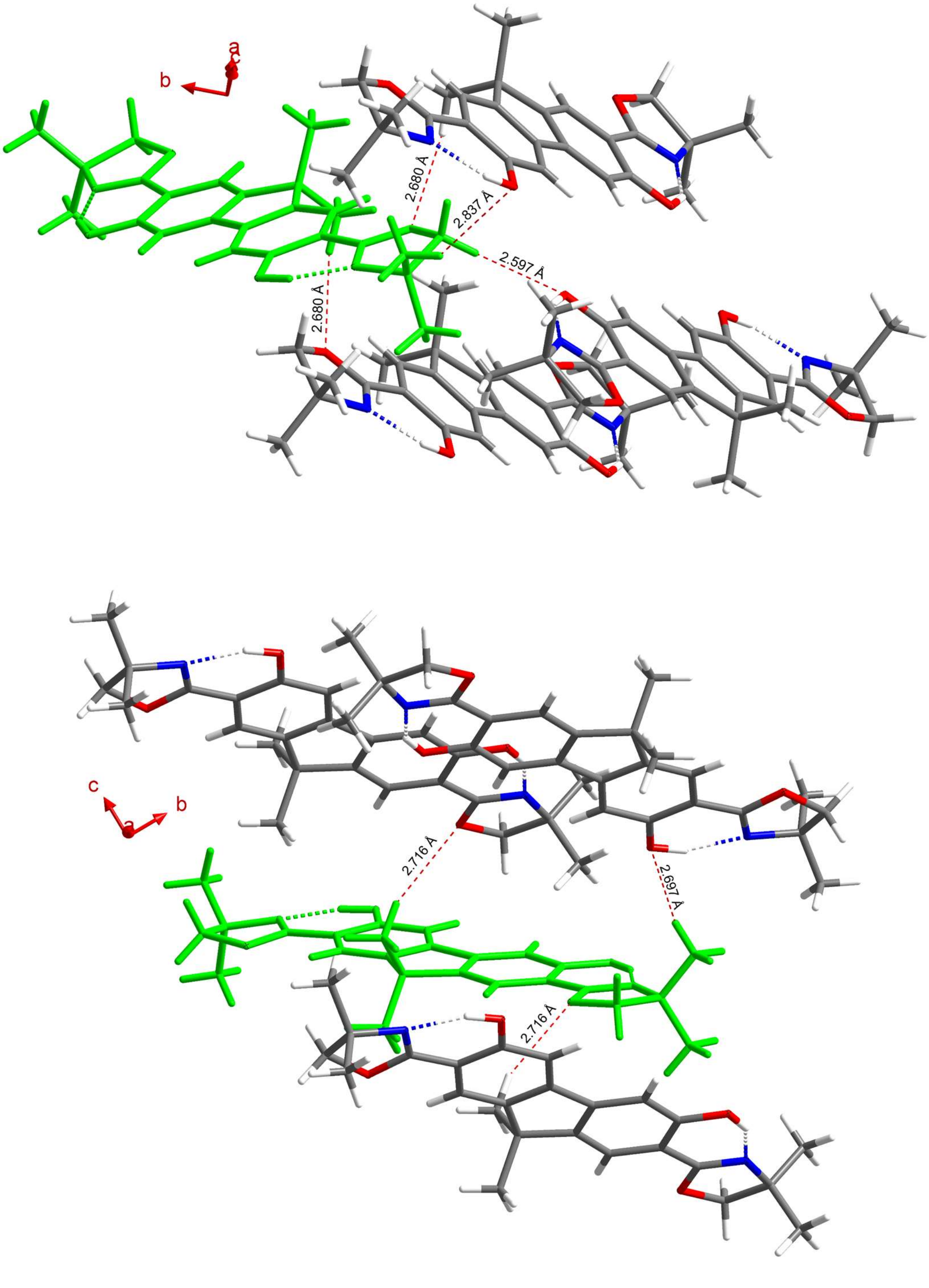

Figure S58: Various intermolecular interactions in **(Oxa-OH)₂** crystal lattice up to 3.000 Å distances, including C–H···O (red dotted lines) interactions. All distances are given in Å. For a greater clarity, one definite molecule is colored green and disordered cyclohexane molecules are omitted.

4.4 2-(Benzo[*d*]oxazol-2-yl)-7-bromo-9,9-dimethyl-9*H*-fluoren-3-ol (BO-OH)

4.4.1 Crystal Data for BO-OH

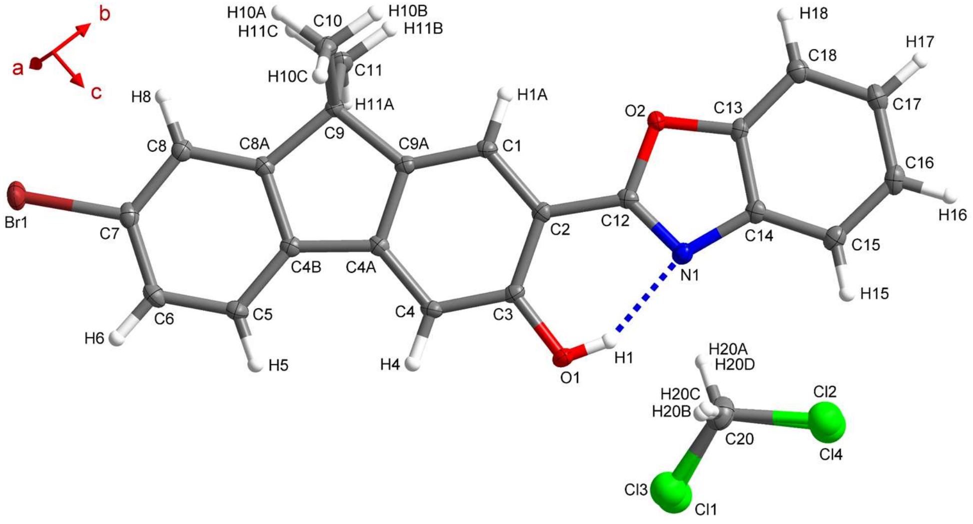

Figure S59: Asymmetric unit of **BO-OH** with atom labels.

Table S23: Crystal data and structure refinement for **BO-OH**.

Empirical formula	$C_{23}H_{18}BrCl_2NO_2$
Formula weight/g mol^{-1}	491.19
Crystal system	Triclinic
Crystal size/mm^3	0.4 × 0.23 × 0.18
Space group	P–1
a/Å	8.7249(3)
b/Å	9.7643(3)
c/Å	13.4767(5)
α/°	90.1410(10)
β/°	106.2860(10)
γ/°	112.4560(10)
Volume/Å^3	1010.68(6)
Z	2
$\rho_{calc,}$ g/cm^3	1.614
μ/mm^{-1}	2.319
F(000)	496.0

2Θ range for data collection/°	4.548 to 56.692
Index ranges	$-11 \leq h \leq 11$, $-13 \leq k \leq 12$, $-17 \leq l \leq 17$
No. of reflections collected	26422
No. of independent reflections	5026 [R_{int} = 0.0267, R_{sigma} = 0.0213]
Data/restraints/parameters	5026/0/288
Goodness-of-fit on F^2	1.029
Final R indexes [I ≥ 2σ (I)]	R_1 = 0.0247, wR_2 = 0.0539
Final R indexes [all data]	R_1 = 0.0303, wR_2 = 0.0560
Largest diff. peak/hole / e Å^{-3}	0.40/–0.33
CCDC number	2018047

Table S24: Fractional Atomic Coordinates (×10^4) and Equivalent Isotropic Displacement Parameters (Å^2×10^3) for **BO-OH**. U_{eq} is defined as 1/3 of the trace of the orthogonalized U_{IJ} tensor.

Atom	*x*	*y*	*z*	U(eq)
Br1	8673.9(2)	-2089.4(2)	263.7(2)	20.80(6)
O1	8500.0(15)	3160.7(12)	6146.7(8)	17.1(2)
O2	6941.7(13)	6274.4(11)	4446.7(7)	12.9(2)
N1	7519.9(16)	5406.6(13)	5999.8(9)	13.7(2)
C1	7469.1(17)	3998.4(15)	3415.0(11)	12.1(3)
C2	7694.1(17)	4113.2(15)	4496.3(11)	11.8(3)
C3	8247.1(18)	3111.5(15)	5106.4(10)	12.3(3)
C4	8544.8(18)	2008.8(15)	4639.0(11)	12.7(3)
C4A	8282.4(17)	1897.1(15)	3574.3(11)	11.8(3)
C4B	8466.3(17)	827.9(15)	2904.4(11)	12.0(3)
C5	8931.7(18)	-369.9(16)	3142.3(11)	14.1(3)
C6	9007.7(19)	-1236.1(16)	2350.5(12)	16.3(3)
C7	8594.0(18)	-880.6(16)	1335.8(12)	15.5(3)
C8	8120.5(18)	315.6(16)	1079.6(11)	14.2(3)
C8A	8069.7(17)	1179.9(15)	1879.8(11)	12.2(3)
C9	7605.7(18)	2538.0(15)	1820.0(10)	12.1(3)
C9A	7755.7(17)	2893.5(15)	2957.5(11)	11.8(3)

C10	5751.4(19)	2138.7(17)	1093.1(11)	16.8(3)
C11	8917(2)	3842.0(16)	1457.2(12)	17.1(3)
C12	7393.2(17)	5241.7(15)	5013.6(11)	12.3(3)
C13	6795.6(18)	7186.4(15)	5176.0(11)	13.0(3)
C14	7155.7(18)	6656.4(15)	6134.8(11)	13.5(3)
C15	7132.6(19)	7382.2(16)	7017.9(11)	16.0(3)
C16	6747.3(19)	8638.0(17)	6885.6(12)	17.7(3)
C17	6376.7(19)	9140.7(16)	5915.0(12)	17.1(3)
C18	6381.1(19)	8413.1(16)	5023.7(12)	16.2(3)
C20	5565(2)	3015(2)	7850.3(14)	28.4(4)
Cl1	7264(5)	2376(5)	8367(3)	25.9(6)
Cl2	5602(12)	4346(9)	8729(7)	30.6(8)
Cl3	6980(30)	2210(20)	8255(14)	45(2)
Cl4	5810(30)	4430(20)	8815(17)	29(2)

Table S25: Bond Lengths for **BO-OH**.

Atom	Atom	Length/Å	Atom	Atom	Length/Å
Br1	C7	1.8964(14)	C6	C7	1.394(2)
O1	C3	1.3544(17)	C7	C8	1.395(2)
O2	C12	1.3763(16)	C8	C8A	1.3885(19)
O2	C13	1.3884(16)	C8A	C9	1.5236(19)
N1	C12	1.3067(18)	C9	C9A	1.5289(19)
N1	C14	1.3968(18)	C9	C10	1.5351(19)
C1	C2	1.4129(19)	C9	C11	1.540(2)
C1	C9A	1.3769(19)	C13	C14	1.391(2)
C2	C3	1.4169(19)	C13	C18	1.378(2)
C2	C12	1.4454(19)	C14	C15	1.393(2)
C3	C4	1.3877(19)	C15	C16	1.390(2)
C4	C4A	1.3853(19)	C16	C17	1.394(2)
C4A	C4B	1.4611(19)	C17	C18	1.396(2)
C4A	C9A	1.4072(19)	C20	Cl1	1.790(4)
C4B	C5	1.3882(19)	C20	Cl2	1.740(8)

C4B	C8A	1.4035(19)	C20	Cl3	1.678(16)
C5	C6	1.392(2)	C20	Cl4	1.803(18)

Table S26: Bond Angles for **BO-OH**.

Atom	Atom	Atom	Angle/°	Atom	Atom	Atom	Angle/°
C12	O2	C13	103.96(11)	C8	C8A	C4B	120.09(13)
C12	N1	C14	105.24(12)	C8	C8A	C9	128.64(13)
C9A	C1	C2	119.71(13)	C8A	C9	C9A	100.97(11)
C1	C2	C3	119.86(12)	C8A	C9	C10	110.83(11)
C1	C2	C12	121.97(12)	C8A	C9	C11	111.36(11)
C3	C2	C12	118.16(12)	C9A	C9	C10	112.04(11)
CL2a	C20	Cl1	112.0(4)	C9A	C9	C11	111.19(11)
CL3b	C20	Cl4	113.0(9)	C10	C9	C11	110.18(12)
O1	C3	C2	123.12(13)	C1	C9A	C4A	119.57(13)
O1	C3	C4	116.92(12)	C1	C9A	C9	129.76(12)
C4	C3	C2	119.96(12)	C4A	C9A	C9	110.66(12)
C4A	C4	C3	119.23(13)	O2	C12	C2	119.18(12)
C4	C4A	C4B	129.61(13)	N1	C12	O2	114.56(12)
C4	C4A	C9A	121.64(13)	N1	C12	C2	126.26(13)
C9A	C4A	C4B	108.75(12)	O2	C13	C14	107.94(12)
C5	C4B	C4A	130.48(13)	C18	C13	O2	128.18(13)
C5	C4B	C8A	121.19(13)	C18	C13	C14	123.89(13)
C8A	C4B	C4A	108.33(12)	C13	C14	N1	108.30(12)
C4B	C5	C6	119.39(13)	C13	C14	C15	120.29(13)
C5	C6	C7	118.71(13)	C15	C14	N1	131.40(14)
C6	C7	Br1	118.15(11)	C16	C15	C14	116.78(14)
C6	C7	C8	122.82(13)	C15	C16	C17	121.88(14)
C8	C7	Br1	119.03(11)	C16	C17	C18	121.76(14)
C8A	C8	C7	117.79(13)	C13	C18	C17	115.38(14)
C4B	C8A	C9	111.26(12)				

Table S27: Torsion Angles for **BO-OH**.

A	B	C	D	Angle/°	A	B	C	D	Angle/°
Br1	C7	C8	C8A	179.88(10)	C6	C7	C8	C8A	0.2(2)
O1	C3	C4	C4A	179.13(12)	C7	C8	C8A	C4B	-0.8(2)
O2	C13	C14	N1	-0.24(15)	C7	C8	C8A	C9	179.82(13)
O2	C13	C14	C15	178.91(12)	C8	C8A	C9	C9A	178.28(14)
O2	C13	C18	C17	-178.34(13)	C8	C8A	C9	C10	59.41(19)
N1	C14	C15	C16	178.61(14)	C8	C8A	C9	C11	-63.61(18)
C1	C2	C3	O1	179.60(13)	C8A	C4B	C5	C6	0.1(2)
C1	C2	C3	C4	-0.8(2)	C8A	C9	C9A	C1	-178.94(14)
C1	C2	C12	O2	-2.7(2)	C8A	C9	C9A	C4A	1.55(14)
C1	C2	C12	N1	177.95(13)	C9A	C1	C2	C3	1.3(2)
C2	C1	C9A	C4A	-0.5(2)	C9A	C1	C2	C12	-179.34(12)
C2	C1	C9A	C9	-179.94(13)	C9A	C4A	C4B	C5	-179.12(14)
C2	C3	C4	C4A	-0.5(2)	C9A	C4A	C4B	C8A	0.61(15)
C3	C2	C12	O2	176.71(12)	C10	C9	C9A	C1	-60.96(19)
C3	C2	C12	N1	-2.7(2)	C10	C9	C9A	C4A	119.53(13)
C3	C4	C4A	C4B	-178.54(13)	C11	C9	C9A	C1	62.83(19)
C3	C4	C4A	C9A	1.3(2)	C11	C9	C9A	C4A	-116.68(13)
C4	C4A	C4B	C5	0.8(2)	C12	O2	C13	C14	-0.26(14)
C4	C4A	C4B	C8A	-179.51(14)	C12	O2	C13	C18	179.46(14)
C4	C4A	C9A	C1	-0.9(2)	C12	N1	C14	C13	0.67(15)
C4	C4A	C9A	C9	178.70(12)	C12	N1	C14	C15	-178.35(15)
C4A	C4B	C5	C6	179.75(14)	C12	C2	C3	O1	0.2(2)
C4A	C4B	C8A	C8	-179.08(12)	C12	C2	C3	C4	179.79(12)
C4A	C4B	C8A	C9	0.43(15)	C13	O2	C12	N1	0.75(15)
C4B	C4A	C9A	C1	179.03(12)	C13	O2	C12	C2	-178.71(12)
C4B	C4A	C9A	C9	-1.40(15)	C13	C14	C15	C16	-0.3(2)
C4B	C5	C6	C7	-0.7(2)	C14	N1	C12	O2	-0.90(16)
C4B	C8A	C9	C9A	-1.18(14)	C14	N1	C12	C2	178.52(13)
C4B	C8A	C9	C10	-120.04(13)	C14	C13	C18	C17	1.3(2)
C4B	C8A	C9	C11	116.93(13)	C14	C15	C16	C17	0.9(2)

C5	C4B	C8A	C8	0.7(2)	C15	C16	C17	C18	-0.3(2)
C5	C4B	C8A	C9	-179.81(12)	C16	C17	C18	C13	-0.8(2)
C5	C6	C7	Br1	-179.16(11)	C18	C13	C14	N1	-179.98(13)
C5	C6	C7	C8	0.6(2)	C18	C13	C14	C15	-0.8(2)

Table S28: Hydrogen Atom Coordinates (Å×10^4) and Isotropic Displacement Parameters (Å^2×10^3) for **BO-OH**.

Atom	*x*	*y*	*z*	U(eq)
H1	8340(30)	3870(30)	6330(17)	33(6)
H1A	7121.57	4680.59	3005.45	15
H4	8924.16	1338.37	5044.38	15
H5	9195.92	-596.07	3839.23	17
H6	9335.18	-2054.05	2498.86	20
H8	7842.02	532.09	380.79	17
H10A	5701.25	1884.98	377.46	25
H10B	5433.95	2994.76	1124.33	25
H10C	4935.3	1281.54	1313.58	25
H11A	10097.16	4074.17	1918.2	26
H11B	8639.91	4722.19	1479.9	26
H11C	8846.69	3559.25	742.51	26
H15	7368.94	7035.5	7678.94	19
H16	6736.09	9169.15	7473.27	21
H17	6114.08	10001.6	5858.75	21
H18	6116.45	8741.1	4358	19
H20A	5694.25	3452.22	7201.27	34
H20B	4424.76	2154.68	7672.98	34
H20C	4365.83	2236.85	7666.94	34
H20D	5726.86	3472.95	7212.51	34

Table S29: Atomic Occupancy for **BO-OH**.

Atom	*Occupancy*	Atom	*Occupancy*	Atom	*Occupancy*
H20A	0.71(4)	H20B	0.71(4)	H20C	0.29(4)
H20D	0.29(4)	Cl1	0.71(4)	Cl2	0.71(4)
Cl3	0.29(4)	Cl4	0.29(4)		

4.4.2 Crystal Packing Views of BO-OH

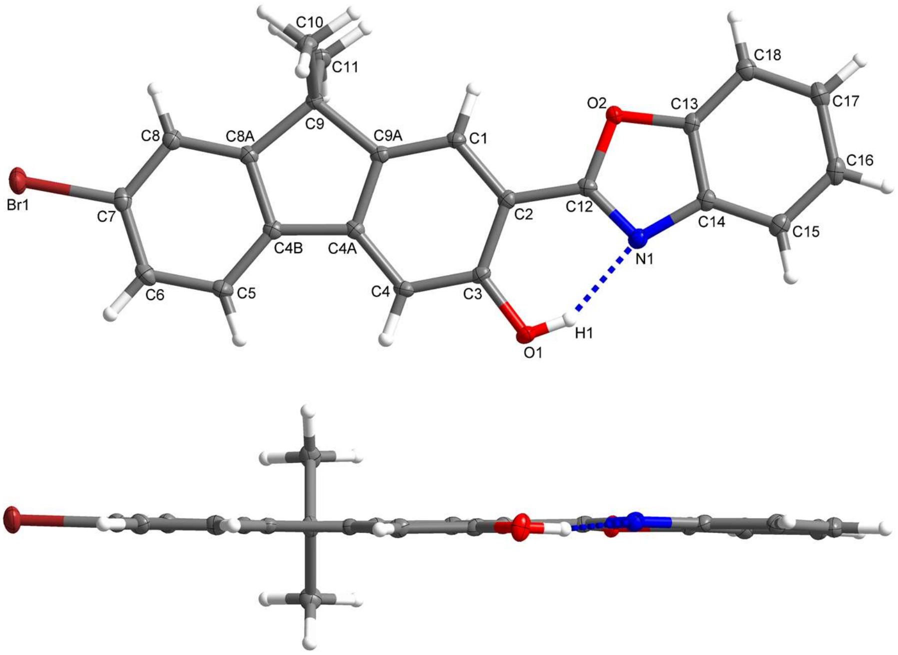

Figure S60: Top view with atom labels (top) and side view (bottom) of **BO-OH**.

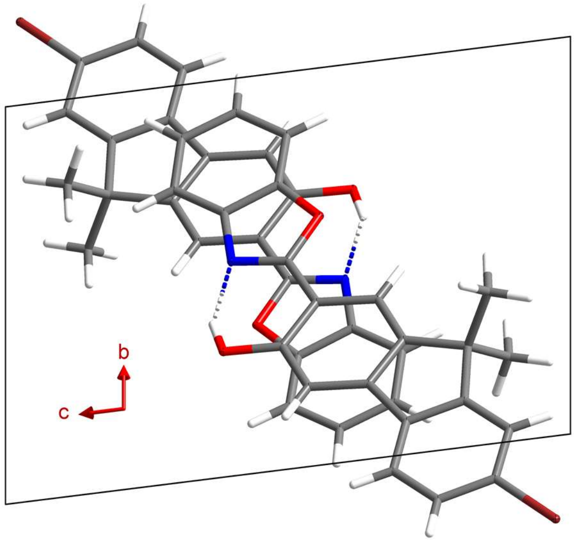

Figure S61: Unit cell packing view of a **BO-OH** crystal along the *a*-axis. For a greater clarity, disordered CH_2Cl_2 molecules are omitted.

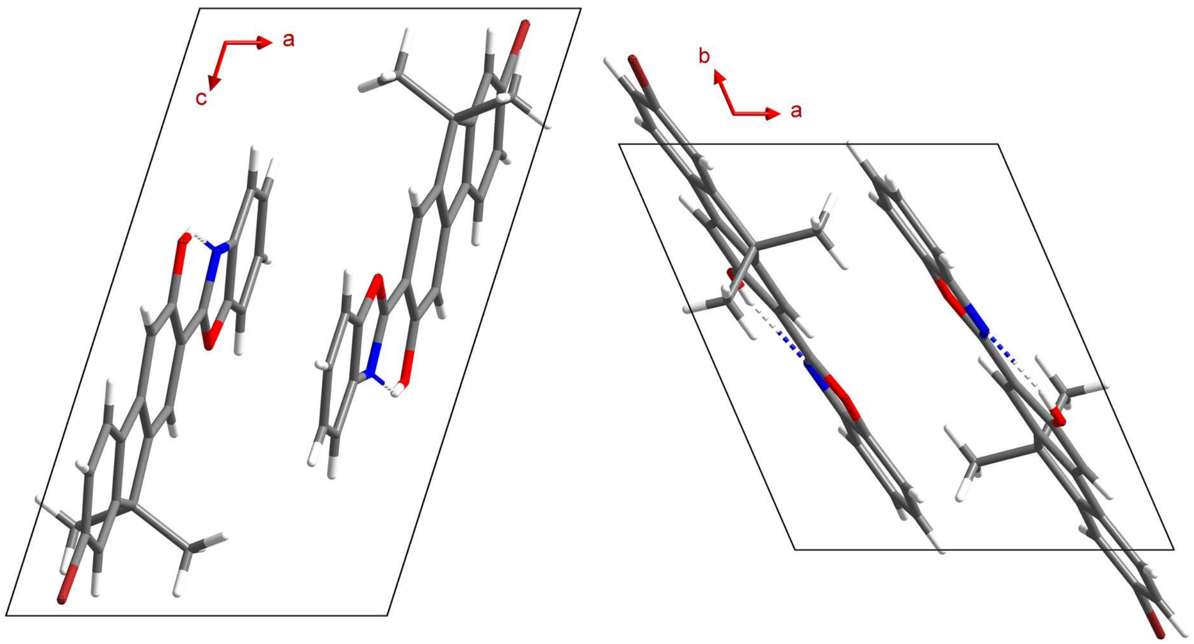

Figure S62: Unit cell packing view of a **BO-OH** crystal along the *b*-axis (left) and the *c*-axis (right). For a greater clarity, disordered CH_2Cl_2 molecules are omitted.

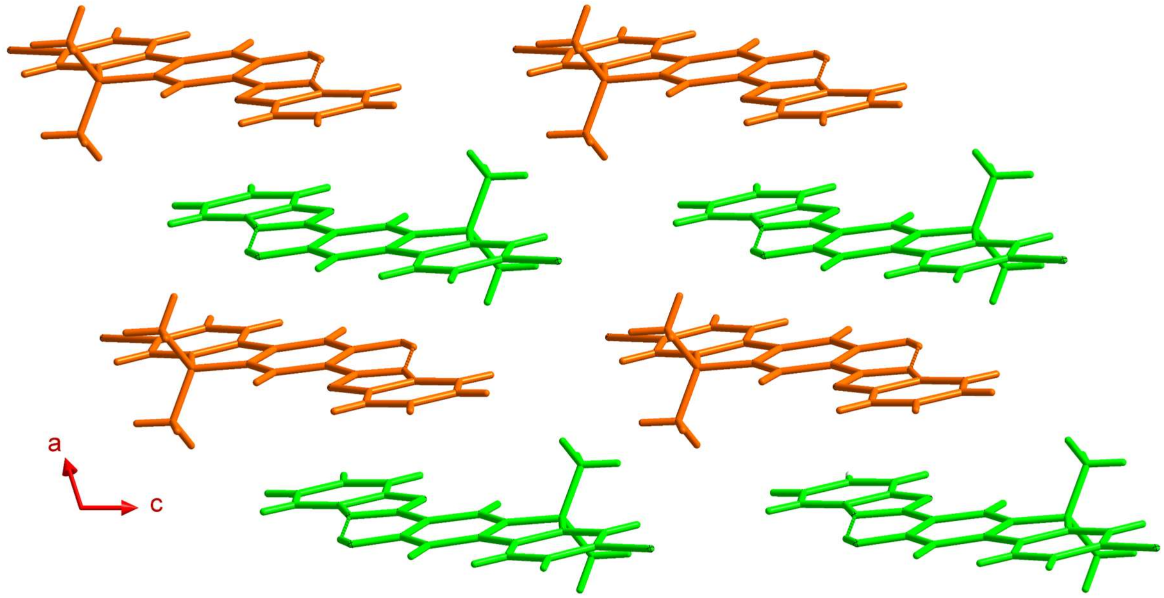

Figure S63: Crystal packing of **BO-OH** along the *b*-axis. For a greater clarity, identical molecules are colored green and orange, and disordered CH_2Cl_2 molecules are omitted.

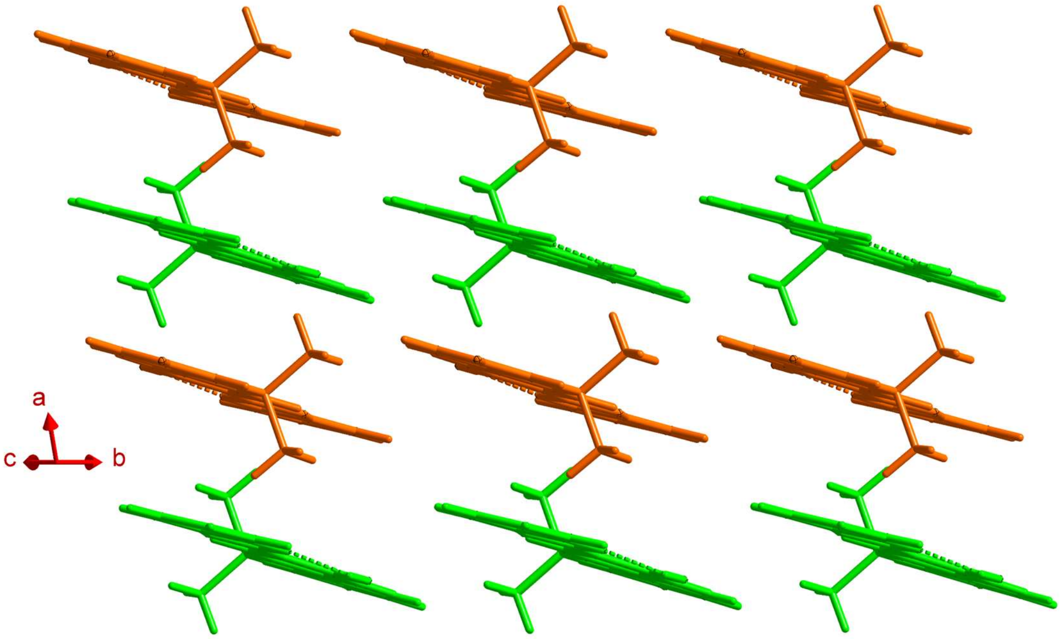

Figure S64: Crystal packing of **BO-OH**. For a greater clarity, identical molecules are colored green and orange, and disordered CH_2Cl_2 molecules are omitted.

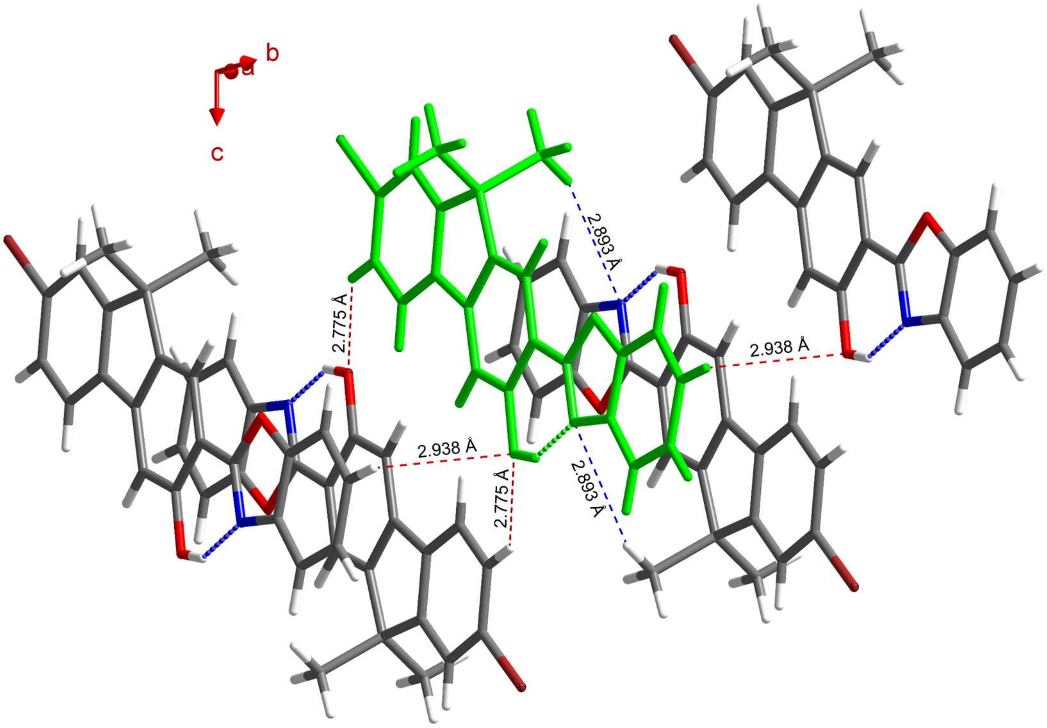

Figure S65: Various intermolecular interactions in **BO-OH** crystal lattice up to 3.000 Å distances, including C–H···O (red dashed lines) and C–H···N (blue dashed lines) interactions. All distances are given in Å. For a greater clarity, one definite molecule is colored green and disordered CH_2Cl_2 molecules are omitted.

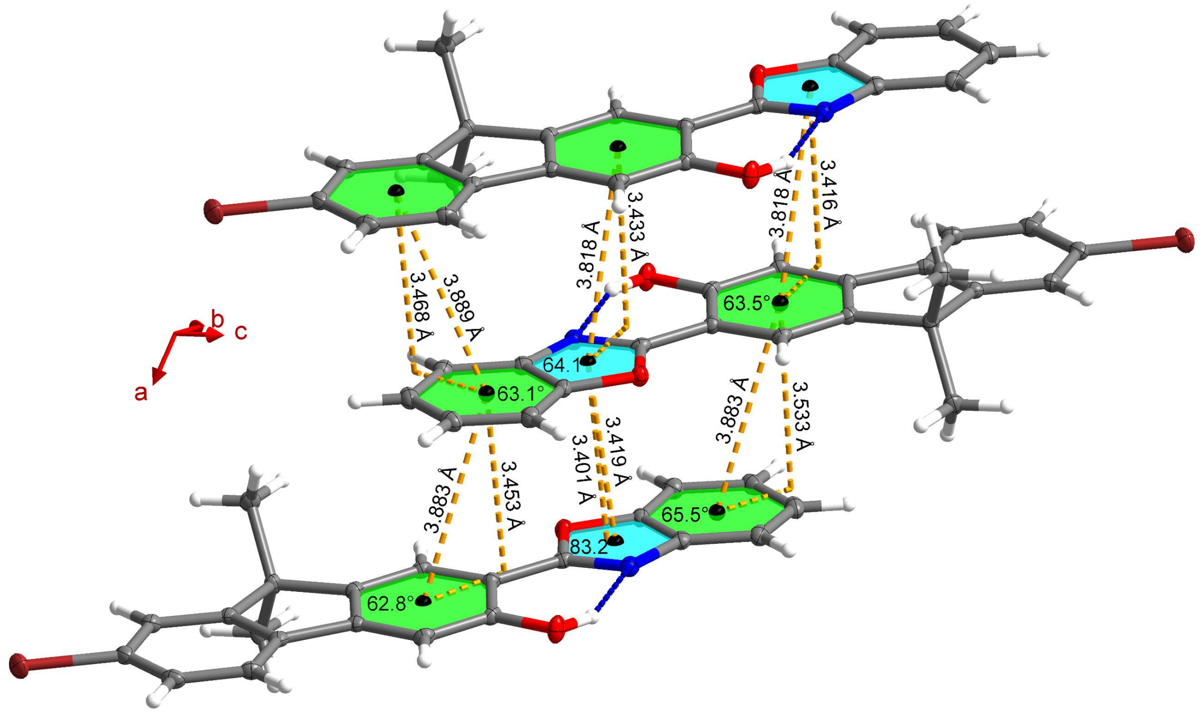

Figure S66: Intermolecular packing of three adjacent molecules in **BO-OH** crystal lattice with plane distance (orange dashed line) and centroid-centroid distance (orange dashed line between black spheres) with the corresponding slip angle. All distances are given in Å. The slip angle is given in °. For a greater clarity, disordered CH_2Cl_2 molecules are omitted.

4.5 7-Bromo-9,9-dimethyl-2-(naphtho[2,3-*d*]oxazol-2-yl)-9*H*-fluoren-3-ol (NO-OH)

4.5.1 Crystal Data for NO-OH

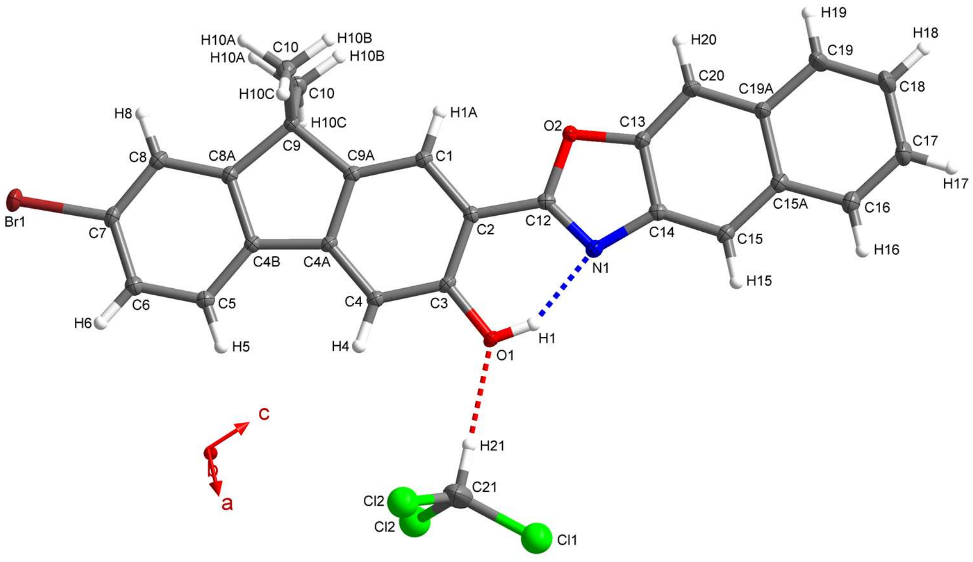

Figure S67: Asymmetric unit of **NO-OH** with atom labels.

Table S30: Crystal data and structure refinement for **NO-OH**.

Empirical formula	$C_{27}H_{19}BrCl_3NO_2$
Formula weight/g mol^{-1}	575.69
Crystal system	Monoclinic
Crystal size/mm^3	0.37 × 0.13 × 0.11
Space group	$P2_1/m$
a/Å	12.6799(6)
b/Å	6.6733(3)
c/Å	15.0742(7)
α/°	90
β/°	109.160(2)
γ/°	90
Volume/Å^3	1204.87(10)
Z	2
$\rho_{calc,}$ g/cm^3	1.587
μ/mm^{-1}	2.065
F(000)	580.0
2Θ range for data collection/°	5.112 to 56.56
Index ranges	$-16 \le h \le 16$, $-8 \le k \le 8$, $-20 \le l \le 20$
No. of reflections collected	33721
No. of independent reflections	3230 [R_{int} = 0.0428, R_{sigma} = 0.0208]
Data/restraints/parameters	3230/3/201
Goodness-of-fit on F^2	1.119
Final R indexes [$I \ge 2\sigma$ (I)]	R_1 = 0.0448, wR_2 = 0.1172
Final R indexes [all data]	R_1 = 0.0512, wR_2 = 0.1209
Largest diff. peak/hole / e Å^{-3}	0.74/–1.60
CCDC number	2018051

Table S31: Fractional Atomic Coordinates (×10^4) and Equivalent Isotropic Displacement Parameters (Å^2×10^3) for **NO-OH**. U_{eq} is defined as 1/3 of the trace of the orthogonalized U_{IJ} tensor.

Atom	*x*	*y*	*z*	U(eq)
Br1	495.5(3)	7500	-878.5(2)	22.19(13)
O1	6500.7(19)	7500	4910.8(17)	16.9(5)
O2	4405(2)	7500	6559.0(16)	16.8(5)
N1	6167(2)	7500	6524(2)	13.8(6)
C1	3477(3)	7500	4574(2)	12.6(6)
C2	4643(3)	7500	5027(2)	11.7(6)
C3	5372(3)	7500	4497(2)	12.2(6)
C4	4958(3)	7500	3524(2)	12.7(6)
C4A	3807(3)	7500	3083(2)	12.1(6)
C4B	3152(3)	7500	2089(2)	11.9(6)
C5	3495(3)	7500	1303(2)	14.4(6)
C6	2691(3)	7500	421(2)	15.5(7)
C7	1567(3)	7500	347(2)	15.1(7)
C8	1208(3)	7500	1121(2)	15.5(7)
C8A	2018(3)	7500	2002(2)	12.8(6)
C9	1859(3)	7500	2963(2)	13.3(6)
C9A	3066(3)	7500	3603(2)	11.6(6)
C10	1239(2)	5621(4)	3104.8(17)	18.3(5)
C12	5109(3)	7500	6040(2)	12.4(6)
C13	5115(3)	7500	7481(2)	16.9(7)
C14	6217(3)	7500	7463(2)	12.2(6)
C15	7100(3)	7500	8283(2)	14.3(6)
C15A	6876(3)	7500	9136(2)	14.9(7)
C16	7757(3)	7500	10008(2)	18.4(7)
C17	7551(3)	7500	10842(3)	28.4(9)
C18	6446(4)	7500	10842(3)	60(2)
C19	5573(4)	7500	10017(3)	60(2)
C19A	5754(3)	7500	9138(3)	26.3(9)
C20	4843(3)	7500	8280(3)	30.7(10)

Cl1	9588.3(15)	7500	4890.1(12)	93.2(9)
Cl2	8129.2(11)	5372(3)	3306.2(10)	81.2(4)
C21	8338(5)	7500	3995(4)	64(2)

Table S32: Bond Lengths for **NO-OH**.

Atom	Atom	Length/Å	Atom	Atom	Length/Å
Br1	C7	1.900(3)	C8	C8A	1.387(5)
O1	C3	1.361(4)	C8A	C9	1.526(5)
O2	C12	1.366(4)	C9	C9A	1.518(4)
O2	C13	1.386(4)	C9	C10[1]	1.532(3)
N1	C12	1.300(4)	C9	C10	1.532(3)
N1	C14	1.397(4)	C13	C14	1.406(5)
C1	C2	1.411(4)	C13	C20	1.357(5)
C1	C9A	1.384(4)	C14	C15	1.367(5)
C2	C3	1.406(4)	C15	C15A	1.406(5)
C2	C12	1.446(4)	C15A	C16	1.418(5)
C3	C4	1.386(5)	C15A	C19A	1.424(5)
C4	C4A	1.391(4)	C16	C17	1.366(5)
C4A	C4B	1.455(4)	C17	C18	1.401(6)
C4A	C9A	1.408(4)	C18	C19	1.367(6)
C4B	C5	1.390(5)	C19	C19A	1.417(5)
C4B	C8A	1.401(4)	C19A	C20	1.423(5)
C5	C6	1.385(5)	Cl1	C21	1.712(6)
C6	C7	1.393(5)	Cl2	C21	1.727(4)
C7	C8	1.385(5)	C21	Cl2[1]	1.727(4)

[1]+X,3/2-Y,+Z

Table S33: Bond Angles for **NO-OH**.

Atom	Atom	Atom	Angle/°	Atom	Atom	Atom	Angle/°
C12	O2	C13	104.0(3)	C9A	C9	C10[1]	111.60(18)
C12	N1	C14	105.3(3)	C10	C9	C10[1]	109.9(3)
C9A	C1	C2	118.8(3)	C1	C9A	C4A	120.1(3)

C1	C2	C12	120.7(3)	C1	C9A	C9	128.6(3)
C3	C2	C1	120.4(3)	C4A	C9A	C9	111.3(3)
C3	C2	C12	118.9(3)	O2	C12	C2	119.2(3)
O1	C3	C2	121.9(3)	N1	C12	O2	115.3(3)
O1	C3	C4	117.5(3)	N1	C12	C2	125.5(3)
C4	C3	C2	120.7(3)	O2	C13	C14	107.7(3)
C3	C4	C4A	118.6(3)	C20	C13	O2	128.3(3)
C4	C4A	C4B	130.3(3)	C20	C13	C14	124.1(3)
C4	C4A	C9A	121.4(3)	N1	C14	C13	107.7(3)
C9A	C4A	C4B	108.3(3)	C15	C14	N1	131.9(3)
C5	C4B	C4A	130.1(3)	C15	C14	C13	120.4(3)
C5	C4B	C8A	121.3(3)	C14	C15	C15A	118.4(3)
C8A	C4B	C4A	108.5(3)	C15	C15A	C16	121.0(3)
C6	C5	C4B	118.7(3)	C15	C15A	C19A	120.2(3)
C5	C6	C7	119.3(3)	C16	C15A	C19A	118.8(3)
C6	C7	Br1	117.7(3)	C17	C16	C15A	121.6(3)
C8	C7	Br1	119.4(3)	C16	C17	C18	119.5(4)
C8	C7	C6	122.9(3)	C19	C18	C17	120.8(4)
C7	C8	C8A	117.5(3)	C18	C19	C19A	121.3(4)
C4B	C8A	C9	111.3(3)	C19	C19A	C15A	118.1(3)
C8	C8A	C4B	120.3(3)	C19	C19A	C20	121.2(4)
C8	C8A	C9	128.5(3)	C20	C19A	C15A	120.7(3)
C8A	C9	C10[1]	111.45(18)	C13	C20	C19A	116.1(3)
C8A	C9	C10	111.45(18)	Cl1	C21	Cl2	113.2(2)
C9A	C9	C8A	100.6(3)	Cl1	C21	Cl2[1]	113.2(2)
C9A	C9	C10	111.59(18)	Cl2	C21	Cl2[1]	110.6(3)

[1]+X,3/2-Y,+Z

Table S34: Torsion Angles for **NO-OH**.

**A**	**B**	**C**	**D**	**Angle/°**	**A**	**B**	**C**	**D**	**Angle/°**
Br1	C7	C8	C8A	180.000(0)	C8A	C4B	C5	C6	0.000(0)
O1	C3	C4	C4A	180.000(1)	C8A	C9	C9A	C1	180.000(1)

O2	C13	C14	N1	0.000(1)	C8A	C9	C9A	C4A	0.000(1)
O2	C13	C14	C15	180.000(1)	C9A	C1	C2	C3	0.000(1)
O2	C13	C20	C19A	180.000(2)	C9A	C1	C2	C12	180.000(1)
N1	C14	C15	C15A	180.000(2)	C9A	C4A	C4B	C5	180.000(1)
C1	C2	C3	O1	180.000(1)	C9A	C4A	C4B	C8A	0.000(1)
C1	C2	C3	C4	0.000(1)	C10	C9	C9A	C1	-61.7(2)
C1	C2	C12	O2	0.000(1)	C10[1]	C9	C9A	C1	61.7(2)
C1	C2	C12	N1	180.000(1)	C10[1]	C9	C9A	C4A	-118.3(2)
C2	C1	C9A	C4A	0.000(1)	C10	C9	C9A	C4A	118.3(2)
C2	C1	C9A	C9	180.000(1)	C12	O2	C13	C14	0.000(1)
C2	C3	C4	C4A	0.000(1)	C12	O2	C13	C20	180.000(2)
C3	C2	C12	O2	180.000(1)	C12	N1	C14	C13	0.000(1)
C3	C2	C12	N1	0.000(1)	C12	N1	C14	C15	180.000(2)
C3	C4	C4A	C4B	180.000(1)	C12	C2	C3	O1	0.000(1)
C3	C4	C4A	C9A	0.000(1)	C12	C2	C3	C4	180.000(1)
C4	C4A	C4B	C5	0.000(1)	C13	O2	C12	N1	0.000(1)
C4	C4A	C4B	C8A	180.000(1)	C13	O2	C12	C2	180.000(1)
C4	C4A	C9A	C1	0.000(1)	C13	C14	C15	C15A	0.000(2)
C4	C4A	C9A	C9	180.000(1)	C14	N1	C12	O2	0.000(1)
C4A	C4B	C5	C6	180.000(0)	C14	N1	C12	C2	180.000(1)
C4A	C4B	C8A	C8	180.000(0)	C14	C13	C20	C19A	0.000(2)
C4A	C4B	C8A	C9	0.000(0)	C14	C15	C15A	C16	180.000(2)
C4B	C4A	C9A	C1	180.000(1)	C14	C15	C15A	C19A	0.000(2)
C4B	C4A	C9A	C9	0.000(1)	C15	C15A	C16	C17	180.000(2)
C4B	C5	C6	C7	0.000(0)	C15	C15A	C19A	C19	180.000(2)
C4B	C8A	C9	C9A	0.000(1)	C15	C15A	C19A	C20	0.000(2)
C4B	C8A	C9	C10[1]	118.41(19)	C15A	C16	C17	C18	0.000(3)
C4B	C8A	C9	C10	-118.40(19)	C15A	C19A	C20	C13	0.000(2)
C5	C4B	C8A	C8	0.000(0)	C16	C15A	C19A	C19	0.000(3)
C5	C4B	C8A	C9	180.000(0)	C16	C15A	C19A	C20	180.000(2)
C5	C6	C7	Br1	180.000(0)	C16	C17	C18	C19	0.000(3)
C5	C6	C7	C8	0.000(0)	C17	C18	C19	C19A	0.000(3)

C6	C7	C8	C8A	0.000(0)	C18	C19	C19A	C15A	0.000(3)
C7	C8	C8A	C4B	0.000(0)	C18	C19	C19A	C20	180.000(2)
C7	C8	C8A	C9	180.000(0)	C19	C19A	C20	C13	180.000(2)
C8	C8A	C9	C9A	180.000(0)	C19A	C15A	C16	C17	0.000(2)
C8	C8A	C9	C10[1]	-61.59(19)	C20	C13	C14	N1	180.000(1)
C8	C8A	C9	C10	61.60(19)	C20	C13	C14	C15	0.000(2)

[1]+X,3/2-Y,+Z

Table S35: Hydrogen Atom Coordinates (Å×10^4) and Isotropic Displacement Parameters (Å^2×10^3) for **NO-OH**.

Atom	*x*	*y*	*z*	U(eq)
H1	6657.9	7500	5497.26	25
H1A	2981.85	7500	4930.52	15
H4	5450.18	7500	3165.53	15
H5	4266.72	7500	1369.63	17
H6	2903.87	7500	-127.14	19
H8	435.28	7500	1051.08	19
H10A	482.43	5616.37	2648.16	27
H10B	1197.06	5618.53	3742.48	27
H10C	1640.48	4424.82	3014.2	27
H15	7845.86	7500	8275.01	17
H16	8507.21	7500	10011.84	22
H17	8153.23	7500	11419.03	34
H18	6301.68	7500	11421.68	72
H19	4830.11	7500	10033.34	71
H20	4089.15	7500	8266.91	37
H21	7743.33	7499.99	4296.26	76

4.5.2 Crystal Packing Views of NO-OH

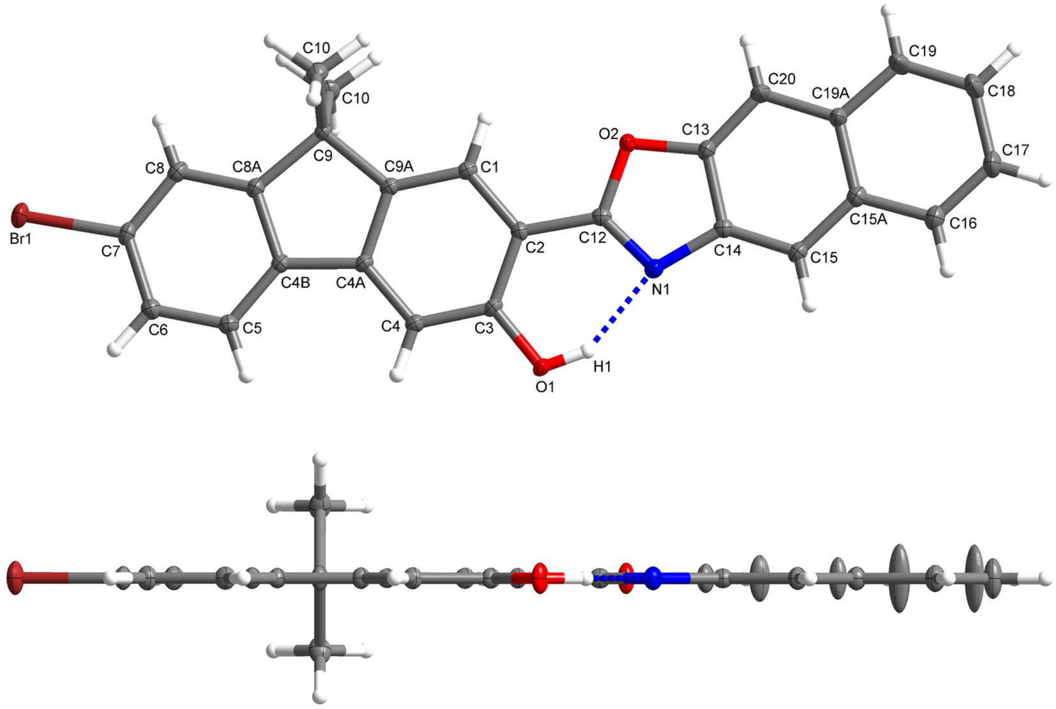

Figure S68: Top view with atom labels and coordinated $CHCl_3$ molecule (top) and side view (bottom) of **NO-OH**.

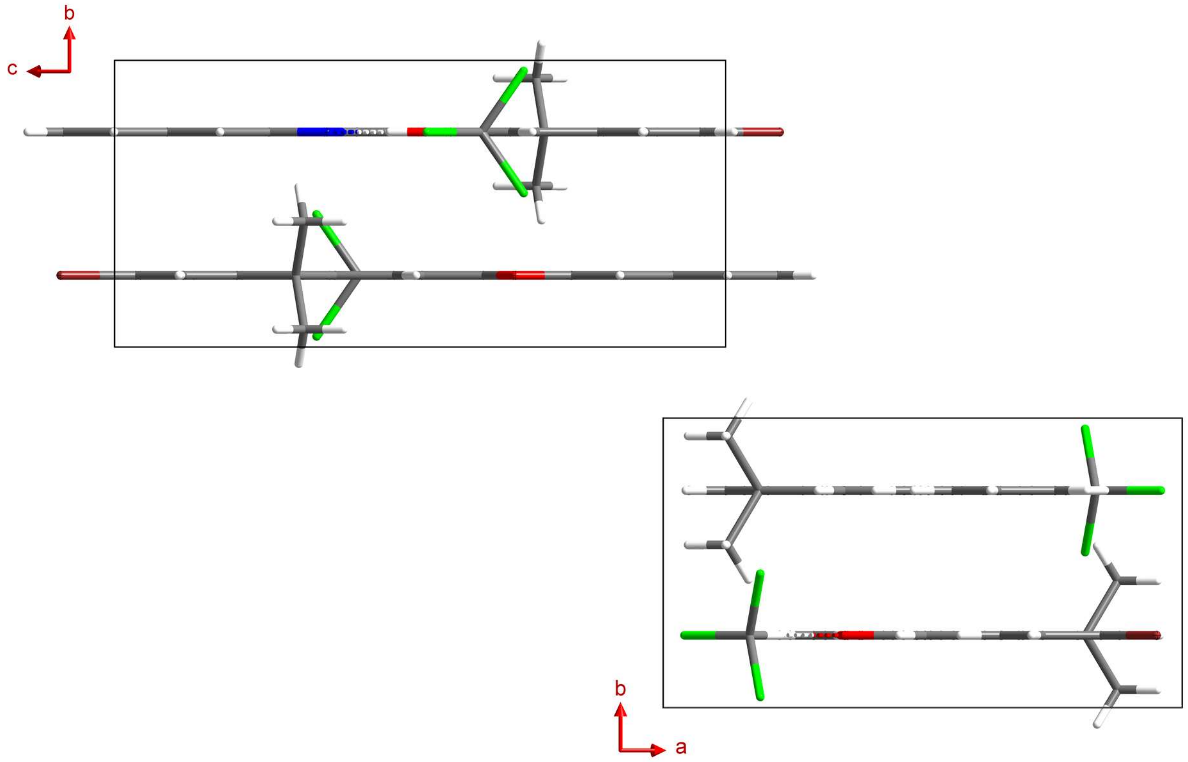

Figure S69: Unit cell packing view of a **NO-OH** crystal along the *a*-axis (top) and the *c*-axis (bottom).

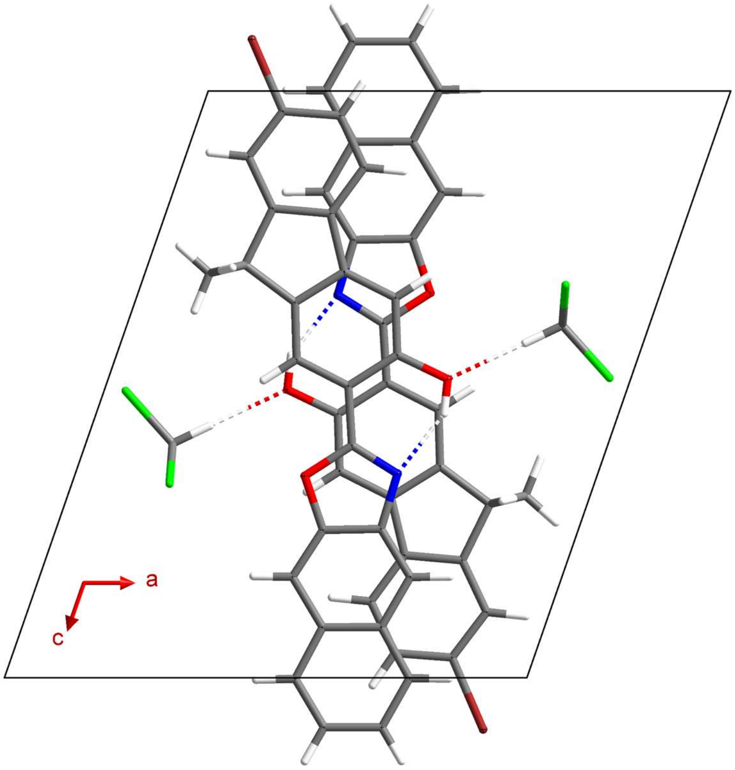

Figure S70: Unit cell packing view of a **NO-OH** crystal along the *b*-axis.

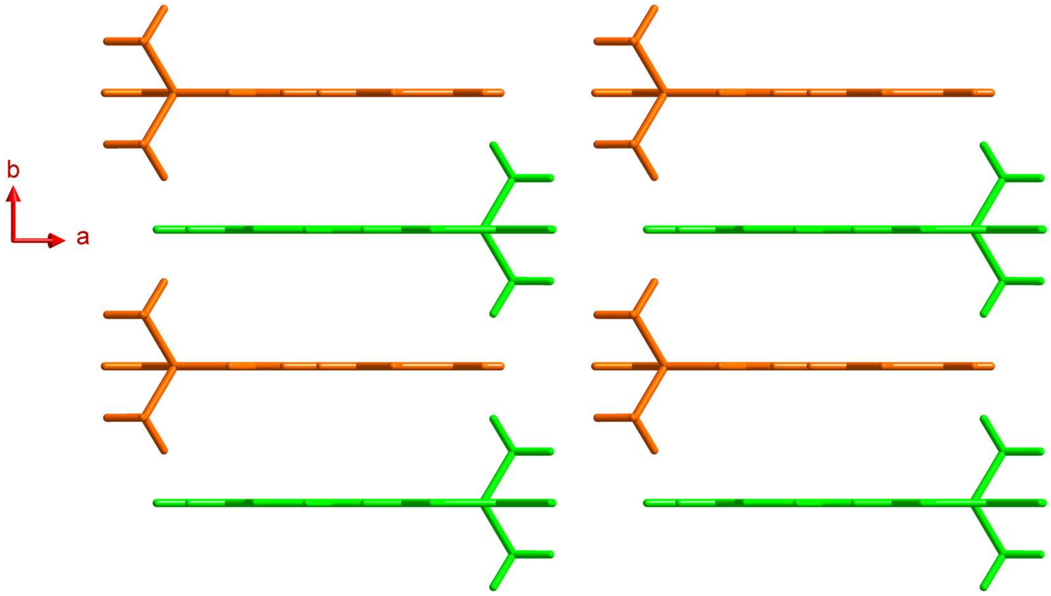

Figure S71: Crystal packing of **NO-OH** along the *c*-axis. For a greater clarity, identical molecules are colored green and orange, and coordinated $CHCl_3$ molecules are omitted.

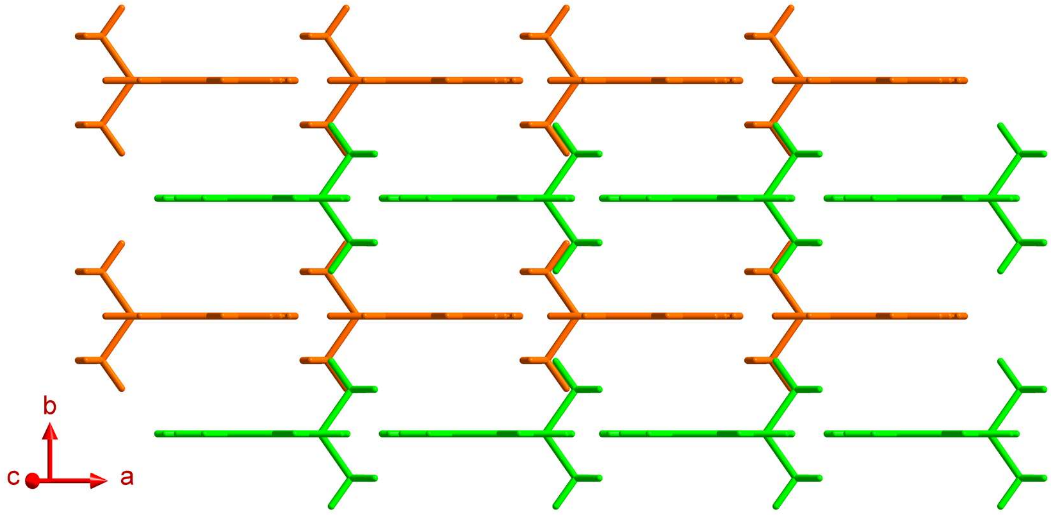

Figure S72: Crystal packing of **NO-OH**. For a greater clarity, identical molecules are colored green and orange, and coordinated $CHCl_3$ molecules are omitted.

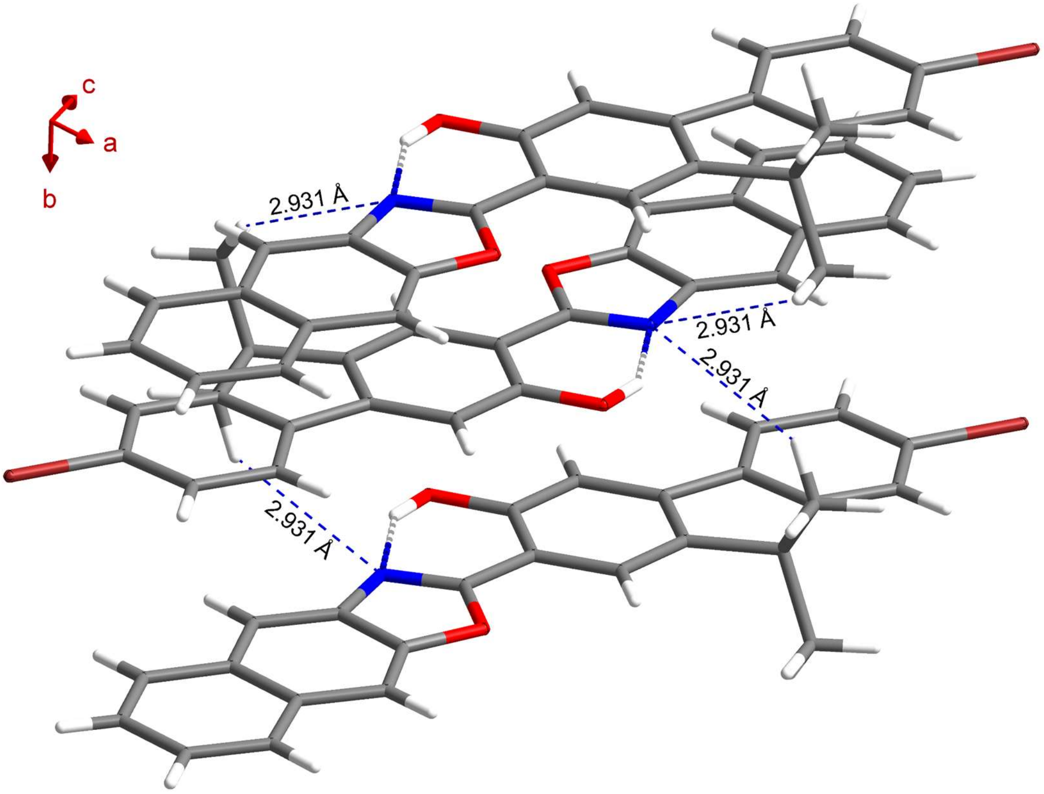

Figure S73: Various intermolecular interactions in **NO-OH** crystal lattice up to 3.000 Å distances, including C–H···N (blue dashed lines) interactions. All distances are given in Å. For a greater clarity, coordinated $CHCl_3$ molecules are omitted.

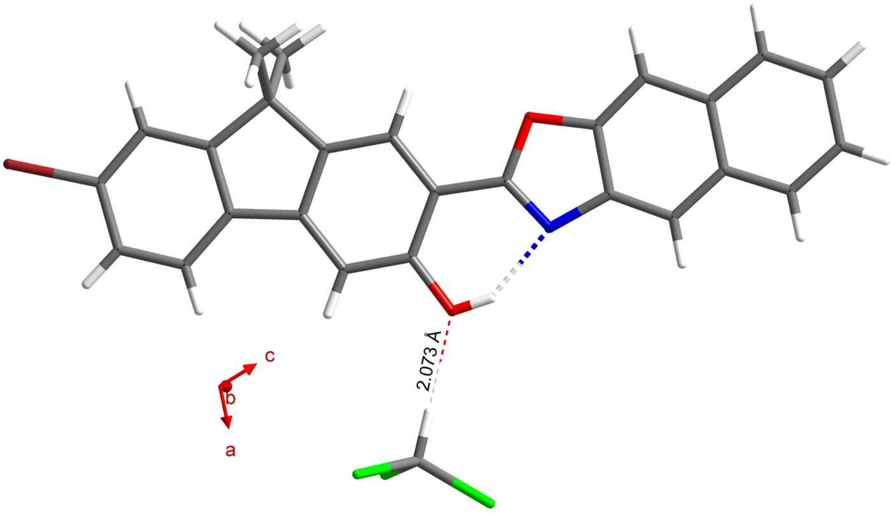

Figure S74: $CHCl_3$ coordination to phenolic oxygen of **NO-OH**.

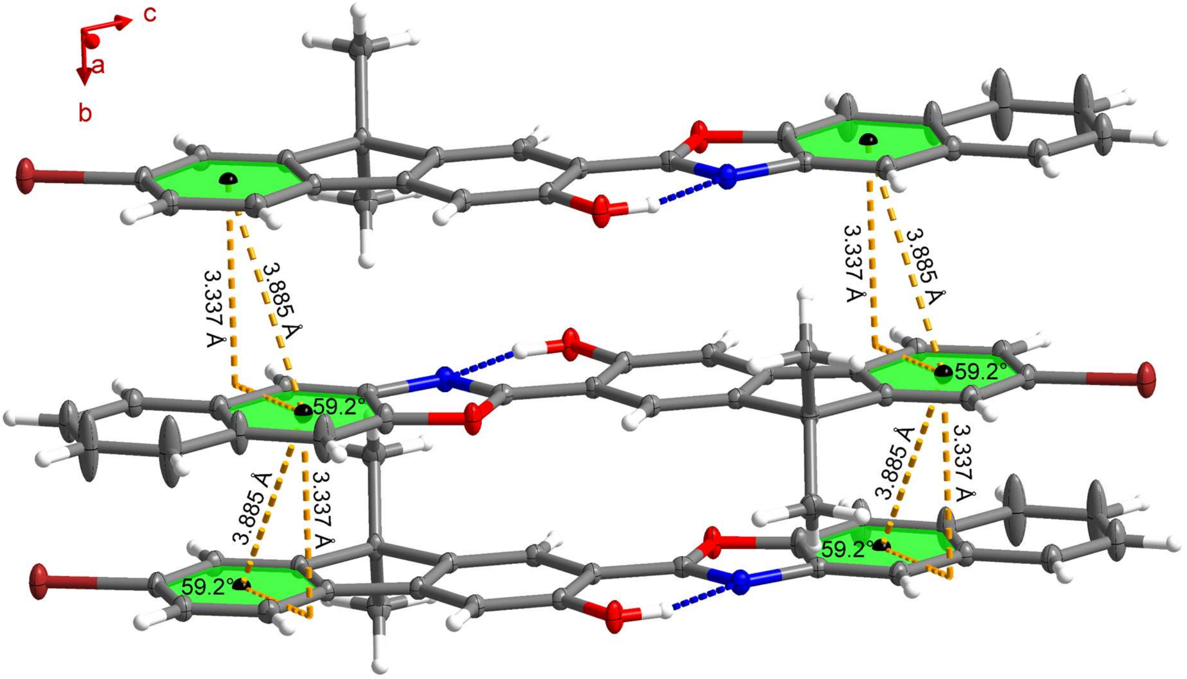

Figure S75: Intermolecular packing of three adjacent molecules in **NO-OH** crystal lattice with plane distance (orange dashed line) and centroid-centroid distance (orange dashed line between black spheres) with the corresponding slip angle. All distances are given in Å. The slip angle is given in °. For a greater clarity, coordinated $CHCl_3$ molecules are omitted.

4.6 2-(Benzo[*d*]thiazol-2-yl)-7-bromo-9,9-dimethyl-9*H*-fluoren-3-ol (BS-OH)

4.6.1 Crystal Data for BS-OH

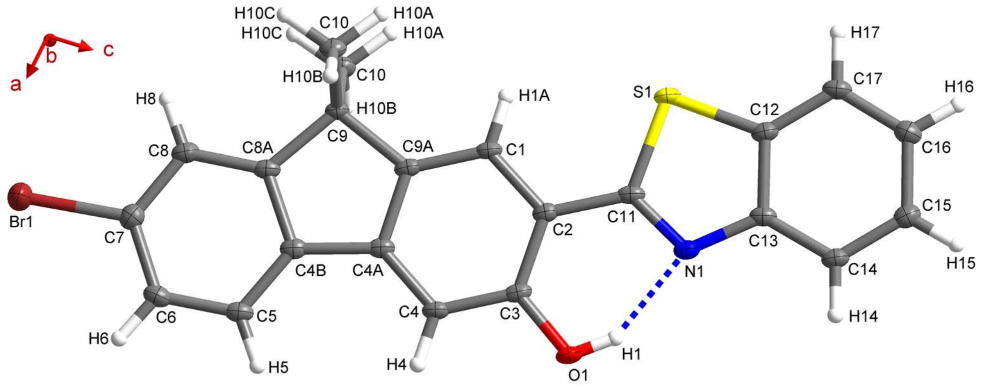

Figure S76: Asymmetric unit of **BS-OH** with atom labels.

Table S36: Crystal data and structure refinement for **BS-OH**.

Empirical formula	$C_{22}H_{16}NOSBr$
Formula weight/g mol^{-1}	422.33
Crystal system	Monoclinic
Crystal size/mm^3	0.2 × 0.15 × 0.15
Space group	$P2_1/m$
a/Å	8.5848(3)
b/Å	6.8902(3)
c/Å	15.8079(6)
α/°	90
β/°	98.9800(10)
γ/°	90
Volume/Å^3	923.59(6)
Z	2
$\rho_{calc,}$ g/cm^3	1.519
μ/mm^{-1}	2.349
F(000)	428.0
2Θ range for data collection/°	5.096 to 56.682

Index ranges	$-11 \le h \le 11$, $-9 \le k \le 9$, $-21 \le l \le 21$
No. of reflections collected	29406
No. of independent reflections	2485 [R_{int} = 0.0301, R_{sigma} = 0.0140]
Data/restraints/parameters	2485/0/162
Goodness-of-fit on F^2	1.076
Final R indexes [$I \ge 2\sigma$ (I)]	R_1 = 0.0243, wR_2 = 0.0602
Final R indexes [all data]	R_1 = 0.0277, wR_2 = 0.0618
Largest diff. peak/hole / e Å^{-3}	0.44/–0.28
CCDC number	2018046

Table S37: Fractional Atomic Coordinates (×10^4) and Equivalent Isotropic Displacement Parameters (Å^2×10^3) for **BS-OH**. U_{eq} is defined as 1/3 of the trace of the orthogonalized U_{IJ} tensor.

Atom	*x*	*y*	*z*	U(eq)
Br1	7131.1(3)	7500	-188.5(2)	33.25(10)
S1	1488.0(5)	7500	5521.6(3)	16.68(11)
O1	6928.3(15)	7500	5826.5(9)	19.0(3)
N1	4266.7(17)	7500	6444.1(10)	14.6(3)
C1	3546(2)	7500	4070.7(11)	14.7(3)
C2	4387(2)	7500	4913.8(11)	13.8(3)
C3	6056(2)	7500	5032.3(12)	14.9(3)
C4B	6524(2)	7500	2677.0(12)	14.7(3)
C4	6861(2)	7500	4335.7(12)	15.3(3)
C4A	6002(2)	7500	3513.0(12)	14.2(3)
C5	8033(2)	7500	2461.0(12)	16.7(3)
C6	8204(2)	7500	1601.0(12)	18.2(4)
C7	6861(2)	7500	979.0(12)	18.9(4)
C8A	5184(2)	7500	2038.0(12)	14.9(3)
C8	5344(2)	7500	1182.8(12)	17.8(4)
C9A	4340(2)	7500	3384.0(11)	14.0(3)
C9	3675(2)	7500	2432.3(11)	14.2(3)
C10	2676.8(14)	5678.3(19)	2181.4(8)	17.5(3)

C11	3554(2)	7500	5644.6(12)	14.9(3)
C12	1598(2)	7500	6627.5(12)	16.0(3)
C13	3194(2)	7500	7014.0(12)	14.9(3)
C14	3566(2)	7500	7907.7(12)	16.4(3)
C15	2355(2)	7500	8390.4(12)	18.0(4)
C16	775(2)	7500	7995.6(13)	19.5(4)
C17	377(2)	7500	7113.8(13)	18.2(4)

Table S38: Bond Lengths for **BS-OH**.

Atom	Atom	Length/Å	Atom	Atom	Length/Å
Br1	C7	1.8959(19)	C4A	C9A	1.409(2)
S1	C11	1.7539(18)	C5	C6	1.390(3)
S1	C12	1.7359(19)	C6	C7	1.394(3)
O1	C3	1.358(2)	C7	C8	1.389(3)
N1	C11	1.315(2)	C8A	C8	1.380(3)
N1	C13	1.385(2)	C8A	C9	1.523(2)
C1	C2	1.413(2)	C9A	C9	1.524(2)
C1	C9A	1.369(3)	C9	C10[1]	1.5364(15)
C2	C3	1.416(2)	C9	C10	1.5365(16)
C2	C11	1.450(3)	C12	C13	1.410(2)
C3	C4	1.388(3)	C12	C17	1.393(3)
C4B	C4A	1.460(3)	C13	C14	1.399(3)
C4B	C5	1.391(2)	C14	C15	1.382(3)
C4B	C8A	1.407(2)	C15	C16	1.402(3)
C4	C4A	1.391(2)	C16	C17	1.382(3)

[1]+X,3/2-Y,+Z

Table S39: Bond Angles for **BS-OH**.

Atom	Atom	Atom	Angle/°	Atom	Atom	Atom	Angle/°
C12	S1	C11	89.61(9)	C8A	C8	C7	117.87(17)
C11	N1	C13	111.60(15)	C1	C9A	C4A	120.23(16)
C9A	C1	C2	120.23(16)	C1	C9A	C9	128.78(15)

C1	C2	C3	118.82(16)	C4A	C9A	C9	110.99(15)
C1	C2	C11	120.57(16)	C8A	C9	C9A	101.07(13)
C3	C2	C11	120.61(15)	C8A	C9	C10[1]	111.72(10)
O1	C3	C2	121.52(17)	C8A	C9	C10	111.72(10)
O1	C3	C4	117.56(16)	C9A	C9	C10[1]	111.28(9)
C4	C3	C2	120.91(16)	C9A	C9	C10	111.28(9)
C5	C4B	C4A	130.64(17)	C10[1]	C9	C10	109.56(14)
C5	C4B	C8A	120.81(17)	N1	C11	S1	114.65(14)
C8A	C4B	C4A	108.55(16)	N1	C11	C2	123.52(16)
C3	C4	C4A	119.00(16)	C2	C11	S1	121.83(13)
C4	C4A	C4B	130.81(16)	C13	C12	S1	109.45(14)
C4	C4A	C9A	120.80(17)	C17	C12	S1	128.92(14)
C9A	C4A	C4B	108.39(15)	C17	C12	C13	121.64(17)
C6	C5	C4B	119.01(17)	N1	C13	C12	114.69(16)
C5	C6	C7	119.18(17)	N1	C13	C14	125.89(16)
C6	C7	Br1	118.22(14)	C14	C13	C12	119.42(17)
C8	C7	Br1	119.17(14)	C15	C14	C13	118.97(16)
C8	C7	C6	122.61(18)	C14	C15	C16	120.87(17)
C4B	C8A	C9	111.00(16)	C17	C16	C15	121.24(18)
C8	C8A	C4B	120.53(17)	C16	C17	C12	117.86(17)
C8	C8A	C9	128.47(16)				

[1]+X,3/2-Y,+Z

Table S40: Torsion Angles for **BS-OH**.

A	B	C	D	Angle/°	A	B	C	D	Angle/°
Br1	C7	C8	C8A	180.000(0)	C4A	C9A	C9	C10[1]	-118.75(10)
S1	C12	C13	N1	0.000(1)	C5	C4B	C4A	C4	0.000(1)
S1	C12	C13	C14	180.000(1)	C5	C4B	C4A	C9A	180.000(0)
S1	C12	C17	C16	180.000(1)	C5	C4B	C8A	C8	0.000(0)
O1	C3	C4	C4A	180.000(0)	C5	C4B	C8A	C9	180.000(0)
N1	C13	C14	C15	180.000(1)	C5	C6	C7	Br1	180.000(0)
C1	C2	C3	O1	180.000(0)	C5	C6	C7	C8	0.000(0)

C1	C2	C3	C4	0.000(1)	C6	C7	C8	C8A	0.000(0)
C1	C2	C11	S1	0.000(1)	C8A	C4B	C4A	C4	180.000(0)
C1	C2	C11	N1	180.000(1)	C8A	C4B	C4A	C9A	0.000(0)
C1	C9A	C9	C8A	180.000(0)	C8A	C4B	C5	C6	0.000(0)
C1	C9A	C9	C10[1]	61.25(10)	C8	C8A	C9	C9A	180.000(0)
C1	C9A	C9	C10	-61.25(10)	C8	C8A	C9	C10[1]	-61.57(10)
C2	C1	C9A	C4A	0.000(0)	C8	C8A	C9	C10	61.57(10)
C2	C1	C9A	C9	180.000(0)	C9A	C1	C2	C3	0.000(1)
C2	C3	C4	C4A	0.000(1)	C9A	C1	C2	C11	180.000(0)
C3	C2	C11	S1	180.000(0)	C9	C8A	C8	C7	180.000(0)
C3	C2	C11	N1	0.000(1)	C11	S1	C12	C13	0.000(0)
C3	C4	C4A	C4B	180.000(0)	C11	S1	C12	C17	180.000(1)
C3	C4	C4A	C9A	0.000(1)	C11	N1	C13	C12	0.000(1)
C4B	C4A	C9A	C1	180.000(0)	C11	N1	C13	C14	180.000(1)
C4B	C4A	C9A	C9	0.000(0)	C11	C2	C3	O1	0.000(1)
C4B	C5	C6	C7	0.000(0)	C11	C2	C3	C4	180.000(1)
C4B	C8A	C8	C7	0.000(0)	C12	S1	C11	N1	0.000(0)
C4B	C8A	C9	C9A	0.000(0)	C12	S1	C11	C2	180.000(0)
C4B	C8A	C9	C10[1]	118.43(10)	C12	C13	C14	C15	0.000(1)
C4B	C8A	C9	C10	-118.43(10)	C13	N1	C11	S1	0.000(1)
C4	C4A	C9A	C1	0.000(0)	C13	N1	C11	C2	180.000(1)
C4	C4A	C9A	C9	180.000(0)	C13	C12	C17	C16	0.000(1)
C4A	C4B	C5	C6	180.000(0)	C13	C14	C15	C16	0.000(1)
C4A	C4B	C8A	C8	180.000(0)	C14	C15	C16	C17	0.000(1)
C4A	C4B	C8A	C9	0.000(0)	C15	C16	C17	C12	0.000(1)
C4A	C9A	C9	C8A	0.000(0)	C17	C12	C13	N1	180.000(1)
C4A	C9A	C9	C10	118.75(10)	C17	C12	C13	C14	0.000(1)

[1]+X,3/2-Y,+Z

Table S41: Hydrogen Atom Coordinates (Å×10^4) and Isotropic Displacement Parameters (Å^2×10^3) for **BS-OH**.

Atom	*x*	*y*	*z*	U(eq)
H1	6340(30)	7500	6182(17)	25(7)

H1A	2425.74	7500	3980.03	18
H4	7981.05	7500	4419.47	18
H5	8933.91	7500	2894.74	20
H6	9224.61	7500	1439.03	22
H8	4446.13	7500	747.4	21
H10A	1753.56	5684.72	2476.11	26
H10B	3311.21	4517.65	2347.43	26
H10C	2329	5670.46	1560.75	26
H14	4630(30)	7500	8181(14)	15(5)
H15	2596.94	7500	8997.73	22
H16	-37.43	7500	8340.47	23
H17	-694.83	7500	6847.88	22

4.6.2 Crystal Packing Views of BS-OH

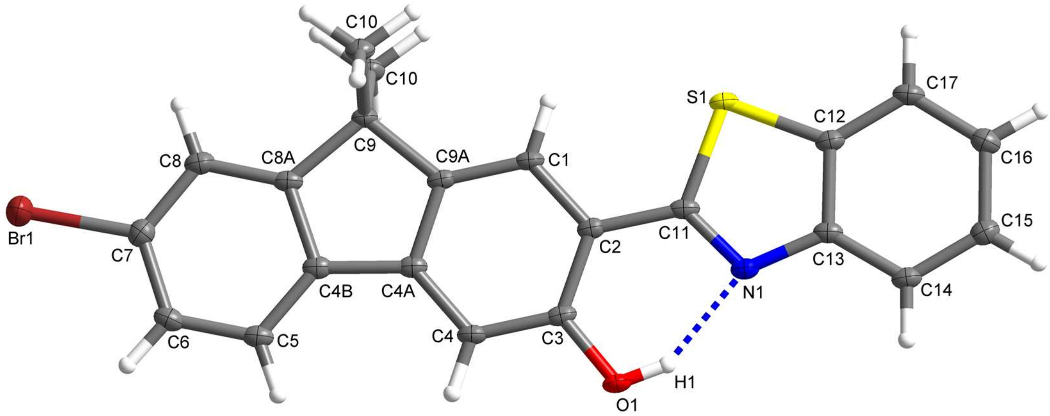

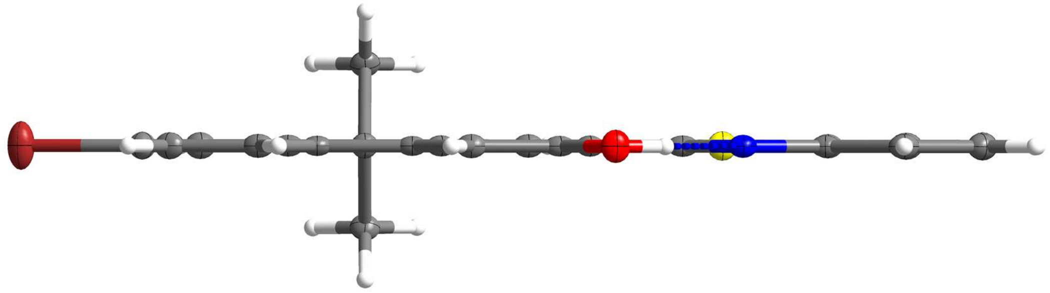

Figure S77: Top view with atom labels (top) and side view (bottom) of **BS-OH**.

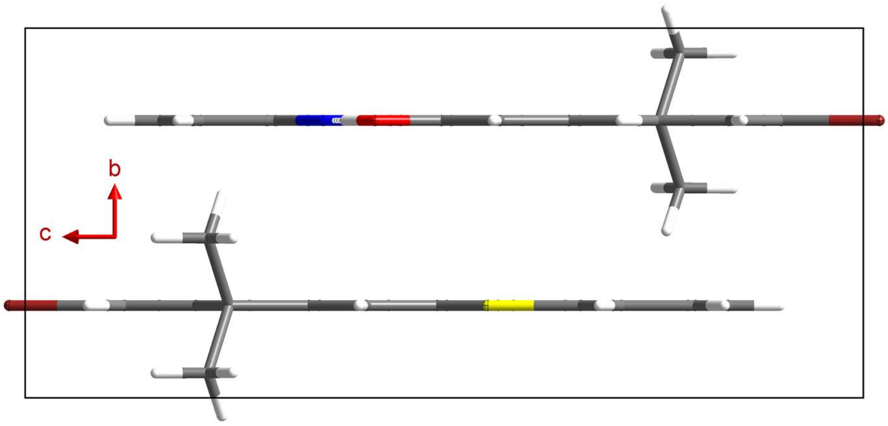

Figure S78: Unit cell packing view of a **BS-OH** crystal along the *a*-axis.

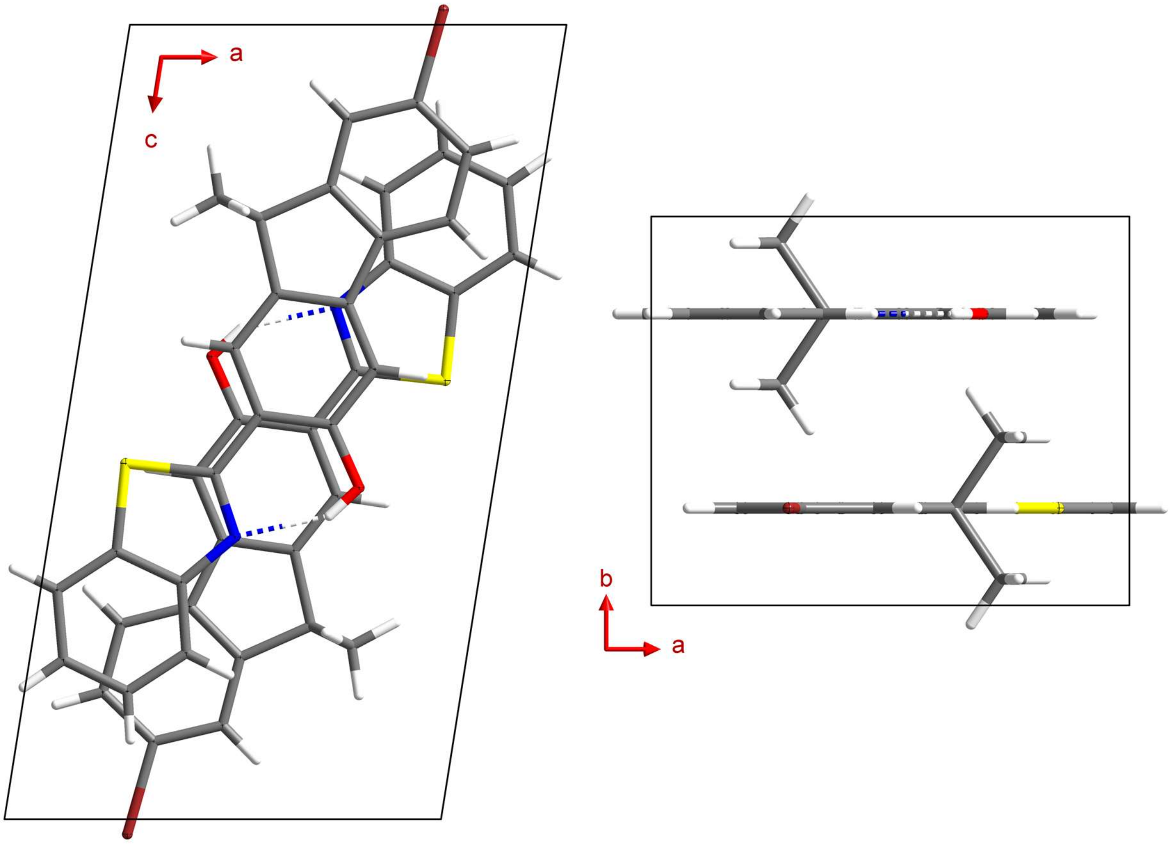

Figure S79: Unit cell packing view of a **BS-OH** crystal along the *b*-axis (left) and the *c*-axis (right).

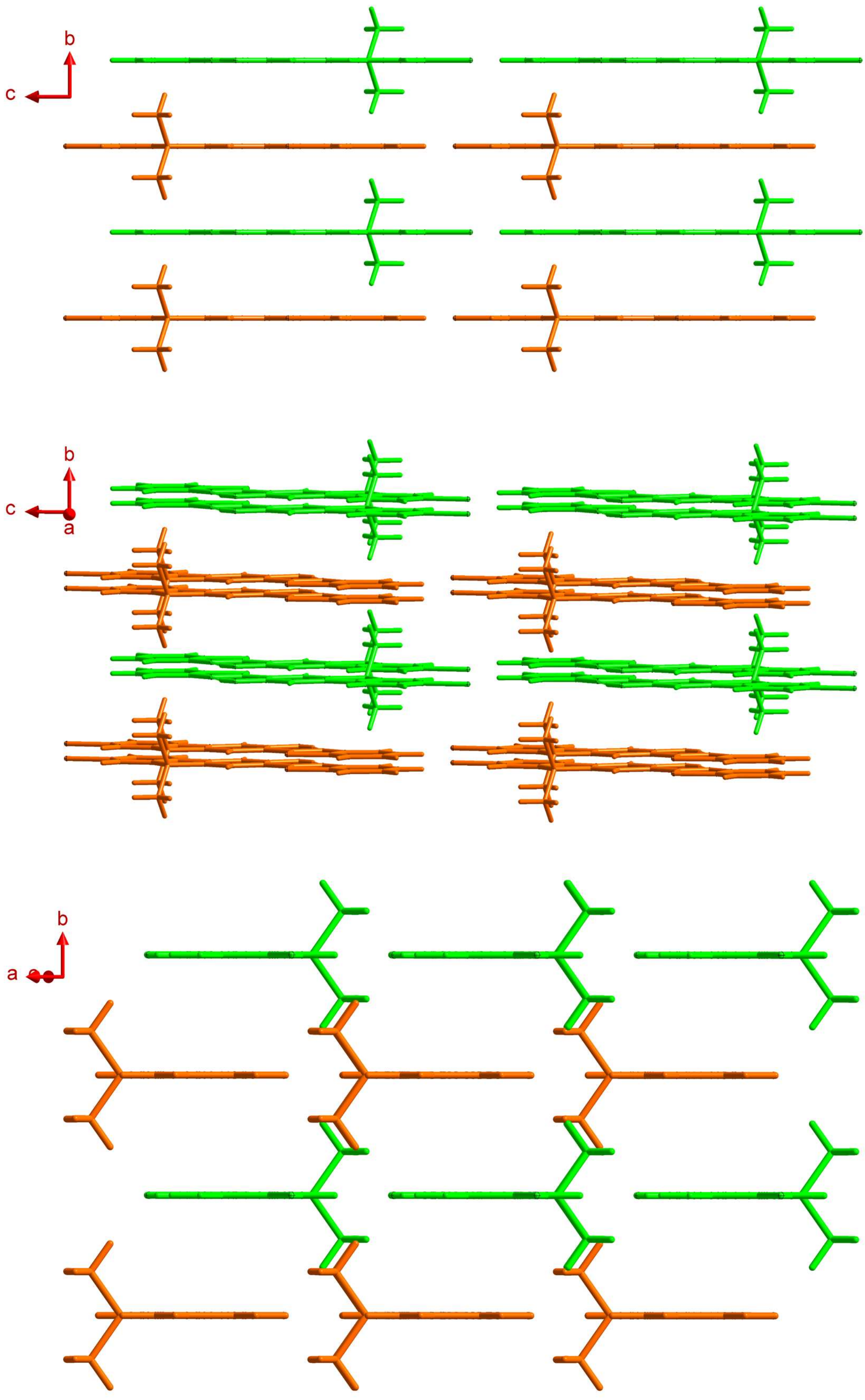

Figure S80: Crystal packing of **BS-OH**. For a greater clarity, identical molecules are colored green and orange.

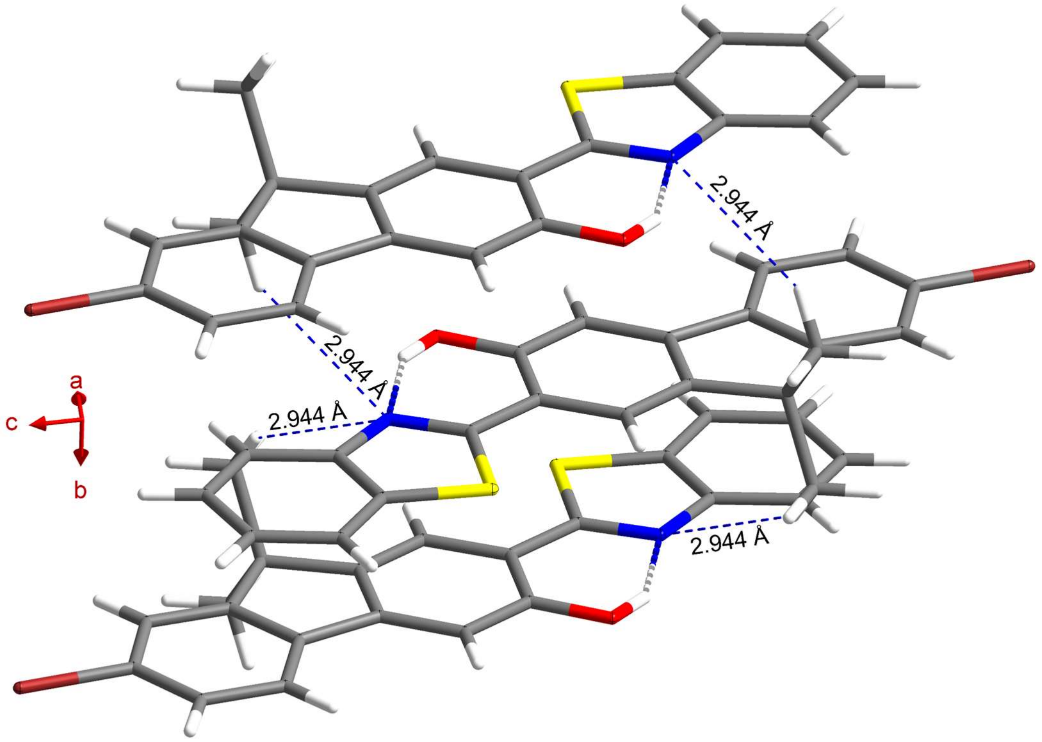

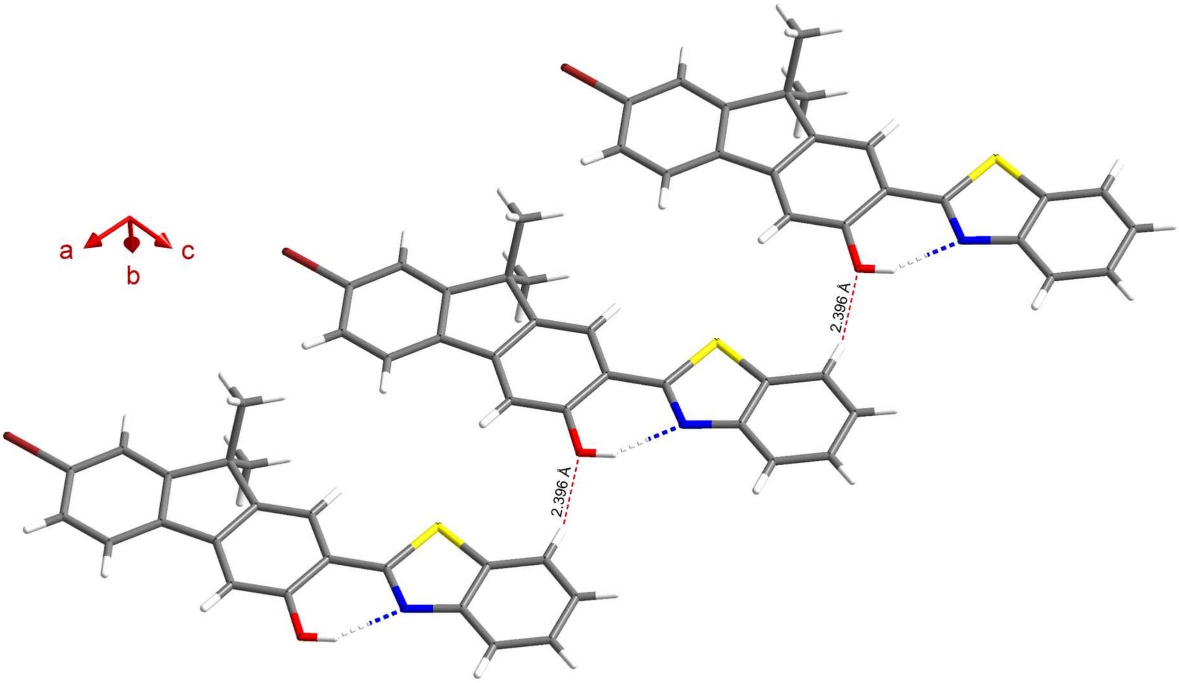

Figure S81: Various intermolecular interactions in **BS-OH** crystal lattice up to 3.000 Å distances, including C–H···O (red dashed lines) and C–H···N (blue dashed lines) interactions. All distances are given in Å.

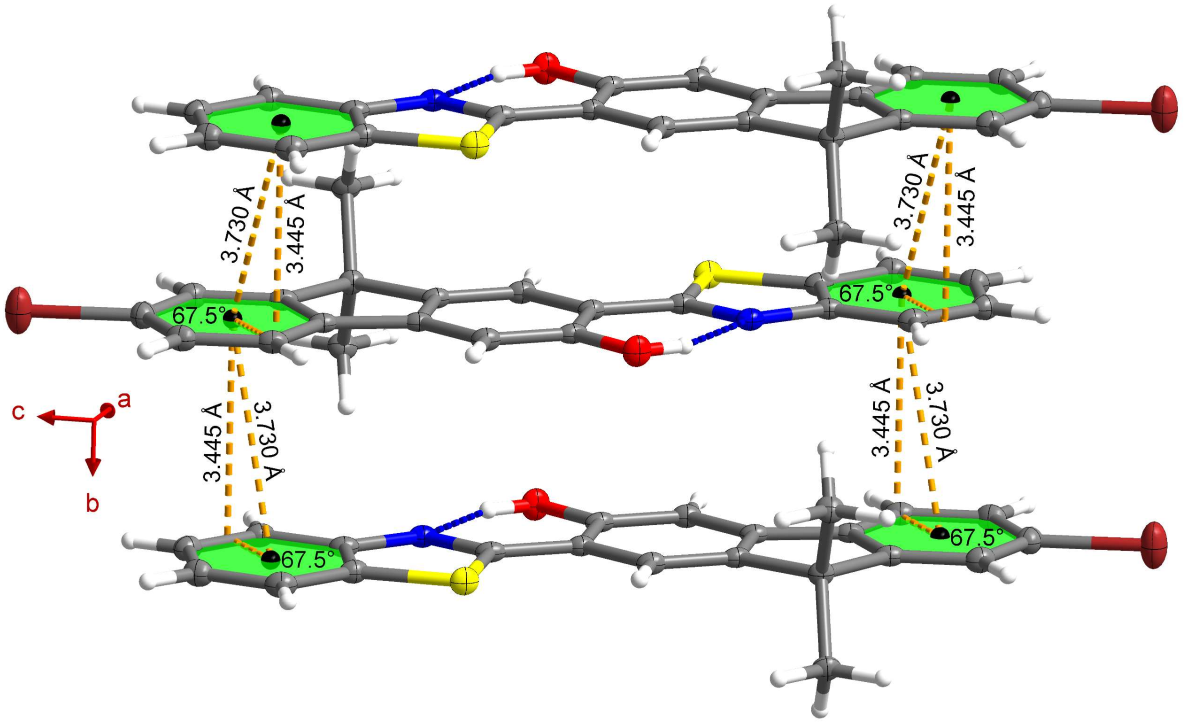

Figure S82: Intermolecular packing of three adjacent molecules in **BS-OH** crystal lattice with plane distance (orange dashed line) and centroid-centroid distance (orange dashed line between black spheres) with the corresponding slip angle. All distances are given in Å. The slip angle is given in °.

4.7 2-(Benzo[*d*][1,3]selenazol-2-yl)-7-bromo-9,9-dimethyl-9*H*-fluoren-3-ol (BSe-OH)

4.7.1 Crystal Data for BSe-OH

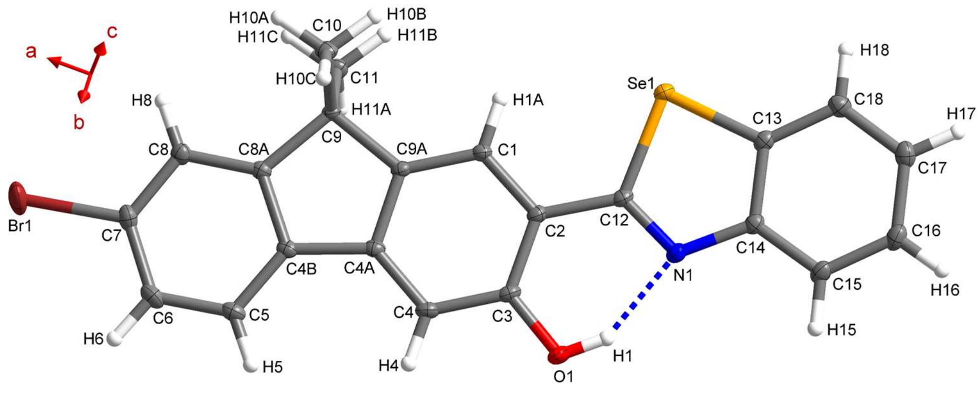

Figure S83: Asymmetric unit of **BSe-OH** with atom labels.

Table S42: Crystal data and structure refinement for **BSe-OH**.

Empirical formula	$C_{22}H_{16}BrNOSe$
Formula weight/g mol^{-1}	469.23

Crystal system	Orthorhombic
Crystal size/mm^3	0.28 × 0.27 × 0.19
Space group	Pccn
a/Å	30.762(3)
b/Å	10.8014(11)
c/Å	11.0953(11)
α/°	90
β/°	90
γ/°	90
Volume/Å^3	3686.7(6)
Z	8
$\rho_{calc,}$ g/cm^3	1.691
μ/mm^{-1}	4.215
F(000)	1856.0
2Θ range for data collection/°	3.996 to 66.588
Index ranges	–47 ≤ h ≤ 47, –16 ≤ k ≤ 16, –17 ≤ l ≤ 17
No. of reflections collected	203320
No. of independent reflections	7095 [R_{int} = 0.0505, R_{sigma} = 0.0164]
Data/restraints/parameters	7095/0/238
Goodness-of-fit on F^2	1.055
Final R indexes [I ≥ 2σ (I)]	R_1 = 0.0274, wR_2 = 0.0562
Final R indexes [all data]	R_1 = 0.0374, wR_2 = 0.0593
Largest diff. peak/hole / e Å^{-3}	0.56/–0.54
CCDC number	2018049

Table S43: Fractional Atomic Coordinates (×10^4) and Equivalent Isotropic Displacement Parameters (Å^2×10^3) for **BSe-OH**. U_{eq} is defined as 1/3 of the trace of the orthogonalized U_{IJ} tensor.

Atom	*x*	*y*	*z*	U(eq)
Br1	7597.5(2)	4348.5(2)	1559.3(2)	27.22(4)
Se1	4514.9(2)	1852.4(2)	5907.4(2)	13.86(3)
O1	4562.1(3)	5144.5(10)	3052.9(10)	17.2(2)

N1	4155.0(4)	3652.7(11)	4514.0(11)	13.2(2)
C1	5329.8(4)	2946.3(12)	4443.2(12)	12.3(2)
C2	4933.4(4)	3534.5(12)	4173.3(12)	11.8(2)
C3	4930.4(4)	4535.1(12)	3342.2(12)	12.5(2)
C4	5313.2(4)	4911.4(12)	2778.5(12)	13.5(2)
C4A	5697.5(4)	4304.4(12)	3060.1(11)	11.3(2)
C4B	6139.4(4)	4500.8(12)	2611.5(11)	11.1(2)
C5	6297.3(5)	5323.7(13)	1753.2(12)	14.4(2)
C6	6734.5(5)	5280.4(13)	1446.2(13)	16.8(3)
C7	7003.7(5)	4425.4(13)	2017.7(13)	15.9(2)
C8	6854.5(4)	3609.6(12)	2893.8(12)	13.5(2)
C8A	6416.9(4)	3652.3(12)	3182.5(11)	10.8(2)
C9	6167.9(4)	2838.3(11)	4063.9(11)	10.4(2)
C9A	5708.8(4)	3325.2(12)	3894.1(11)	10.9(2)
C10	6323.3(4)	3032.7(13)	5365.8(12)	14.2(2)
C11	6207.6(4)	1461.3(12)	3735.3(13)	14.1(2)
C12	4530.7(4)	3142.1(12)	4741.1(12)	12.3(2)
C13	3917.5(4)	2207.8(12)	5994.2(12)	12.8(2)
C14	3815.1(4)	3161.8(12)	5181.9(12)	12.5(2)
C15	3385.3(4)	3574.1(13)	5092.2(13)	15.3(2)
C16	3070.5(4)	3051.9(13)	5821.0(14)	17.1(3)
C17	3176.7(5)	2108.1(13)	6631.9(14)	17.1(3)
C18	3600.7(5)	1673.0(13)	6723.1(13)	15.6(2)

Table S44: Bond Lengths for **BSe-OH**.

Atom	Atom	Length/Å	Atom	Atom	Length/Å
Br1	C7	1.8981(14)	C4B	C8A	1.4037(18)
Se1	C12	1.9019(13)	C5	C6	1.388(2)
Se1	C13	1.8796(13)	C6	C7	1.393(2)
O1	C3	1.3489(16)	C7	C8	1.3900(19)
N1	C12	1.3050(16)	C8	C8A	1.3845(18)
N1	C14	1.3872(17)	C8A	C9	1.5219(18)

C1	C2	1.4071(18)	C9	C9A	1.5187(18)
C1	C9A	1.3777(18)	C9	C10	1.5359(18)
C2	C3	1.4208(18)	C9	C11	1.5363(18)
C2	C12	1.4531(18)	C13	C14	1.4048(19)
C3	C4	1.3939(19)	C13	C18	1.3920(19)
C4	C4A	1.3874(18)	C14	C15	1.3986(18)
C4A	C4B	1.4631(18)	C15	C16	1.382(2)
C4A	C9A	1.4057(17)	C16	C17	1.398(2)
C4B	C5	1.3903(18)	C17	C18	1.390(2)

Table S45: Bond Angles for **BO-OH**.

Atom	Atom	Atom	Angle/°	Atom	Atom	Atom	Angle/°
C13	Se1	C12	84.85(6)	C8	C8A	C9	128.23(12)
C12	N1	C14	113.75(12)	C8A	C9	C10	111.63(10)
C9A	C1	C2	120.33(12)	C8A	C9	C11	111.51(11)
C1	C2	C3	119.16(12)	C9A	C9	C8A	100.85(10)
C1	C2	C12	120.99(12)	C9A	C9	C10	111.02(11)
C3	C2	C12	119.85(11)	C9A	C9	C11	112.33(10)
O1	C3	C2	122.08(12)	C10	C9	C11	109.32(11)
O1	C3	C4	117.41(12)	C1	C9A	C4A	119.58(12)
C4	C3	C2	120.50(12)	C1	C9A	C9	128.99(11)
C4A	C4	C3	118.75(12)	C4A	C9A	C9	111.43(11)
C4	C4A	C4B	130.28(12)	N1	C12	Se1	114.73(10)
C4	C4A	C9A	121.66(12)	N1	C12	C2	123.22(12)
C9A	C4A	C4B	108.06(11)	C2	C12	Se1	122.03(9)
C5	C4B	C4A	130.57(12)	C14	C13	Se1	109.65(9)
C5	C4B	C8A	120.94(12)	C18	C13	Se1	129.02(11)
C8A	C4B	C4A	108.47(11)	C18	C13	C14	121.33(12)
C6	C5	C4B	119.02(13)	N1	C14	C13	117.00(11)
C5	C6	C7	119.11(13)	N1	C14	C15	123.57(12)
C6	C7	Br1	118.62(10)	C15	C14	C13	119.43(12)
C8	C7	Br1	118.52(11)	C16	C15	C14	119.39(13)

C8	C7	C6	122.84(13)	C15	C16	C17	120.68(13)
C8A	C8	C7	117.50(13)	C18	C17	C16	120.84(13)
C4B	C8A	C9	111.17(11)	C17	C18	C13	118.32(13)
C8	C8A	C4B	120.57(12)				

Table S46: Torsion Angles for **BSe-OH**.

A	B	C	D	Angle/°	A	B	C	D	Angle/°
Br1	C7	C8	C8A	-177.72(10)	C5	C6	C7	C8	-0.3(2)
Se1	C13	C14	N1	-0.86(15)	C6	C7	C8	C8A	1.0(2)
Se1	C13	C14	C15	179.73(10)	C7	C8	C8A	C4B	-0.57(19)
Se1	C13	C18	C17	179.27(11)	C7	C8	C8A	C9	177.44(13)
O1	C3	C4	C4A	179.82(12)	C8	C8A	C9	C9A	-177.04(13)
N1	C14	C15	C16	-178.12(13)	C8	C8A	C9	C10	65.00(17)
C1	C2	C3	O1	-179.42(12)	C8	C8A	C9	C11	-57.61(17)
C1	C2	C3	C4	1.53(19)	C8A	C4B	C5	C6	1.3(2)
C1	C2	C12	Se1	1.76(18)	C8A	C9	C9A	C1	179.32(13)
C1	C2	C12	N1	-179.52(13)	C8A	C9	C9A	C4A	-1.53(13)
C2	C1	C9A	C4A	-0.04(19)	C9A	C1	C2	C3	-0.96(19)
C2	C1	C9A	C9	179.05(12)	C9A	C1	C2	C12	179.74(12)
C2	C3	C4	C4A	-1.08(19)	C9A	C4A	C4B	C5	177.71(13)
C3	C2	C12	Se1	-177.54(10)	C9A	C4A	C4B	C8A	-0.62(14)
C3	C2	C12	N1	1.2(2)	C10	C9	C9A	C1	-62.27(17)
C3	C4	C4A	C4B	179.89(13)	C10	C9	C9A	C4A	116.88(12)
C3	C4	C4A	C9A	0.1(2)	C11	C9	C9A	C1	60.48(18)
C4	C4A	C4B	C5	-2.1(2)	C11	C9	C9A	C4A	-120.37(12)
C4	C4A	C4B	C8A	179.53(13)	C12	Se1	C13	C14	0.94(10)
C4	C4A	C9A	C1	0.51(19)	C12	Se1	C13	C18	-178.52(13)
C4	C4A	C9A	C9	-178.74(12)	C12	N1	C14	C13	0.13(17)
C4A	C4B	C5	C6	-176.87(13)	C12	N1	C14	C15	179.51(13)
C4A	C4B	C8A	C8	177.94(12)	C12	C2	C3	O1	-0.10(19)
C4A	C4B	C8A	C9	-0.38(14)	C12	C2	C3	C4	-179.16(12)
C4B	C4A	C9A	C1	-179.36(11)	C13	C14	C15	C16	1.2(2)

C4B	C4A	C9A	C9	1.40(14)	C14	N1	C12	Se1	0.68(15)
C4B	C5	C6	C7	-0.8(2)	C14	N1	C12	C2	-178.12(12)
C4B	C8A	C9	C9A	1.13(13)	C14	C13	C18	C17	-0.1(2)
C4B	C8A	C9	C10	-116.84(12)	C14	C15	C16	C17	-0.8(2)
C4B	C8A	C9	C11	120.56(12)	C15	C16	C17	C18	-0.1(2)
C5	C4B	C8A	C8	-0.58(19)	C16	C17	C18	C13	0.5(2)
C5	C4B	C8A	C9	-178.90(12)	C18	C13	C14	N1	178.64(12)
C5	C6	C7	Br1	178.42(11)	C18	C13	C14	C15	-0.8(2)

Table S47: Hydrogen Atom Coordinates (Å×10^4) and Isotropic Displacement Parameters (Å^2×10^3) for **BSe-OH**.

**Atom**	***x***	***y***	***z***	**U(eq)**
H1	4353.7	4854.11	3452.05	26
H1A	5336.07	2284.58	5007	15
H4	5310.96	5571	2212.15	16
H5	6108.75	5906.82	1382.18	17
H6	6848.65	5826.64	854.35	20
H8	7045.62	3044	3280.03	16
H10A	6627.94	2777.43	5435.37	21
H10B	6144.53	2535.01	5913.3	21
H10C	6296.49	3909.81	5579.22	21
H11A	6106.67	1333.86	2906.8	21
H11B	6029.43	967.98	4287.97	21
H11C	6512.14	1203.56	3801.5	21
H15	3310.43	4207.24	4535.32	18
H16	2778.88	3336.97	5770.6	20
H17	2956.33	1759.94	7126.76	21
H18	3672.41	1026.98	7269.07	19

4.7.2 Crystal Packing Views of BSe-OH

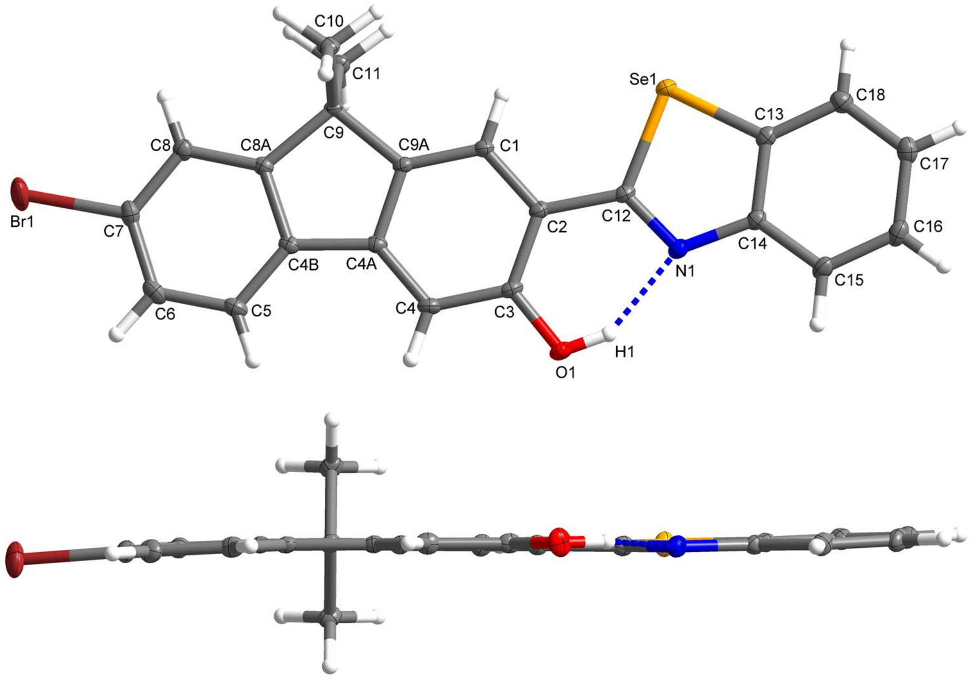

Figure S84: Top view with atom labels (top) and side view (bottom) of **BSe-OH**.

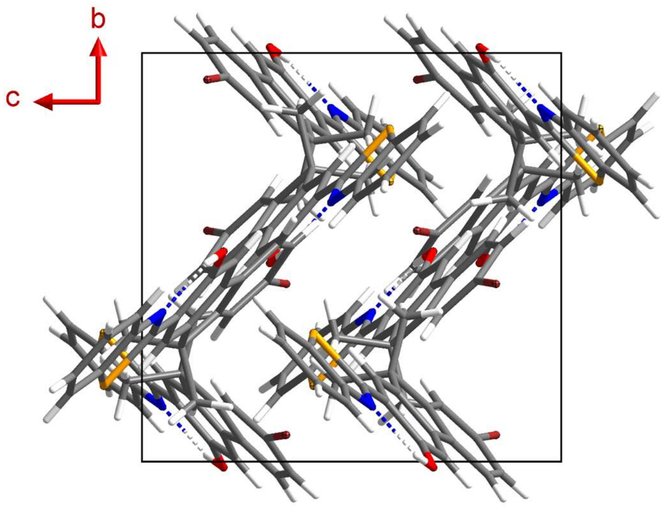

Figure S85: Unit cell packing view of a **BSe-OH** crystal along the *a*-axis.

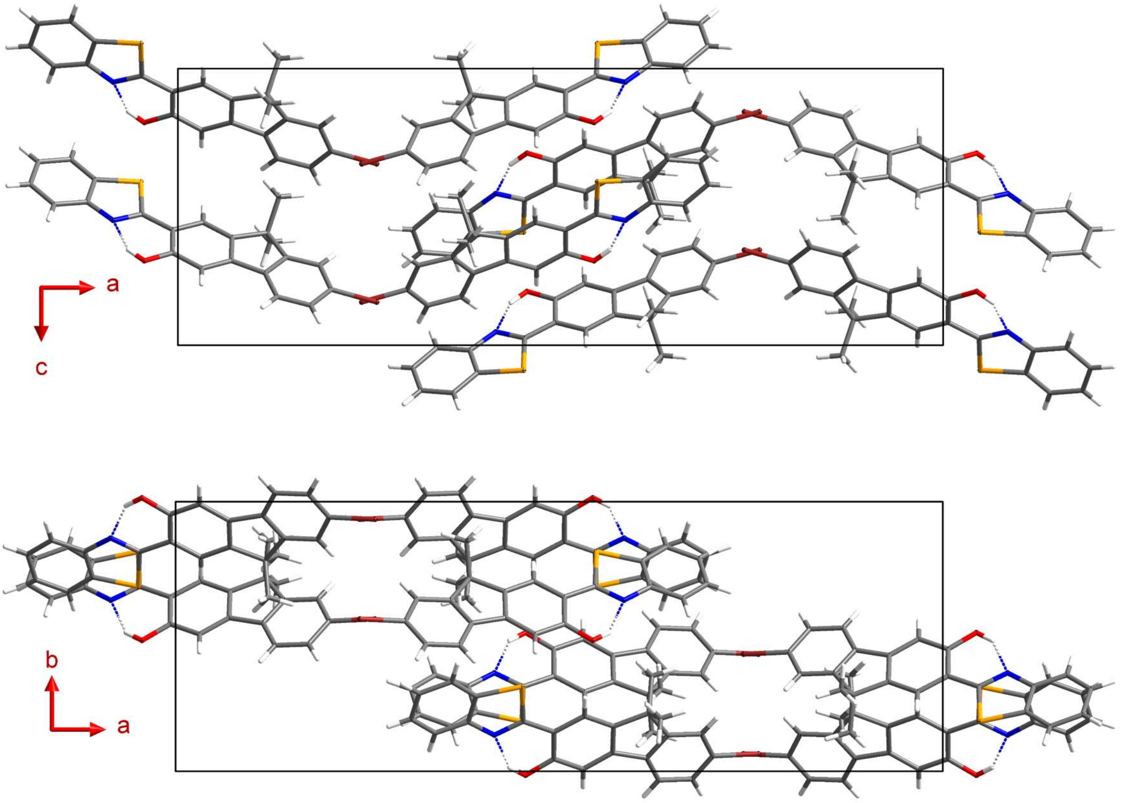

Figure S86: Unit cell packing view of a **BSe-OH** crystal along the *b*-axis (top) and the *c*-axis (bottom).

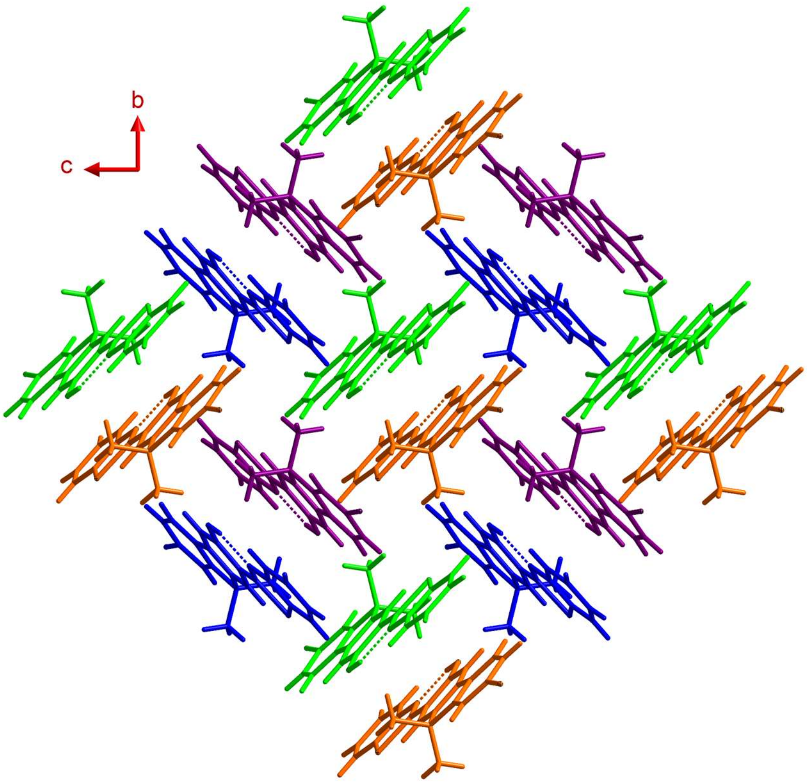

Figure S87: Crystal packing of **BSe-OH** along the *a*-axis. For a greater clarity, identical molecules are colored violet, blue, green and orange.

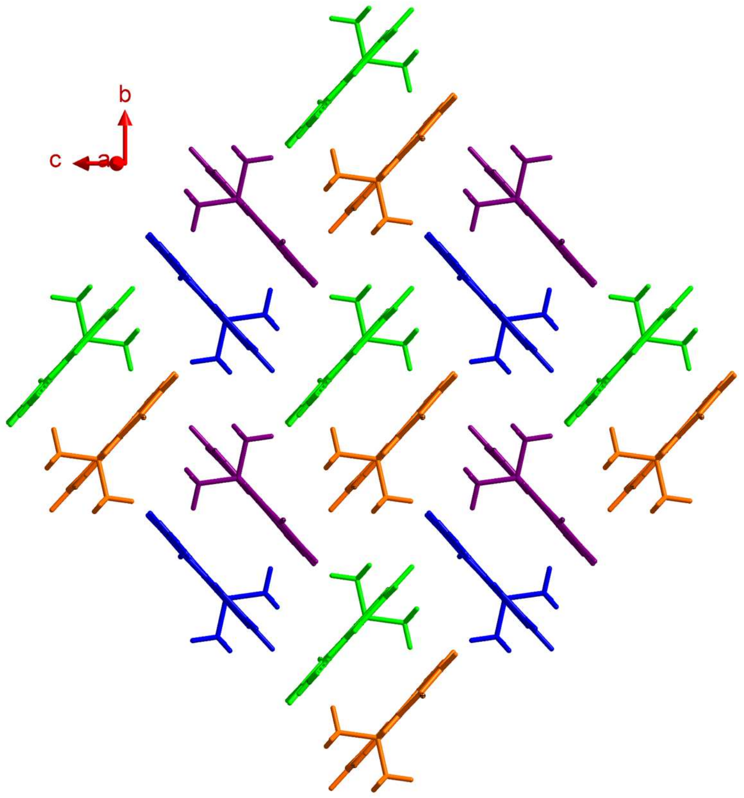

Figure S88: Crystal packing of **BSe-OH** demonstrating the sandwich herringbone crystal packing. For a greater clarity, identical molecules are colored violet, blue, green and orange.

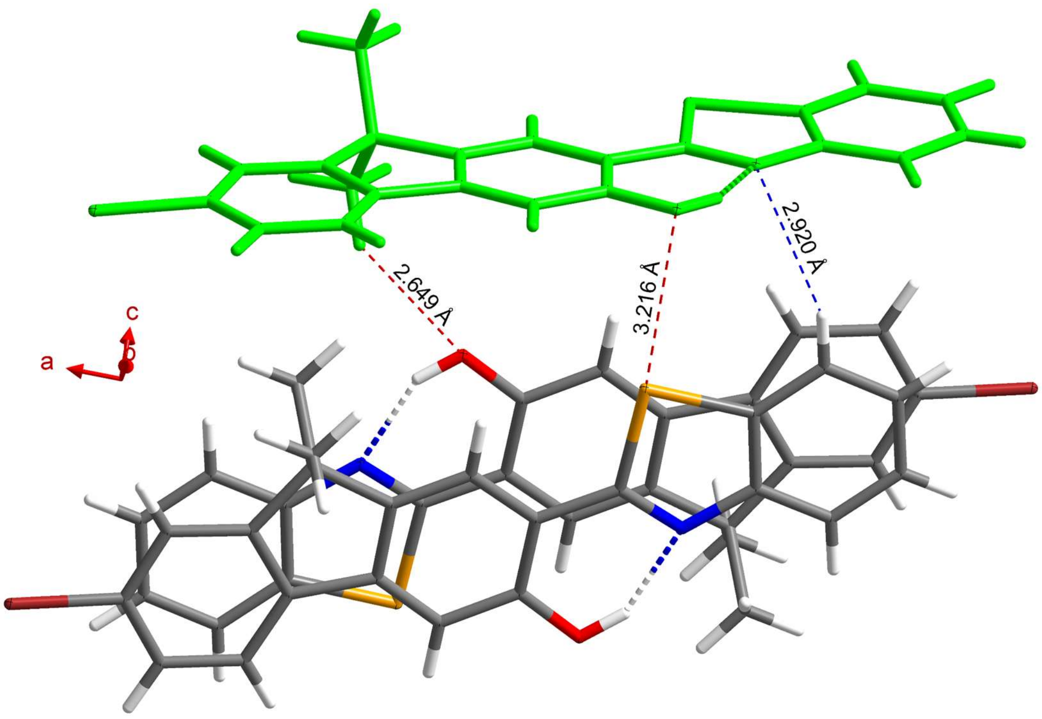

Figure S89: Various intermolecular interactions in **BSe-OH** crystal lattice up to 3.000 Å distances, including C–H···O (red dashed lines) and C–H···N (blue dashed lines) interactions. All distances are given in Å. For a greater clarity, one definite molecule is colored green.

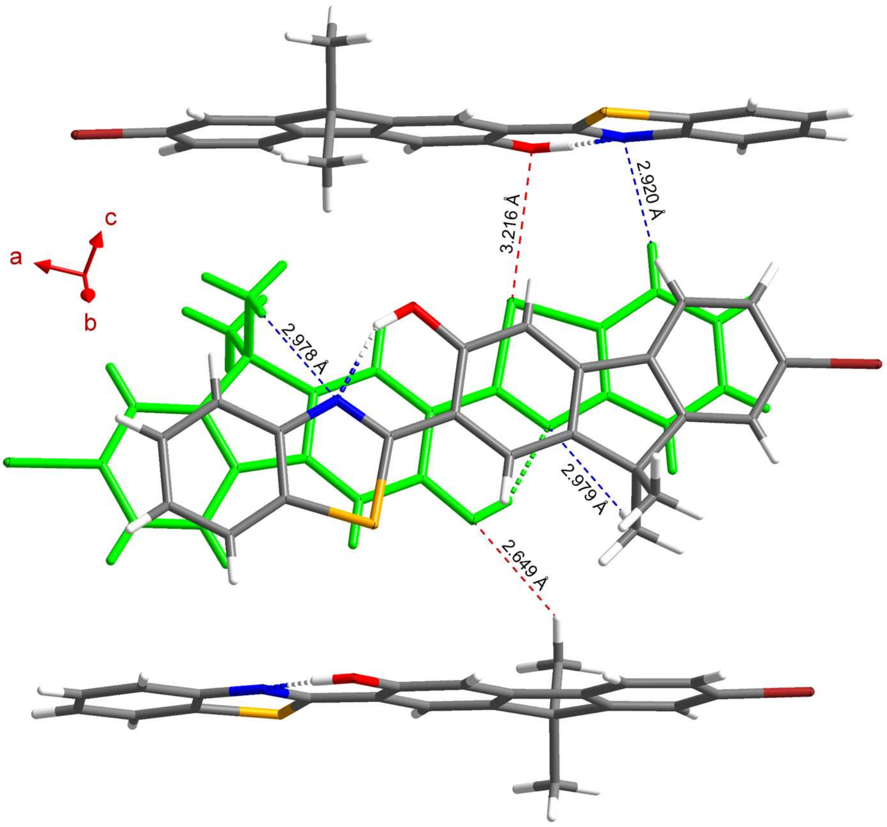

Figure S90: Various intermolecular interactions in **BSe-OH** crystal lattice up to 3.000 Å distances, including C–H···O (red dashed lines) and C–H···N (blue dashed lines) interactions. All distances are given in Å. For a greater clarity, one definite molecule is colored green.

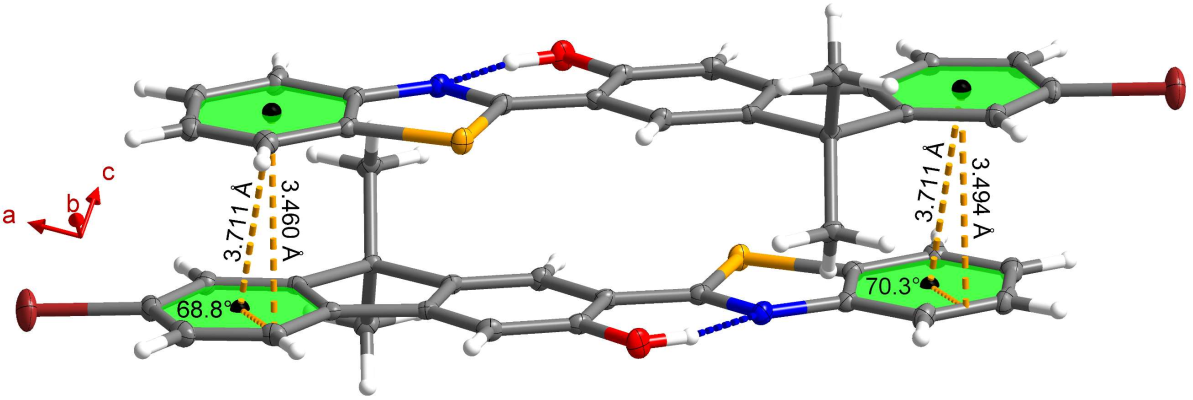

Figure S91: Intermolecular packing of two adjacent molecules in **BSe-OH** crystal lattice with plane distance (orange dashed line) and centroid-centroid distance (orange dashed line between black spheres) with the corresponding slip angle. All distances are given in Å. The slip angle is given in °.

4.8 Crystal Properties of Hydroxylated Fluorenes

Table S48: Hydrogen bond (···) and crystal parameters of **Oxa-OH**, **(Oxa)$_2$-OH**, **(Oxa-OH)$_2$**, **BO-OH**, **NO-OH**, **BS-OH** and **BSe-OH**.

Compd	O1–H1···N1			Dihedral angle C3–C2–C12–N1 [°]	Slip angles [°]	Plane distance [Å] with corresponding centroid-centroid distance ([Å])
	Length H1···N1 [Å]	Length O1···N1 [Å]	Angle [°]			
**Oxa-OH**	1.8670(6)[1]	2.6189(7)[1]	148.255(4)[1]	2.7(9)[1]	–	–
	1.8724(6)[2]	2.6179(7)[2]	147.196(4)[2]	3.118(9)[2]	–	–
	1.8696(6)[3]	2.5983(8)[3]	144.212(4)[3]	1.927(9)[3]	–	–
	1.850(5)[4]	2.5945(7)[4]	146.955(4)[4]	3.536(9)[4]	–	–
**(Oxa)$_2$-OH**	1.8618(18)	2.6122(3)	147.970(12)	–2.1(3)	–	–
**(Oxa-OH)$_2$**	1.7523(3)<br>1.7284(2)[5]	2.5850(13)<br>2.5933(14)[5]	149.850(19)<br>150.856(18)[5]	–0.46(17)<br>–1.42(17)[6]	–	–
**BO-OH**	1.8989(4)	2.6305(3)	150.144(3)	–2.7(2)	62.8 – 83.2[7]	3.401 (3.419) – 3.533 (3.883)[7]
**NO-OH**	1.8481(4)	2.6015(5)	148.495(19)	0	59.2	3.337 (3.885)
**BS-OH**	1.8894(3)	2.6212(3)	149.390(3)	0[8]	67.5	3.445 (3.730)
**BSe-OH**	1.8563(12)	2.6063(16)	147.891(8)	1.2(2)	68.8; 70.3	3.460 (3.711);<br>3.494 (3.711)

[1] Data for molecule 1 of the asymmetric unit. [2] Data for molecule 2 of the asymmetric unit. [3] Data for molecule 3 of the asymmetric unit. [4] Data for molecule 4 of the asymmetric unit. [5] Data for hydrogen bond O3–H3···N2. [6] Data for dihedral angle C6–C7–C17–N2 [7] Due to several π-π-interactions, a range of slip angles and plane distances is given [8] Atom C12 is replaced by atom C11.

Detailed Analysis of Crystal Properties

As can be seen at first glance, oxazolinyl-substituted fluorenes exhibit a distorted structure along the fluorene-oxazoline-plane, while arylchalcogenazolyl-substituted fluorenes are planar (Figure 3). Due to their distorted structure, oxazolinyl-substituted fluorenes show no molecular packing motifs in the crystal lattice, hence, slip angles and plane distances cannot be determined. However, hydrogen bond distances H···N of **Oxa-OH** and **(Oxa)$_2$-OH** are ranging between 1.850 Å and 1.8724 Å, while O···N distances varying from 2.5945 to 2.6189 Å (Table S48). Hydrogen bond angles are rather small with values in between 144.212° and 148.255°. These parameters indicate that hydrogen bonds of **Oxa-OH** and **(Oxa)$_2$-OH** exhibit an average strength compared to known ESIPT luminophores.[25] Dihedral angles between the fluorene unit and the oxazoline group are assigned to be between |1.927°| and |3.536°|. Due to this relatively large torsion, proton transfer is rather not facilitated, which is in contrast to the identified quantum yield of 38%.

(Oxa-OH)$_2$ with its two hydrogen bonds exhibits significantly shortened H···N lengths with 1.728 and 1.752 Å, respectively. Additionally, O···N distances of 2.5850 and 2.5933 Å, hydrogen bond angles of 149.850° and 150.856°, and dihedral angles of |0.46°| and |1.42°| indicate stronger

hydrogen bonds for **(Oxa-OH)$_2$**. Despite the improved hydrogen bond parameters, the quantum yield of **(Oxa-OH)$_2$** (18%) is not higher than of **Oxa-OH** (38%).

General tendencies can be observed for arylchalcogenazolyl-substituted fluorenes. In comparison to **BO-OH**, hydrogen bond lengths and angles decreases while walking down the chalcogenic group (**BS-OH** and **BSe-OH**) and upon π-expansion (**NO-OH**). Since smaller hydrogen bond angles should impede proton transfer in the excited state, the reduced quantum yields of **NO-OH** (3%) and **BSe-OH** (7%) confirm this tendency. In contrast, the shortened hydrogen bond lengths should result in improved quantum yields, which is indeed no the case for heavier chalcogen analogues. The molecular structures of **NO-OH** and **BS-OH** demonstrate perfect planarity with a dihedral angle of 0°, while **BO-OH** and **BSe-OH** exhibit a slightly increased torsion of |2.7°| and |1.2°|. Due to their great planarity, arylchalcogenazolyl-substituted fluorophores exhibit intense intermolecular π-π-interactions. According to slip angels, ranging from 59.2° (**NO-OH**) to 83.2° (**BO-OH**), cross-stacking in antiparallel sheet structures are observed for **BO-OH**, **NO-OH**, **BS-OH**. Except **BSe-OH**, which demonstrates crystal packing of a sandwich herringbone type. Since all slip angles of arylchalcogenazolyl-substituted fluorenes ranging above 54.7°, the molecular packing motifs are attributed to be H-aggregates.[26]

5 References

1 W. L. F. Armarego and C. L. L. Chai, *Purification of Laboratory Chemicals*, Elsevier/BH, Oxford, 6th edn., 2009.

2 a) O. V. Dolomanov, L. J. Bourhis, R. J. Gildea, J. A. K. Howard and H. Puschmann, *J. Appl. Cryst.*, 2009, **42**, 339–341; b) G. M. Sheldrick, *Acta Cryst. A*, 2008, **64**, 112–122;

3 A. Krasovskiy and P. Knochel, *Angew. Chem. Int. Ed.*, 2004, **43**, 3333–3336.

4 F. Bodroux, *C. R. Chim.*, 1902, **135**, 1350–1351.

5 A. Krasovskiy, V. Krasovskaya and P. Knochel, *Angew. Chem. Int. Ed.*, 2006, **45**, 2958–2961.

6 L. P. Hammett, G. H. Walden and S. M. Edmonds, *J. Am. Chem. Soc.*, 1934, **56**, 1092–1094.

7 D. Göbel, N. Clamor, E. Lork and B. J. Nachtsheim, *Org. Lett.*, 2019, **21**, 5373–5377.

8 K. Seth, M. Nautiyal, P. Purohit, N. Parikh and A. K. Chakraborti, *Chem. Commun.*, 2015, **51**, 191–194.

9 J. Yang, C. Jiang, Y. Zhang, R. Yang, W. Yang, Q. Hou and Y. Cao, *Macromolecules*, 2004, **37**, 1211–1218.

10 C. Chakraborty, M. K. Bera, U. Rana and S. Malik, *Chem. Commun.*, 2015, **51**, 13123–13126.

11 D. Göbel, N. Clamor and B. J. Nachtsheim, *Org. Biomol. Chem.*, 2018, **16**, 4071–4075.

12 V. Singh, S. Wang and E. T. Kool, *J. Am. Chem. Soc.*, 2013, **135**, 6184–6191.

13 R. Hosseinzadeh, M. Mohadjerani and M. Pooryousef, *Luminescence*, 2015, **30**, 549–555.

14 S. E. Denmark and L. R. Cullen, *J. Org. Chem.*, 2015, **80**, 11818–11848.

15 K. Schwekendiek and F. Glorius, *Synthesis*, 2006, **2006**, 2996–3002.

16 C.-C. Chi, Y.-J. Huang and C.-T. Chen, *J. Chin. Chem. Soc.*, 2012, **59**, 305–316.

17 M. Ishihara and H. Togo, *Tetrahedron*, 2007, **63**, 1474–1480.

18 H. Naeimi, Z. Rouzegar and S. Rahmatinejad, *Synth. Commun.*, 2017, **47**, 2087–2095.

19 J. Chang, K. Zhao and S. Pan, *Tetrahedron Lett.*, 2002, **43**, 951–954.

20 R. Kannan, G. S. He, L. Yuan, F. Xu, P. N. Prasad, A. G. Dombroskie, B. A. Reinhardt, J. W. Baur, R. A. Vaia and L.-S. Tan, *Chem. Mater.*, 2001, **13**, 1896–1904.

21 A. M. Deobald, L. R. Simon de Camargo, G. Tabarelli, M. Hörner, O. E.D. Rodrigues, D. Alves and A. L. Braga, *Tetrahedron Lett.*, 2010, **51**, 3364–3367.

22 D. Singh, A. M. Deobald, L. R. S. Camargo, G. Tabarelli, O. E. D. Rodrigues and A. L. Braga, *Org. Lett.*, 2010, **12**, 3288–3291.

23 S. J. Balkrishna, B. S. Bhakuni and S. Kumar, *Tetrahedron*, 2011, **67**, 9565–9575.

24 C. S. Radatz, D. Alves and P. H. Schneider, *Tetrahedron*, 2013, **69**, 1316–1321.

25 D. Göbel, D. Duvinage, T. Stauch and B. J. Nachtsheim, *J. Mater. Chem. C*, 2020, **8**, 9213–9225.

26 N. J. Hestand and F. C. Spano, *Chem. Rev.*, 2018, **118**, 7069–7163.

6 NMR Spectra

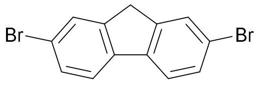

S1, $CDCl_3$

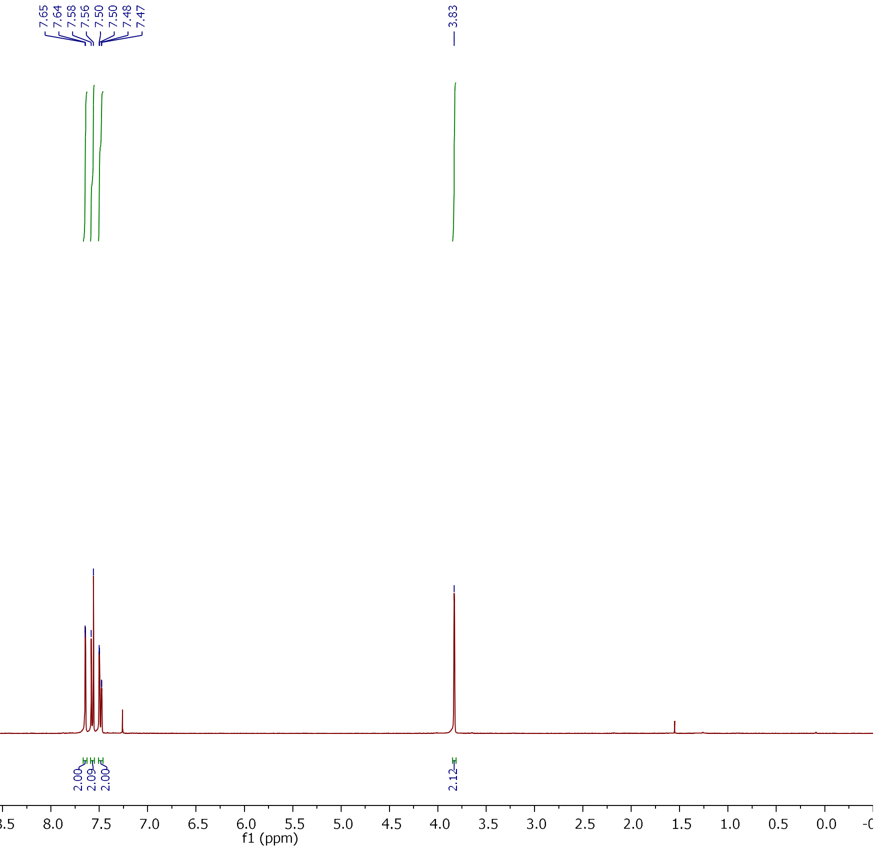

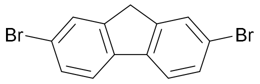

S1, $CDCl_3$

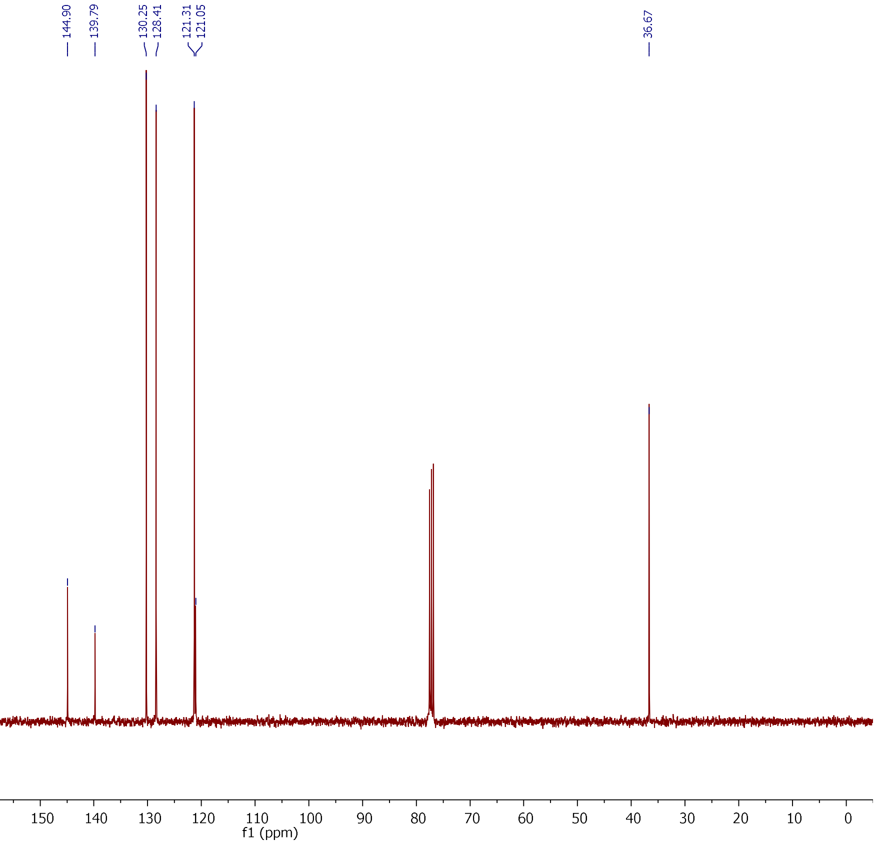

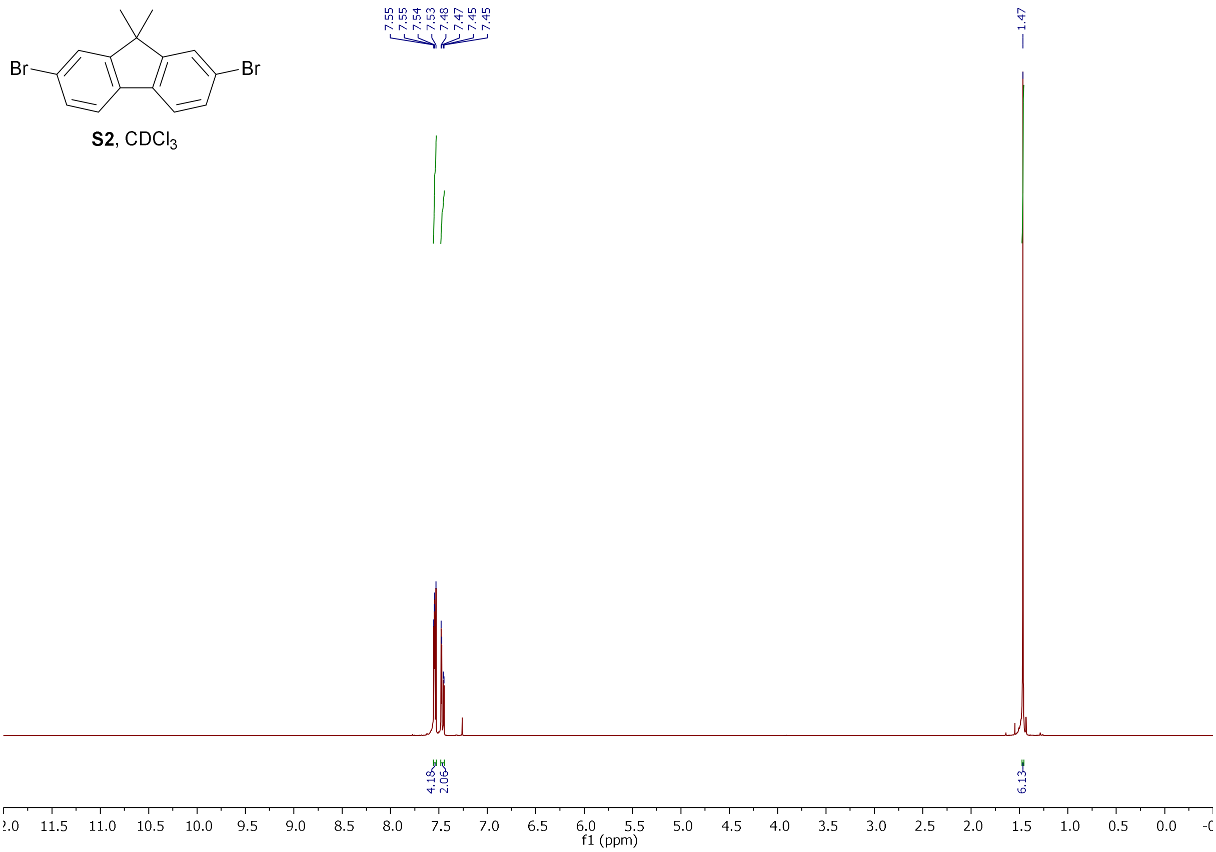

Br
Br
S2, $CDCl_3$
7.55 7.55 7.54 7.53 7.48 7.47 7.45 7.45
1.47
4.18
2.06
6.13
f1 (ppm)

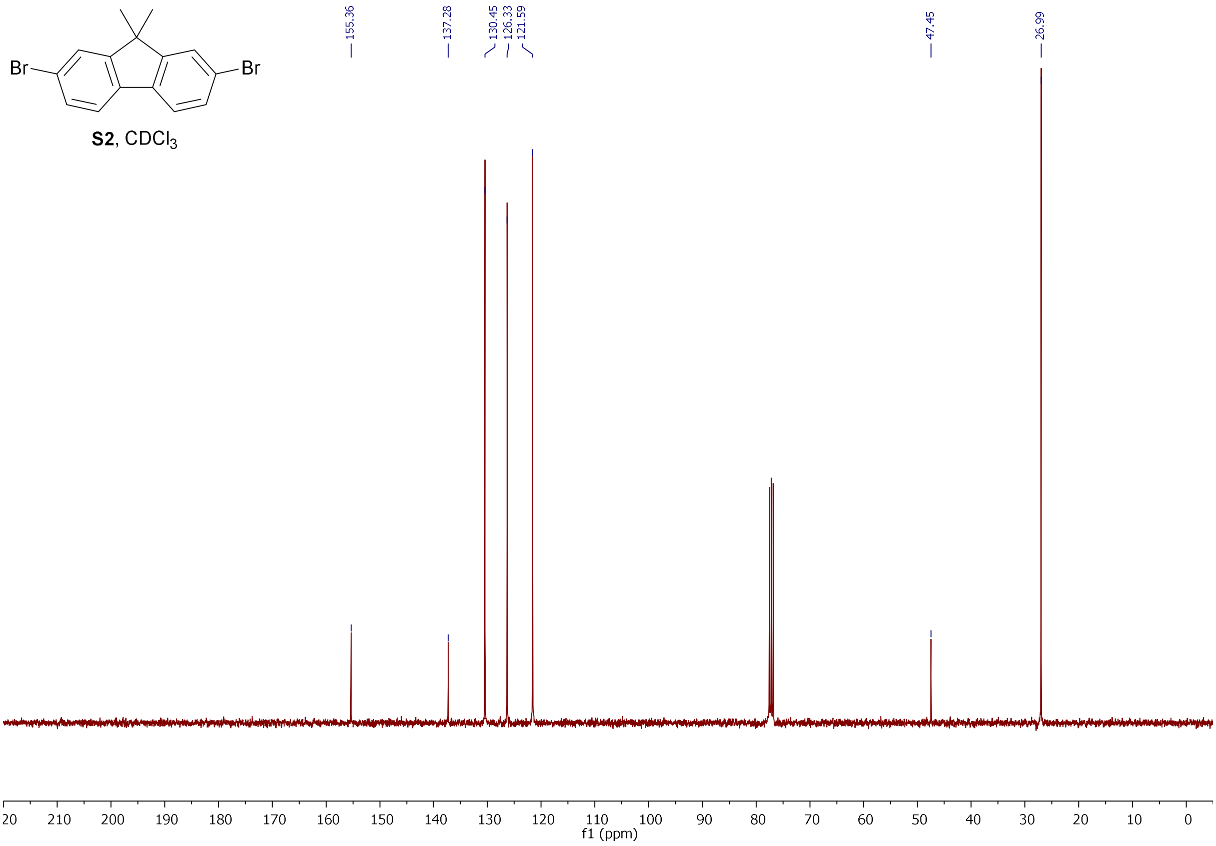

Br
Br
S2, $CDCl_3$
155.36
137.28
130.45
126.33
121.59
47.45
26.99
f1 (ppm)

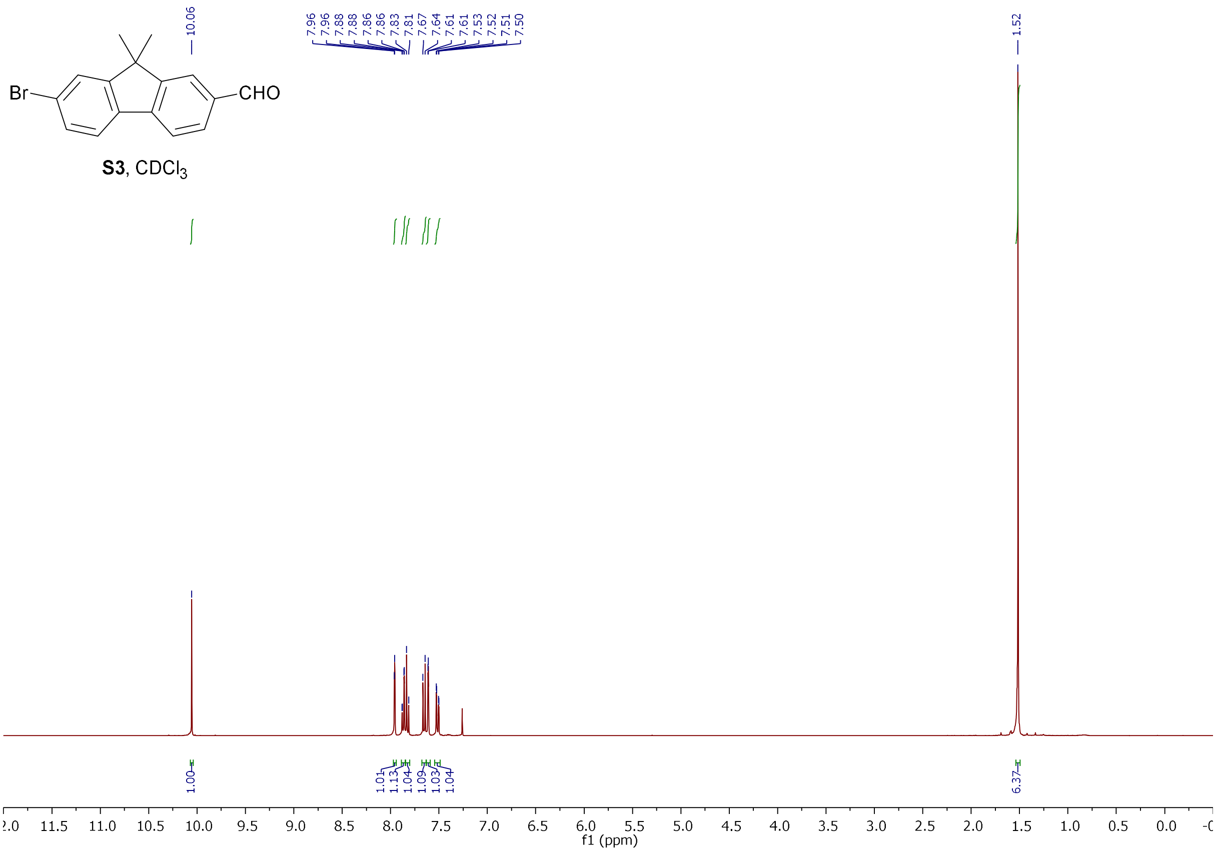
Br
CHO
S3, CDCl3
10.06
7.96 7.96 7.88 7.88 7.86 7.86 7.83 7.81 7.67 7.64 7.61 7.61 7.53 7.52 7.51 7.50
1.52
1.00
1.01 1.13 1.04 1.09 1.03 1.04
6.37
f1 (ppm)

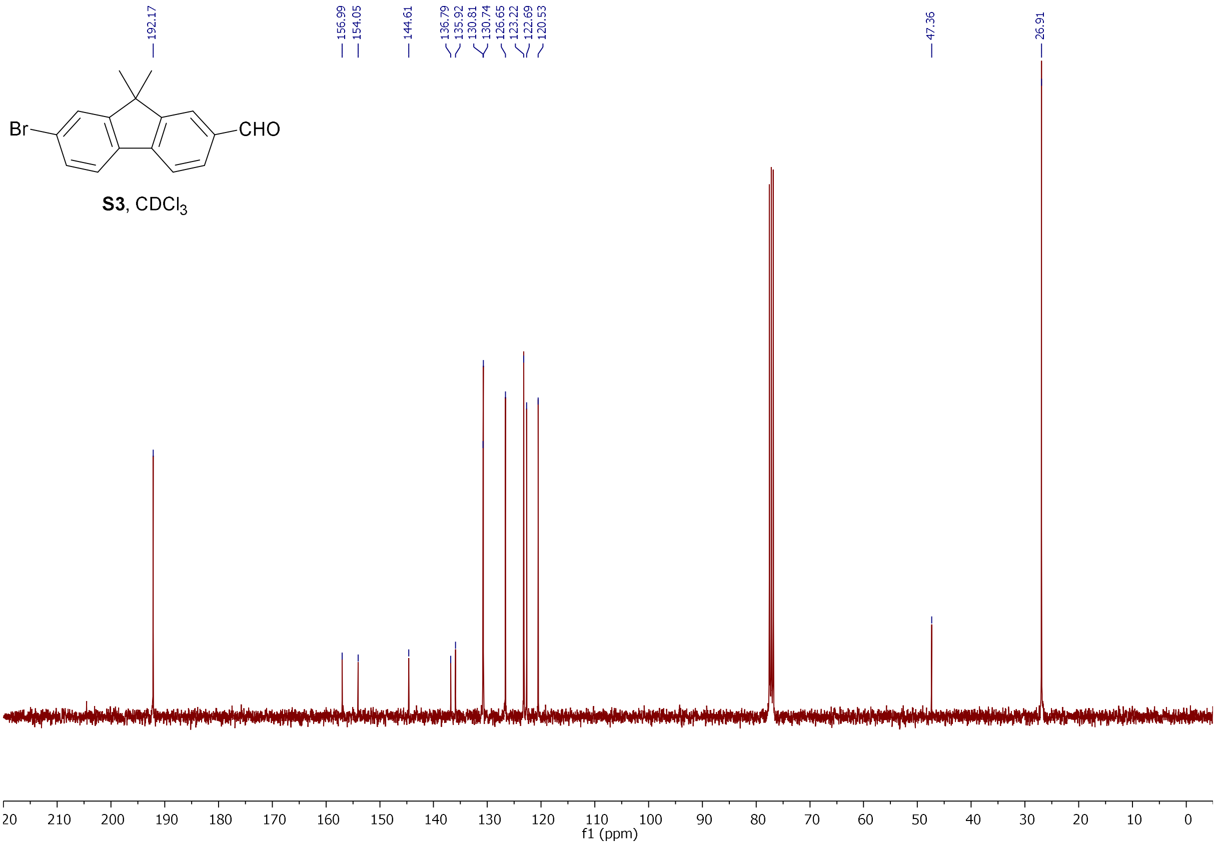
Br
CHO
S3, CDCl3
192.17
156.99 154.05
144.61
136.79 135.92 130.81 130.74 126.65 123.22 122.69 120.53
47.36
26.91
f1 (ppm)

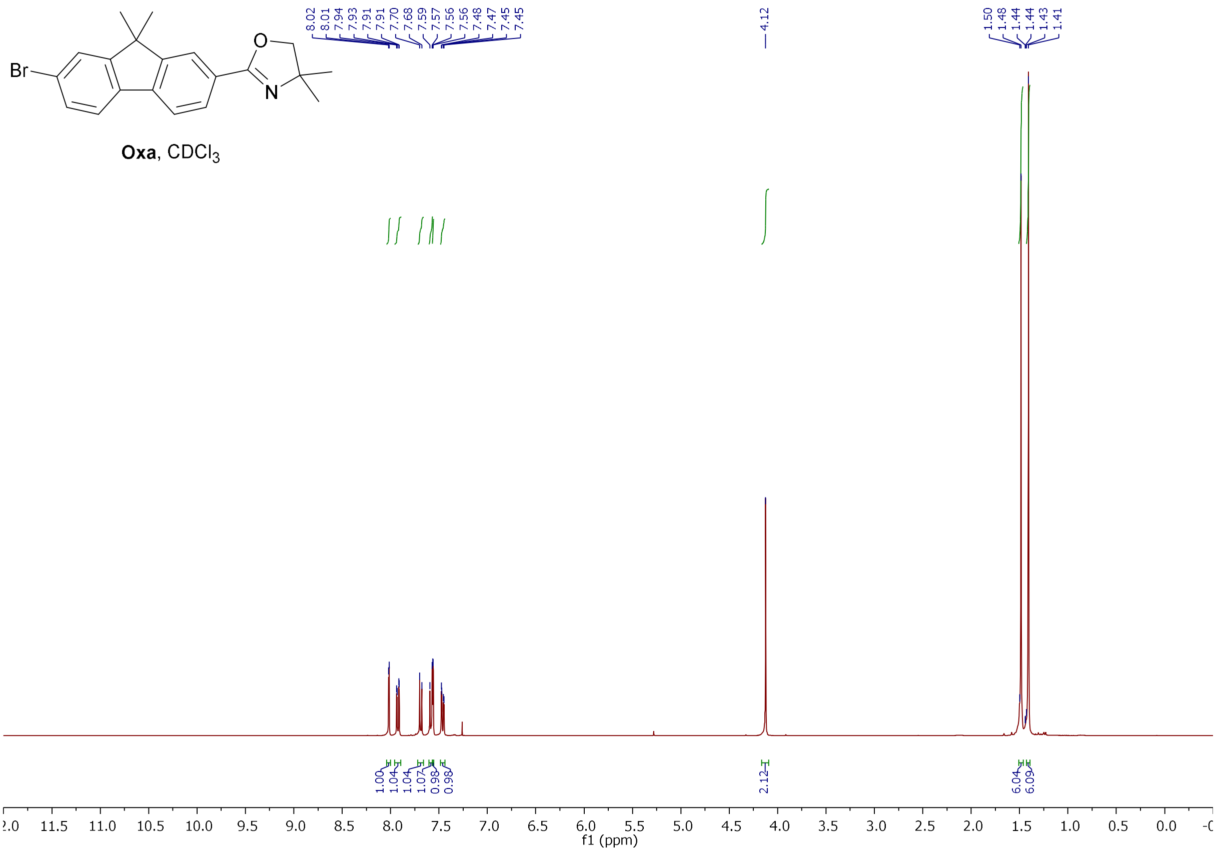
Br
O
N
Oxa, CDCl3
8.02
8.01
7.94
7.93
7.91
7.91
7.70
7.68
7.59
7.57
7.56
7.56
7.48
7.47
7.45
7.45
4.12
1.50
1.48
1.44
1.44
1.43
1.41
1.00
1.04
1.04
1.07
0.98
0.98
2.12
6.04
6.09
f1 (ppm)

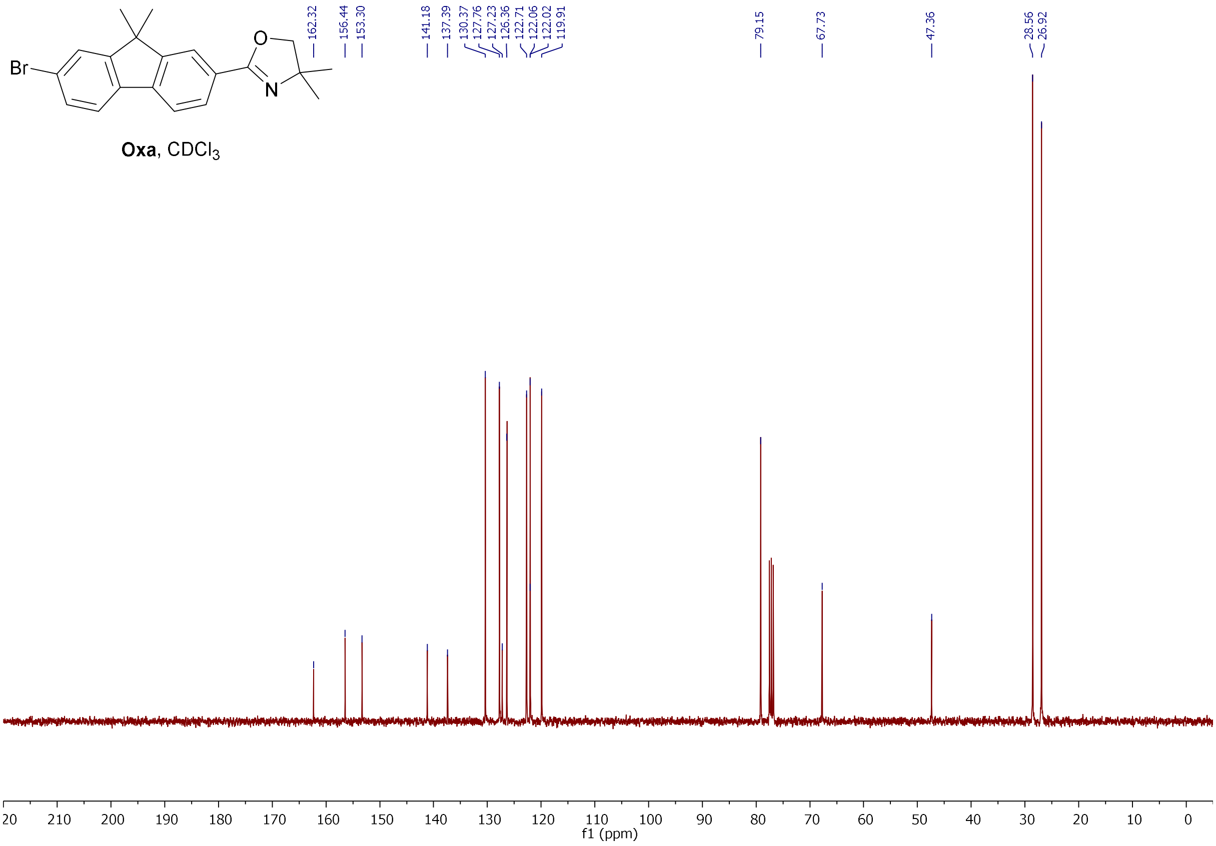
Br
O
N
Oxa, CDCl3
162.32
156.44
153.30
141.18
137.39
130.37
127.76
127.23
126.36
122.71
122.06
122.02
119.91
79.15
67.73
47.36
28.56
26.92
f1 (ppm)

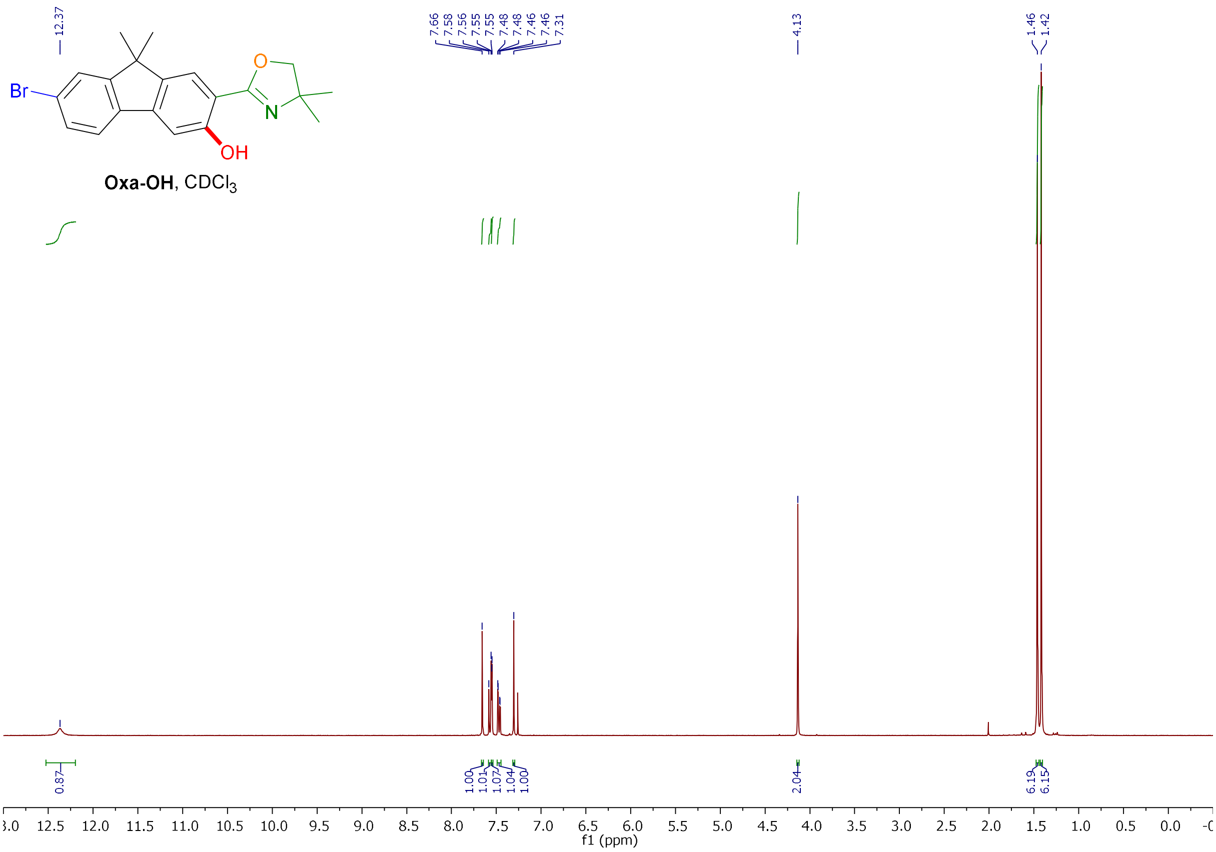
Br
O
N
OH
Oxa-OH, CDCl3
12.37
7.66 7.58 7.56 7.55 7.55 7.48 7.48 7.46 7.46 7.31
4.13
1.46 1.42
0.87
1.00 1.01 1.07 1.04 1.00
2.04
6.19 6.15
f1 (ppm)

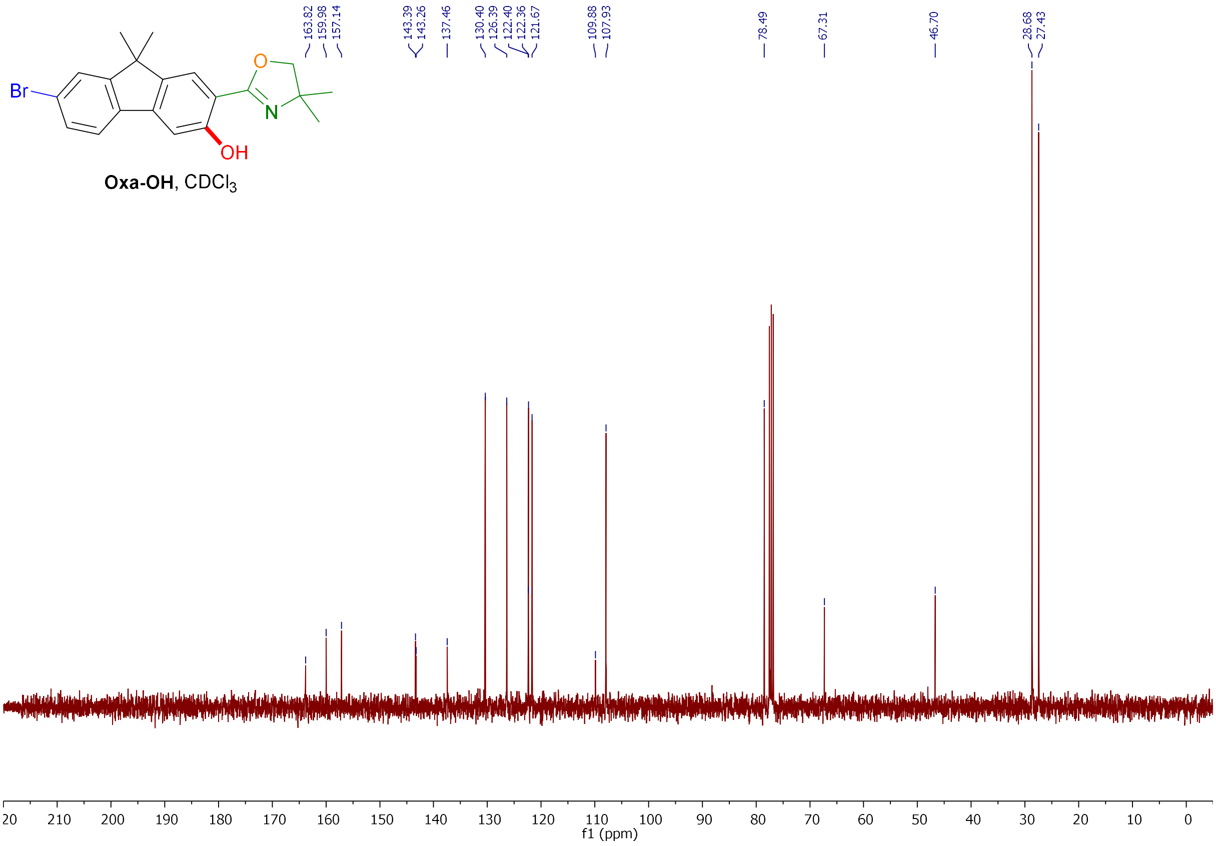
Br
O
N
OH
Oxa-OH, CDCl3
163.82 159.98 157.14
143.39 143.26
137.46
130.40 126.39 122.40 122.36 121.67
109.88 107.93
78.49
67.31
46.70
28.68 27.43
f1 (ppm)

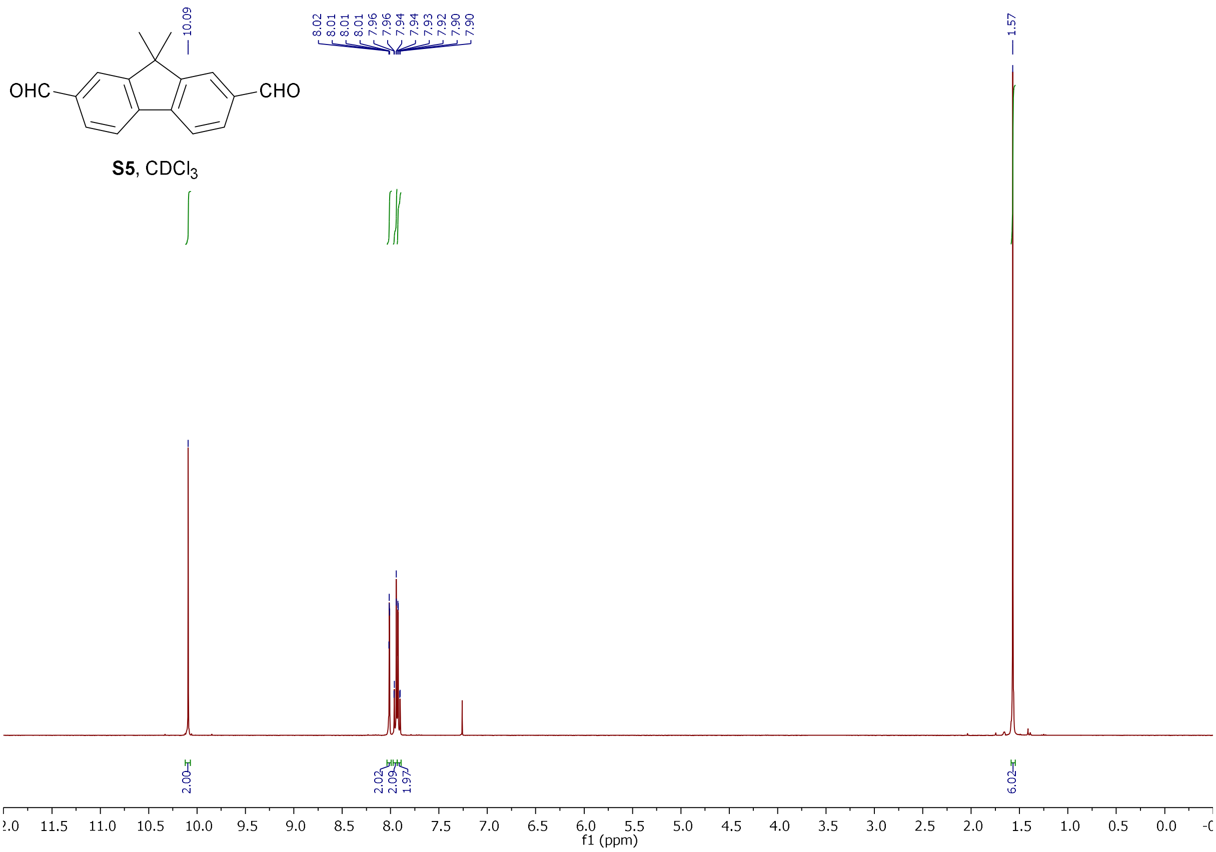
OHC
CHO
S5, CDCl3
10.09
8.02 8.01 8.01 8.01 7.96 7.96 7.94 7.94 7.93 7.92 7.90 7.90
1.57
2.00
2.02 2.09 1.97
6.02
f1 (ppm)

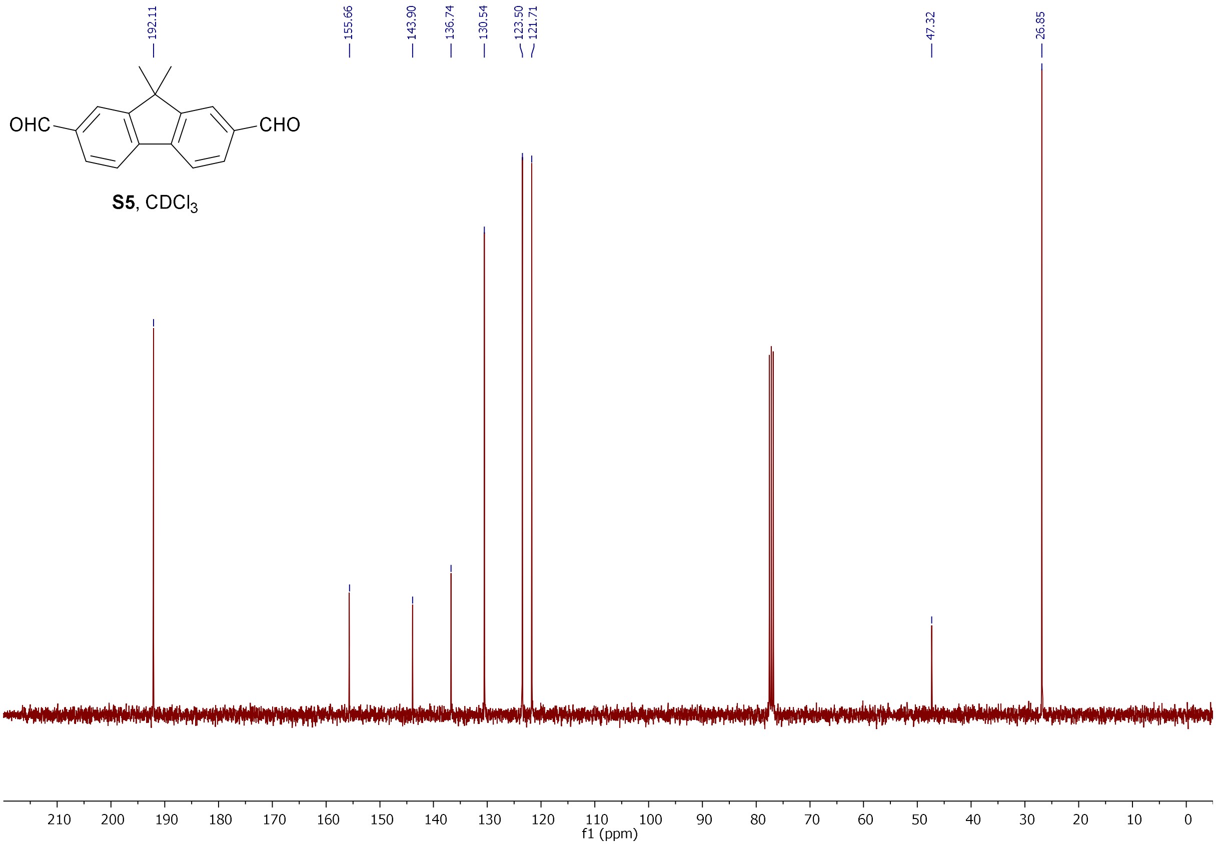
OHC
CHO
S5, CDCl3
192.11
155.66
143.90
136.74
130.54
123.50
121.71
47.32
26.85
f1 (ppm)

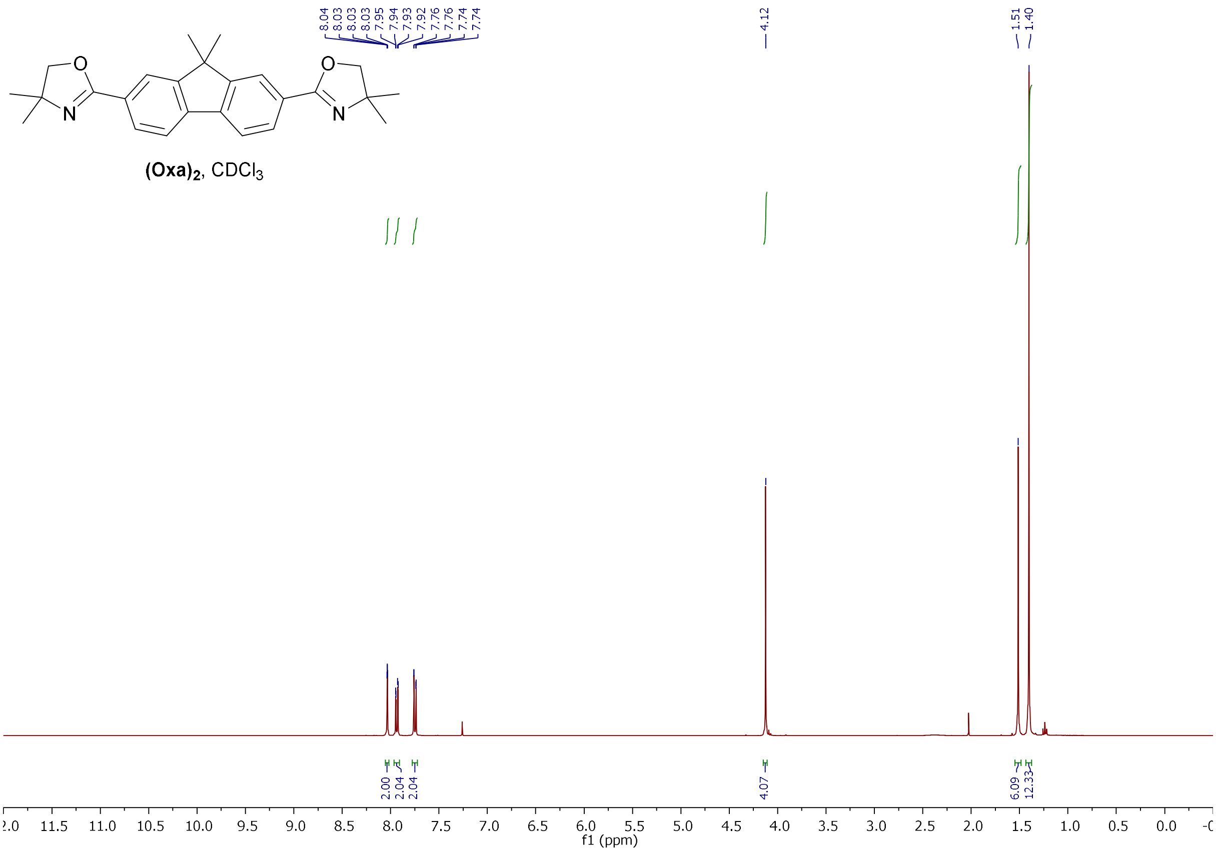
(Oxa)$_2$, CDCl$_3$
8.04 8.03 8.03 8.03 7.95 7.94 7.93 7.92 7.76 7.76 7.74 7.74
4.12
1.51 1.40
2.00 2.04 2.04
4.07
6.09 12.33
f1 (ppm)

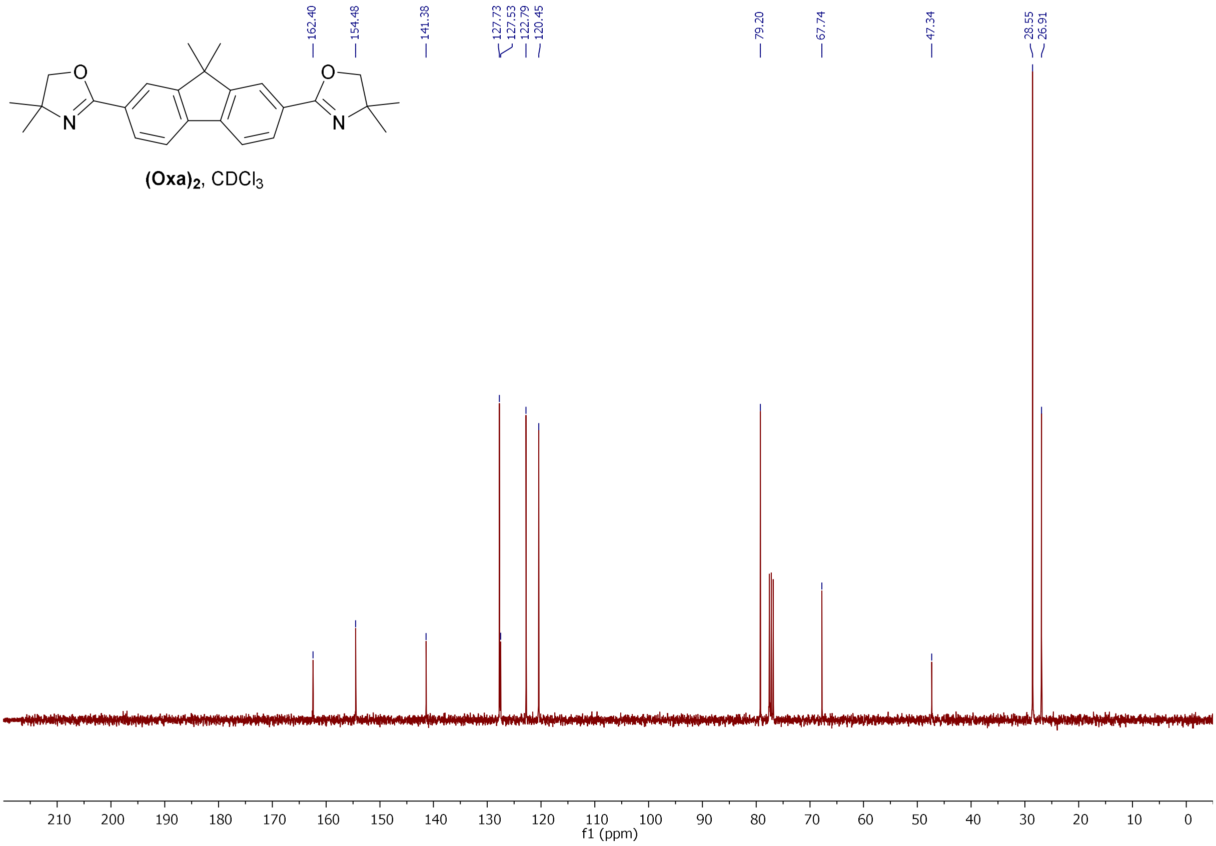
(Oxa)$_2$, CDCl$_3$
162.40
154.48
141.38
127.73 127.53 122.79 120.45
79.20
67.74
47.34
28.55 26.91
f1 (ppm)

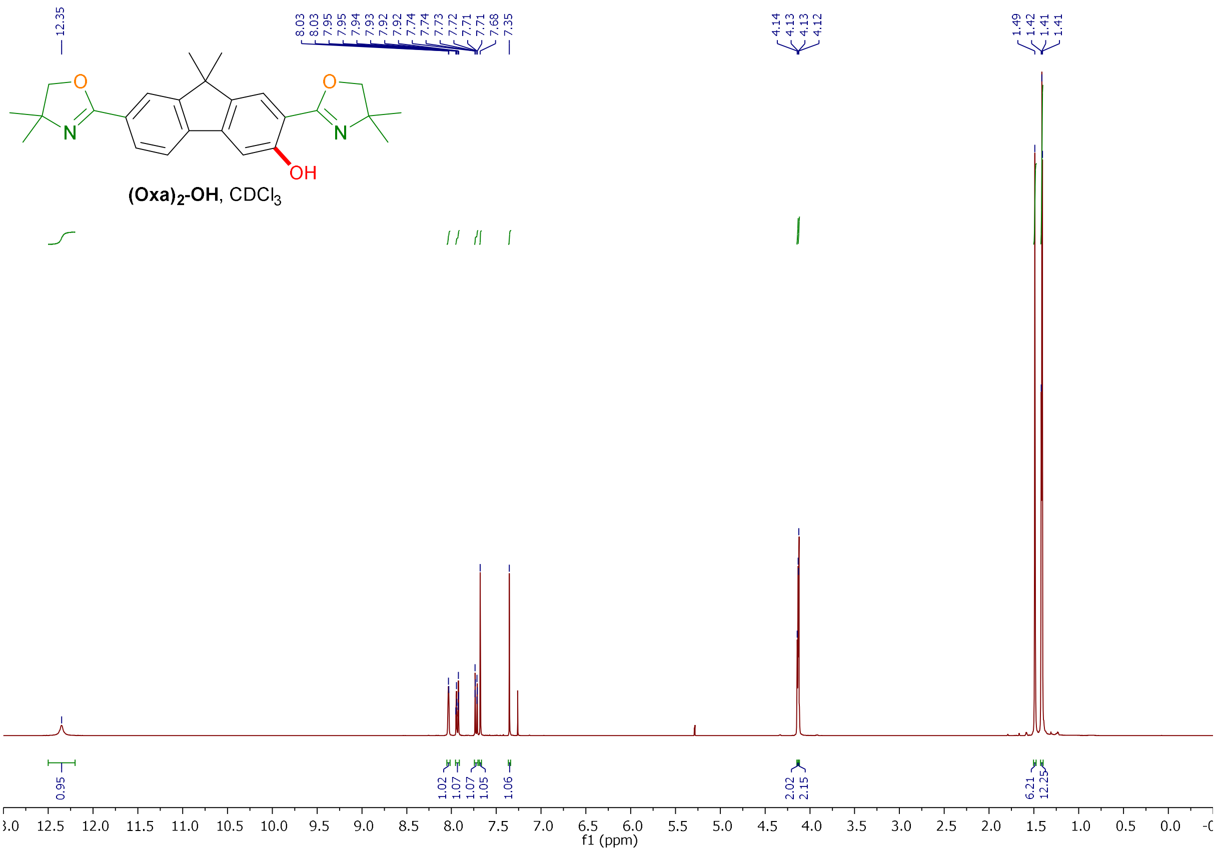
12.35
8.03 8.03 7.95 7.95 7.94 7.93 7.92 7.92 7.74 7.74 7.73 7.72 7.71 7.71 7.68 7.35
4.14 4.13 4.13 4.12
1.49 1.42 1.41 1.41
(Oxa)2-OH, CDCl3
0.95
1.02 1.07 1.07 1.05 1.06
2.02 2.15
6.21 12.25
f1 (ppm)

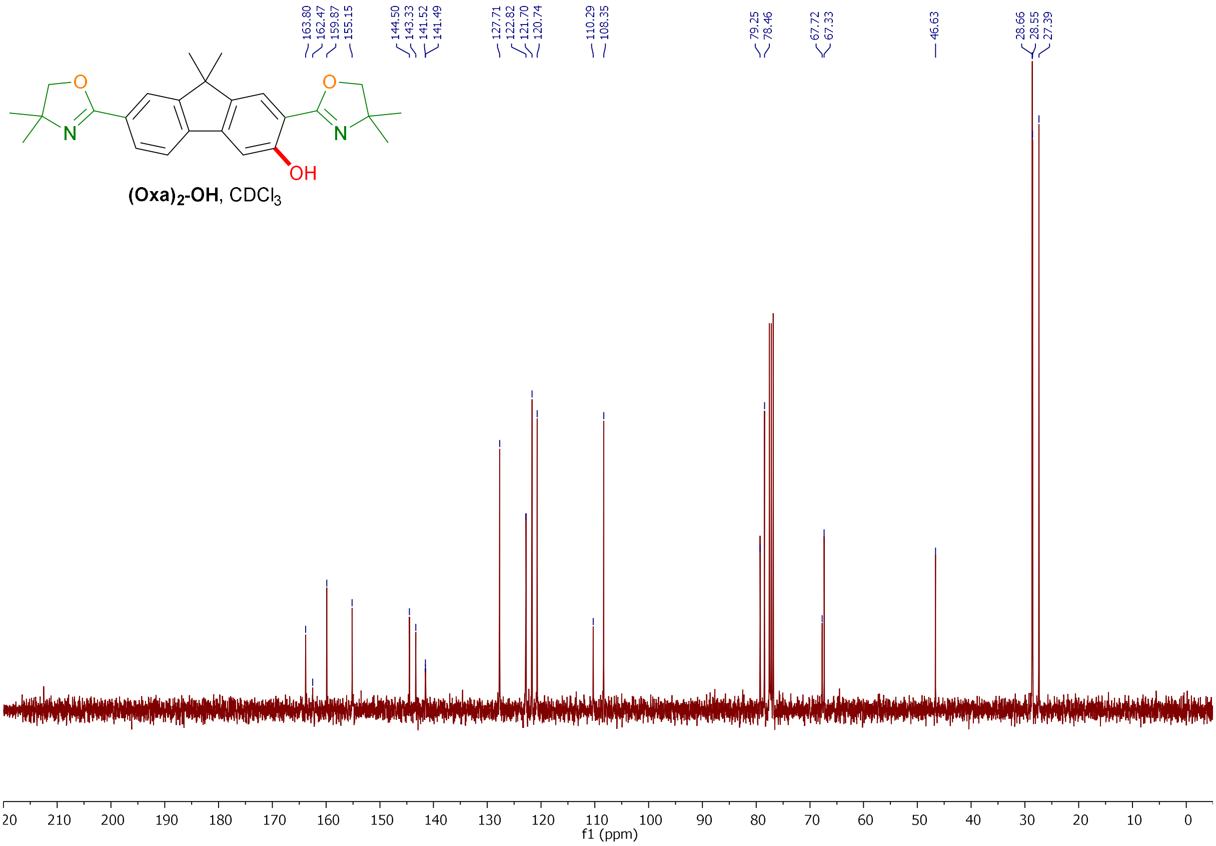
163.80 162.47 159.87 155.15
144.50 143.33 141.52 141.49
127.71 122.82 121.70 120.74
110.29 108.35
79.25 78.46
67.72 67.33
46.63
28.66 28.55 27.39
(Oxa)2-OH, CDCl3
f1 (ppm)

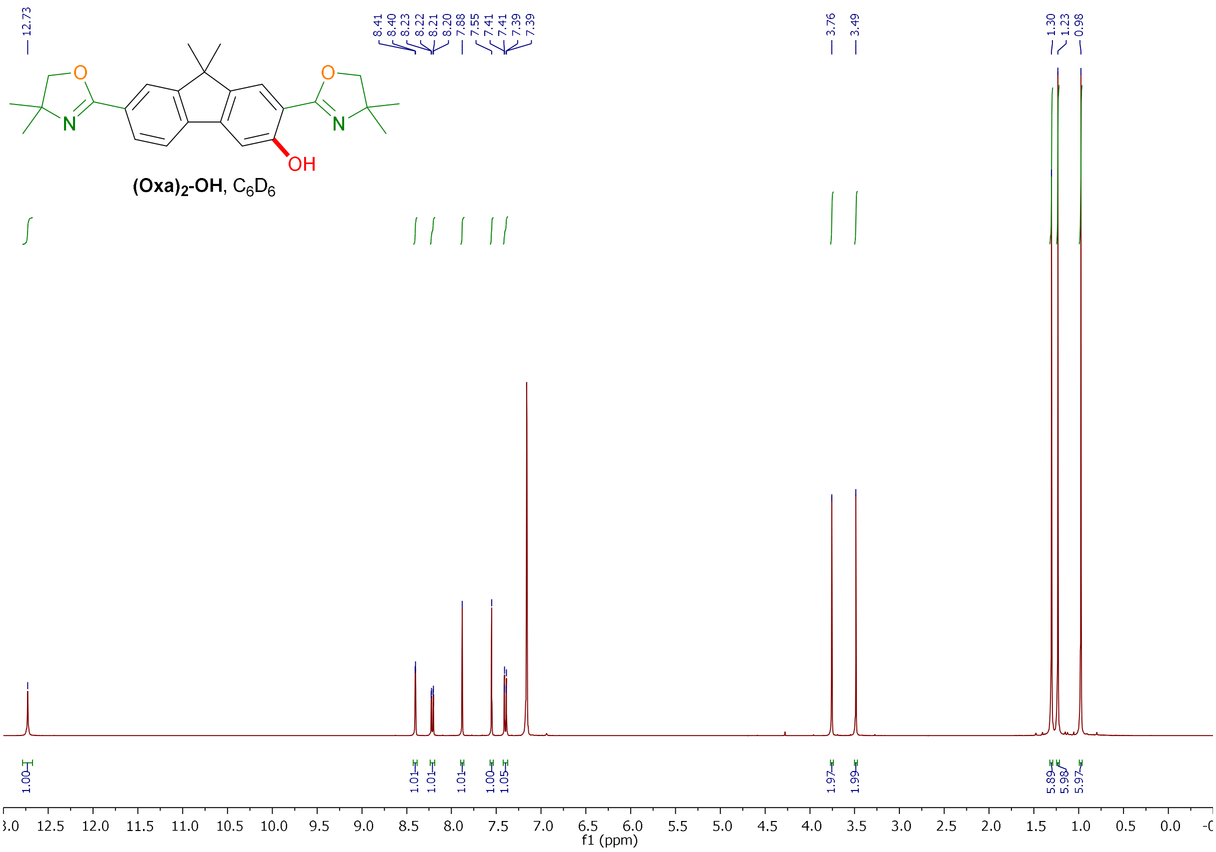
(Oxa)2-OH, C6D6
f1 (ppm)

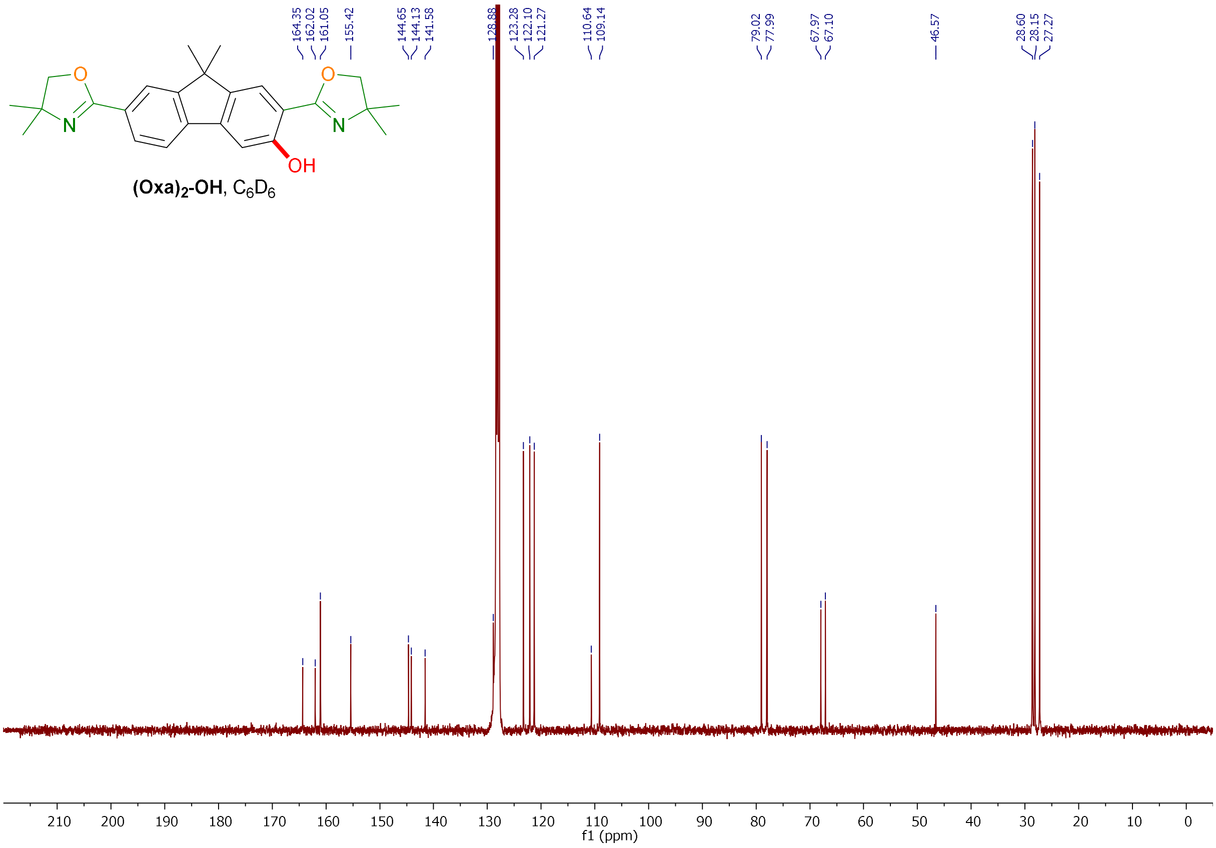
(Oxa)2-OH, C6D6
f1 (ppm)

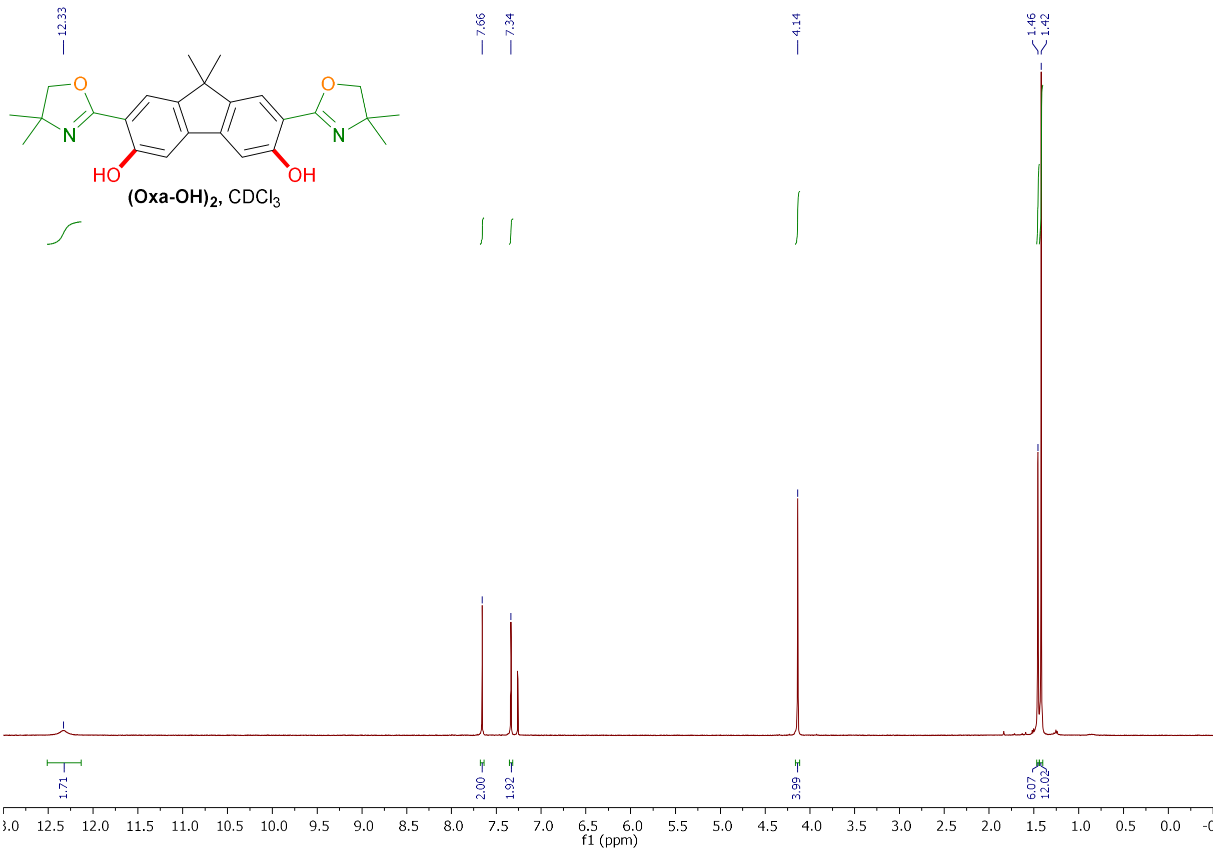
12.33
7.66
7.34
4.14
1.46
1.42
O
N
HO
OH
(Oxa-OH)$_2$, $CDCl_3$
1.71
2.00
1.92
3.99
6.07
12.02
f1 (ppm)

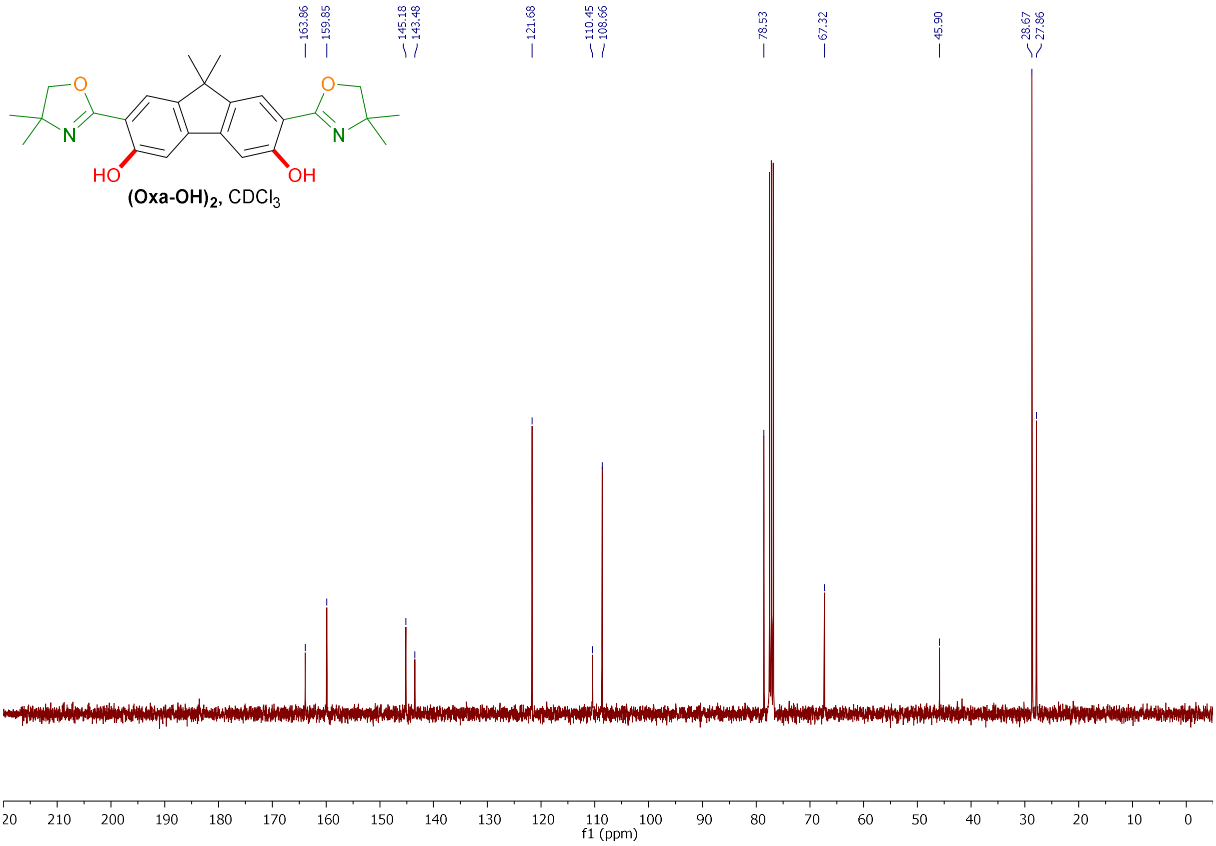
163.86
159.85
145.18
143.48
121.68
110.45
108.66
78.53
67.32
45.90
28.67
27.86
O
N
HO
OH
(Oxa-OH)$_2$, $CDCl_3$
f1 (ppm)

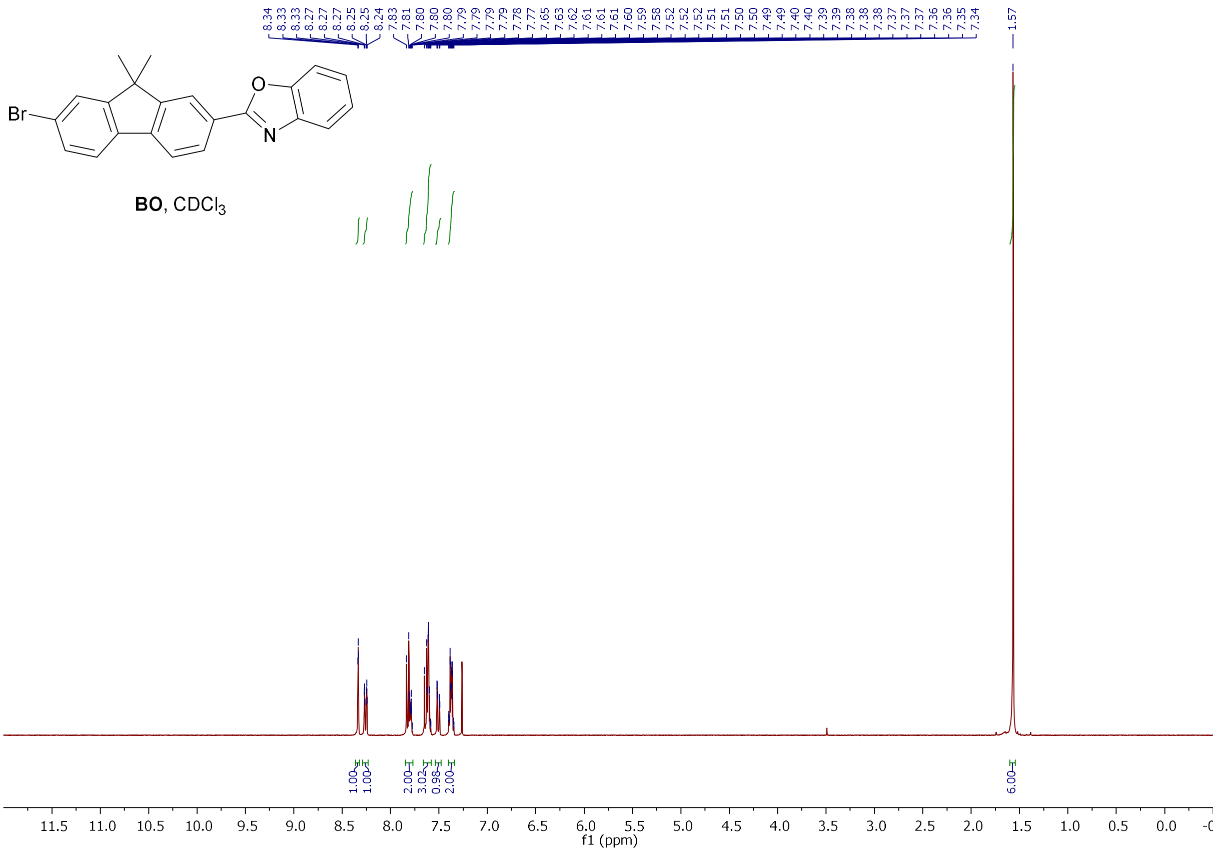
8.34 8.33 8.33 8.27 8.27 8.27 8.25 8.25 8.24 7.83 7.81 7.80 7.80 7.80 7.79 7.79 7.79 7.79 7.78 7.77 7.65 7.63 7.62 7.61 7.61 7.60 7.59 7.58 7.52 7.52 7.52 7.51 7.51 7.50 7.50 7.49 7.49 7.40 7.39 7.39 7.38 7.38 7.38 7.37 7.37 7.36 7.36 7.35 7.34
1.57
Br
O
N
BO, $CDCl_3$
1.00 1.00 2.00 3.02 0.98 2.00
6.00
f1 (ppm)

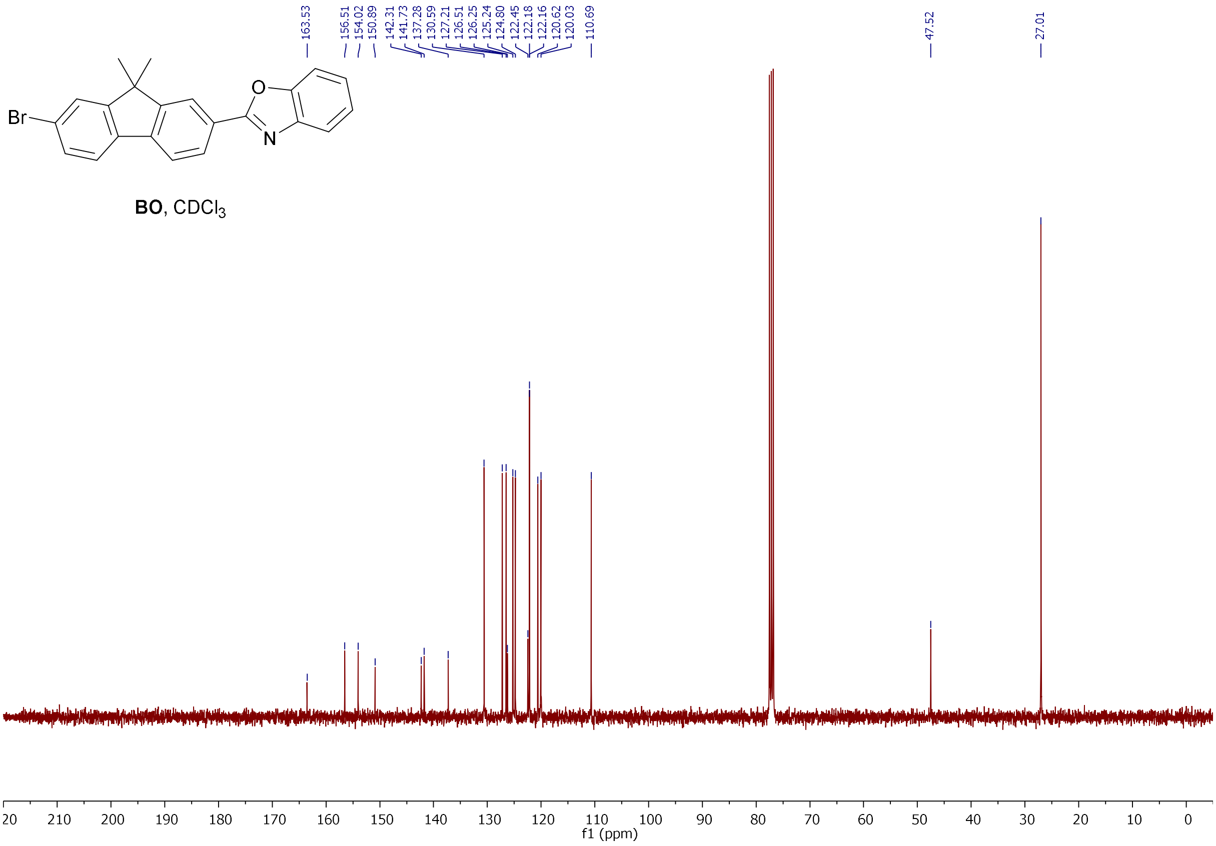
163.53
156.51 154.02 150.89
142.31 141.73 137.28 130.59 127.21 126.51 126.25 125.24 124.80 122.45 122.18 122.16 120.62 120.03
110.69
47.52
27.01
Br
O
N
BO, $CDCl_3$
f1 (ppm)

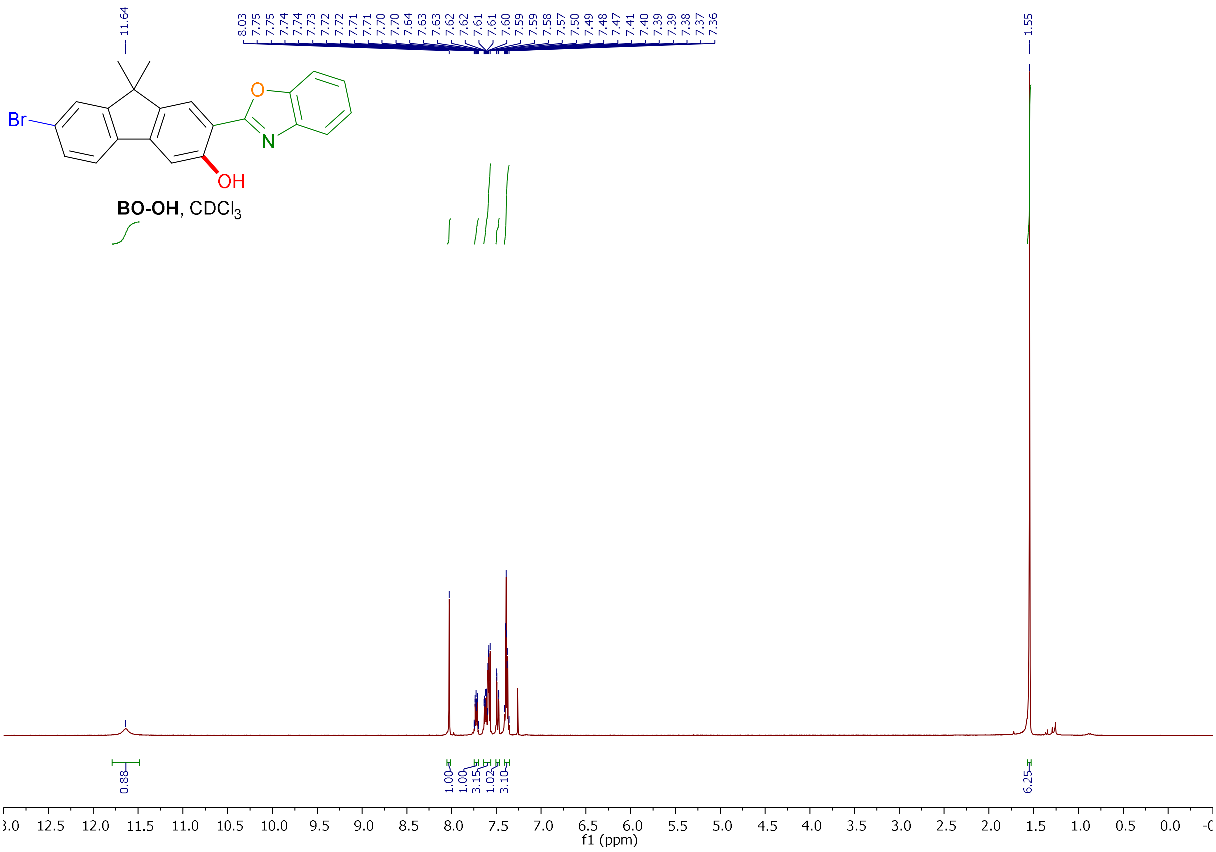
BO-OH, CDCl3
f1 (ppm)

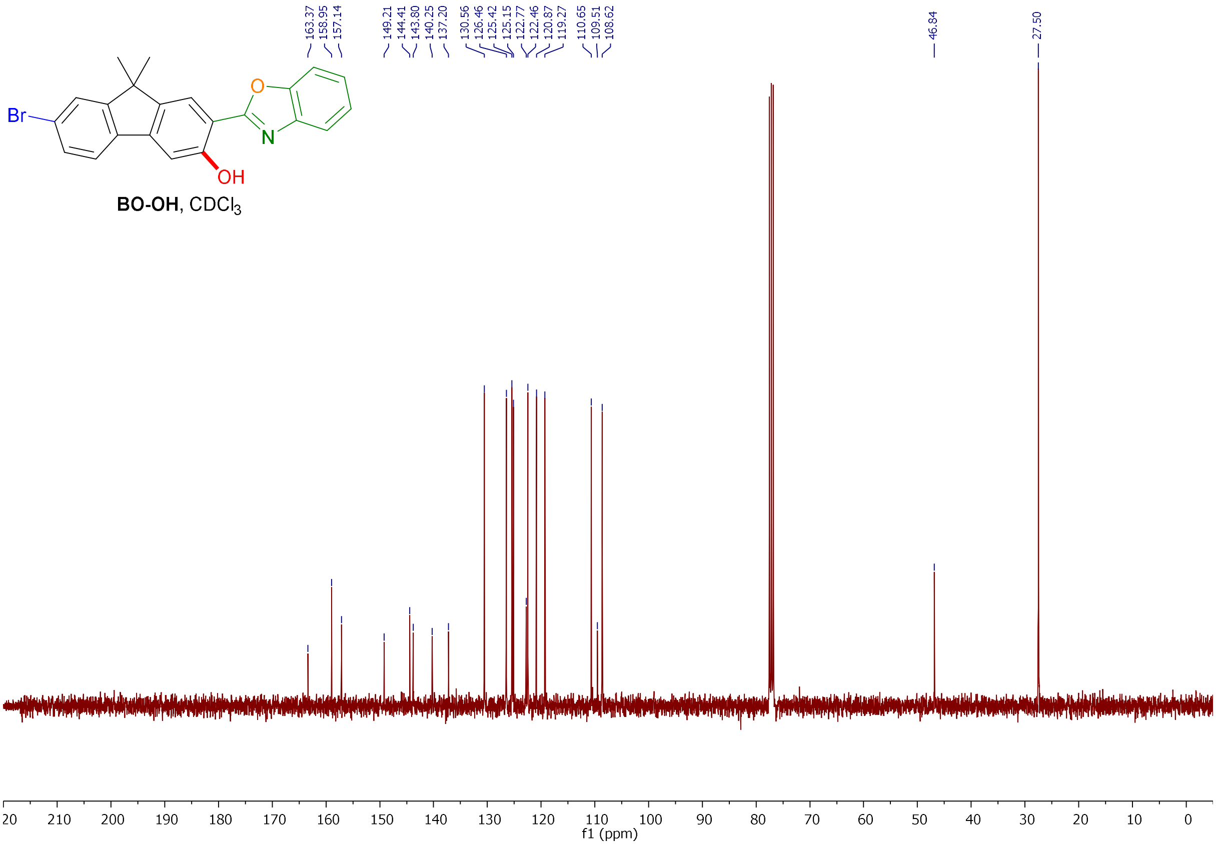
BO-OH, CDCl3
f1 (ppm)

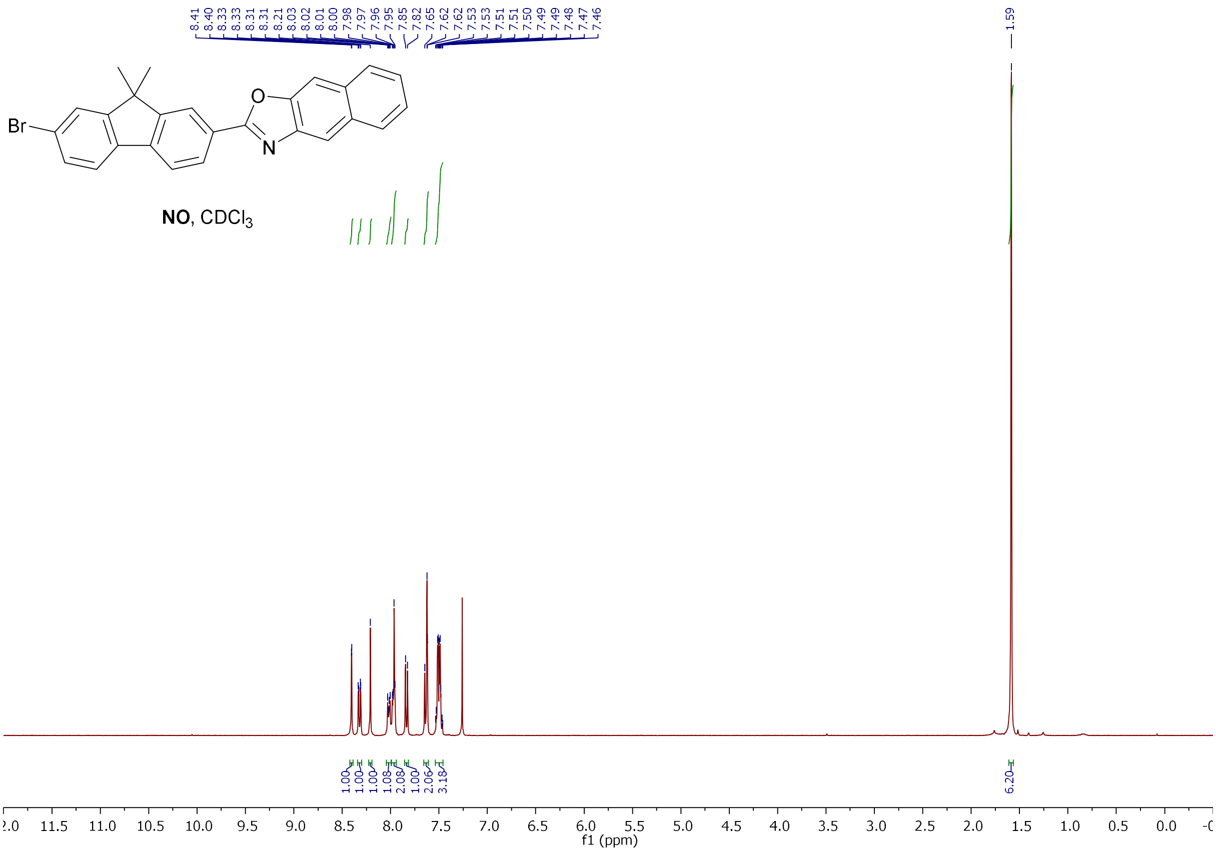
8.41
8.40
8.33
8.33
8.31
8.31
8.21
8.03
8.02
8.01
8.00
7.98
7.97
7.96
7.95
7.85
7.82
7.65
7.62
7.62
7.53
7.53
7.51
7.51
7.50
7.49
7.49
7.48
7.47
7.46
1.59
Br
O
N
NO, $CDCl_3$
1.00
1.00
1.00
1.08
2.08
1.00
2.06
3.18
6.20
f1 (ppm)

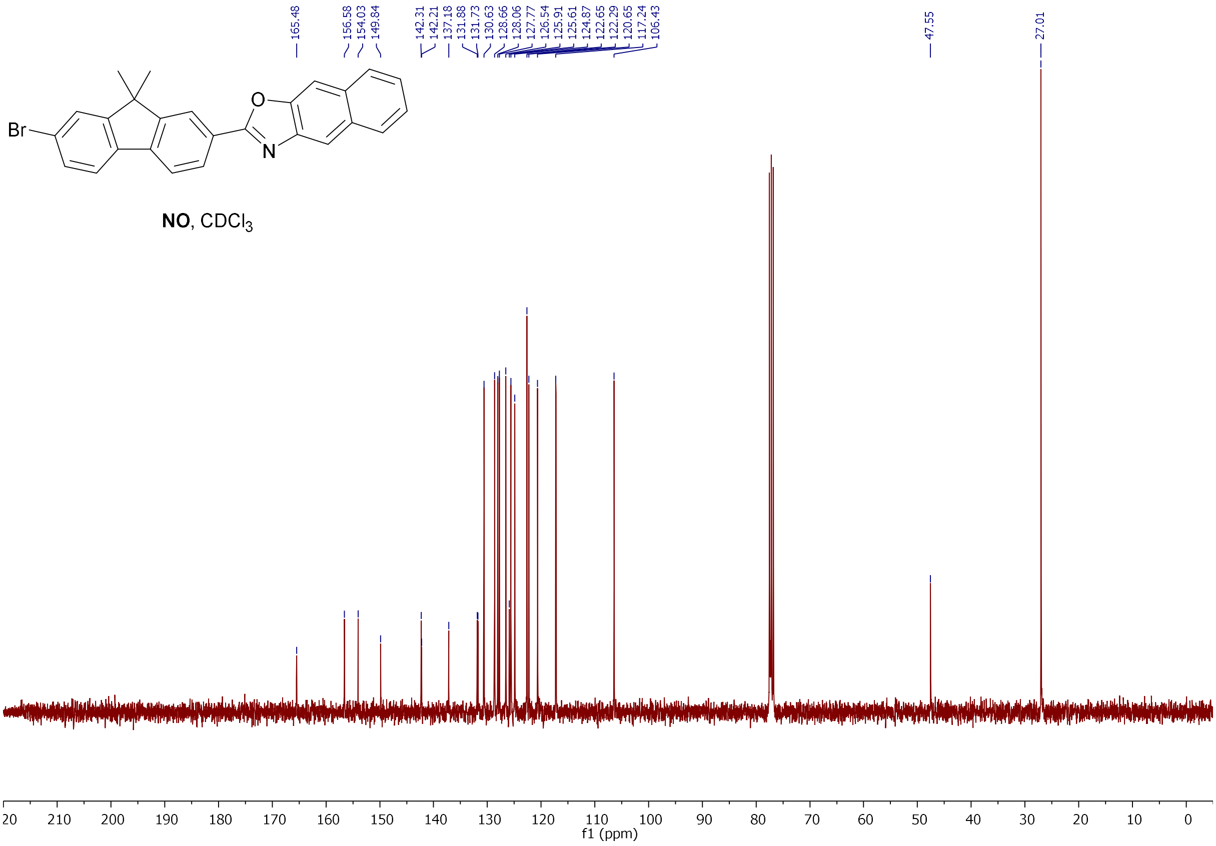
165.48
156.58
154.03
149.84
142.31
142.21
137.18
131.88
131.73
130.63
128.66
128.06
127.77
126.54
125.91
125.61
124.87
122.65
122.29
120.65
117.24
106.43
47.55
27.01
Br
O
N
NO, $CDCl_3$
f1 (ppm)

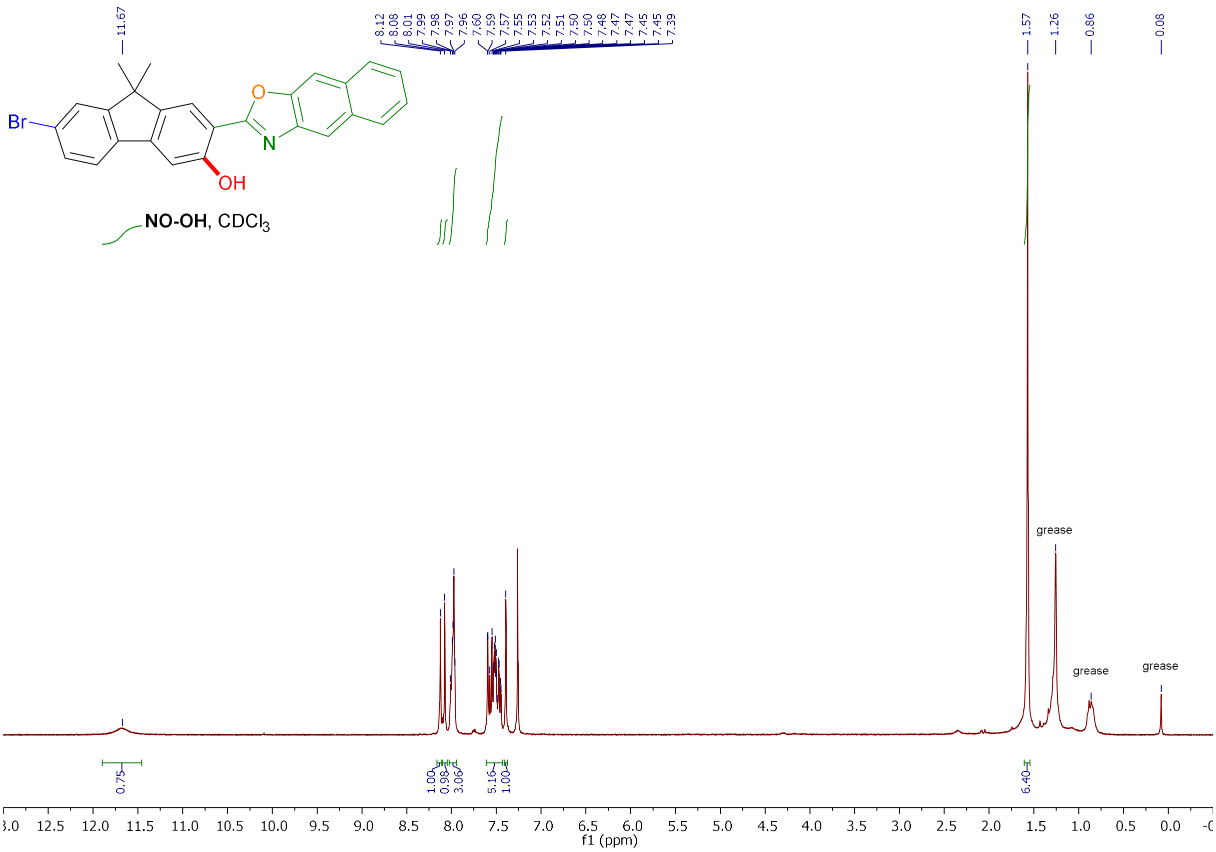
11.67
8.12
8.08
8.01
7.99
7.98
7.97
7.96
7.60
7.59
7.57
7.55
7.53
7.52
7.51
7.50
7.48
7.47
7.45
7.45
7.39
1.57
1.26
0.86
0.08
Br
O
N
OH
NO-OH, CDCl3
grease
grease
grease
0.75
1.00
0.98
3.06
5.16
1.00
6.40
f1 (ppm)

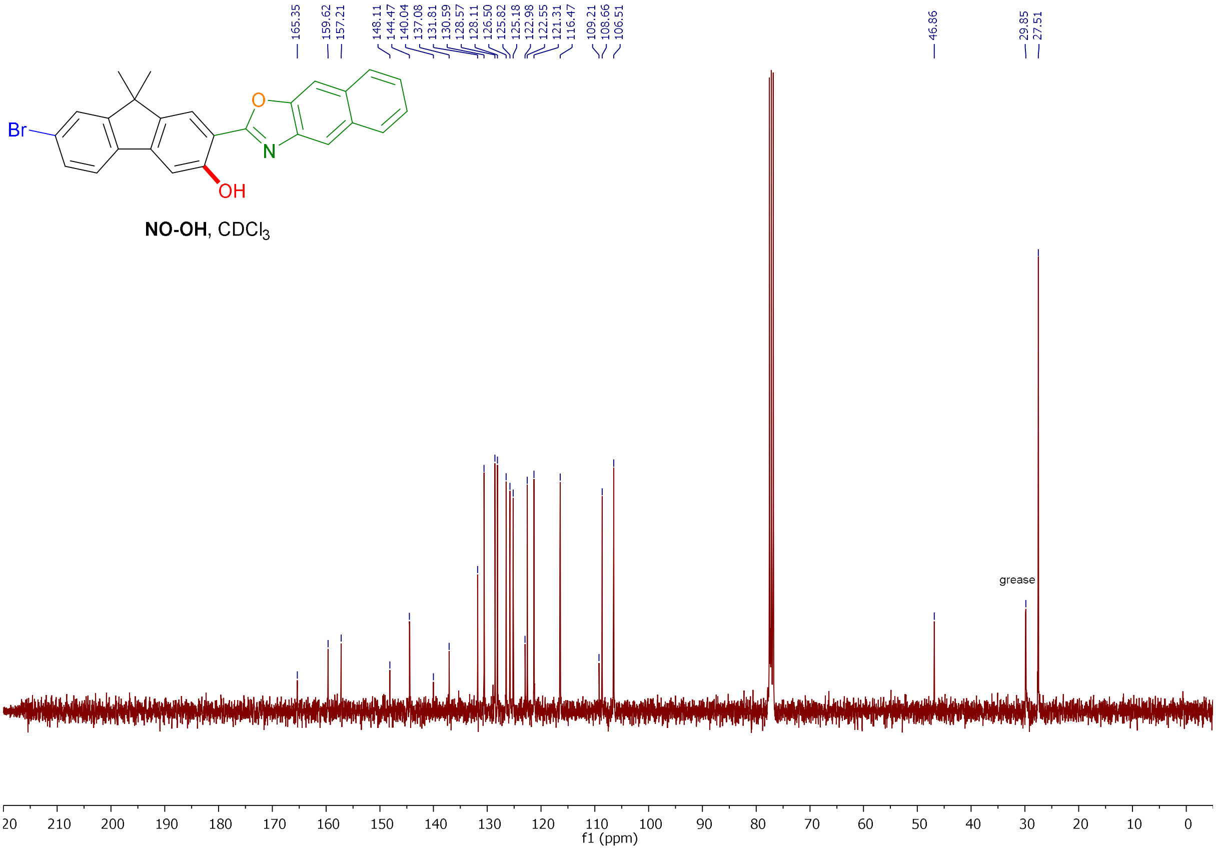
165.35
159.62
157.21
148.11
144.47
140.04
137.08
131.81
130.59
128.57
128.11
126.50
125.82
125.18
122.98
122.55
121.31
116.47
109.21
108.66
106.51
46.86
29.85
27.51
Br
O
N
OH
NO-OH, CDCl3
grease
f1 (ppm)

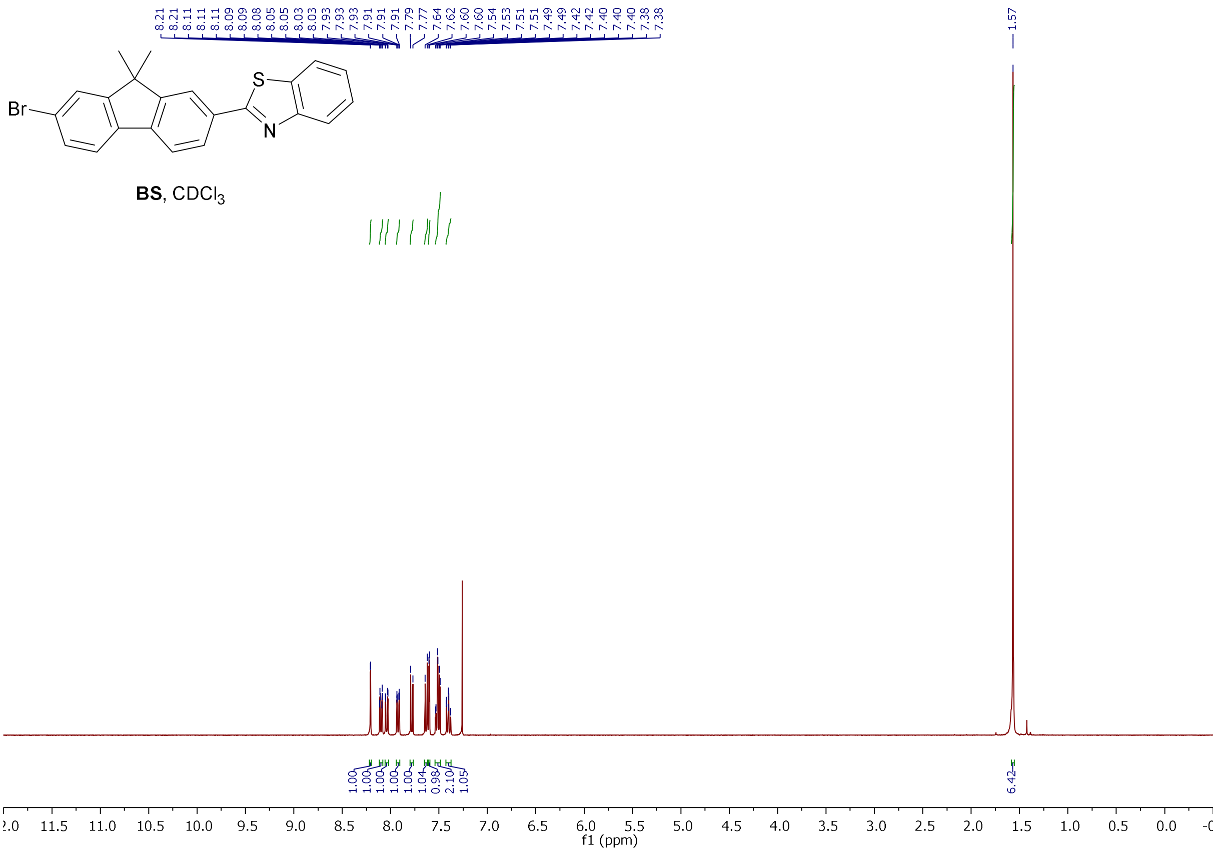

BS, $CDCl_3$

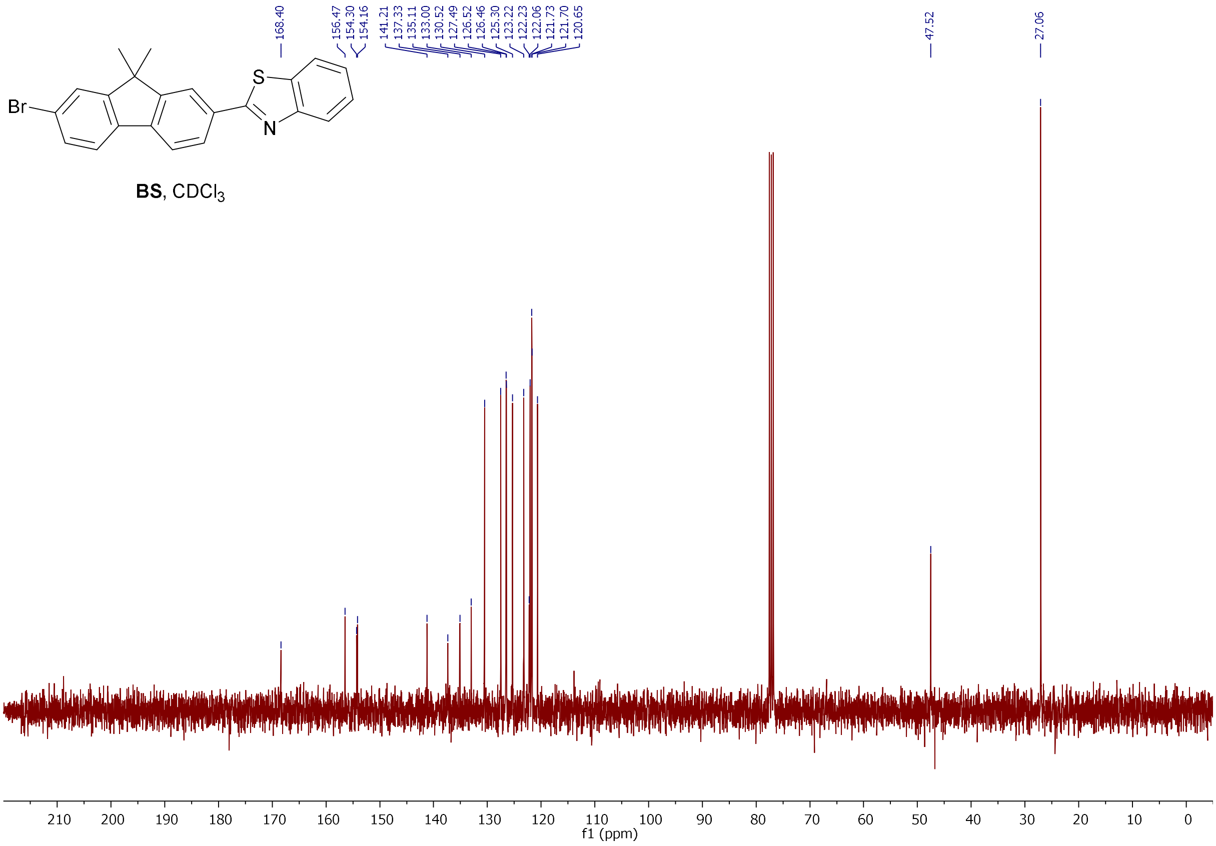

BS, $CDCl_3$

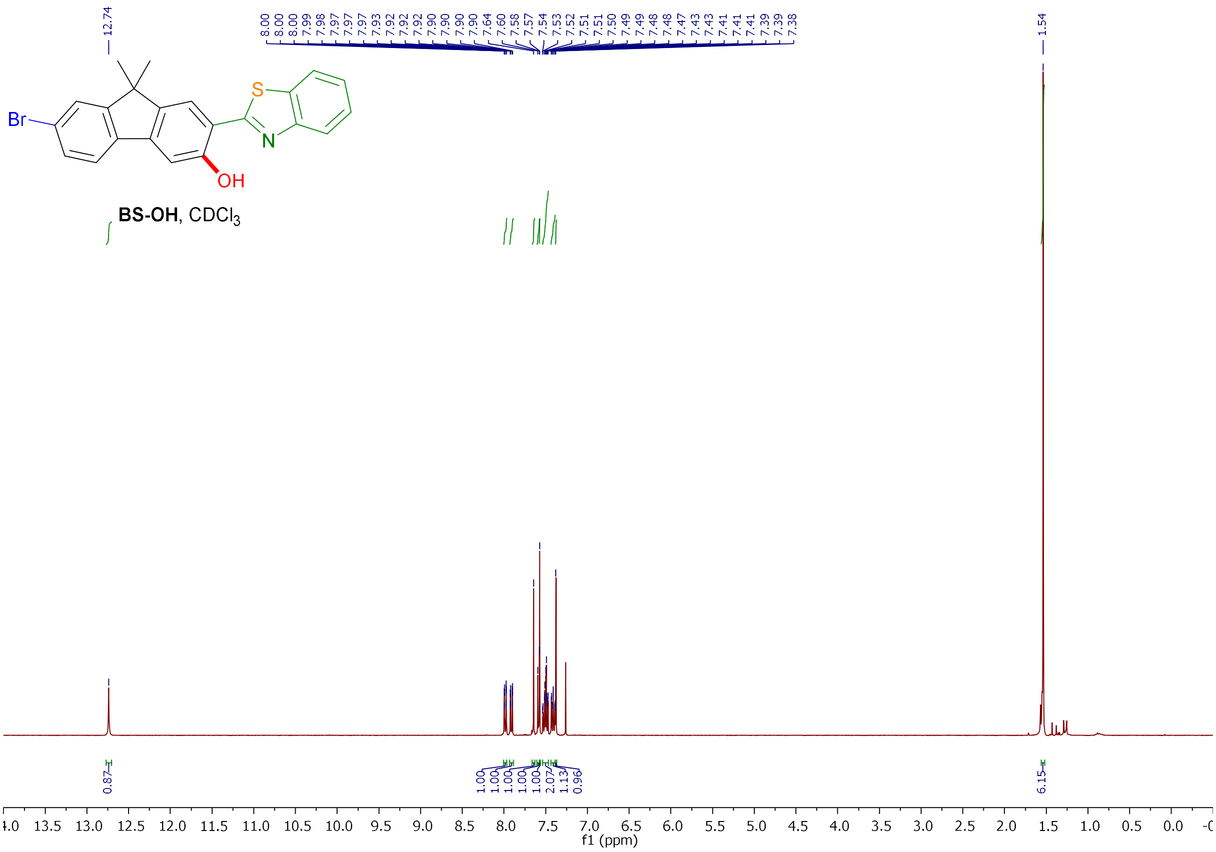

BS-OH, CDCl3
12.74
8.00
8.00
8.00
7.99
7.98
7.97
7.97
7.97
7.97
7.93
7.92
7.92
7.92
7.90
7.90
7.90
7.90
7.64
7.60
7.58
7.57
7.54
7.53
7.52
7.51
7.51
7.50
7.49
7.49
7.48
7.48
7.47
7.43
7.43
7.41
7.41
7.41
7.39
7.39
7.38
1.54
0.87
1.00
1.00
1.00
1.00
1.00
2.07
1.13
0.96
6.15
f1 (ppm)

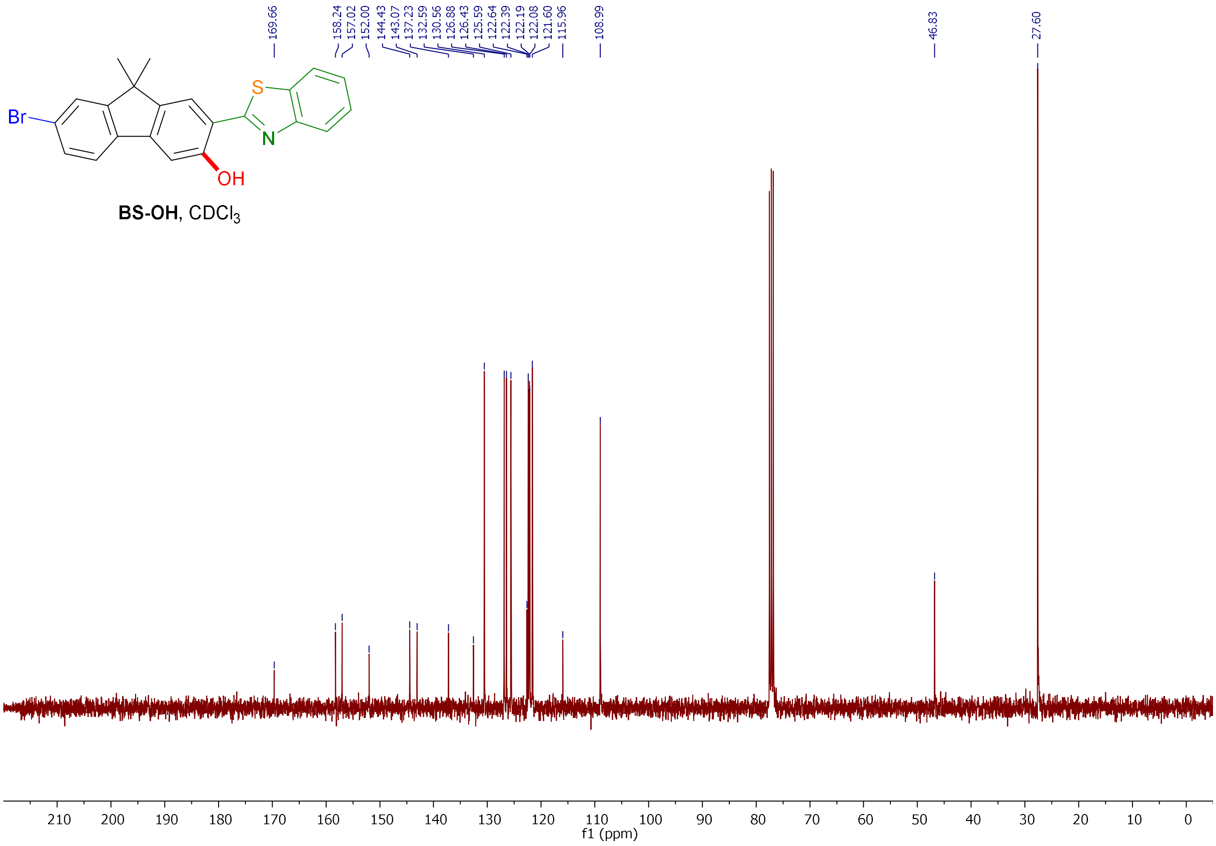

BS-OH, CDCl3
169.66
158.24
157.02
152.00
144.43
143.07
137.23
132.59
130.56
126.88
126.43
125.59
122.64
122.39
122.19
122.08
121.60
115.96
108.99
46.83
27.60
f1 (ppm)

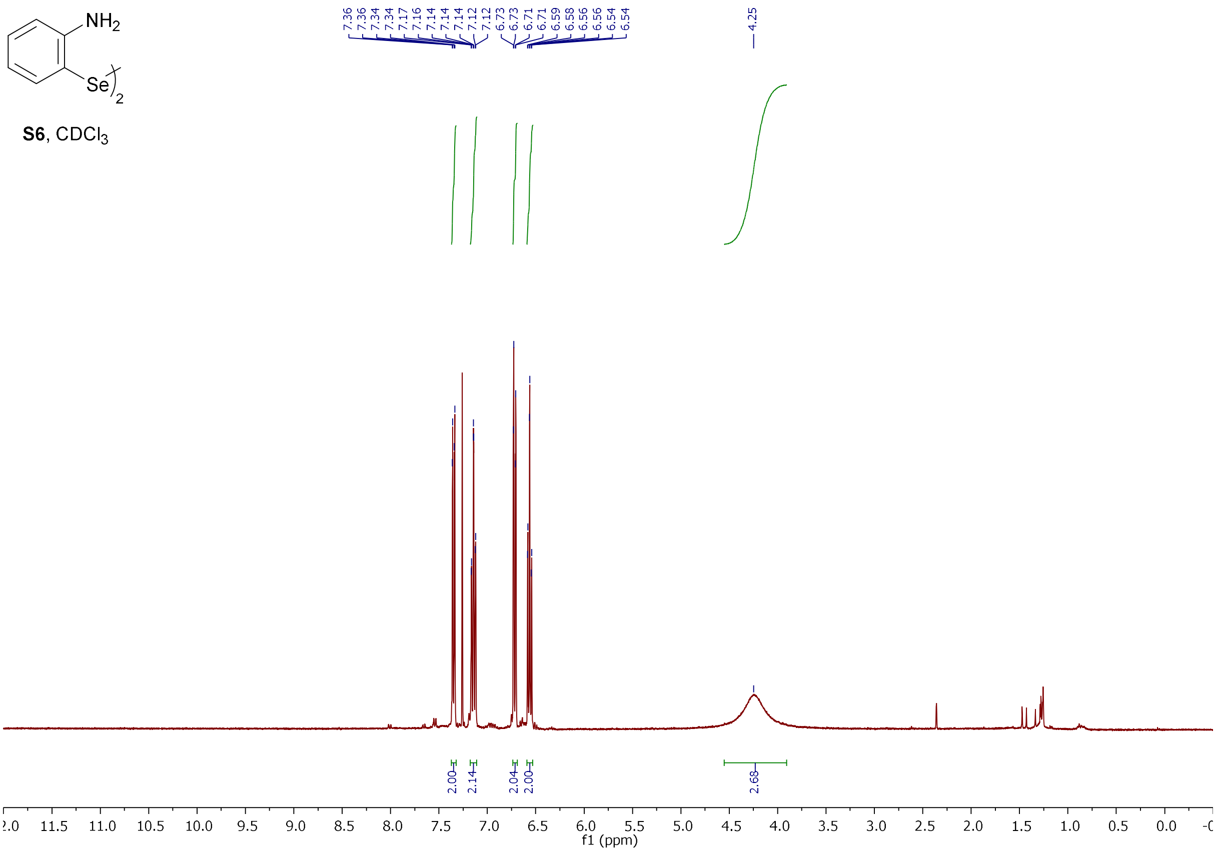

S6, $CDCl_3$

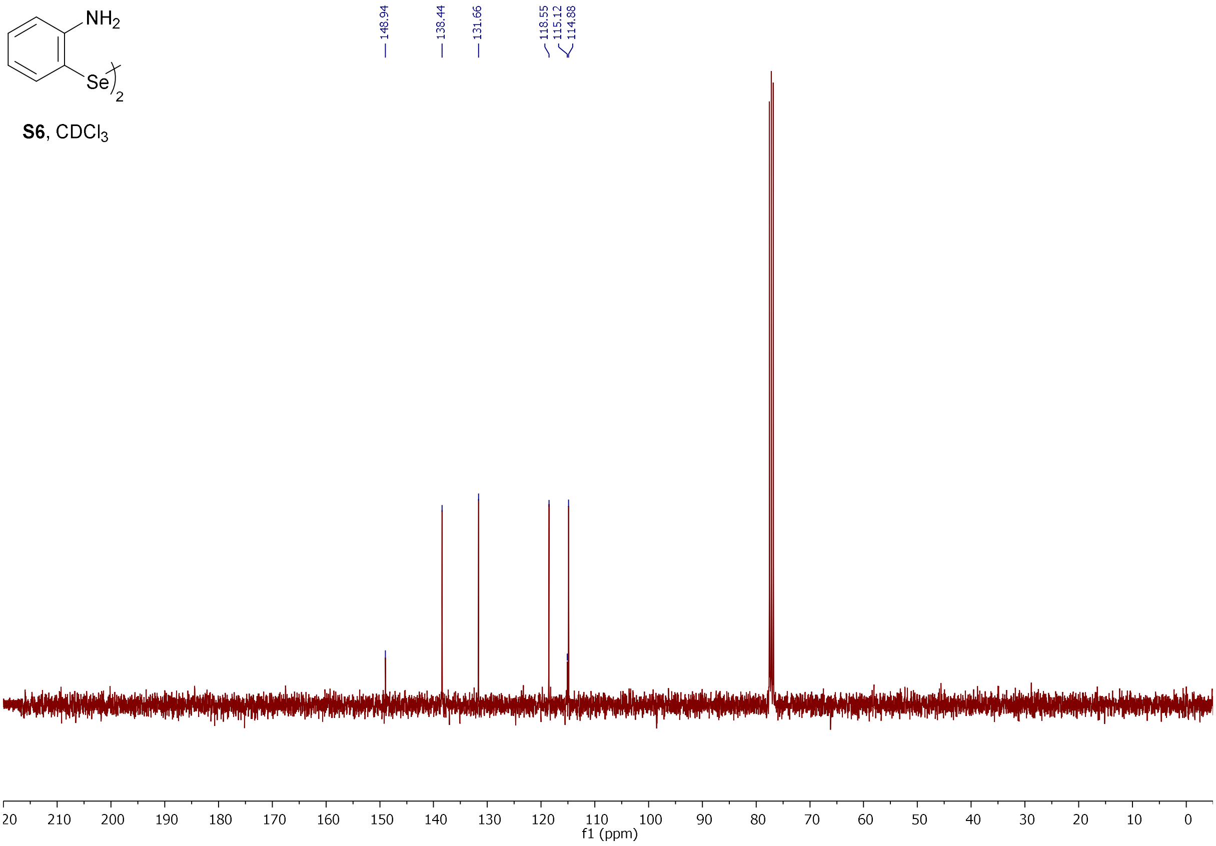

S6, $CDCl_3$

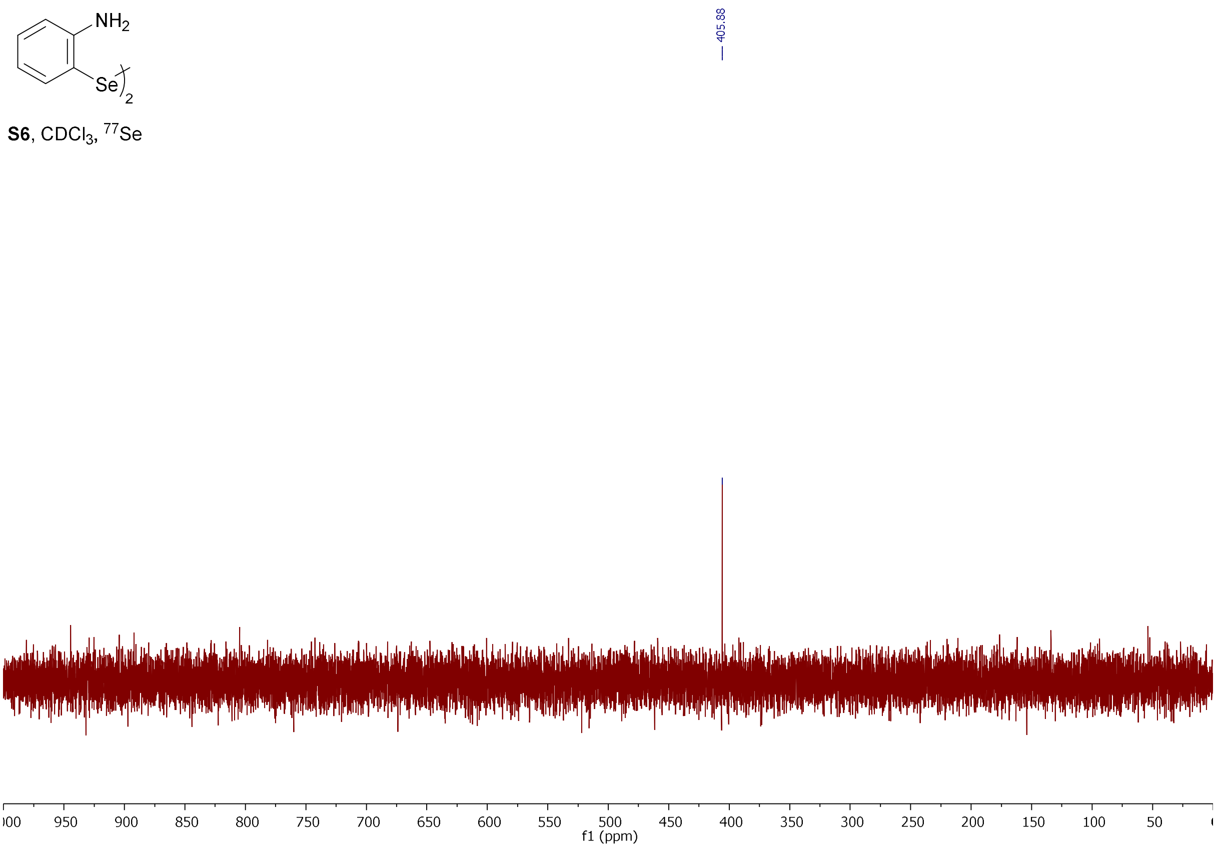
NH2
Se
S6, CDCl3, 77Se
405.88
f1 (ppm)

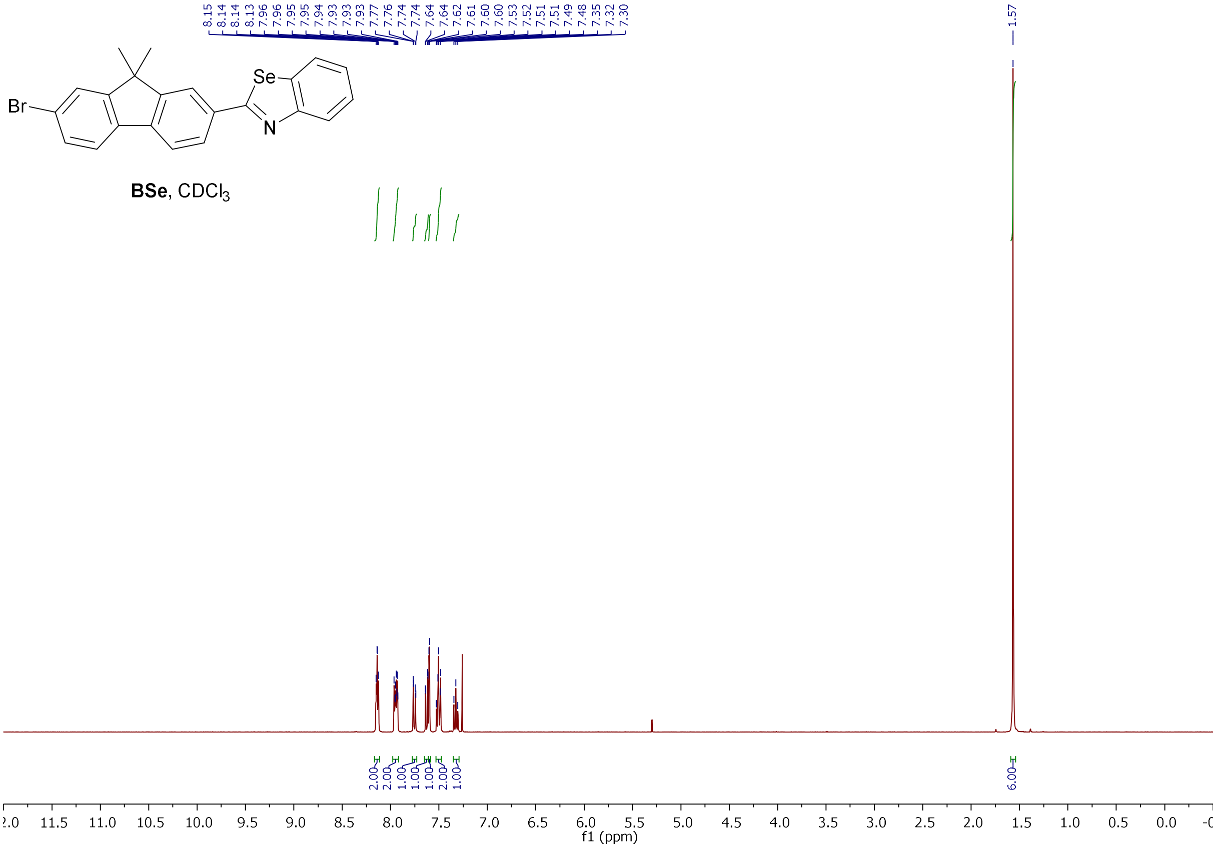
8.15 8.14 8.14 8.13 7.96 7.95 7.95 7.94 7.93 7.93 7.93 7.77 7.76 7.74 7.74 7.64 7.64 7.62 7.61 7.60 7.60 7.53 7.52 7.51 7.51 7.49 7.48 7.35 7.32 7.30
1.57
Br
Se
N
BSe, CDCl3
2.00 2.00 1.00 1.00 1.00 2.00 1.00
6.00
f1 (ppm)

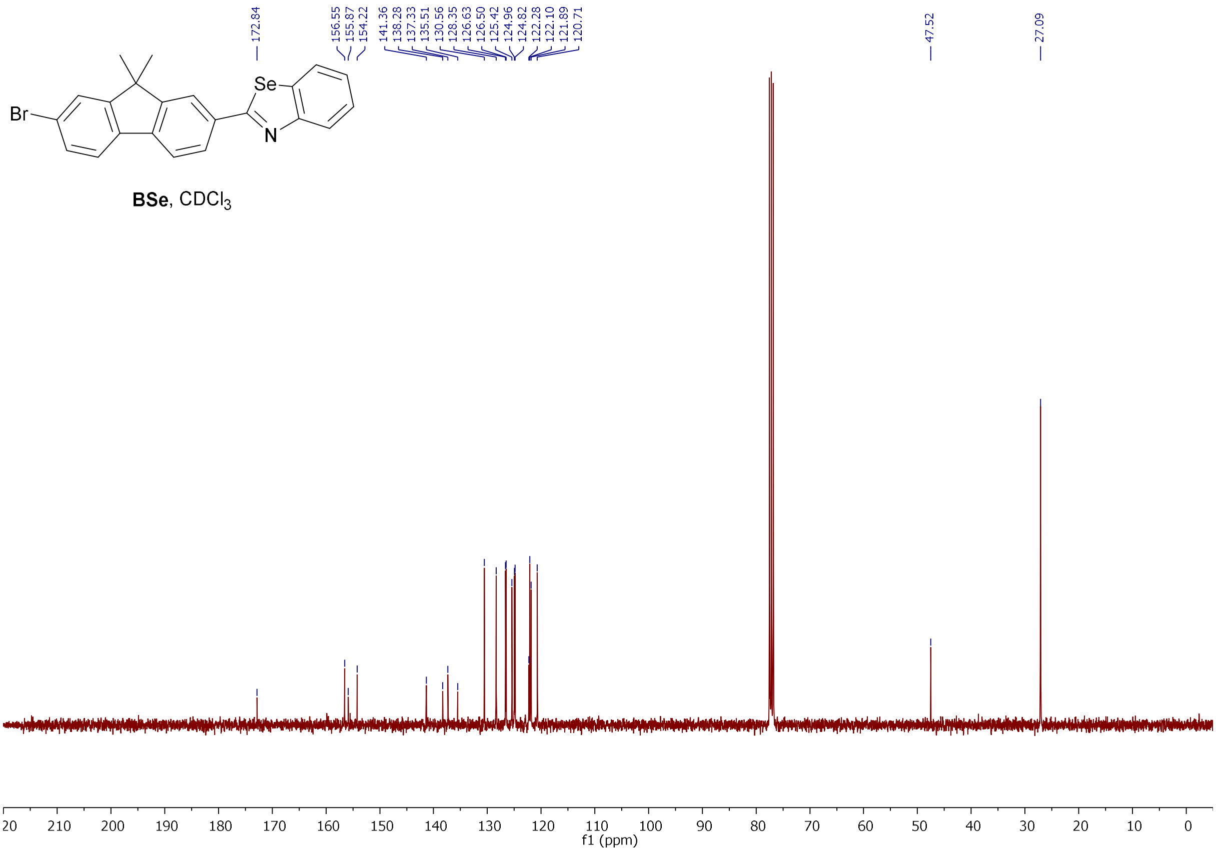

BSe, $CDCl_3$

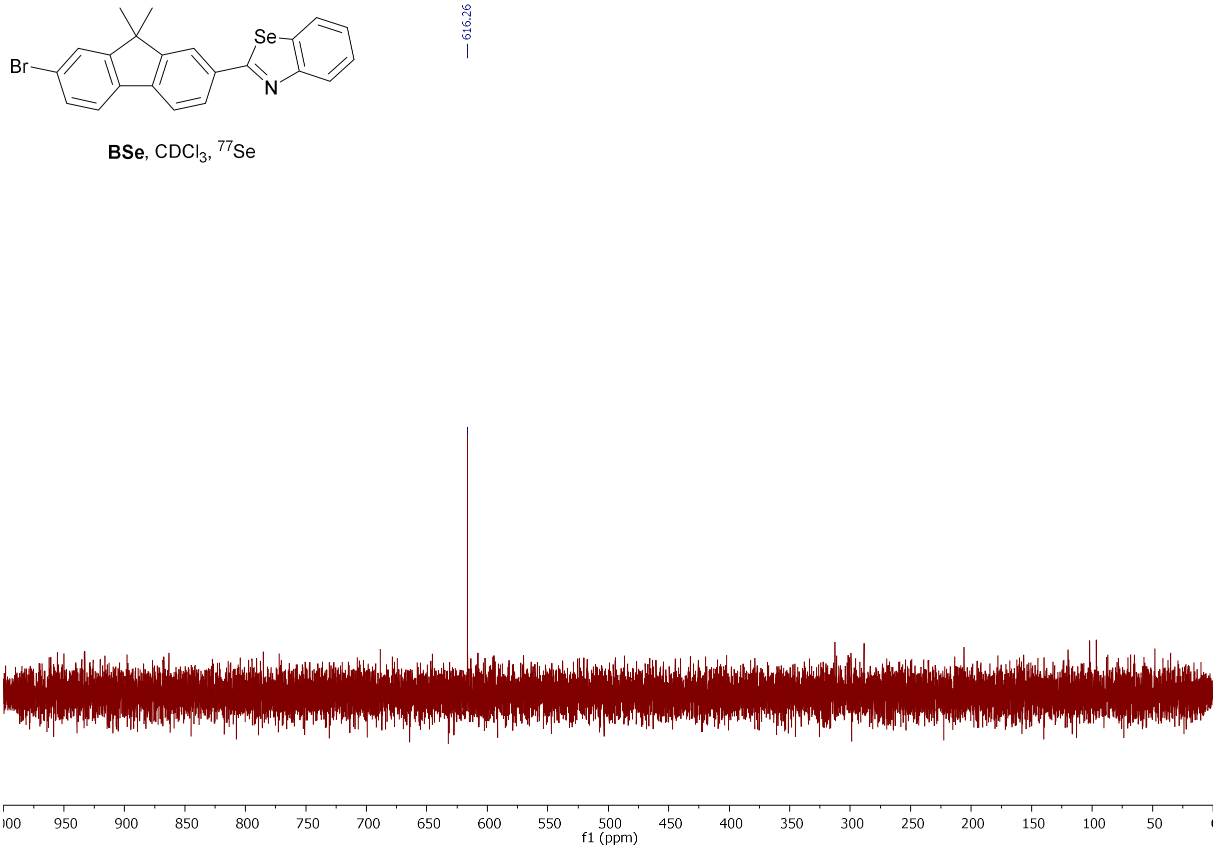

BSe, $CDCl_3$, ^{77}Se

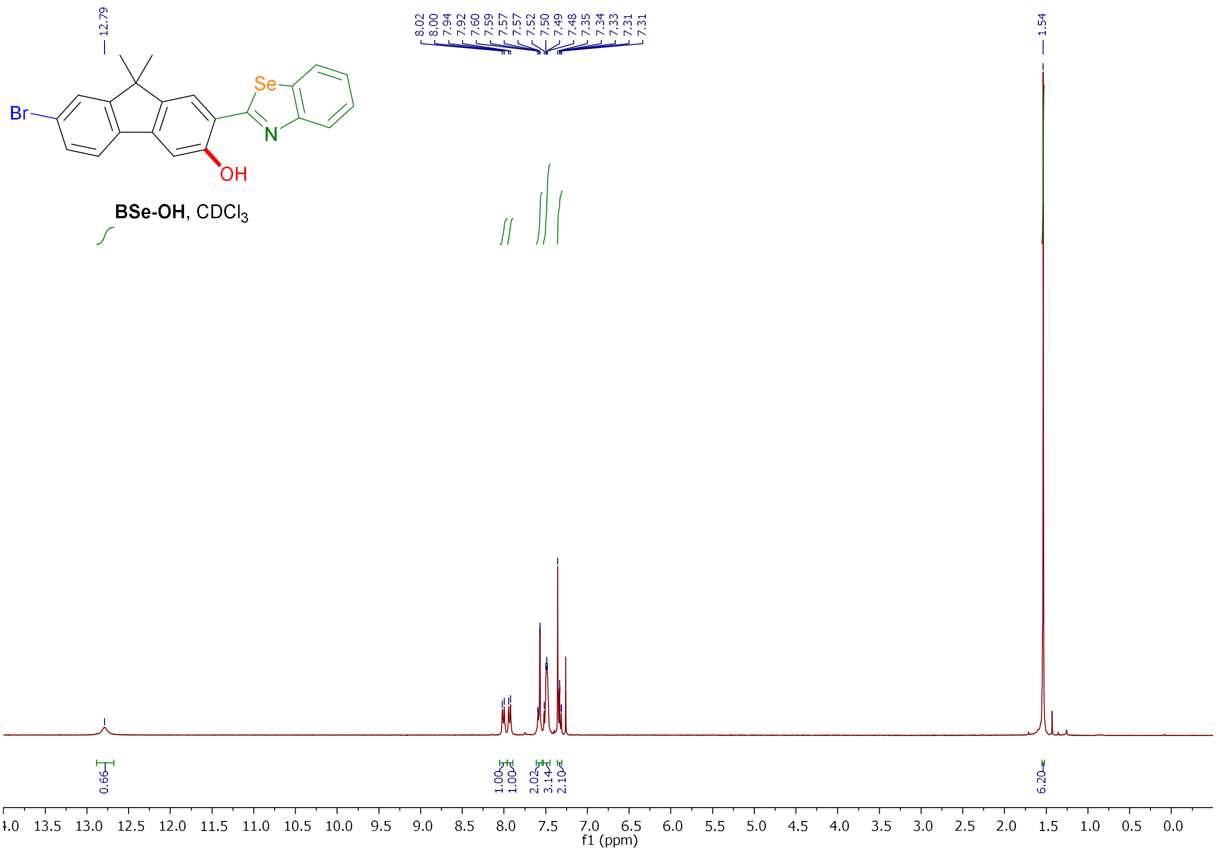
12.79
8.02
8.00
7.94
7.92
7.60
7.59
7.57
7.57
7.52
7.50
7.49
7.48
7.35
7.34
7.33
7.31
7.31
1.54
Br
Se
N
OH
BSe-OH, CDCl3
0.66
1.00
1.00
2.02
3.14
2.10
6.20
f1 (ppm)

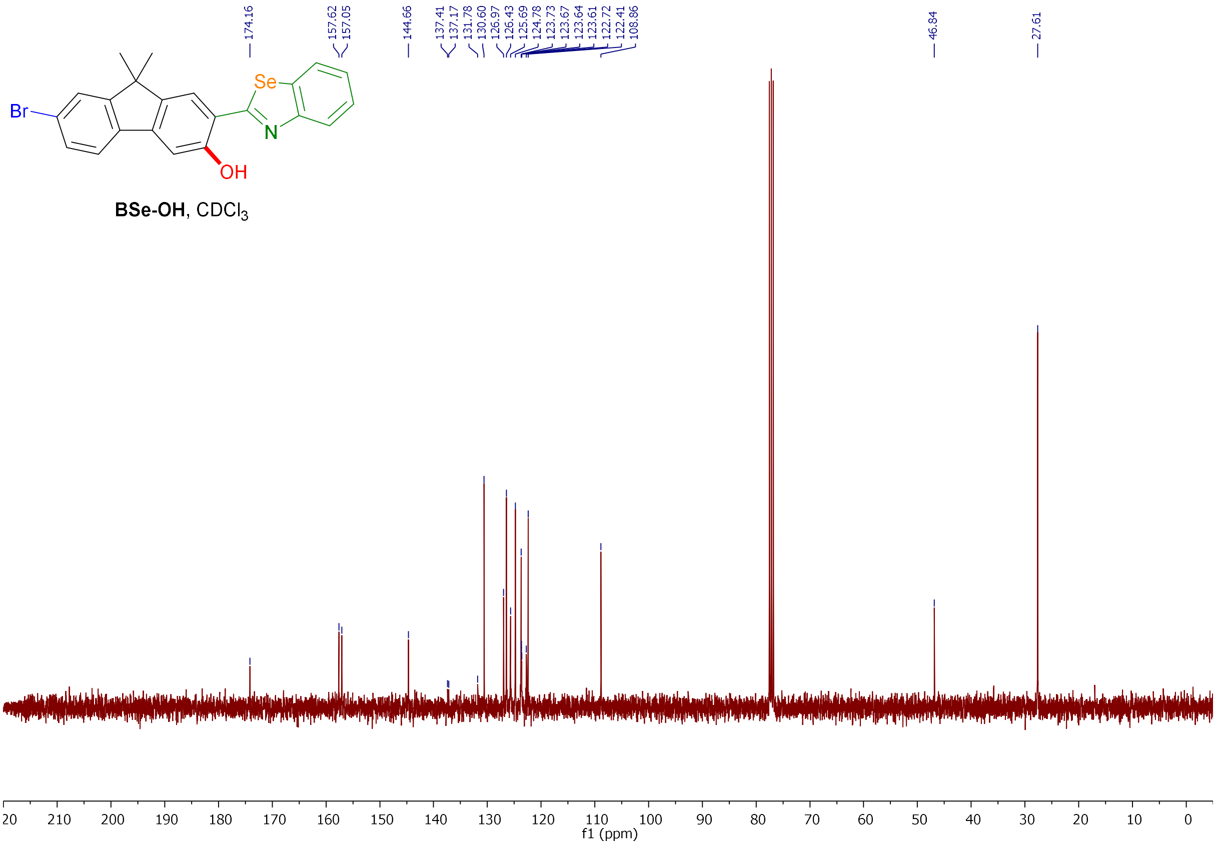
174.16
157.62
157.05
144.66
137.41
137.17
131.78
130.60
126.97
126.43
125.69
124.78
123.73
123.67
123.64
123.61
122.72
122.41
108.86
46.84
27.61
Br
Se
N
OH
BSe-OH, CDCl3
f1 (ppm)

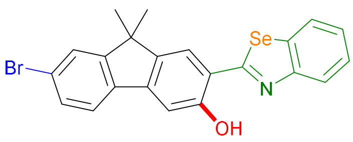

BSe-OH, $CDCl_3$, ^{77}Se

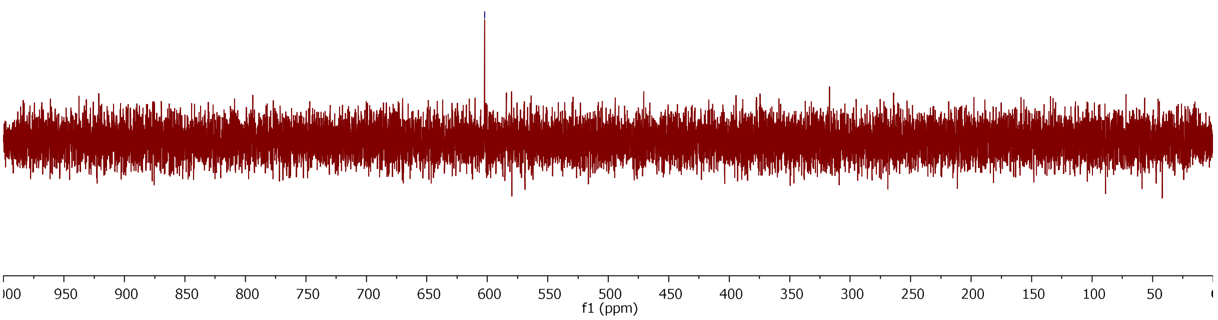

Supplement: CC-056-D0CC05780K-s001 [file CC-056-D0CC05780K-s001.pdf]
